# Iron‐Catalysed Carbene Transfer to Isocyanides as a Platform for Heterocycle Synthesis

**DOI:** 10.1002/chem.202203074

**Published:** 2022-12-20

**Authors:** Thomas R. Roose, H. Daniel Preschel, Helena Mayo Tejedor, Jasper C. Roozee, Trevor A. Hamlin, Bert U. W. Maes, Eelco Ruijter, Romano V. A. Orru

**Affiliations:** ^1^ Department of Chemistry & Pharmaceutical Sciences Amsterdam Institute for Molecular & Life Sciences Vrije Universiteit Amsterdam De Boelelaan 1108 1081 HZ Amsterdam The Netherlands; ^2^ Organic Synthesis Division Department of Chemistry University of Antwerp Groenenborgerlaan 171 B-2020 Antwerp Belgium; ^3^ Department of Organic Chemistry Aachen-Maastricht Institute for Biobased Materials (AMIBM) Maastricht University Urmonderbaan 22 6167 KD Geleen The Netherlands

**Keywords:** carbene transfer, Hieber anion, iron catalysis, isocyanide, ketenimine

## Abstract

An iron‐catalysed carbene transfer reaction of diazo compounds to isocyanides has been developed. The resulting ketenimines are trapped in situ with various bisnucleophiles to access a range of densely functionalized heterocycles (pyrimidinones, dihydropyrazolones, 1*H*‐tetrazoles) in a one‐pot process. The electron‐rich Hieber anion ([Fe(CO)_3_NO]^−^) facilitates efficient catalytic carbene transfer from acceptor‐type α‐diazo carbonyl compounds to isocyanides, providing a cost‐efficient and benign alternative to similar noble metal‐catalysed processes. Based on DFT calculations a plausible reaction mechanism for activation of the α‐diazo carbonyl carbene precursor and ketenimine formation is provided.

## Graphical abstract

**The readily available** low‐valent iron complex [Fe(CO)_3_NO]^−^ catalyzes the efficient transfer of carbenes to isocyanides. The resulting ketenimines undergo in situ cyclocondensation with various bisnucleophiles to give a broad range of medicinally relevant heterocycles. A plausible mechanism for the carbene transfer is proposed based on DFT calculations.

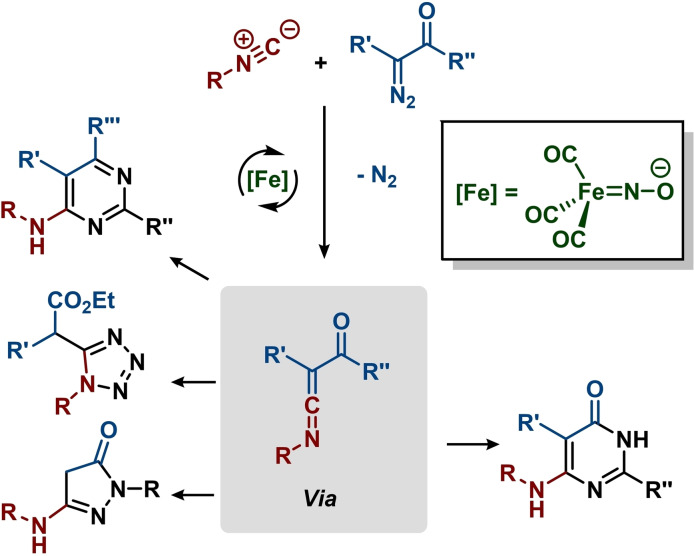

## Introduction

**Scheme 1:**
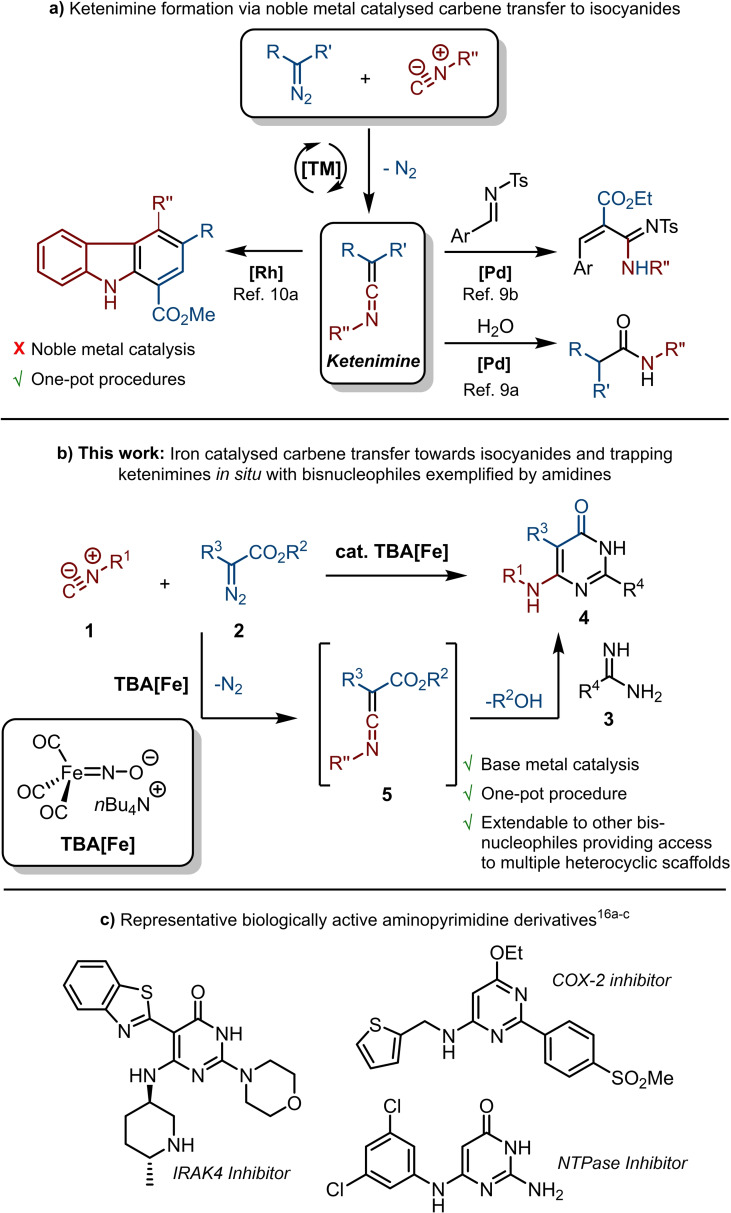
Transition metal‐catalysed carbene transfer to isocyanides and one‐pot reaction of in situ formed ketenimines with bisnucleophiles as exemplified by amidines.

Iron‐based catalysis in organic synthesis receives considerable attention,^[1]^ but still the use of noble metals remains the standard for most catalytic transformations.^[2]^ The popularity of iron has several reasons. As the first‐row transition metal (TM) in group VIII, iron has a wide range of oxidation states (‐II to +VI) that potentially display rather diverse reactivity profiles.^[2]^ Furthermore, iron has a very high abundance in the Earth crust and is relatively inexpensive to mine. This makes it an attractive metal for the development of more sustainable catalytic synthetic methodologies towards high added value chemicals.^[2]^ Also in the field of imidoylative cross‐coupling reactions,[^3^, ^4^] which employ isocyanides as versatile C1 building blocks,^[5]^ moving away from traditional noble metals towards of base metals (such as iron) seems highly attractive. A relatively underexplored reaction in this area is the TM‐catalysed carbene transfer to isocyanides (Scheme 1A). Although some isolated examples of metal‐free carbene transfer to isocyanides have been reported,^[6]^ they are limited to difluorocarbene^[6a]^ or accompanied by side reactions, such as carbene dimerization.^[6b]^ The coupling of carbenes and isocyanides results in ketenimines, which are valuable building blocks in organic synthesis.^[7]^ To date, only a handful of carbene transfer reactions to isocyanides have been reported,^[8]^ which are predominantly catalysed by noble metals, such as palladium^[9]^ and rhodium.^[10]^ Although iron complexes have been used as catalysts in carbene transfer reactions to various functionalities,[^11^, ^12^] their use in carbene transfer to isocyanides is unprecedented.^[13]^ Here we report the use of the nucleophilic ferrate complex Bu_4_N[Fe(CO)_3_NO] (TBA[Fe]),[^12^, ^14^] also referred to as the Hieber anion, as a suitable catalyst for carbene transfer to isocyanides (**1**). In this process diazo compounds (**2**) are employed as readily available carbene precursors (Scheme 1B).^[15]^ The resulting ketenimine intermediates **5** are not isolated but directly used in cyclocondensation reactions with bisnucleophiles. This results in the one‐pot synthesis of diverse, highly functionalized heterocycles, including 6‐aminopyrimidin‐4(3*H*)‐ones (**4**) (Scheme 1B), 4‐amino‐pyrimidines (**6**), 5‐amino‐2,4‐dihydro‐3*H*‐pyrazol‐3‐ones (**9**), and 1*H*‐tetrazoles (**10**). Derivatives of these aminopyrimidine scaffolds are featured in various medicinally relevant compounds[^16^, ^17^] with diverse biological activities as exemplified for aminopyrimidine derivatives in Scheme 1C.[^17a^, ^17b^, ^17c^] The occurrence of 34 aminopyrimidines in the top 200 pharmaceutical sales list of 2021 further supports the importance of this scaffold.^[18]^

## Results and Discussion

We started our investigation by optimizing the reaction of *tert*‐butyl isocyanide (**1 a**) with ethyl diazoacetate (**2 a**) (step 1) providing ketenimine **5 aa** and its subsequent reaction with benzamidine (**3 a**) (step 2) to give aminopyrimidone **4 aa** (Table 1; see Table S1 for the full optimization). Amidine **3 a** was added after 15 minutes (i. e., after complete formation of ketenimine **5 a**, see below). Inspired by iron catalysts known to facilitate carbene transfer,^[11]^ a variety of iron species with different oxidation states and appropriate additives were evaluated at 5 mol % catalyst loading (Table 1, entries 1–11). The highest yield was observed for TBA[Fe], affording **4 aa** in 88 % isolated yield (Table 1, entry 11). Exclusion of molecular sieves (4 Å) decreased the yield slightly (Table 1, entry 12). Decreasing the loading of TBA[Fe] from 5 mol % to 2.5 mol % provided a lower yield of **4 aa** (Table 1, entry 13). Running the reaction at a lower temperature (60 ^°^C) also led to diminished yield (Table 1, entry 14), and changing the solvent proved detrimental as well (Table 1, entries 15–18). Addition of **3 a** from the start of the reaction (instead of after 15 minutes reaction of **1 a** and **2 a**) led to a moderate yield and low consistency (Table 1, entry 19). The change in concentration was not the cause, as for the one‐pot procedure **4 aa** was formed in higher yield (Table 1, entry 20). The involvement of ketenimine **5 aa** in our one‐pot process was confirmed by ^1^H NMR analysis (Scheme S1). When the reaction was performed in a deuterated solvent (1,2‐CD_4_Cl_2_), full conversion of **1 a** and **2 a** after 10 minutes was observed and **5 aa** was formed in 93 % ^1^H NMR yield (Scheme S3). Interestingly, prolonged stirring for 30 minutes did not lead to any degradation of the ketenimine providing sufficient time for the addition of (and reaction with) the amidine **3** (Scheme S4).

**Table 1:** Optimisation studies for the synthesis of 6‐*t*‐butylamino‐2‐phenylpyrimidin‐4(3*H*)‐one (**4 aa**) via a one‐pot Fe‐catalysed reaction of ethyl diazoacetate (**2 a**) and *tert*‐butyl isocyanide (**1 a**) and in situ trapping of ketenimine **5 aa** by benzamidine (**3 a**).

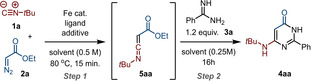					
Entry	Fe cat. [5 mol %]	Ligand [mol %]	Additive [mol %]	Solvent step 1	Yield [%]
1^[b]^	FeCl_3_	–	–	DCE	22
2^[b]^	Fe(TPP)Cl	–	–	DCE	35
3^[b]^	Fe(TPP)Cl	–	Zn (50)	DCE	40
4^[b]^	Fe(Pc)	–	–	DCE	75
5^[b]^	FeCl_2_	–	–	DCE	trace
6^[b]^	FeCl_2_	**L1** (6)	NaBAR_F_ (6)	DCE	trace
7^[b]^	Fe(ClO_4_)_2_.4H_2_O	–	–	DCE	40
8^[b]^	Fe(ClO_4_)_2_.4H_2_O	**L1** (6)	NaBAR_F_ (6)	DCE	68
9^[b]^	Fe(ClO_4_)_2_.4H_2_O	**L2** (6)	NaBAR_F_ (6)	DCE	7
10^[b]^	Fe(CO)_5_	–	–	DCE	77
11^[b]^	TBA[Fe]	–	–	DCE	92 (88)^c^
12	TBA[Fe]	–	–	DCE	81
13^[b,d]^	TBA[Fe]	–	–	DCE	74
14^[e]^	TBA[Fe]	–	–	DCE	35
15	TBA[Fe]	–	–	dioxane	57
16	TBA[Fe]	–	–	MeCN	58
17	TBA[Fe]	–	–	PhMe	54
18	TBA[Fe]	–	–	DMF	36
19^[f]^	TBA[Fe]	–	–	DCE^[g]^	65
20	TBA[Fe]	–	–	DCE^[g]^	78
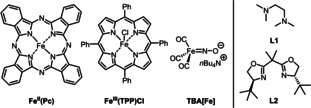					

[a] Reactions performed on a 0.5 mmol scale starting with 1.0 equiv. of **1 a** and 1.0 equiv. of **2 a** and yields determined via ^1^H NMR, using 1,3,5‐trimethoxybenzene as internal standard. [b] Addition of 4 Å MS. [c] Isolated yield. [d] 2.5 mol % catalyst [e] Reaction performed at 60 °C. [f] Benzamidine (**3 a**) added in step 1. [g] 0.25 M concentration.

**Scheme 2:**
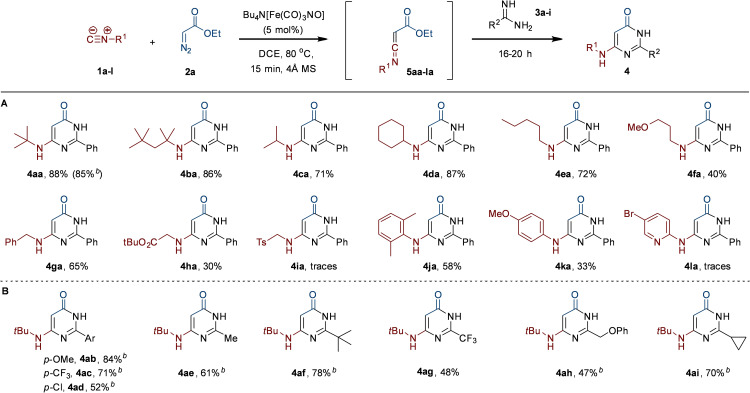
Scope regarding **A**) isocyanide (**1**) and **B**) amidine (**3**). [a] Reaction conditions: **1 a**–**l** (0.5 mmol), **2 a** (0.5 mmol), TBA[Fe] (5 mol %), DCE, 80 °C, 15 min; then **3 a**–**i** (0.6 mmol), 16–20 h. [b] Deprotonation of amidine ⋅ HCl salt with *n‐*BuLi (1.2 equiv.) in THF prior to addition.

With the optimal conditions of this one‐pot procedure in hand, we then turned our attention to determine the scope and limitations with regard to the isocyanide (Scheme 2A). Other tertiary aliphatic isocyanides, such as Walborsky's isocyanide (**1 b**), were well tolerated, affording **4 ba** in excellent yield. To our delight, primary and secondary aliphatic isocyanides were also smoothly converted to the corresponding pyrimidinones **4 ca**, **4 da**, and **4 ea** in good to excellent yields, which is typically challenging in transition metal‐catalysed reactions with these isocyanides.[^3^, ^4^]

Incorporation of an ether functionality in isocyanide **1 f** gave pyrimidinone **4 fa** in 40 % yield. Next, we investigated isocyanides featuring an activated methylene group. Benzyl isocyanide (**1 g**) underwent smooth conversion under the standard conditions, resulting in the pyrimidinone **4 ga** in good yield. However, reaction of the more acidic α‐isocyano ester **1 h** afforded **4 ha** in only moderate yield, while the use of the highly α‐acidic (and even less nucleophilic) isocyanide TOSMIC (**1 i**) gave no notable formation of **4 ia**. Aromatic isocyanides tend to be less stable compared to aliphatic isocyanides^[5f]^ and their enhanced electrophilicity often results in polymerization under TM catalysis. Consequently, they are typically poor reactants for TM‐catalysed imidoylative cross‐coupling reactions.[^3^, ^4^] In this respect, it is noteworthy that the use of 2,6‐dimethylphenyl isocyanide (**1 j**) furnishes **4 ja** in 58 % yield. Also, the less stable 4‐methoxyphenyl isocyanide (**1 k**) is converted to **4 ka**, albeit in only 33 % yield. Unfortunately, pyridyl isocyanide **1 l** proved incompatible and no formation of **4 la** was observed.

Next, we also investigated the scope and limitations with respect to the amidine input (Scheme 2B). To our delight, performing the benchmark reaction by in situ deprotonation of benzamidine⋅HCl (**3 a** ⋅ HCl) with *n‐*BuLi afforded **4 aa** in nearly identical yield as with the free benzamidine (**3 a**). In addition, pyrimidinones **4 ab**, **4 ac**, and **4 ad** were obtained in good yields with various *para‐*substituted benzamidines (**3 b**–**3 d**) with both electron‐donating and ‐withdrawing substituents. Aliphatic amidines (**3 e**–**3 i**) also proved compatible. Reaction of acetimidamide (**3 e**) and pivalimidamide (**3 f**) provided **4 ae** and **4 af** in 61 % and 78 % yield, respectively. Notably, the use of electron‐poor trifluoroacetamidine (**3 g**) in the transfer/cyclocondensation cascade **4 ag** in 48 % yield. In addition, reaction of 2‐phenoxyacetimidamide (**3 h**) and cyclopropanecarboximidamide (**3 i**) gave the corresponding heterocycles **4 ah** and **4 ai** in moderate to good yield.

**Scheme 3:**
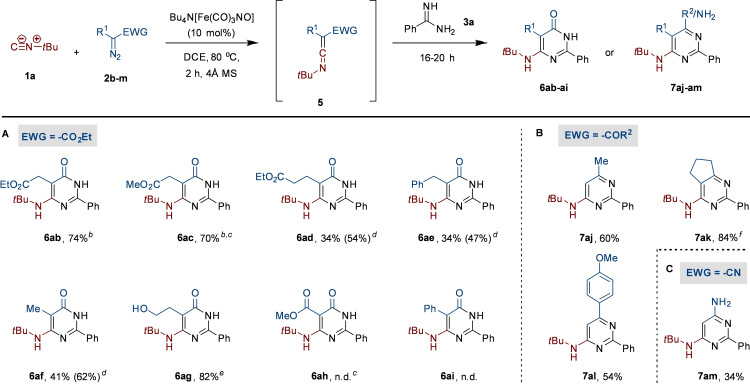
Scope regarding α‐diazo compounds (**2**). [a] Reaction conditions: **1 a** (0.5 mmol), **2 b**–**m** (0.5 mmol), TBA[Fe] (10 mol %), DCE, 80 °C, 2 h; then **3 a** (0.6 mmol), 16–20 h. [b] Use of 5 mol % TBA[Fe]. [c] Use of methyl α‐diazo ester instead of ethyl α‐diazo ester, CO_2_Me as EWG. [d] Addition of 5.0 equiv. benzamidine (**3 a**); [e] Use of α‐diazobutyrolactone as carbene precursor. [f] Use of α‐diazocyclopentanone as carbene precursor.

**Scheme 4:**
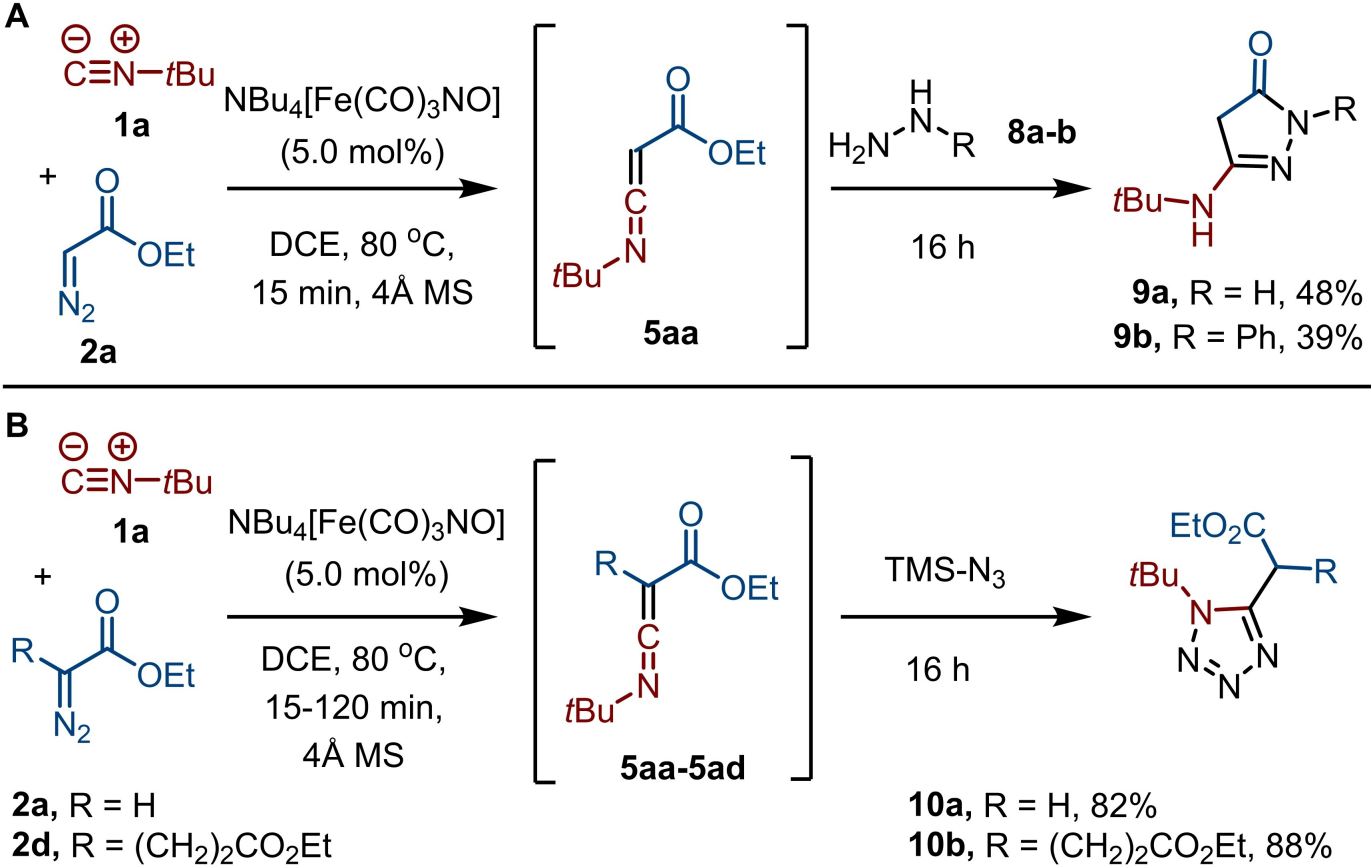
Scope regarding bisnucleophiles; a) hydrazines **8 a**–**b**; and b) TMS‐N_3._

Subsequently, we shifted our attention to the scope and limitations regarding the diazo carbene precursors **2** employing *tert*‐butyl isocyanide (**1 a**) and benzamidine (**3 a**) as the coupling partners (Scheme 3A). First, other ethyl diazoalkanoates **2 b**–**i** of the acceptor‐type carbene class were studied.^[19]^ We started with the use of diethyl 2‐diazosuccinate (**2 b**), which was converted to the corresponding ketenimine within two hours based on TLC analysis. Subsequent addition of benzamidine (**3 a**) led to the formation of **6 ab** in 74 % yield. Increasing the catalyst loading from 5 to 10 mol % did not lead to a significant improvement in yield of **6 ab** and **6 ac**. However, for products **6 ad**–**6 ag** the yield could be improved by increasing the catalyst loading to 10 mol %. Changing to the corresponding dimethyl ester **2 c** afforded **6 ac** in a similar yield, indicating no significant effect of the ester moiety on the process. However, when extending the carbon chain to diethyl 2‐diazoglutarate (**2 d**), the corresponding 6‐aminopyrimidinone **6 ad** was obtained in only moderate yield (34 %). This was also observed with simple benzyl (**6 ae**) and methyl (**6 af**) substitution of ethyl diazoacetate, i. e., the use of ethyl 3‐phenyl‐2‐diazopropanoate (**2 e**) and ethyl 2‐diazopropanoate (**2 f**). Increasing the excess of benzamidine (**3 a**) to 5 equivalents improved the yield of **6 ad** to 54 %, of **6 ae** to 47 %, and of **6 af** to 62 %, indicating that addition of the amidine to the ketenimine is the rate‐limiting step of the process. In addition, the use of α‐diazo butyrolactone (**2 g**) affords **6 ag** featuring a hydroxyethyl group in excellent yield. In contrast, acceptor‐acceptor carbenes as exemplified by dimethyl diazomalonate (**2 h**) are not compatible with our catalytic system, as **6 ah** was not formed. Although full conversion of **2 h** was observed within several hours, no successful carbene transfer to the isocyanide took place.^[20]^ Similarly, the use of donor‐acceptor class carbene precursors as exemplified by ethyl 2‐diazo‐2‐phenylacetate (**2 i**) furnished only traces of **6 ai**.^[21]^ Interestingly, also α‐diazo ketones proved compatible, providing access to 6‐alkyl or 6‐aryl substituted aminopyrimidines **7 aj**–**7 al** (Scheme 3B). α‐Diazoacetone (**2 j**) reacted smoothly to give **7 aj** in 60 % yield, while reaction of 2‐diazocyclopentanone (**2 k**) gave the corresponding fused pyrimidine **7 ak** in high yield. The aromatic α‐diazo 4‐methoxyacetophenone (**2 l**) also performs well in the carbene transfer reaction, furnishing **7 al** in 54 % yield. Furthermore, even α‐diazoacetonitrile (**2 m**) proved a suitable reaction partner to give 4‐*t*‐butylamino‐6‐amino‐2‐phenylpyrimidine (**7 am**), albeit in modest yield (Scheme 3C). Finally, to demonstrate the versatility of the process to access diverse heterocyclic scaffolds, we trapped the ketenimine with other nitrogen bisnucleophiles. Thus, reaction with hydrazine (**8 a**) afforded aminopyrazolone **9 a** in moderate yield (Scheme 4A). In addition, reaction with phenylhydrazine (**8 b**) furnished **9 b** in moderate yield as a single regioisomer, with the more nucleophilic unsubstituted nitrogen of the hydrazine moiety attacking the ketenimine carbon first. Interestingly, the 2,4‐dihydro‐3H‐pyrazol‐3‐one scaffold (**9**) is present in numerous bioactive compounds.^[22]^ In addition, the ketenimine also smoothly undergoes 1,3‐dipolar cycloadditions without intermediate isolation. Accordingly, the use of TMS‐N_3_ as an HN_3_ surrogate afforded 1*H*‐tetrazoles **10 a** and **10 b** in excellent yields when using ethyl diazoacetate (**2 a**) and diethyl 2‐diazoglutarate (**2 d**), respectively (Scheme 4B). The high yield of **10 b** further supports our hypothesis that the moderate yield of **6 ad** (Scheme 3) is not due to poor ketenimine formation, but rather to inefficient nucleophilic addition of the amidine. Tetrazoles have found widespread use in medicinally relevant compounds.^[23]^

**Scheme 5:**
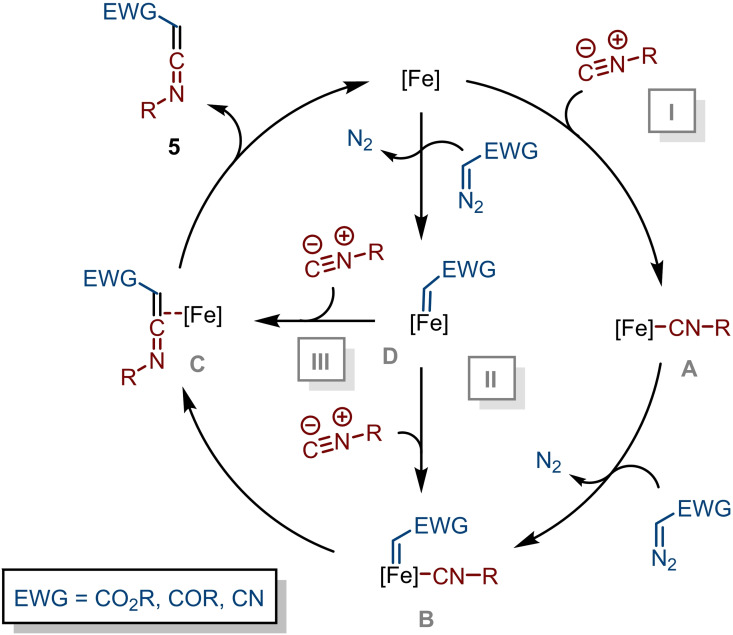
Plausible mechanistic pathways for the Fe‐catalysed carbene transfer of diazo compounds **1** to isocyanides **2** providing ketenimines **5**.

From a mechanistic point of view, three distinct pathways for the mechanism of the Hieber anion‐catalysed ketenimine formation (Scheme 5) seem plausible.^[8]^ These three pathways are discussed in more detail below and are based on theoretical studies on general carbene transfer reactions^[24]^ as well as previous theoretical and spectroscopic studies[^25^, ^26^, ^27^] on the Hieber anion, in combination with mechanistic insight gathered from the palladium‐catalysed carbene transfer to isocyanides.^[9c]^ The first pathway (I) commences with the formation of Fe‐isocyanide complex **A**, followed by formation of the corresponding Fe‐isocyanide/carbene complex **B**, which after 1,1‐migratory insertion affords iron‐coordinated ketenimine complex **C**. Subsequent or simultaneous liberation of ketenimine **5** closes the catalytic cycle. Alternatively, intermediate **B** can be reached via carbene complex **D** (pathway II), where the carbene complex forms prior to isocyanide coordination. Pathways I and II both rely on an inner‐sphere mechanism involving migratory insertion of a coordinated isocyanide moiety. A third possibility is the direct intermolecular attack of the isocyanide on the electrophilic carbene center in complex **D** (pathway III). This outer‐sphere mechanism is commonly observed in, e. g., carbene transfer transformations involving iron^[28]^ and cobalt^[29]^ porphyrin‐based catalysts that lack suitably located coordination sites.

We explored these three potential pathways for the carbene transfer from α‐diazo esters to isocyanides in more detail using DFT calculations to identify the most viable route. Based on previous calculations^[27]^ of reaction pathways involving [Fe(CO)_3_NO]^−^, we employed the COSMO(1,2‐DCE)‐ZORA‐BP86‐ D3(BJ)/TZ2P level of theory throughout this work (see Section S6.1 for detailed description of computational methods). To our delight, bond distances and bond angles of the optimized structure of [Fe(CO)_3_NO]^−^ were in good accordance with previous calculations^[25]^ and the reported crystal structure^[30]^ (see Tables S2a–c). For the DFT calculations methyl α‐diazoacetate (**R2**) and methyl isocyanide (**R3**) were chosen as reactants. Generally, isocyanides are suitable ligands for TM complexes.^[31]^ We envisioned that isocyanide complex formation could proceed in an associative or dissociative fashion (see Scheme S5).

**Figure 1:**
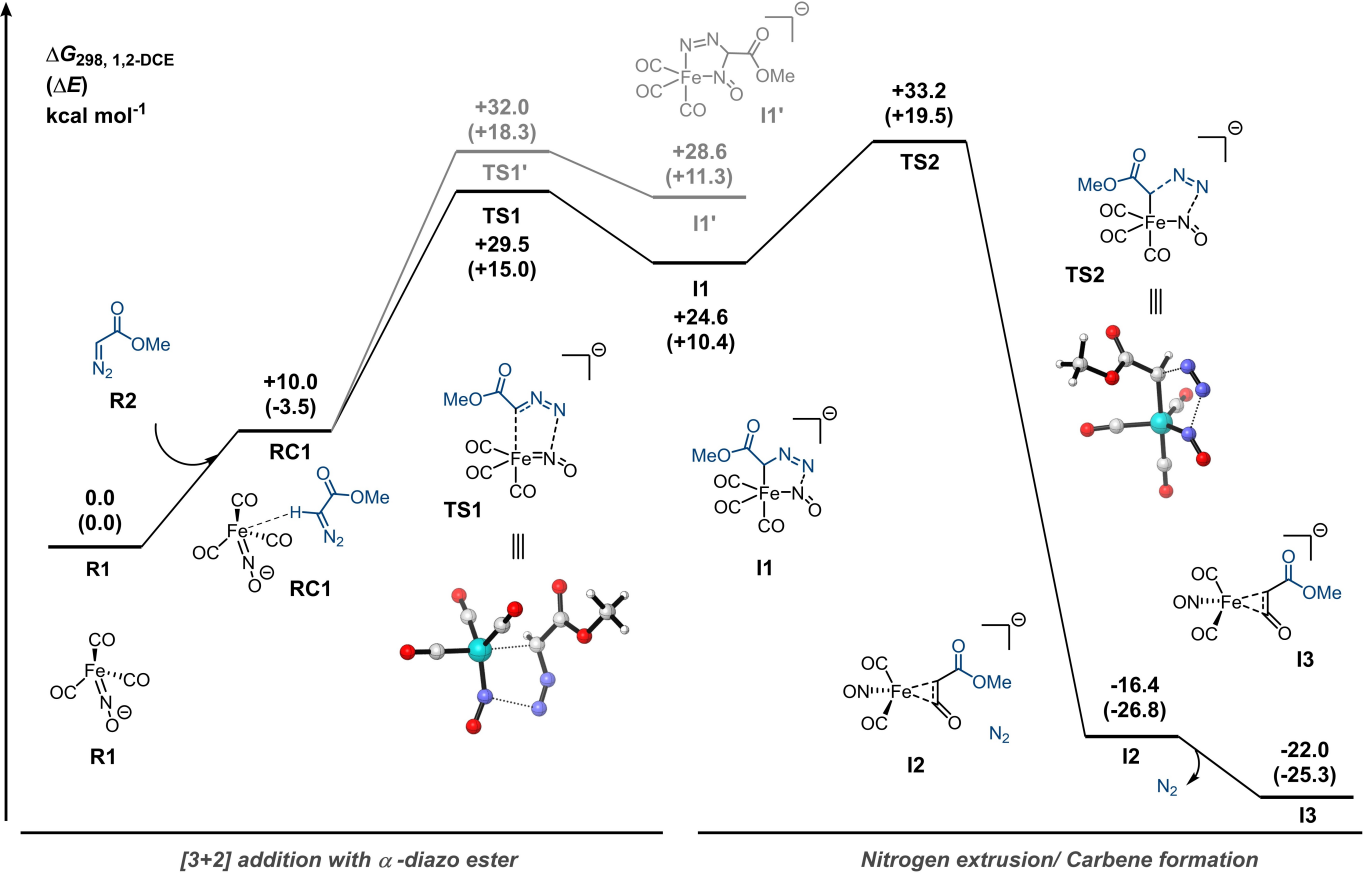
Gibbs free energy (in kcal mol^−1^) reaction profile for the reaction of α‐diazoacetate (**R2**) with [Fe(CO)_3_NO]^−^ (**R1**) with nitrogen extrusion computed at COSMO(1,2‐DCE)‐ZORA‐BP86‐D3(BJ)/TZ2P.

**Figure 2:**
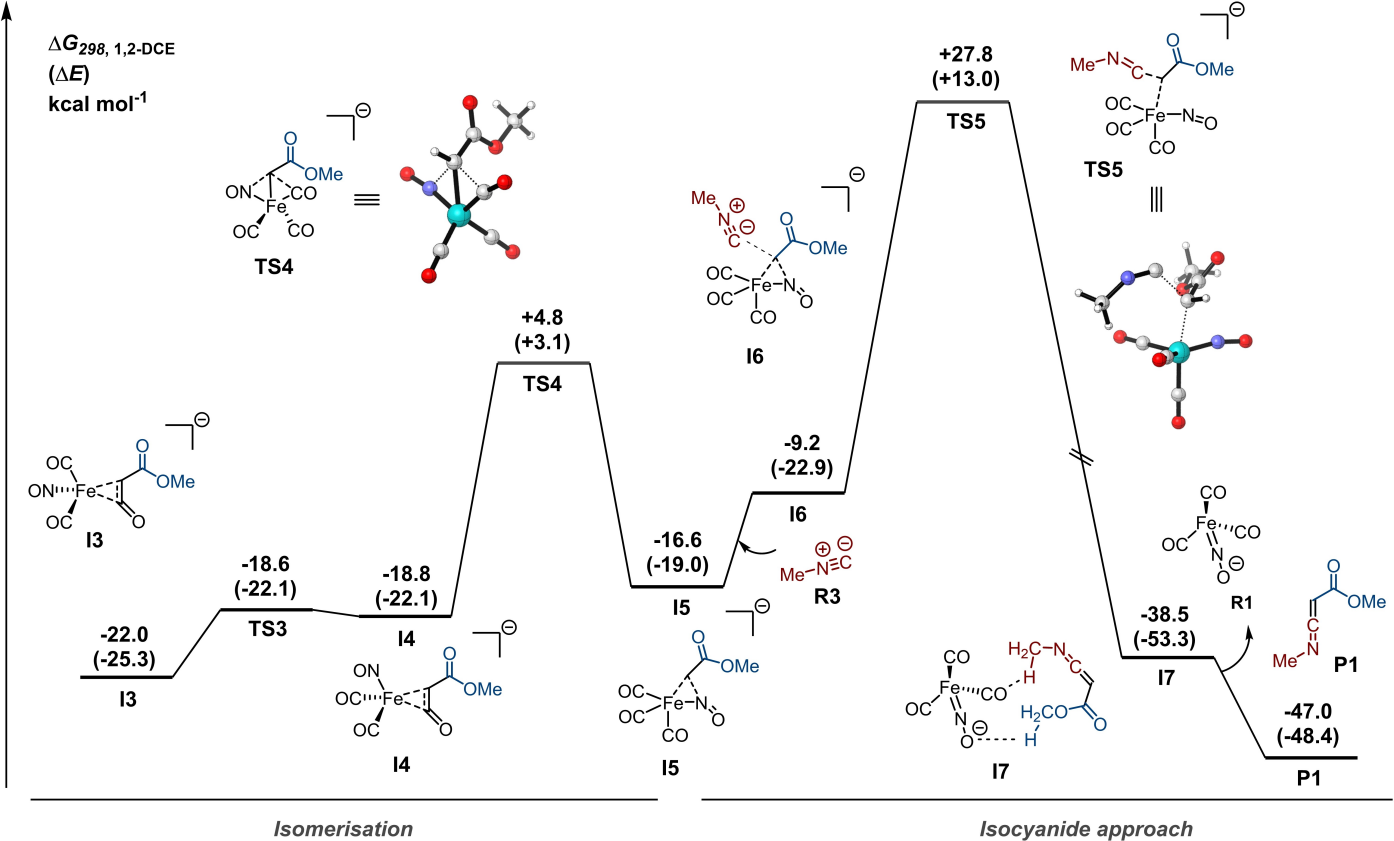
Continuation of Gibbs free energy (in kcal mol^−1^) reaction profile for the carbene transfer of **I3** to methyl isocyanide (**R3**) computed at COSMO(1,2‐DCE)‐ZORA‐BP86‐D3(BJ)/TZ2P.

However, the energy of the mono‐isocyanide complex; [Fe(CO)_2_(CNMe)NO]^−^ (**P2**), along the potential energy surface PES is higher (Δ*G*=10.2 kcal mol^−1^) than that of the [Fe(CO)_3_NO]^−^ fragment (**R1**) (see Scheme S5). In addition, formation of the intermediates associated with both the dissociative (**I10**) and associative pathways (**I8** and **I9**) towards the mono‐isocyanide complex **P2** are even more endergonic (see Scheme S5). Furthermore, given that the Fe center in [Fe(CO)_3_NO]^−^ is relatively electron‐rich and isocyanides are strong σ‐donors and weak π‐acceptors compared to CO, formation of mono‐isocyanide complex prior to the reaction with methyl α‐diazoacetate (**R2**) is unlikely. Moreover, facilitating isocyanide complex formation by addition of *p*‐nitro‐anisole (10 mol %) as a decarbonylation agent^[12c]^ to the benchmark reaction (see Table S1, entry 15) resulted in a lower yield of **4 aa** (69 %), indicating less facile ketenimine formation. Combined, these findings suggest that the reaction most likely does not proceed via pathway I (Scheme 5). Next, we investigated the interaction of [Fe(CO)_3_NO]^−^ (**R1**) with methyl α‐diazoacetate (**R2**) (Figure 1). Despite little consensus in the literature about the physical oxidation state of the Fe centre of Fe(CO)_3_NO]^−^,[^25^, ^26^] a previous theoretical study on the role of the Hieber anion in the Cloke‐Wilson rearrangement indicates that the nitrosyl group can act as a non‐innocent ligand via two electron reduction/oxidation during the catalytic cycle, and the Fe=N−O moiety is considered to be of significant importance for its catalytic activity.^[27a]^ In addition, detailed computational and spectroscopic studies by Plietker suggest the Hieber anion is best represented as an Fe(0) species with the negative charge predominantly localized on the nitrosyl group, where the iron centre is anti‐ferromagnetically coupled to the NO ligand via two π‐bonds.^[25]^ Interestingly, the search for an energy minimum corresponding to interaction between **R1** and **R2** resulted in [3+2]‐cycloadduct **I1** (Figure 1). In principle, **R2** could interact with the Fe=N−O moiety in two ways, and both pathways to the distinct regioisomeric [3+2]‐adducts were located on the PES, with **I1** being both kinetically and thermodynamically favoured over **I1′**. From intermediate **I1** onward, extrusion of nitrogen towards **I3** via **I2** was found to be highly beneficial. Starting directly from **I3**, no pathway for the isocyanide approach could be located (Figure 2). In addition, CO dissociation from intermediate **I3** tends to be highly endergonic (Scheme S5), indicating that ligand exchange with an isocyanide is unlikely, thereby disfavoring Pathway II (Scheme 5). Gratifyingly, we were able to find a rotational isomer **I4** on the PES, which had a low barrier for rotation (**TS3**; Δ*G* ^≠^=3.4 kcal mol^−1^, Figure 2), followed by subsequent intramolecular transfer of the carbene fragment to the NO moiety via **TS4** resulting in pseudo‐trigonal bipyramidal intermediate **I5**.

Intrinsic reaction coordinate (IRC) calculations unambiguously connected complex **I4** and **I5** via **TS4 (**Δ*G* ^≠^=23.6 kcal mol^−1^). From **I5** onward, a trajectory for the isocyanide to approach was found along the PES. The outer‐sphere addition of the isocyanide to the carbene carbon via a surprisingly high energy barrier **TS5** (Δ*G* ^≠^=+37.0 kcal mol^−1^) results in **I7**. Subsequent product release from **I7** furnishes the ketenimine **P1** and regenerates the presumed catalytic species, [Fe(CO)_3_NO]^−^ (**R1**). Overall, the carbene transfer towards isocyanides from α‐diazo esters leading to the formation of ketenimine **P1** is highly exergonic. The presented DFT studies shed some light on the potentially preferred pathway involving the [Fe(CO)_3_NO]^−^ species and provides support for pathway III being operative with a non‐innocent role of the NO ligand of the Hieber anion in the activation step of the α‐diazo compound (Scheme 5).

## Conclusion

We disclosed the first ketenimine synthesis via iron‐catalysed carbene transfer reaction to isocyanides. The ferrate complex *n*Bu_4_N[Fe(CO)_3_NO], formally featuring a negative oxidation state, was identified as a highly efficient catalyst for this reaction. Trapping the versatile ketenimine products with bisnucleophiles provides access to various valuable heterocycles in a one‐pot process. Cyclocondensation with amidines afforded substituted 6‐aminopyrimid‐4(3*H*)‐ones. The ketenimine can be trapped by other nucleophiles as well, as exemplified by hydrazines, affording substituted 5‐amino‐2,4‐dihydro‐3*H*‐pyrazol‐3‐ones, and by TMS‐N_3_ to unlock 1*H*‐tetrazoles via formal cycloaddition. The reactions tolerate a broad range of different isocyanides and carbenes, obtained from the corresponding readily available acceptor‐type diazo compounds. Generally moderate to excellent yields are obtained considering that the one‐pot process involves two consecutive reactions. DFT studies on the transfer of carbene to isocyanide reveal the pathway involving an outer‐sphere attack of the isocyanide to the pre‐formed [Fe]‐carbene species and a non‐innocent role for the NO ligand in the activation step of the diazo compound to be the most plausible mechanism for ketenimine formation.

## Experimental Section

To a flame dried Schlenk flask under N_2_ atmosphere, charged with 4 Å MS and a stirring bar, was added TBA[Fe] (10.3 mg, 0.025 mmol, 0.05 equiv.). Subsequently, 1,2‐DCE (1.0 mL) was added and the mixture was stirred until the catalyst was dissolved. This was followed by the addition of α‐diazo compound **2** (0.5 mmol, 1.0 equiv) and isocyanide **1** (0.5 mmol, 1.0 equiv.). The solution was placed in a pre‐heated oil bath and stirred for 15–120 minutes at 80 °C. Subsequently, amidine **3** (0.6 mmol, 1.2 equiv.) was added as a solution in 1,2‐DCE (1.0 mL). The reaction was allowed to stir overnight at 80 °C. Subsequently, the reaction mixture was filtered through a pad of silica using 5 % MeOH in CH_2_Cl_2_ as eluent. The filtrate was collected and concentrated *in vacuo*. The crude product was purified by flash column chromatography (MeOH/CH_2_Cl_2_) to afford **4**.

## Conflict of interest

The authors declare no conflict of interest.

1

## Supporting information

As a service to our authors and readers, this journal provides supporting information supplied by the authors. Such materials are peer reviewed and may be re‐organized for online delivery, but are not copy‐edited or typeset. Technical support issues arising from supporting information (other than missing files) should be addressed to the authors.

Supporting Information

## Data Availability

The data that support the findings of this study are available in the supplementary material of this article.
