# Supplementary material for: Iron‐Catalysed Carbene Transfer to Isocyanides as a Platform for Heterocycle Synthesis
Source: Chemistry. 2022 Dec 20;29(9):e202203074. doi: 10.1002/chem.202203074 (PMC10108253; doi:10.1002/chem.202203074)

Chemistry–A European Journal

Supporting Information

Iron-Catalysed Carbene Transfer to Isocyanides as a Platform for Heterocycle Synthesis

Thomas R. Roose, H. Daniel Preschel, Helena Mayo Tejedor, Jasper C. Roozee, Trevor A. Hamlin,* Bert U. W. Maes,* Eelco Ruijter,* and Romano V. A. Orru*

Table of Contents

1. General information **1**

2. Optimization data: One-Pot Fe-catalysed carbene transfer to isocyanides followed by cyclocondensation **2**

3. Ketenimine formation **4**

3.1 Observation of ketenimine in crude reaction mixture 4

3.2 Quantitative ^{1}H-NMR analysis of ketenimine formation 5

4. Synthesis of starting materials **8**

5. Scope data **10**

5.1 General procedure free amidine 10

5.2 General procedure amidine HCl salt 10

6. Attempted bis-nucleophiles **18**

7. Computational studies **20**

7.1 Computational methods 20

7.2 Computational results 21

7.2.1 Benchmark studies 21

7.2.2 Isocyanide complex & Iron-carbene formation 21

7.2.3 Isomerization and isocyanide approach 22

7.2.4 Entire PES of proposed pathway 23

7.3 Computational details 23

8. References **36**

9. ^{1}H- & ^{13}C-NMR spectra **37**

1. General information

Unless stated otherwise, all solvents and commercially available reagents were used as purchased. Anhydrous dichloromethane, THF, DMF and toluene were obtained by an Inert Solvent Purification System. All other solvents were used as purchased. Nuclear magnetic resonance (NMR) spectra were recorded on a Bruker Avance 600 (150.1 MHz for ^{13}C), Bruker Avance 500 (125.7 MHz for ^{13}C) & (470.4 MHz for ^{19}F) or Bruker Avance 300 (83.85 MHz for ^{13}C) using the residual solvent as internal standard (^{1}H: δ 7.26 ppm, ^{13}C {^{1}H}: δ 77.16 ppm for $CDCl_3$, ^{1}H: δ 2.50 ppm, ^{13}C{^{1}H}: δ 39.52 ppm for DMSO-d6). Chemical shifts (δ) are given in ppm and coupling constants (J) are quoted in hertz (Hz). Resonances are described as s (singlet), d (doublet), t (triplet), q (quartet), quint (quintet), sex (sextet), sep (septet), br (broad singlet) and m (multiplet) or combinations thereof. Infrared (IR) spectra were recorded neat using a Shimadzu FTIR-8400s spectrophotometer and wavelengths are reported in cm^{-1}. Electrospray Ionization (ESI) high-resolution mass spectrometry (HRMS) was carried out using a Bruker microTOF-Q instrument in positive ion mode (capillary potential of 4500 V). Flash chromatography was performed on Silicycle Silia-P Flash Silica Gel (particle size 40-63 μm, pore diameter 60Å) using the indicated eluent. Thin Layer Chromatography (TLC) was performed using TLC plates from Merck (SiO_2, Kieselgel 60 F254 neutral, on aluminium with fluorescence indicator) and compounds were visualized by UV detection (254 nm) and $KMnO_4$ stain. SFC-MS analysis was conducted using a Shimadzu Nexera SFC-MS equipped with a Nexera X2 SIL-30AC autosampler, Nexera UC LC-30AD SF CO_2 pump, Nexera X2 LC-30AD liquid chromatograph, Nexera UC SFC-30A back pressure regulator, prominence SPD-M20A diode array detector, prominence CTO-20AC column oven and CBM-20A system controller. A gradient of supercritical CO_2 (A) and methanol (B) was used. Method: 2% B/98% A ◊ 100% B/0% A over the course of 7 min. The flow was maintained at 2.0 mL/min and the sample injection volume was 5 μL. Mass spectrometry analyses were performed using a Shimadzu LCMS-2020 mass spectrometer. The data were acquired in full-scan APCI mode (MS) from m/z 100 to 800 in positive ionisation mode. Data was processed using Shimadzu Labsolutions 5.82.

2. Optimization data: One-Pot Fe-catalysed carbene transfer to isocyanides followed by cyclocondensation

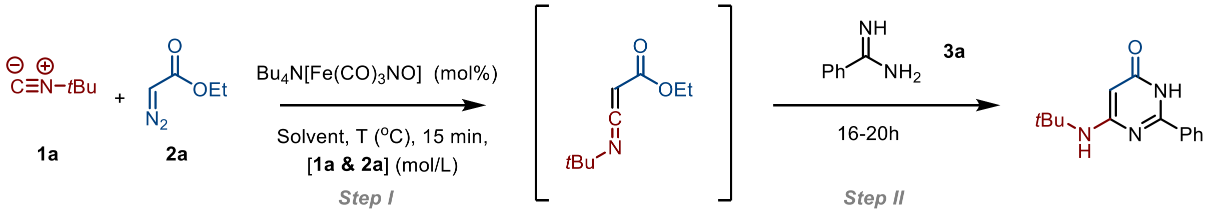

To a flame dried Schlenk flask under N_2 atmosphere, charged with a stirring bar, was added Catalyst (0.025 mmol, 0.05 equiv) and respective additive(s). Subsequently, the solvent (1.0 mL) was added, and the mixture was stirred until the catalyst was dissolved. This was followed by the addition of *tert*-butyl isocyanide (**1a**) (0.5 mmol, 1.0 equiv., 56.6 µL) and ethyl diazoacetate (**2a**) (0.5 mmol, 1.0 equiv, 62.0 µL). The solution was placed in a pre-heated oil bath and stirred for 10-30 minutes at 80 °C. Subsequently, benzamidine (**3a**) (0.6 mmol, 1.2 equiv., 72.1 mg) was added as a solution in Solvent (1.0 mL). The reaction was allowed to stir overnight at 80 °C. Subsequently, the reaction mixture was filtered through a pad of silica using 5% MeOH in CH_2Cl_2 as eluent. The filtrate was collected and concentrated *in vacuo*. All reactions were performed on a 0.5 mmol scale and yields were determined via ^{1}H-NMR, using 1,3,5-trimethoxybenzene as internal standard.

Table S1. Optimization data Iron-catalyzed carbene transfer of **1a** tot **2a** followed by cyclocondensation with **3a**.

Entry	Catalyst (mol %)	Additive(s) (mol %)	Solvent	T (°C)	Equiv (1a)	Equiv (2a)	Equiv (3a)	[1 & 2] (mol/L)	Yield (%)[a]
1[b]	TBA[Fe] (5)	-	1,2-DCE	80	1.0	1.0	1.2	0.25	65
2[b]	TBA[Fe] (5)	-	1,2-DCE	80	1.2	1.2	1.0	0.25	64
3	TBA[Fe] (5)	-	1,2-DCE	80	1.0	1.0	1.2	0.25	78
4	TBA[Fe] (5)	-	1,2-DCE	80	1.0	1.0	1.2	0.5	81
5	TBA[Fe] (5)	-	1,4-dioxane	80	1.0	1.0	1.2	0.5	57
6	TBA[Fe] (5)	-	MeCN	80	1.0	1.0	1.2	0.5	58
7	TBA[Fe] (5)	-	PhMe	80	1.0	1.0	1.2	0.5	54
8	TBA[Fe] (5)	-	1,2-DCE	60	1.0	1.0	1.2	0.5	35
9	TBA[Fe] (5)	-	1,2-DCE	80	1.0	1.0	2.0	0.5	78
10	TBA[Fe] (5)	-	1,2-DCE	80	1.0	1.0	1.05	0.5	74
11[d)]	TBA[Fe] (5)	-	1,2-DCE	80	1.0	1.0	1.2	0.5	92 (88)[c)]
12[d)]	TBA[Fe] (2.5)	-	1,2-DCE	80	1.0	1.0	1.2	0.5	74
13	TBA[Fe] (5)	-	1,2-DCE	80	1.2	1.2	1.0	0.5	70
14[e)]	TBA[Fe] (5)	-	1,2-DCE	80	1.0	1.0	1.2	0.5	80
15	TBA[Fe] (5)	*p*-NO_2- PhOMe (10)	1,2-DCE	80	1.0	1.0	1.2	0.5	69
16[d)]	$Fe^{0}(CO)_5$ (5)	-	1,2-DCE	80	1.0	1.0	1.2	0.5	77
17[d)]	Fe^{II}(Pc) (5)	-	1,2-DCE	80	1.0	1.0	1.2	0.5	75
18[d)]	Fe^{III}(TPP)Cl (5)	-	1,2-DCE	80	1.0	1.0	1.2	0.5	35

**19**[d)]	Fe^{III} (TPP)Cl (5)	Zn (50)	1,2-DCE	80	1.0	1.0	1.2	0.5	40
**20** [d)]	$Fe^{III}Cl_3$ (5)	-	1,2-DCE	80	1.0	1.0	1.2	0.5	22
**21** [d)]	$Fe^{III}Cl_3$ (5)	Phen (6) + $NaBAR_F$ (15)	1,2-DCE	80	1.0	1.0	1.2	0.5	trace
**22** [d)]	$Fe^{II}Cl_2$ (5)	-	1,2-DCE	80	1.0	1.0	1.2	0.5	trace
**23** [d)]	$Fe^{II}Cl_2$ (5)	TMEDA (6) + $NaBAR_F$ (6)	1,2-DCE	80	1.0	1.0	1.2	0.5	trace
**24** [d)]	$Fe^{II}Cl_2$ (5)	DPPE (5)	1,2-DCE	80	1.0	1.0	1.2	0.5	trace
**25** [d)]	$Fe^{II}(ClO_4)_2.4H_2O$ (5)	-	1,2-DCE	80	1.0	1.0	1.2	0.5	40
**26** [d)]	Fe^{II} $(ClO_4)_2.4H_2O$ (5)	TMEDA (6) + $NaBAR_F$ (6)	1,2-DCE	80	1.0	1.0	1.2	0.5	68
**27** [d)]	Fe^{II} $(ClO_4)_2.4H_2O$ (5)	Bisoxaline (6) + $NaBAR_F$ (6)	1,2-DCE	80	1.0	1.0	1.2	0.5	7

[a] Reactions performed on a 0.5 mmol scale and yields determined via ^{1}H-NMR, using 1,3,5-trimethoxybenzene as internal standard.

[b] Addition of benzamidine at start of the reaction.

[c] Isolated yield.

[d] Addition of 4Å MS.

[e] reaction performed in the dark.

3. Ketenimine formation

3.1 Observation of ketenimine in crude reaction mixture

The involvement of the ketenimine was proven by ^{1}H-NMR analysis of the crude reaction mixture prior to the addition of amidine **3** (Scheme S7). The synthesis of C-ethoxycarbonyl-*N*-t-butylketenimine (**5aa**) via Hieber-anion catalyzed coupling of ethyl diazoacetate (**2a**) and *tert*-butyl isocyanide (**1a**) at a 0.5 mmol scale was elected as model.

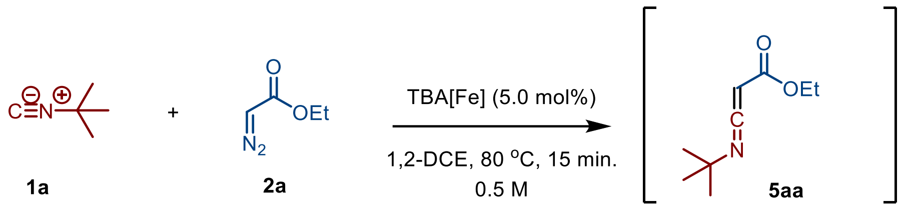

To a flame dried Schlenk flask under N_2 atmosphere, charged with a stirring bar, was added TBA[Fe] (10.3 mg, 0.025 mmol, 0.05 equiv.). Subsequently, 1,2-DCE (1.0 mL) was added, and the mixture was stirred until the catalyst was dissolved. This was followed by the addition of α-diazo compound (**2a**) (0.5 mmol, 1.0 equiv, 62.0 µL) and isocyanide **1a** (0.5 mmol, 1.0 equiv., 56.6 µL). After stirring the reaction mixture for 30 min. The sample was diluted with $CDCl_3$, filtered, transferred to a NMR tube and directly measured (Scheme S7). 1,3,5-trimethoxybenzene ((1,3,5-TMB (0.5 mmol/ 3 (normalized to 1H)) was used as internal standard and added to the reaction mixture prior to heating. ^{1}H-NMR matches that of the one reported in literature. [11]

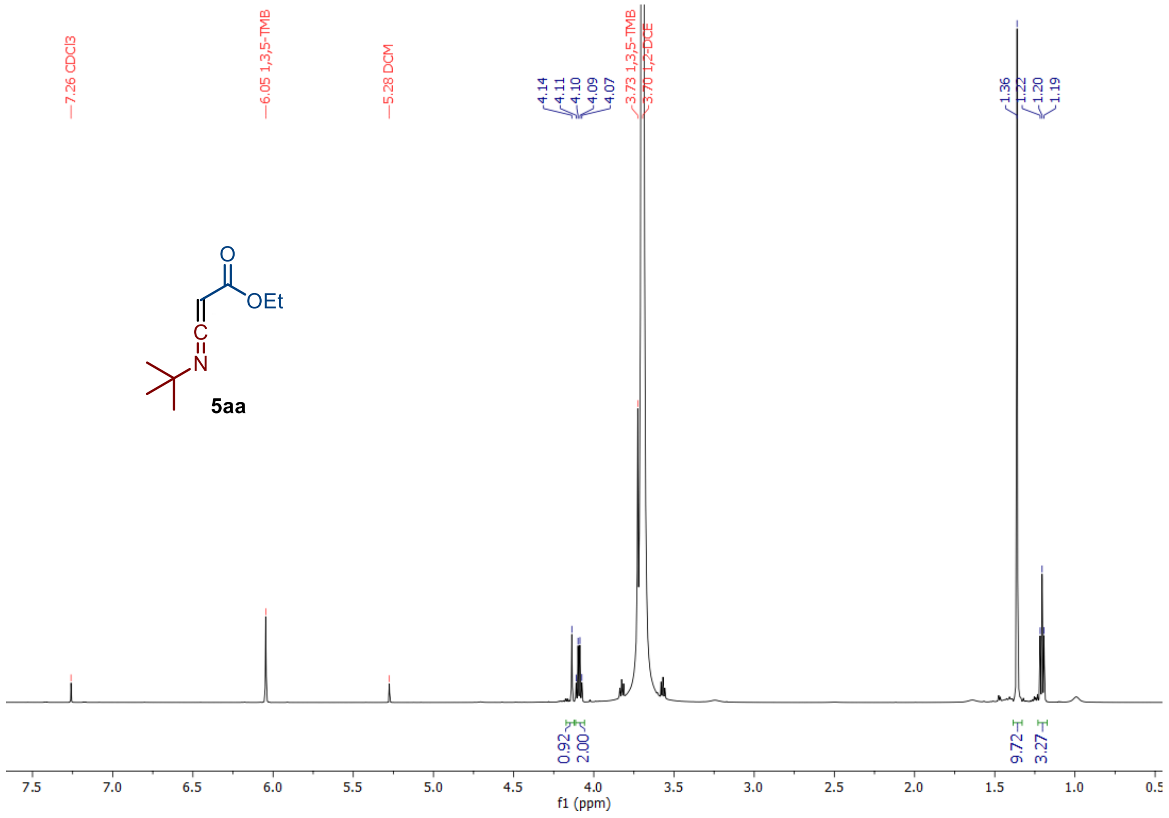

Scheme S1: ^{1}H-NMR spectrum in $CDCl_3$ of diluted sample of reaction mixture after stirring for 30 min. at 80 °C. 1,3,5-trimethoxybenzene (1,3,5-TMB) present as internal standard.

3.2 Quantitative ^{1}H-NMR analysis of ketenimine formation

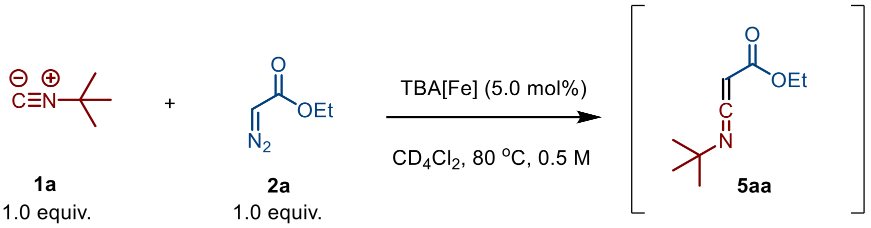

After identification of ketenimine **5aa**, the reaction was performed in CD_4Cl_2 at a 0.375 mmol scale, using 1,3,5-trimethoxybenzene as internal standard in order to quantify the yield of ketenimine formation. Samples were taken at the given time to get an indication of the reaction time at the given conditions (Scheme S9-S11). The sample was diluted with CD_2Cl_2, filtered, transferred to a NMR tube and directly measured. 1,3,5-trimethoxybenzene (normalized to 1H) was used as internal standard and added to the reaction mixture prior the start of the reaction. Both reactants could readily be observed after 2 minutes (Scheme S9). After 10 minutes no reactants remained and ketenimine **5aa** is formed (Scheme S10). In addition, no degradation was observed after 30 min (Scheme S11). Isolated ^{1}H-NMR signals of ketenimine **5aa** at δ 4.15 ppm and δ 4.10 ppm were used to determine internal standard yield.

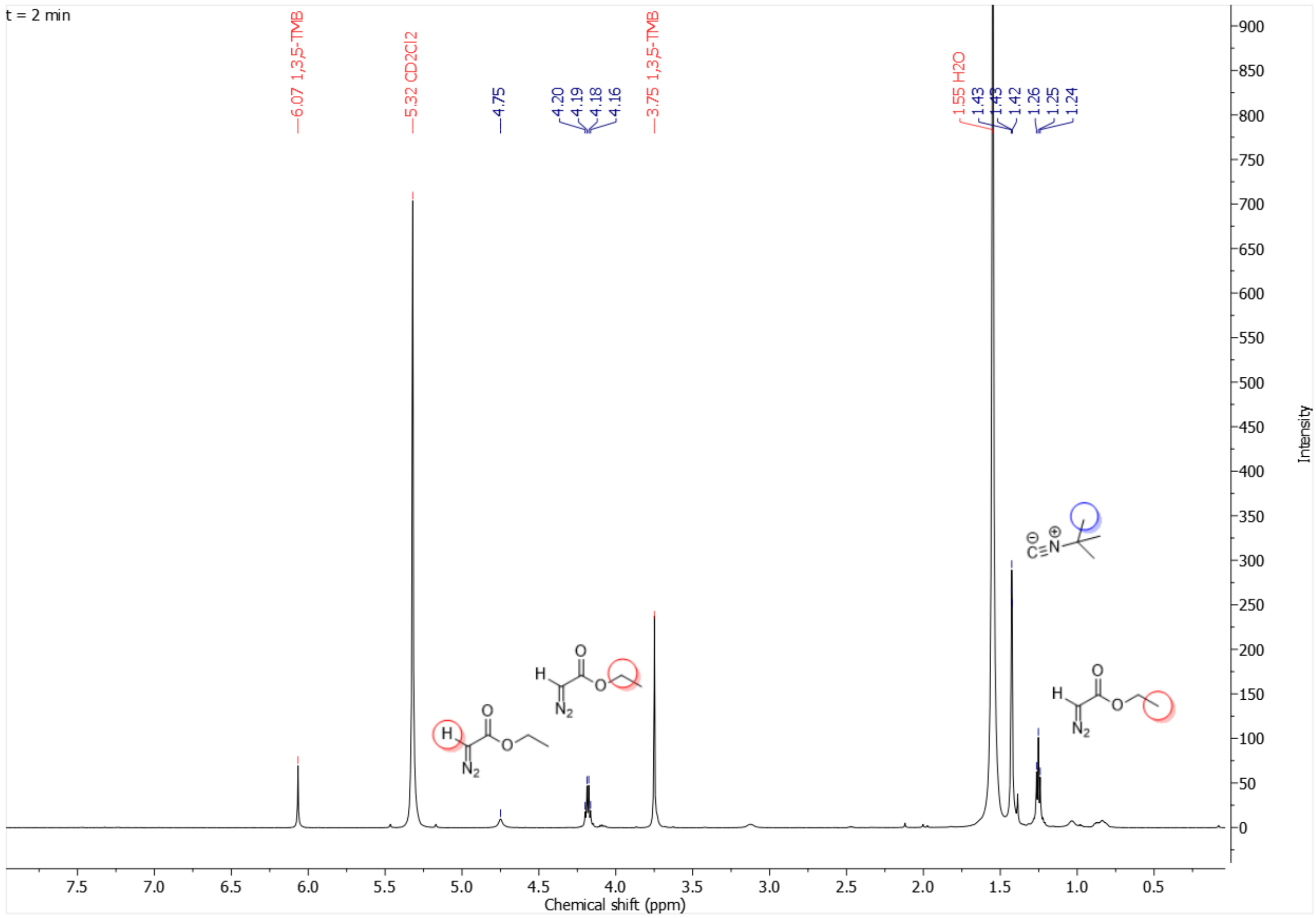

Scheme S2: ^{1}H-NMR spectrum of the reaction mixture after stirring for 2 min. 1,3,5-trimethoxybenzene present as the internal standard.

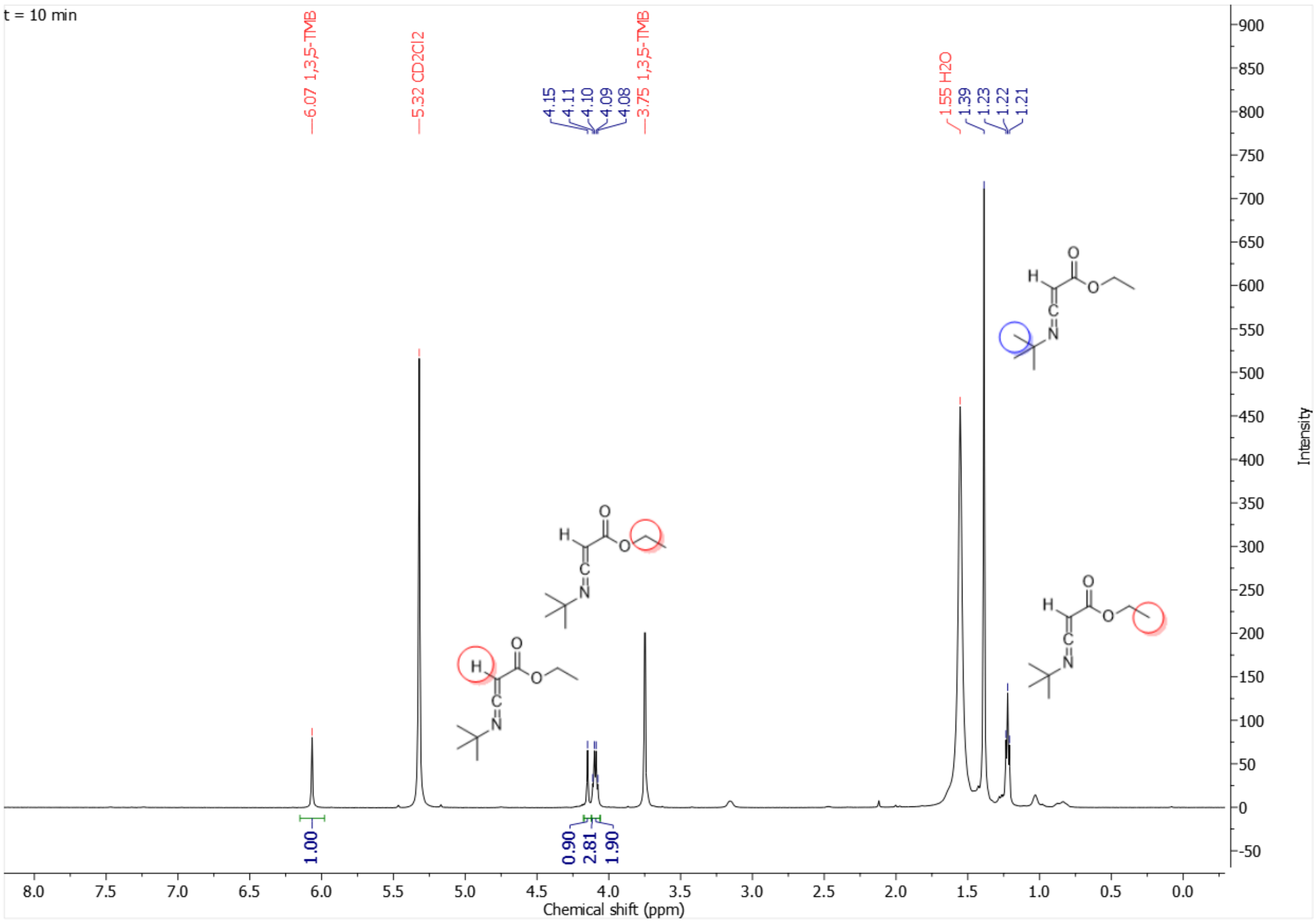

Scheme S3: ^{1}H-NMR spectrum of the reaction mixture after stirring for 10 min. 1,3,5-trimethoxybenzene present as the internal standard.

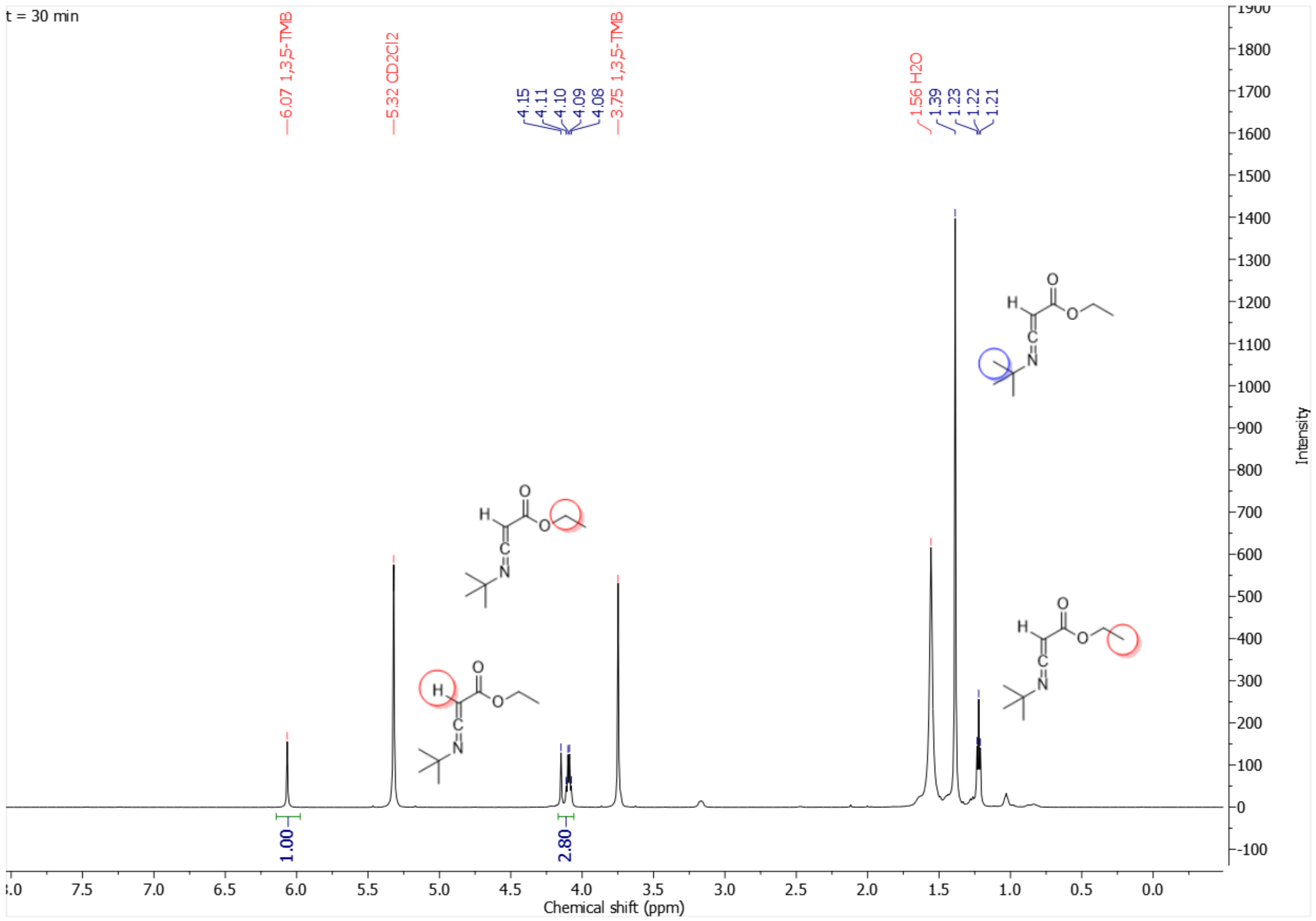

Scheme S4: ^{1}H-NMR spectrum of the reaction mixture after stirring for 30 min. 1,3,5-trimethoxybenzene present as the internal standard.

4. Synthesis of starting materials

Used diazo compounds in this work were either obtained commercially or synthesized according to the corresponding literature procedures. *It should be noted that diazo compounds could be potentially explosive. Correct safety measures and careful handling are required.*

Tetra-*N*-butylammonium Tricarbonylnitosoferrate (TBA[Fe])

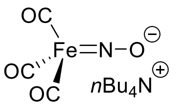

TBA[Fe] was synthesized according to well-established literature procedures[10] starting from $Fe(CO)_5$ (15.0 mmol, 1.0 equiv., 1.97 mL), $NaNO_2$ (15.0 mmol, 1.0 equiv., 1.035 g) and BrN(*n*-Bu)$_4$ (15.0 mmol, 1.0 equiv., 4.863 g) to give TBA[Fe] as a yellow solid (5.196 g, 84%). **IR (Neat):** *v*max (cm^{-1}) = 1975 (w), 1846 (s), 1636 (m). **IR (1,2-DCE, film):** *v*max (cm^{-1}) = 1988 (w), 1872 (s), 1639 (m).

diethyl 2-diazosuccinate (2b)

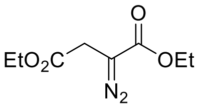

α-Diazo ester **2b** was synthesized according a procedure found in literature, starting from diethyl acetylsuccinate (1.53 mL, 7.1 mmol).[1] The title compound was isolated as a yellow oil (593 mg, 3.0 mmol, 42%). The characterization data matched the data reported in literature.[2] **^{1}H NMR** (500 MHz, $CDCl_3$): δ 4.23 (q, *J* = 7.2 Hz, 2H), 4.20 (q, *J* = 7.1 Hz, 2H), 3.31 (s, 2H), 1.30-1.26 (m, 6H) ppm.

dimethyl 2-diazosuccinate (2c)

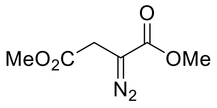

α-Diazo ester **1c** was synthesized according a procedure found in literature, starting from dimethyl acetylsuccinate (1.46 mL, 9.0 mmol).[3] The title compound was isolated as a yellow oil (1.27 g, 7.3 mmol, 83%). The characterization data matched the data reported in literature.[1] **^{1}H NMR** (500 MHz, $CDCl_3$): δ 3.77 (s, 3H), 3.74 (s, 3H), 3.32 (s, 2H) ppm.

diethyl 2-diazopentanedioate (2d)

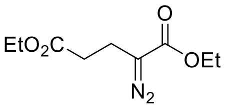

α-Diazo ester **2d** was synthesized according to a procedure found in literature, starting from diethyl 2-acetylgluterate (1.07 mL, 5.0 mmol).[1] The title compound was isolated as a yellow oil (747 mg, 3.1 mmol, 62%). The characterization data matched the data reported in literature.[5] **^{1}H NMR** (300 MHz, $CDCl_3$): δ 4.25-4.11 (m, 4H), 2.63-2.53 (m, 4H), 1.27 (t, *J* = 7.1 Hz, 3H), 1.26 (t, 7.1 Hz, 3H) ppm.

ethyl 2-diazopropanoate (2f)

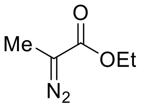

α-Diazo ester **2f** was synthesized according a procedure found in literature starting from 2-methylacetoacetate (2.36 mL, 15.0 mmol).[6] The title compound was isolated as a yellow liquid (1.50 g, 11.7 mmol, 78%). The characterization data matched the data reported in literature.[6] **^{1}H NMR** (500 MHz, $CDCl_3$): δ 4.22 (q, *J* = 7.1 Hz, 2H), 1.96 (s, 3H), 1.27 (t, *J* = 7.1 Hz, 3H) ppm.

3-diazodihydrofuran-2(3H)-one (2g)

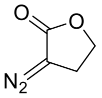

α-Diazo ester **2g** was synthesized according a procedure found in literature, starting from α-acetylbutyrolactone (1.08 mL, 40.0 mmol).[7] The title compound was isolated as a yellow oil (1.15 g, 10.2 mmol, 26%). The characterization data matched the data reported in literature.[7] **^{1}H NMR** (500 MHz, $CDCl_3$): δ 4.40 (t, *J* = 7.6 Hz, 2H), 3.37 (t, *J* = 7.6 Hz, 2H) ppm.

dimethyl 2-diazomalonate (2h)

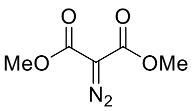

α-Diazo ester **1h** was synthesized according a procedure found in literature, starting from dimethyl malonate (3.43 mL, 30 mmol).[1] The title compound was isolated as a white solid (3.75 g, 23.7 mmol, 79%). The characterization data matched the data reported in literature.[3] **^{1}H NMR** (300 MHz, $CDCl_3$) δ 3.86 (s, 6H) ppm.

ethyl 2-diazo-2-phenylacetate (2i)

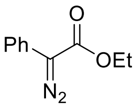

α-Diazo ester **2i** was synthesized according to a procedure found in literature, starting from iodobenzene (111.5 µL, 1.0 mmol).[8] The title compound was isolated as a yellow solid (103 mg, 0.54 mmol, 54%). The characterization data matched the data reported in literature.[8] **^{1}H NMR** (500 MHz, $CDCl_3$): δ 7.49 (t, *J* = 8.6, 2H), 7.38 (dd, *J* = 8.4, 7.5 Hz, 2H), 7.18 (t, *J* = 7.4 Hz, 1H), 4.34 (q, *J* = 7.1 Hz, 2H), 1.34 (t, *J* = 7.1 Hz, 3H) ppm.

1-diazopropan-2-one (2j)

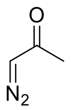

Diazoacetone **2j** was synthesized according to a procedure found in literature starting from acetylacetone (3.1 mL, 30 mmol).[9] The title compound was isolated as a volatile yellow liquid (1.51 g, 18 mmol, 60%). The characterization data matched the data reported in literature.[9] **^{1}H NMR** (300 MHz, $CDCl_3$): δ 5.26 (s, 1H), 1.97 (s, 3H) ppm.

2-diazocyclopentan-1-one (2k)

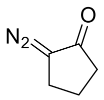

α-Diazo ester **2k** was synthesized according a procedure found in literature starting from cyclopentanone (0.18 mL, 2.0 mmol).[2] The title compound was isolated as a yellow oil (156 mg, 1.42 mmol, 71%). The characterization data matched the data reported in literature.[2] **^{1}H NMR** (300 MHz, $CDCl_3$) δ 3.04 (t, *J* = 7.4 Hz, 2H), 2.39 (t, *J* = 7.8 Hz, 1H), 2.03 (p, *J* = 7.9 Hz, 1H) ppm.

2-diazo-1-(4-methoxyphenyl)ethan-1-one (2l)

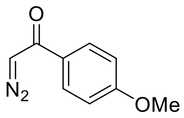

α-Diazo ester **2l** was synthesized according to a procedure found in literature starting from 4-methoxyacetophenone (300 mg, 2.0 mmol).[2] The title compound was isolated as a yellow solid (148 mg, 0.84 mmol, 42%). The characterization data matched the data reported in literature.[2] **^{1}H NMR** (300 MHz, $CDCl_3$): δ 7.74 (d, *J* = 8.7 Hz, 2H), 6.93 (d, *J* = 8.9 Hz, 2H), 5.85 (s, 1H), 3.86 (s, 3H) ppm.

2-diazoacetonitrile (2m)

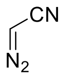

Diazonitrile was synthesized according a procedure found in literature starting from aminoacetonitrile (631 mg, 15 mmol).[3] The title compound was isolated as a yellow volatile liquid (382 mg, 5.7 mmol, 38%). The characterization data matched the data reported in literature.[3] **^{1}H NMR** (500 MHz, $CDCl_3$): δ 4.41 (s, 1H) ppm.

5. Scope data

5.1 General procedure free amidine

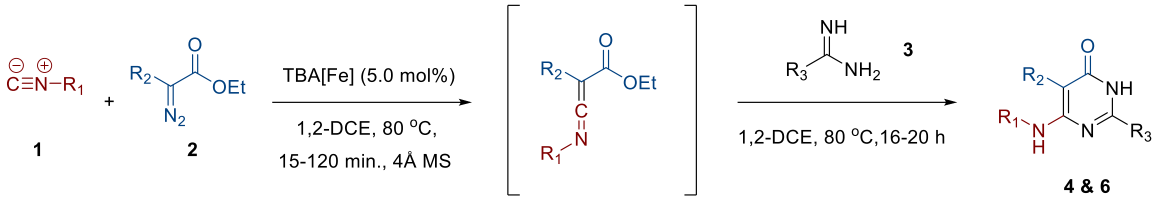

To a flame dried Schlenk flask under N_2 atmosphere, charged with 4Å MS and a stirring bar, was added TBA[Fe] (10.3 mg, 0.025 mmol, 0.05 equiv). Subsequently, 1,2-DCE (1.0 mL) was added and the mixture was stirred until the catalyst was dissolved. This was followed by the addition of α-diazo compound (**2**) (0.5 mmol, 1.0 equiv) and isocyanide (**1**) (0.5 mmol, 1.0 equiv.). The solution was placed in a pre-heated oil bath and stirred for 15-120 minutes at 80 °C. Subsequently, amidine **3** (0.6 mmol, 1.2 equiv.) was added as a solution in 1,2-DCE (1.0 mL). The reaction was allowed to stir overnight at 80 °C. Subsequently, the reaction mixture was filtered through a pad of silica using 5% MeOH in CH_2Cl_2 as eluent. The filtrate was collected and concentrated *in vacuo*. The crude product was then subjected to flash column chromatography with a mixture of MeOH and CH_2Cl_2 as eluent to obtain the title compound.

5.2 General procedure amidine HCl salt

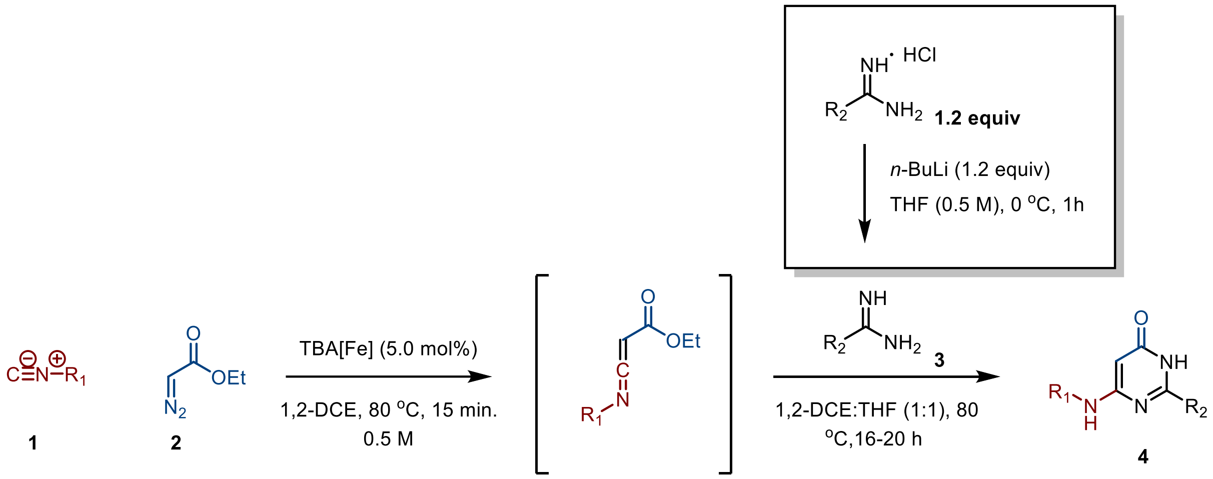

To a flame dried flask under N_2 atmosphere amdine HCl salt (0.6 mmol, 1.2 equiv) was added followed by the addition of THF (1.0 mL). Subsequently, *n*-BuLi 2,5 M in hexanes (0.24 mL, 0.6 mmol, 1,2 equiv) was added dropwise at 0 °C. The suspension was allowed to stir for 1 h at the given temperature. In the meanwhile, to a flame dried Schlenk flask under N_2 atmosphere, charged with 4Å MS and a stirring bar, was added TBA[Fe] (10.3 mg, 0.025 mmol, 0.05 equiv). Subsequently, 1,2-DCE (1.0 mL) was added and the mixture was stirred until the TBA[Fe] was dissolved. This was followed by the addition of ethyl diazoacetate (57.8 µL, 0.5 mmol, 1.0 equiv) and *t*-butyl isocyanide (56.6 µL, 0.5 mmol, 1.0 equiv.). The solution was placed in a pre-heated oil bath and stirred for 15-30 minutes at 80 °C. Subsequently, the solution of the deprotonated amidine in THF was added. The reaction was allowed to stir overnight at 80 °C, after which the mixture was filtered through a pad of silica using 5% MeOH in CH_2Cl_2 as eluent. The filtrate was collected and concentrated *in vacuo*. The crude product was then subjected to flash column chromatography with a mixture of MeOH and CH_2Cl_2 as eluent to obtain the title compound.

6-(*tert*-butylamino)-2-phenylpyrimidin-4(3H)-one (4aa)

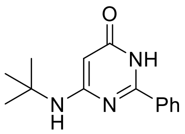

6-aminopyrimid-4(3*H*)-one **4aa** was synthesized according to general procedure **4.1.** The crude product was purified by flash chromatography using MeOH:CH_2Cl_2 = (2: 98) as eluent to obtain the product as an amorphous red-orange solid (107 mg, 0.44 mmol, 88%). **R_f** = 0.21 (MeOH:CH_2Cl_2 = 2:98); **^{1}H NMR** (500 MHz, $CDCl_3$): δ 12.93 (s, 1H), 8.20 – 8.09 (m, 2H), 7.54 – 7.41 (m, 3H), 5.42 (s, 1H), 5.07 (s, 1H), 1.43 (s, 9H) ppm; **^{13}C NMR** (150.9 MHz, $CDCl_3$): 165.96 (C_q), 161.96 (C_q), 156.42 (C_q), 132.58 (C_q), 131.69 (CH), 128.90 (CH), 127.78 (CH), 85.74 (CH), 51.00 (C_q), 29.21 (CH_3) δ ppm; **IR**

(Neat): *v*max (cm^{-1}) = 2924 (w), 1690 (m), 1594 (s), 1466 (m), 1412 (w), 1288 (m), 980 (w), 810 (m), 648 (w), 525 (m); **HRMS (ESI):** m/z calculated for $C_{14}H_{18}N_3O$ [M+H$^+$] = 244.1444 , found = 244.1456.

6-(*tert*-butylamino)-2-phenylpyrimidin-4(3H)-one (4aa)

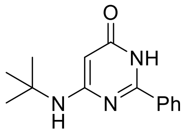

6-aminopyrimid-4(3*H*)-one **4aa** was synthesized according to general procedure **4.2.** The crude product was purified by flash chromatography using MeOH:CH_2Cl_2 = (2: 98) as eluent to obtain the product as an amorphous red-orange solid (103 mg, 0.43 mmol, 85%). **R$_f$** = 0.21 (MeOH:CH_2Cl_2 = 2:98); **^{1}H NMR** (500 MHz, $CDCl_3$): δ 12.93 (s, 1H), 8.20 – 8.09 (m, 2H), 7.54 – 7.41 (m, 3H), 5.42 (s, 1H), 5.07 (s, 1H), 1.43 (s, 9H) ppm; **^{13}C NMR** (150.9 MHz, $CDCl_3$): 165.96 (C_q), 161.96 (C_q), 156.42 (C_q), 132.58 (C_q), 131.69 (CH), 128.90 (CH), 127.78 (CH), 85.74 (CH), 51.00 (C_q), 29.21 (CH_3) δ ppm; **IR (Neat):** *v*max (cm^{-1}) = 2924 (w), 1690 (m), 1594 (s), 1466 (m), 1412 (w), 1288 (m), 980 (w), 810 (m), 648 (w), 525 (m); **HRMS (ESI):** m/z calculated for $C_{14}H_{18}N_3O$ [M+H$^+$] = 244.1444 , found = 244.1456.

2-phenyl-6-((2,4,4-trimethylpentan-2-yl)amino)pyrimidin-4(3H)-one (4ba)

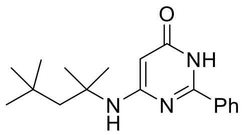

6-aminopyrimid-4(3*H*)-one **4ba** was synthesized according to general procedure 4.1. The crude product was purified by flash chromatography using MeOH:CH_2Cl_2 = (3: 97) as eluent to obtain the product as a yellow amorphous solid (128 mg, 0.43 mmol, 86%). **R$_f$** = 0.22 (MeOH:CH_2Cl_2 =3:97); **^{1}H NMR** (500 MHz, DMSO-*d*6): δ 11.68 (s, 1H), 8.13 (d, *J* = 5.7 Hz, 1H), 7.54-7.48 (m, 3H), 6.62 (s, 1H), 5.21 (s, 1H), 1.90 (s, 2H), 1.43 (s, 6H), 0.93 (s, 9H) ppm; **^{13}C NMR** (150.9 MHz, DMSO-*d*6): δ 167.90 (C_q), 164.08 (C_q), 156.53(C_q), 133.78(C_q), 131.15 (CH), 128.55 (CH), 127.39 (CH), 84.90 (CH), 54.44 (C_q), 50.44 (C_q), 31.38 (CH_2), 31.29 (CH_3), 30.57 (CH_3) ppm; **IR (Neat):** *v*max (cm^{-1}) = 2932 (w), 1629 (s), 1605 (s), 1466 (m), 1366 (m), 1281 (m), 1227 (s), 1103 (w), 802 (m), 555 (s) ; **HRMS (ESI):** m/z calculated for $C_{18}H_{26}N_3O$ [M+H$^+$] = 300.2070 , found = 300.2057.

6-(isopropylamino)-2-phenylpyrimidin-4(3H)-one (4ca)

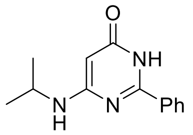

6-aminopyrimid-4(3*H*)-one **4ca** was synthesized according to general procedure 4.1. The crude product was purified by flash chromatography using MeOH:CH_2Cl_2 = (2: 98) as eluent to obtain the product as an light brown solid (81 mg, 0.35 mmol, 71%). **R$_f$** = 0.20 (MeOH:CH_2Cl_2 = 2:98); **^{1}H NMR** (500 MHz, DMSO-*d*6, 80 °C): δ 11.36 (s, 1H), 8.12 (d, *J* = 7.1 Hz, 1H), 7.71 – 6.99 (m, 3H), 6.52 (d, *J* = 7.9 Hz, 1H), 5.10 (s, 1H), 3.88 (h, *J* = 6.9 Hz, 1H), 1.19 (d, *J* = 6.4 Hz, 5H). ppm; **^{13}C NMR** (150.9 MHz, DMSO-*d*6, 80 °C): δ 163.82 (C_q), 161.89 (C_q), 156.70 (C_q), 133.23 (C_q), 130.62 (CH), 127.92 (CH), 127.13 (CH), 82.31 (CH), 41.97 (CH), 21.94 (CH_3) ppm; **IR (Neat):** *v*max (cm^{-1}) = 2970 (w), 1627 (s), 1551 (m), 1466 (w), 1288 (m), 1180 (w), 995 (s), 926 (w), 818 (m), 596 (m); **HRMS (ESI):** m/z calculated for $C_{13}H_{16}N_3O$ [M+H$^+$] = 230.1288 , found =230.1283.

6-(cyclohexylamino)-2-phenylpyrimidin-4(3H)-one (4da)

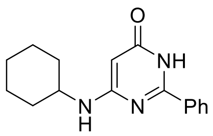

6-aminopyrimid-4(3*H*)-one **4da** was synthesized according to general procedure 4.1. The crude product was purified by flash chromatography using MeOH:CH_2Cl_2 = (2: 98) as eluent to obtain the product as an orange amorphous solid (118 mg, 0.44 mmol, 87%). **R$_f$** = 0.15 (MeOH:CH_2Cl_2 = 2:98); **^{1}H NMR** (500 MHz, $CDCl_3$): δ 12.96 (s, 1H), 8.17 – 8.00 (m, 1H), 7.50-7.45 (m, 3H), 5.27 (s, 1H), 5.11 (d, *J* = 7.0 Hz, 1H), 3.30 (s, 1H), 2.03 (dd, *J* = 13.1, 3.8 Hz, 2H), 1.83 – 1.69 (m, 2H), 1.63 (dt, *J* = 13.0, 3.9 Hz, 1H), 1.46 – 1.28 (m, 2H), 1.28-1.15 (m, 3H) ppm; **^{13}C NMR** (150.9 MHz, $CDCl_3$): δ 166.28 (C_q), 162.20 (C_q), 156.91 (C_q), 132.49 (C_q), 131.70 (CH), 128.85 (CH), 127.83 (CH), 83.21 (CH), 50.74 (CH), 32.68 (CH_2), 25.66 (CH_2), 24.81 (CH_2) ppm; **IR (Neat):** *v*max (cm^{-1}) = 2924 (w), 2854 (w), 1620 (s), 1551 (s), 1435 (m), 1288 (m), 980 (m), 810 (w), 687 (m), 532 (w); **HRMS (ESI):** m/z calculated for $C_{16}H_{20}N_3O$ [M+H$^+$] = 270.1601 , found = 270.1599.

6-(pentylamino)-2-phenylpyrimidin-4(3H)-one (4ea)

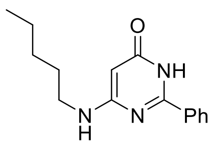

6-aminopyrimid-4(3*H*)-one **4ea** was synthesized according to general procedure 4.1. The crude product was purified by flash chromatography using MeOH:CH_2Cl_2 = (2: 98) as eluent to obtain the product as an off brown solid (93 mg, 0.36 mmol, 72%). **R$_f$** = 0.31 (MeOH:CH_2Cl_2 = 2:98); **^{1}H NMR** (500 MHz, DMSO-*d*6): δ 11.72 (s, 1H), 8.08 (d, *J* = 7.6 Hz, 2H), 7.71 – 7.31 (m, 3H), 7.02 (t, *J* = 5.7 Hz, 1H), 5.00 (s, 1H), 3.07 (s, 2H), 1.53 (t, *J* = 7.1 Hz, 2H), 1.34 – 1.26 (m, 4H), 0.90 – 0.81 (m, 3H) ppm; **^{13}C NMR** (150.9 MHz, DMSO-*d*6, 80 °C): δ 163.80 (C_q), 162.77 (C_q), 156.58 (C_q), 133.12 (C_q), 130.77 (CH), 128.02 (CH), 127.19 (CH), 81.91 (CH), 40.77 (CH_2), 28.37 (CH_2), 28.05 (CH_2), 21.44 (CH_2), 13.38 (CH_3) ppm; **IR (Neat):** *v*max (cm^{-1}) = 3294(w), 2932 (w), 2862 (w), 1605 (s), 1528 (s), 1281 (m), 1150 (w), 1080 (w), 972 (w), 687 (m); **HRMS (ESI):** m/z calculated for $C_{15}H_{20}N_3O$ [M+H$^+$] = 258.1601 , found = 258.1587.

6-((3-methoxypropyl)amino)-2-phenylpyrimidin-4(3H)-one (4fa)

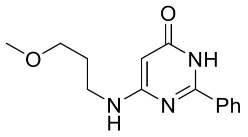

6-aminopyrimid-4(3*H*)-one **4fa** was synthesized according to general procedure 4.1. The crude product was purified by flash chromatography using MeOH:CH_2Cl_2 = (2: 98) as eluent to obtain the product as light brown solid (52 mg, 0.20 mmol, 40%). **R_f** = 0.21 (MeOH:CH_2Cl_2 = (2: 98); ^{1}H NMR (500 MHz, $CDCl_3$) δ 12.47 (s, 1H), 8.13 (dd, *J* = 7.8, 1.9 Hz, 2H), 7.92 – 7.37 (m, 3H), 5.34 (s, 1H), 5.27 (s, 1H), 3.51 (t, *J* = 5.8 Hz, 2H), 3.36 (s, 3H), 3.31 (d, *J* = 7.3 Hz, 1H), 1.91 (p, *J* = 6.2 Hz, 2H). ppm; **^{13}C NMR** (150.9 MHz, $CDCl_3$): δ 165.97 (C_q), 163.34 (C_q), 156.62 (C_q), 132.48 (C_q), 131.81 (CH), 128.94 (CH), 127.77 (CH), 83.11 (CH), 70.87 (CH_2), 58.98 (CH_3), 40.19 (CH_2), 28.96 (CH_2) ppm; **IR (Neat):** *v*max (cm^{-1}) = 3333 (w), 2931 (w), 2839 (w), 1629 (s), 1535 (s), 1373 (m), 1095 (m), 964 (m), 887 (m), 787 (s); **HRMS (ESI):** m/z calculated for $C_{14}H_{18}N_3O_2$ [M+H$^+$] = 260.1394, found = 260.1397.

6-(benzylamino)-2-phenylpyrimidin-4(3H)-one (4ga)

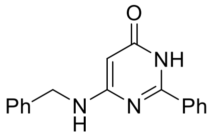

6-aminopyrimid-4(3*H*)-one **4ga** was synthesized according to general procedure 4.1. The crude product was purified by flash chromatography using MeOH:CH_2Cl_2 = (3: 97) as eluent to obtain the product as orange solid (90 mg, 0.32 mmol, 65%). **R_f** = 0.36 (MeOH:CH_2Cl_2 = (3: 97)); **^{1}H NMR** (500 MHz, DMSO-*d*6, 80 °C): δ 11.36 (s, 1H), 8.11 (d, *J* = 7.0 Hz, 1H), 7.58 – 7.42 (m, 3H), 7.38-7.35 (m, 4H), 7.29 (t, *J* = 5.96 Hz, 1H), 7.24 (t, *J* = 7.1 Hz, 1H), 5.12 (s, 1H), 4.47 (d, *J* = 6.2 Hz, 2H) ppm; **^{13}C NMR** (150.9 MHz, DMSO-*d6*, 80 °C): δ 163.78 (C_q), 162.66 (C_q), 156.83 (C_q), 139.14 (C_q), 133.15 (C_q), 130.67 (CH), 127.92 (CH), 127.86 (CH), 127.17 (CH), 126.75 (CH), 126.32 (CH), 82.83 (CH), 44.26 (CH_2) ppm; **IR (Neat):** *v*max (cm^{-1}) = 3394 (w), 3063 (w) 1627 (s), 1519 (s), 1435 (m), 1281 (s), 1142 (w), 972 (m), 872 (s), 687 (s); **HRMS (ESI):** m/z calculated for $C_{17}H_{16}N_3O$ [M+H$^+$] = 278.1288 , found = 278.1281.

***tert*-butyl (6-oxo-2-phenyl-1,6-dihydropyrimidin-4-yl)glycinate (4ha)**

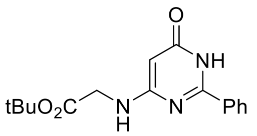

6-aminopyrimid-4(3*H*)-one **4ha** was synthesized according to general procedure 4.1. The crude product was purified by flash chromatography using MeOH:CH_2Cl_2 = (2: 98) as eluent to obtain the product as light brown solid (45 mg, 0.15 mmol, 30%). **R_f** = 0.25 (MeOH:CH_2Cl_2 = (2: 98)); **^{1}H NMR** (500 MHz, DMSO-*d*6, 80 °C): δ 11.38 (s, 1H), 8.14 (d, *J* = 7.1 Hqz, 1H), 7.53 (t, *J* = 7.2 Hz, 1H), 7.48 (t, *J* = 7.2 Hz, 2H), 7.00 (d, *J* = 6.3 Hz, 1H), 5.22 (s, 1H), 3.93 (d, *J* = 6.0 Hz, 2H), 1.42 (s, 9H). ppm; **^{13}C NMR** (150.9 MHz, DMSO-*d*6, 80 °C): δ 169.14 (C_q), 164.21 (C_q), 162.74 (C_q), 157.05 (C_q), 133.19 (C_q), 130.76 (CH), 127.91 (CH), 127.19 (CH), 83.74 (CH), 80.23 (C_q), 43.51 (CH_2), 27.45 (CH_3). ppm; **IR (Neat):** *v*max (cm^{-1}) = 3063 (w), 1736 (m), 1628 (s), 1558 (s), 1366 (w), 1149 (s), 910 (m), 802 (m), 694 (s), 563 (m); **HRMS (ESI):** m/z calculated for $C_{16}H_{19}NaO_3$ [M+Na$^+$] = 324.1319 , found = 324.1302.

6-((2,6-dimethylphenyl)amino)-2-phenylpyrimidin-4(3H)-one (4ja)

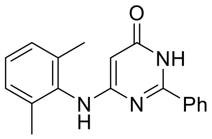

6-aminopyrimid-4(3*H*)-one **4ja** was synthesized according to general procedure 4.1. The crude product was purified by flash chromatography using MeOH:CH_2Cl_2 = (3: 97) as eluent to obtain the product as light brown solid (84 mg, 0.29 mmol, 58%). **R_f** = 0.27 (MeOH:CH_2Cl_2 = (3: 97)); **^{1}H NMR** (500 MHz, $CDCl_3$): δ 11.94 (s, 1H), 8.18 – 7.91 (m, 1H), 7.56 – 7.48 (m, 3H), 7.21 – 7.07 (m, 3H), 6.24 (s, 1H), 4.85 (s, 1H), 2.27 (s, 6H). ppm; **^{13}C NMR** (150.9 MHz, DMSO-*d*6, 80 °C): δ 163.91 (C_q), 161.91 (C_q), 157.14 (C_q), 135.74 (C_q), 135.42 (C_q), 132.94 (C_q), 130.79 (CH), 127.96 (CH), 127.73 (CH), 127.22 (CH), 126.39 (CH), 82.35 (CH), 17.36 (CH_3). ppm; **IR (Neat):** *v*max (cm^{-1}) = 3364 (w), 2924 (w), 1628 (s), 1566 (s), 1474 (m), 1312 (m), 1072 (w), 966 (m), 748 (m), 663 (m); **HRMS (ESI):** m/z calculated for $C_{18}H_{17}N_3O$ [M+H$^+$] = 292.1444, found = 292.1421.

6-((4-methoxyphenyl)amino)-2-phenylpyrimidin-4(3H)-one (4ka)

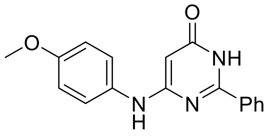

6-aminopyrimid-4(3*H*)-one **4ka** was synthesized according to general procedure 4.1. The crude product was purified by flash chromatography using MeOH:CH_2Cl_2 = (2: 98) as eluent to obtain the product as light brown solid (48 mg, 0.17 mmol, 33%). **R_f** = 0.26 (MeOH:CH_2Cl_2 = (2: 98)); **^{1}H NMR** (500 MHz, DMSO-*d6*): δ ppm; **^{13}C NMR** (150.9 MHz, DMSO-*d6*): δ 164.27 (C_q), 161.20 (C_q), 157.51 (C_q), 155.44 (C_q), 133.14 (C_q), 132.52 (C_q), 131.47 (CH), 128.58 (CH), 127.66 (CH), 123.37 (CH), 114.21 (CH), 84.52 (CH), 55.24 (CH_3). ppm; **IR (Neat):** *v*max (cm^{-1}) = 3387 (w), 2924 (w), 1612 (s), 1504 (s), 1435 (m), 1296 (m), 1165 (m), 972 (m), 625 (m), 540 (m); **HRMS (ESI):** m/z calculated for $C_{17}H_{16}N_3O_2$ [M+H$^+$] = 294.1237 , found = 294.1243.

6-(*tert*-butylamino)-2-(4-methoxyphenyl)pyrimidin-4(3H)-one (4ab)

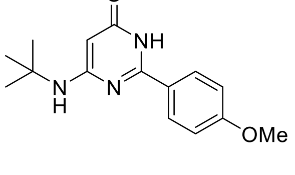

6-aminopyrimid-4(3*H*)-one **4ab** was synthesized according to general procedure 4.2. The crude product was purified by flash chromatography using MeOH:CH_2Cl_2 = (2.5: 97.5) as eluent to obtain the product as pink/red solid (116 mg, 0.42 mmol, 85%). **R_f** = 0.20 (MeOH:CH_2Cl_2 = (2.5: 97.5)); **^{1}H NMR** (500 MHz, $CDCl_3$): δ 12.68 (s, 1H), 8.12 (d, *J* = 8.9 Hz, 1H), 7.00 (d, *J* = 8.9 Hz, 1H), 5.38 (s, 1H), 5.04 (s, 1H), 3.86 (s, 3H), 1.43 (s, 9H) ppm; **^{13}C NMR** (150.9 MHz, $CDCl_3$): δ 165.94 (C_q), 162.50 (C_q), 162.06 (C_q), 156.02

(C_q), 129.51 (CH), 124.92 (C_q), 114.28 (CH), 85.22 (CH), 55.54 (CH_3), 50.98 (C_q), 29.28 (CH_3). ppm; **IR (Neat):** *v*max (cm^{-1}) = 3263 (w), 1605 (s), 1512 (m), 1412 (m) 1250 (w), 941 (w), 879 (w), 802 (m), 594 (w), 517 (m); **HRMS (ESI):** m/z calculated for $C_{15}H_{20}N_3O_2$ [M+H$^+$] = 274.1550 , found = 274.1568.

6-(*tert*-butylamino)-2-(4-(trifluoromethyl)phenyl)pyrimidin-4(3H)-one (4ac)

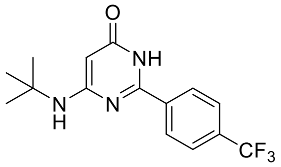

6-aminopyrimid-4(3*H*)-one **4ac** was synthesized according to general procedure 4.2. The crude product was purified by flash chromatography using MeOH:CH_2Cl_2 = (2.5: 97.5) as eluent to obtain the product as orange solid (125 mg, 0.40 mmol, 71%). **R_f** = 0.20 (MeOH:CH_2Cl_2 = (2.5: 97.5)); **^{1}H NMR** (500 MHz, $CDCl_3$) δ ppm; **^{13}C NMR** (150.9 MHz, $CDCl_3$): δ 166.05 (C_q), 161.91 (C_q), 155.16 (C_q), 135.93 (C_q), 133.22 (C_q, q, *J* = 32.6 Hz), 128.39 (CH), 125.89 (CF_3, q, *J* = 271.5 Hz), 120.70 (CH), 86.19 (CH), 51.20 (C_q), 29.22 (CH_3) ppm; **^{19}F {^{1}H} NMR** (471 MHz, $CDCl_3$): δ -62.98 ppm; **IR (Neat):** *v*max (cm^{-1}) = 3155 (w), 2870 (w), 1612 (s), 1497 (m), 1404 (m), 1296 (s), 1134 (s), 980 (w), 810 (w), 540 (w); **HRMS (ESI):** m/z calculated for $C_{15}H_{17}F_3N_3O$ [M+H$^+$] = 312.1318, found = 3w12.1344.

6-(*tert*-butylamino)-2-(4-chlorophenyl)pyrimidin-4(3H)-one (4ad)

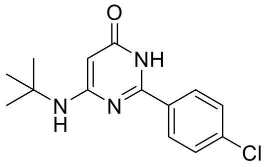

6-aminopyrimid-4(3*H*)-one **4ad** was synthesized according to general procedure 4.2. The crude product was purified by flash chromatography using MeOH:CH_2Cl_2 = (3: 97) as eluent to obtain the product as an off white solid (72 mg, 0.26 mmol, 52%). **R_f** = 0.25 (MeOH:CH_2Cl_2 = (3: 97)); **^{1}H NMR** (500 MHz, DMSO-*d*6): δ 11.50 (s, 1H), 8.14 (d, *J* = 8.6 Hz, 2H), 7.57 (d, *J* = 8.6 Hz, 2H), 6.74 (s, 1H), 5.29 (s, 1H), 1.40 (s, 9H). ppm; **^{13}C NMR** (150.9 MHz, DMSO-*d*6): δ 164.82 (C_q), 163.16 (C_q), 156.75 (C_q), 135.84 (C_q), 133.45 (C_q), 129.26 (CH), 128.62 (CH), 85.04 (CH), 50.73 (C_q), 29.35 (CH_3). ppm; **IR (Neat):** *v*max (cm^{-1}) = 2870 (w), 1636 (s), 1566 (s), 1512 (s), 1396 (w), 1281 (m), 1088 (w), 949 (w), 802 (m), 756 (w); **HRMS (ESI):** m/z calculated for $C_{14}ClH_{20}N_3O$ [M+H$^+$] = 278.1055, found = 278.1063.

6-(*tert*-butylamino)-2-methylpyrimidin-4(3H)-one (4ae)

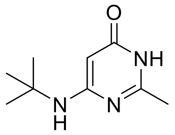

6-aminopyrimid-4(3*H*)-one **4ae** was synthesized according to general procedure 4.2. The crude product was purified by flash chromatography using MeOH:CH_2Cl_2 = (3: 97) as eluent to obtain the product as white solid (55 mg, 0.30 mmol, 61%). **R_f** = 0.20 (MeOH:CH_2Cl_2 = (3: 97)); **^{1}H NMR** (500 MHz, $CDCl_3$): δ 13.22 (s, 1H), 5.29 (s, 1H), 4.95 (s, 1H), 2.32 (s, 3H), 1.37 (s, 9H). ppm; **^{13}C NMR** (150.9 MHz, $CDCl_3$): δ 166.30 (C_q), 162.00 (C_q), 158.32 (C_q), 84.48 (CH), 50.84 (C_q), 28.99 (CH_3), 21.47 (CH_3) ppm; **IR (Neat):** *v*max (cm^{-1}) = 3263 (w), 2854 (w), 1597 (s), 1411 (m), 1288 (s), 1196 (m), 918 (w), 818 (m), 710 (w), 602 (w); **HRMS (ESI):** m/z calculated for $C_9H_{16}N_3O$ [M+H$^+$] = 182.1288, found = 182.1296.

2-(*tert*-butyl)-6-(*tert*-butylamino)pyrimidin-4(3H)-one (4af)

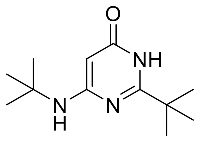

6-aminopyrimid-4(3*H*)-one **4af** was synthesized according to general procedure 4.2. The crude product was purified by flash chromatography using MeOH:CH_2Cl_2 = (2.5: 97.5) as eluent to obtain the product as white solid (88 mg, 0.39 mmol, 78%). **R_f** = 0.28 (MeOH:CH_2Cl_2 = (3: 97)); **^{1}H NMR** (500 MHz, $CDCl_3$): δ 11.17 (s, 1H), 5.23 (s, 1H), 4.81 (s, 1H), 1.39 (s, 9H), 1.32 (s, 9H) ppm; **^{13}C NMR** (150.9 MHz, $CDCl_3$): δ 166.99 (C_q), 165.25 (C_q), 161.79 (C_q), 84.74 (CH), 50.93 (C_q), 37.32 (C_q), 29.19 (CH_3), 28.28 (CH_3) ppm; **IR (Neat):** *v*max (cm^{-1}) = 3279 (w), 2962 (w), 1605 (s), 1574 (s), 1481 (m), 1288 (m), 1180 (s), 941 (w), 810 (w), 540 (m); **HRMS (ESI):** m/z calculated for $C_{12}H_{22}N_3O$ [M+H$^+$] = 224.1757, found = 224.1765.

6-(*tert*-butylamino)-2-(phenoxymethyl)pyrimidin-4(3H)-one (4ag)

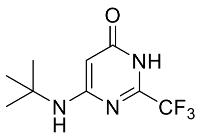

6-aminopyrimid-4(3*H*)-one **4ag** was synthesized according to general procedure 4.1. The crude product was purified by flash chromatography using MeOH:CH_2Cl_2 = (3: 97) as eluent to obtain the product as light orange solid (57 mg, 0.24 mmol, 48%). **R_f** = 0.45 (MeOH:CH_2Cl_2 = (3: 97)); **^{1}H NMR** (500 MHz, DMSO-*d*6): δ 11.50 (s, 1H), 7.28 (s, 1H), 5.77 (s, 1H), 1.36 (s, 9H) ppm; **^{13}C NMR** (150.9 MHz, DMSO-*d*6): δ 168.95 (C_q), 164.43 (C_q), 154.10 (C_q, q, *J* = 34.4 Hz), 119.61 (CF_3, q, *J* = 274.8 Hz), 87.52 (C_q), 51.08 (C_q), 28.77 (CH_3) ppm; **^{19}F NMR** (471 MHz, DMSO-*d*6): δ -65.51 ppm; **IR (Neat):** *v*max (cm^{-1}) = 3279 (w), 2885 (w), 1636 (s), 1489 (s), 1404 (w), 1358 (m), 1312 (m), 1150 (s), 980 (m), 687 (m); **HRMS (ESI):** m/z calculated for $C_9H_{13}F_3N_3O$ [M+H$^+$] = 236.1005, found = 236.1011.

6-(*tert*-butylamino)-2-(phenoxymethyl)pyrimidin-4(3H)-one (4ah)

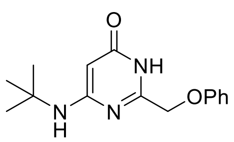

6-aminopyrimid-4(3*H*)-one **4ah** was synthesized according to general procedure 4.2. The crude product was purified by flash chromatography using MeOH:CH_2Cl_2 = (3: 97) as eluent to obtain the product as white solid (64 mg, 0.23 mmol, 47 %). **R_f** = 0.30 (MeOH:CH_2Cl_2 = (3: 97)); **^{1}H NMR** (500 MHz, DMSO-*d*6) δ 11.58 (s, 1H), 7.29 (dd, *J* = 8.7, 7.2 Hz, 2H), 7.05 – 6.88 (m, 3H), 6.61 (s, 1H), 5.03 (s, 1H), 4.83 (s, 2H), 1.26 (s, 9H) ppm; **^{13}C NMR** (150.9 MHz, DMSO-*d*6): δ 162.52 (C_q), 162.33 (C_q), 157.82 (C_q), 156.51 (C_q), 129.47 (CH), 121.14 (CH), 114.65 (CH), 84.91 (CH),

66.75 (CH_2), 50.53 (C_q), 29.13 (CH_3) ppm; **IR (Neat):** *v*max (cm^{-1}) = 3256 (w), 2962 (w), 2839 (w), 1589 (s), 1481 (s), 1412 (m), 1234 (m), 1072 (w), 957 (w), 687 (m); **HRMS (ESI):** m/z calculated for $C_{15}H_{20}N_3O_2$ [$M+H^+$] = 274.1550, found = 274.1567.

6-(*tert*-butylamino)-2-cyclopropylpyrimidin-4(3H)-one (4ai)

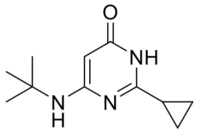

6-aminopyrimid-4(3*H*)-one **4ai** was synthesized according to general procedure 4.2. The crude product was purified by flash chromatography using MeOH:CH_2Cl_2 = (3: 97) as eluent to obtain the product as white solid (73 mg, 0.35 mmol, 70%). **R_f** = 0.25 (MeOH:CH_2Cl_2 = (3: 97)); **^{1}H NMR** (500 MHz, DMSO-*d*6) δ 11.67 (s, 1H), 6.32 (s, 1H), 4.88 (s, 1H), 1.79 (p, *J* = 6.3 Hz, 1H), 1.28 (s, 9H), 0.93 (d, *J* = 6.3 Hz, 4H).ppm; **^{13}C NMR** (150.9 MHz, DMSO-*d*6): δ 163.10 (C_q), 162.66 (C_q), 162.51 (C_q), 83.58 (CH), 50.50 (C_q), 29.53 (CH_3), 13.01 (CH) , 8.98 (CH_2) ppm; **IR (Neat):** *v*max (cm^{-1}) = 3271 (w), 2962 (w), 1597 (s), 1481 (m), 1404 (m), 1250 (m), 1188 (w), 872 (w), 810 (w), 702 (w); **HRMS (ESI):** m/z calculated for $C_{11}H_{18}N_3O$ [$M+H^+$] = 208.1444 , found = 208.1453.

ethyl 2-(4-(*tert*-butylamino)-6-oxo-2-phenyl-1,6-dihydropyrimidin-5-yl)acetate (6ab)

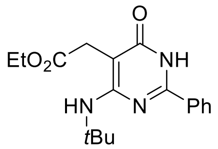

6-aminopyrimid-4(3*H*)-one **6ab** was synthesized according to general procedure 4.1 while mixing isocyanide **1a** and diazo compound **2b** at 80 °C for 2h instead of 15-30 minutes. The crude product was purified by flash chromatography using MeOH:CH_2Cl_2 = (2: 98) as eluent to obtain the product as white solid (128 mg, 0.39 mmol, 74%). **R_f** = 0.26 (MeOH:CH_2Cl_2 = (2: 98)); **^{1}H NMR** (500 MHz, $CDCl_3$) δ ppm; **^{13}C NMR** (150.9 MHz, $CDCl_3$): δ 172.43 (C_q), 164.27 (C_q), 161.42 (C_q), 154.12 (C_q), 132.80 (C_q), 131.48 (CH), 128.77 (CH), 128.03 (CH), 91.07 (C_q), 61.19 (CH_2), 52.55 (C_q), 30.18 (CH_3), 29.94 (CH_2), 14.39 (CH_3). ppm; **IR (Neat):** *v*max (cm^{-1}) = 3394 (w), 2962 (w), 1627 (s), 1713 (m), 1481 (w), 1420 (m), 1250 (w), 926 (m), 694 (w), 555 (w); **HRMS (ESI):** m/z calculated for $C_{18}H_{24}N_3O_3$ [$M+H^+$] = 330.1812, found = 330.1808.

methyl 2-(4-(*tert*-butylamino)-6-oxo-2-phenyl-1,6-dihydropyrimidin-5-yl)acetate (6ac)

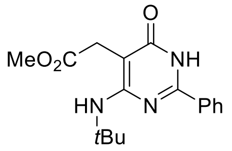

6-aminopyrimid-4(3*H*)-one **6ac** was synthesized according to general procedure 4.1 while mixing isocyanide **1** and diazo compound **2** at 80 °C for 2h instead of 15-30 minutes. The crude product was purified by flash chromatography using MeOH:CH_2Cl_2 = (2: 98) as eluent to obtain the product as white solid (60 mg, 0.35 mmol, 70%). **R_f** = 0.20 (MeOH:CH_2Cl_2 = (2: 98)); **^{1}H NMR** (500 MHz, $CDCl_3$): δ 13.04 (s, 1H), 8.76 – 7.85 (m, 2H), 7.89 – 7.24 (m, 3H), 5.50 (s, 1H), 3.68 (s, 3H), 3.59 (s, 2H), 1.55 (s, 9H).ppm; **^{13}C NMR** (150.9 MHz, $CDCl_3$): δ 172.90 (C_q), 164.14 (C_q), 161.43 (C_q), 154.13 (C_q), 132.73 (C_q), 131.57 (CH), 128.80 (CH), 127.94 (CH), 90.87 (C_q), 52.62 (C_q), 52.31 (CH_3), 30.18 (CH_3), 29.70 (CH_2). ppm; **IR (Neat):** *v*max (cm^{-1}) = 3402 (w), 2962 (w), 1720 (m), 1627 (s), 1420 (m), 1265 (m), 1142 (w), 1011 (w), 779 (w), 687 (m); **HRMS (ESI):** m/z calculated for $C_{17}H_{22}N_3O_3$ [$M+H^+$] = 316.1656, found = 316.1670.

ethyl 3-(4-(*tert*-butylamino)-6-oxo-2-phenyl-1,6-dihydropyrimidin-5-yl)propanoate (6ad)

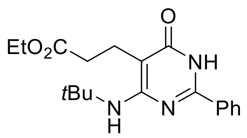

6-aminopyrimid-4(3*H*)-one **6ad** was synthesized according to general procedure 4.1 while mixing isocyanide **1** and diazo compound **2** at 80 °C for 2h instead of 15-30 minutes and 10 mol% TBA[Fe] (20.6 mg, 0.025 mmol, 0.05 equiv.). The crude product was purified by flash chromatography using MeOH:CH_2Cl_2 = (2: 98) as eluent to obtain the product as a yellow crystalline solid (59 mg, 0.17 mmol, 34%). **R_f** = 0.40 (MeOH:CH_2Cl_2 = (2: 98)); **^{1}H NMR** (500 MHz, $CDCl_3$): δ 13.23 (s, 1H), 8.32 (dd, *J* = 6.7, 3.0 Hz, 2H), 7.54 – 7.46 (m, 3H), 5.66 (s, 1H), 4.14 (d, *J* = 7.1 Hz, 1H), 2.71 (dt, *J* = 39.1, 6.5 Hz, 4H), 1.55 (s, 8H), 1.25 (t, *J* = 7.1 Hz, 3H) ppm; **^{13}C NMR** (150.9 MHz, $CDCl_3$): δ 175.30 (C_q), 164.63 (C_q), 160.89 (C_q), 153.37 (C_q), 133.06 (C_q), 131.31 (CH), 128.71 (CH), 127.82 (CH), 96.96 (C_q), 60.76 (CH_2), 52.25 (C_q), 32.65 (CH_2), 30.11 (CH_3), 19.07 (CH_2), 14.36 (CH_3) ppm; **IR (Neat):** *v*max (cm^{-1}) = 3340 (w), 2924 (w), 1720 (m), 1612 (s), 1558 (s), 1427 (m), 1312 (m), 1185 (m), 903 (w), 694 (m), 563 (s); **HRMS (ESI):** m/z calculated for $C_{19}H_{26}N_3O_3$ [$M+H^+$] = 344.1969 , found = 344.1972.

5-benzyl-6-(*tert*-butylamino)-2-phenylpyrimidin-4(3H)-one (4e)

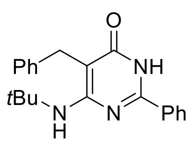

6-aminopyrimid-4(3*H*)-one **4e** was synthesized according to general procedure 4.1 while mixing isocyanide **1a** and diazo compound **2f** at 80 °C for 2h instead of 15-30 minutes and 10 mol% TBA[Fe] (20.6 mg, 0.025 mmol, 0.05 equiv.). The crude product was purified by flash chromatography using MeOH:CH_2Cl_2 = (2: 98) as eluent to obtain the product as off-white solid (60 mg, 0.18 mmol, 34%). **R_f** = 0.30 (MeOH:CH_2Cl_2 = (2: 98)); **^{1}H NMR** (500 MHz, $CDCl_3$) δ 13.35 (s, 1H), 8.34 (d, *J* = 7.0 Hz, 1H), 7.50 – 7.36 (m, 3H), 7.32 (d, *J* = 6.8 Hz, 1H), 7.28 (t, *J* = 7.8 Hz, 2H), 7.22 (t, *J* = 7.2 Hz, 2H), 4.43 (s, 1H), 3.93 (s, 2H), 1.36 (s, 9H).ppm; **^{13}C NMR** (150.9 MHz, $CDCl_3$): δ 164.88 (C_q), 160.83 (C_q), 153.81 (C_q), 139.84 (C_q), 132.92 (C_q), 131.37 (CH), 128.79 (CH), 128.75 (CH), 128.36 (CH), 127.84 (CH), 126.45 (CH), 97.24 (C_q), 52.36 (C_q), 30.01 (CH_2), 29.16 (CH_3). ppm; **IR (Neat):** *v*max (cm^{-1}) = 3433 (w), 2970 (w), 1628 (s), 1530 (s) 1481 (m), 1312 (m), 1188 (w), 1095 (w), 617 (w), 563 (m); **HRMS (ESI):** m/z calculated for $C_{21}H_{24}N_3O$ [$M+H^+$] = 334.1914, found = 334.1918.

6-(*tert*-butylamino)-5-methyl-2-phenylpyrimidin-4(3H)-one (6af)

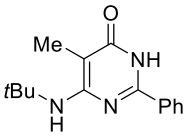

6-aminopyrimid-4(3*H*)-one **6af** was synthesized according to general procedure 4.1 while mixing isocyanide **1a** and diazo compound **2f** at 80 °C for 2h instead of 15-30 minutes and 10 mol% TBA[Fe] (20.6 mg, 0.025 mmol, 0.05 equiv.). The crude product was purified by flash chromatography using MeOH:CH_2Cl_2 = (2: 98) as eluent to obtain the product as white solid (53 mg, 0.21 mmol, 41%). **R_f** = 0.4 (MeOH:CH_2Cl_2 = (2: 98)); **^{1}H NMR** (500 MHz, $CDCl_3$): δ 12.88 (s, 1H), 8.32 – 8.28 (m, 2H), 7.54 – 7.48 (m, 3H), 4.34 (s, 1H), 1.95 (s, 3H), 1.55 (s, 9H) ppm; **^{13}C NMR** (150.9 MHz, $CDCl_3$): δ 164.29 (C_q), 160.67 (C_q), 152.92 (C_q), 133.05 (C_q), 131.29 (CH), 128.77 (CH), 127.71 (CH), 93.30 (C_q), 52.35 (C_q), 30.32 (CH_3), 8.27 (CH_3) ppm; **IR (Neat):** *v*max (cm^{-1}) = 3448 (w), 2862 (w), 1620 (s), 1558 (s), 1396 (m), 1358 (m), 1304 (m), 1211 (m), 1103 (w), 903 (w); **HRMS (ESI):** m/z calculated for $C_{15}H_{18}N_3O$ [M+H$^+$] = 258.1601 , found = 258.1615.

6-(*tert*-butylamino)-5-(2-hydroxyethyl)-2-phenylpyrimidin-4(3H)-one (6ag)

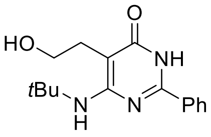

6-aminopyrimid-4(3*H*)-one **6ag** was synthesized according to general procedure 4.1 while mixing isocyanide **1a** and diazo compound **2g** at 80 °C for 2h instead of 15-30 minutes and 10 mol% TBA[Fe] (20.6 mg, 0.025 mmol, 0.05 equiv.). The crude product was purified by flash chromatography using MeOH:CH_2Cl_2 = (4: 96) as eluent to obtain the product as white solid (118 mg, 0.41 mmol, 82%). **R_f** = 0.25 (MeOH:CH_2Cl_2 = (4: 96)); **^{1}H NMR** (500 MHz, DMSO-*d*6, 80 °C): δ 11.18 (s, 1H), 8.13 (d, *J* = 5.6 Hz, 2H), 7.57-7.46 (m, 3H), 5.94 (s, 1H), 4.79 (s, 1H), 3.58 (t, *J* = 6.0 Hz, 2H), 2.58 (t, *J* = 6.0 Hz, 2H), 1.49 (s, 9H).ppm; **^{13}C NMR** (150.9 MHz, DMSO-*d*6, 80 °C): δ 163.15 (C_q), 160.90 (C_q), 153.38 (C_q), 133.45 (C_q), 131.08 (CH), 128.67 (CH), 127.19 (CH), 96.05 (C_q), 60.97 (CH_2), 51.32 (C_q), 29.85 (CH_3), 26.74 (CH_2) ppm; **IR (Neat):** *v*max (cm^{-1}) = 3356 (w), 2885 (w), 1605 (s), 1558 (s), 1474 (m), 1405 (m), 1296 (m), 1188 (w), 957 (w), 694 (m); **HRMS (ESI):** m/z calculated for $C_{16}H_{222}N_3O_2$ [M+H$^+$] = 288.1707, found = 288.1726.

***N*-(*tert*-butyl)-6-methyl-2-phenylpyrimidin-4-amine (7aj)**

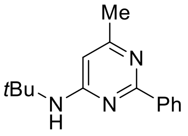

4-aminopyrimidine **7aj** was synthesized according to general procedure 4.1 while mixing isocyanide **1a** and diazo compound **2j** at 80 °C for 2h instead of 15-30 minutes. The crude product was purified by flash chromatography using MeOH:CH_2Cl_2 = (3: 97) as eluent to obtain the product as off-white solid (73 mg, 0.30 mmol, 60%). **R_f** = 0.41 (MeOH:CH_2Cl_2 = (3: 97)); **^{1}H NMR** (500 MHz, $CDCl_3$): δ 8.38 (dd, *J* = 7.8, 1.9 Hz, 2H), 7.57 – 7.34 (m, 3H), 6.08 (s, 1H), 4.78 (s, 1H), 2.39 (s, 3H), 1.51 (s, 9H) ppm; **^{13}C NMR** (150.9 MHz, $CDCl_3$): δ 164.63 (C_q), 163.62 (C_q), 162.55 (C_q), 138.89 (C_q), 129.95 (CH), 128.30 (CH), 128.16 (CH), 102.34 (C_q), 51.43 (C_q), 29.36 (CH_3), 24.30 (CH_3) ppm; **IR (Neat):** *v*max (cm^{-1}) = 3310 (w), 2916 (w), 1573 (s), 1497 (s), 1443 (m), 1358 (m), 972 (w), 694 (m), 563 (w), 517 (w); **HRMS (ESI):** m/z calculated for $C_{15}H_{20}N_3$ [M+H$^+$] = 242.1652, found = 242.1651.

***N*-(*tert*-butyl)-6-(4-methoxyphenyl)-2-phenylpyrimidin-4-amine (7al)**

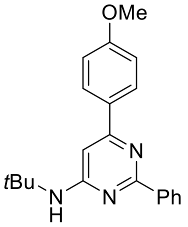

4-aminopyrimidine **7al** was was synthesized according to general procedure 4.1 while mixing isocyanide **1a** and diazo compound **2l** at 80 °C for 2h instead of 15-30 minutes. The crude product was purified by flash chromatography using MeOH:$CHCl_3$ = (2: 98) as eluent to obtain the product yellow oil (90 mg, 0.27 mmol, 54%). **R_f** = 0.88 (MeOH:$CHCl_3$ = (2: 98)); **^{1}H NMR** (500 MHz, $CDCl_3$): δ 8.55 (dd, *J* = 8.0, 1.7 Hz, 2H), 8.09 (d, *J* = 8.8 Hz, 1H), 7.60 – 7.40 (m, 3H), 7.01 (d, *J* = 8.8 Hz, 1H), 6.57 (s, 1H), 3.87 (s, 3H), 1.57 (s, 9H). ppm; **^{13}C NMR** (150.9 MHz, $CDCl_3$): δ 163.62 (C_q), 163.10 (C_q), 161.99 (C_q), 161.25 (C_q), 139.09 (C_q), 130.99 (C_q), 130.06 (CH), 128.38 (CH), 128.29 (CH), 114.06 (CH), 98.38 (CH), 55.48 (CH_3), 51.65 (C_q), 29.47 (CH_3) ppm; **IR (Neat):** *v*max (cm^{-1}) = 3417 (w), 2970 (w), 1566 (s), 1512 (s), 1443 (m), 1358 (m), 1173 (s), 818 (s), 756 (m), 694 (s) ; **HRMS (ESI):** m/z calculated for $C_{21}H_{24}N_3O$ [M+H$^+$] = 334.1914 , found =334.1922.

***N*-(*tert*-butyl)-2-phenyl-6,7-dihydro-5*H*-cyclopenta[d]pyrimidin-4-amine (7ak)**

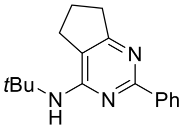

4-aminopyrimidine **7ak** was synthesized according to general procedure **4.1** while mixing isocyanide **1a** and diazo compound **2k** at 80 ˚C for 2h instead of 15-30 minutes. The crude product was purified by flash chromatography using MeOH:CH_2Cl_2 = (4: 96) as eluent to obtain the product as off-white solid (112 mg, 0.42 mmol, 84%). **R_f** = 0.50 (MeOH:CH_2Cl_2 = (4: 96)); **^{1}H NMR** (500 MHz, $CDCl_3$): δ 8.45 – 8.37 (m, 2H), 7.51 – 7.32 (m, 3H), 4.25 (s, 1H), 2.95 (t, *J* = 7.7 Hz, 2H), 2.63 (t, *J* = 7.4 Hz, 2H), 2.11 (p, *J* = 7.7 Hz, 2H), 1.58 (s, 9H). ppm; **^{13}C NMR** (150.9 MHz, $CDCl_3$): δ 170.82 (C_q), 162.81 (C_q), 158.97 (C_q), 139.30 (C_q), 129.56 (CH), 128.22 (CH), 128.07 (CH), 114.59 (C_q), 51.93 (C_q), 34.50 (CH_2), 29.41 (CH_3), 26.90 (CH_2), 21.52 (CH_2) ppm; **IR (Neat):** *v*max (cm^{-1}) = 3333 (m), 2924 (w), 1566 (s), 1489 (m), 1435 (m), 1312 (w), 1211 (w), 910 (w), 756 (m), 694 (m); **HRMS (ESI):** m/z calculated for $C_{21}H_{22}N_3$ [M+H$^+$] = 268.1808 , found = 268.1814.

4*N*-(*tert*-butyl)-2-phenylpyrimidine-4,6-diamine (7am)

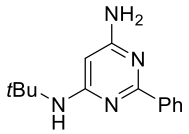

4,6-diaminopyrimidine **7am** was synthesized according to general procedure 4.1 while mixing isocyanide **1a** and diazo compound **2m** at 80 ˚C for 2h instead of 15-30 minutes. The crude product was purified by flash chromatography using MeOH:CH_2Cl_2 = (3: 97) as eluent to obtain the product as dark red/purple solid (41 mg, 0 .17 mmol, 34%). $\mathbf{R_f}$ = 0.27 (MeOH:CH_2Cl_2 = 4:96); **^{1}H NMR** (500 MHz, $CDCl_3$) δ 8.33 – 8.24 (m, 1H), 7.44-7.39 (m, 3H), 5.42 (s, 1H), 4.77 (s, 1H), 4.60 (s, 2H), 1.46 (s, 9H) ppm; **^{13}C NMR** (150.9 MHz, $CDCl_3$): δ 163.86 (C_q), 163.31 (C_q), 162.94 (C_q), 138.66 (C_q), 129.97 (CH), 128.27 (CH), 128.03 (CH), 82.91 (CH), 50.97 (C_q), 29.59 (CH_3) ppm; **IR (Neat):** *v*max (cm^{-1}) = 3479 (w), 3379 (w), 2962 (w), 1489 (s), 1443 (m), 1281 (m), 1157 (w), 972 (w), 756 (s), 694 (m); **HRMS (ESI):** m/z calculated for $C_{14}H_{19}N_4$ [$M+H^+$] = 243.1604, found = 243.1602.

5-(*tert*-butylamino)-2,4-dihydro-3*H*-pyrazol-3-one (9a)

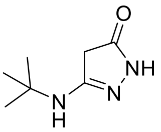

To a flame dried Schlenk flask under N_2 atmosphere, charged with 4Å MS and a stirring bar, was added TBA[Fe] (10.3 mg, 0.025 mmol, 0.05 equiv). Subsequently, 1,2-DCE (1.0 mL) was added and the mixture was stirred until the TBA[Fe] was dissolved. This was followed by the addition of ethyl diazoacetate (57.8 µL, 0.5 mmol, 1.0 equiv) and *t*-butyl isocyanide (56.6 µL, 0.5 mmol, 1.0 equiv.). The solution was placed in a pre-heated oil bath and stirred for 30 minutes at 80 °C. After that the hydrazine hydrate (45.5 µL, 0.6 mmol, 1.2 equiv.) was added as a solution in 1,2-DCE (1.0 mL). The reaction was allowed to stir overnight at 80 °C. Subsequently, the reaction mixture was filtered through a pad of silica using 5% MeOH in CH_2Cl_2 as eluent. The filtrate was collected and concentrated *in vacuo* to obtain the product as white solid (37.2 mg, 0.24 mmol, 48%). $\mathbf{R_f}$ = 0.25 (MeOH:CH_2Cl_2 = (7.5: 92.5)); **^{1}H NMR** (500 MHz, $CDCl_3$): δ 7.83 (s, 1H), 3.99 (s, 1H), 3.14 (s, 2H), 1.36 (s, 9H) ppm; **^{13}C NMR** (150.9 MHz,DMSO-*d*6): δ 170.93 (C_q), 154.80 (C_q), 50.36 (C_q), 37.05 (CH_2), 28.29 (CH_3) ppm; **IR (Neat):** *v*max (cm^{-1}) = 3325 (w), 3178 (w), 1674 (m), 1597 (s), 1558 (s), 1358 (m), 1327 (w), 756 (m), 679 (m), 540 (s); **HRMS (ESI):** m/z calculated for $C_7H_{14}N_3O$ [$M+H^+$] = 156.1131 , found = 156.1127

5-(*tert*-butylamino)-2-phenyl-2,4-dihydro-3*H*-pyrazol-3-one (9b)

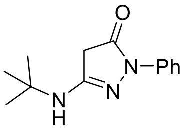

To a flame dried flask under N_2 atmosphere phenyl hydrazine·HCl (86.7 mg, 0.6 mmol, 1.2 equiv.) was added, followed by the addition of THF (1.0 mL). Subsequently, *n*-BuLi 2,5 M in hexanes (0.24 mL, 0.6 mmol, 1,2 equiv) was added dropwise at 0 °C. The suspension was allowed to stir for 1 h at the given temperature. In the meanwhile, to a flame dried Schlenk flask under N_2 atmosphere, charged with 4Å MS and a stirring bar, was added TBA[Fe] (10.3 mg, 0.025 mmol, 0.05 equiv). Subsequently, 1,2-DCE (1.0 mL) was added and the mixture was stirred until the TBA[Fe] was dissolved. This was followed by the addition of ethyl diazoacetate (57.8 µL, 0.5 mmol, 1.0 equiv) and *t*-butyl isocyanide (56.6 µL, 0.5 mmol, 1.0 equiv.). The solution was placed in a pre-heated oil bath and stirred for 30 minutes at 80 °C. Subsequently, the solution of the free phenyl hydrazine in THF was added. The reaction was allowed to stir overnight at 80 °C. Subsequently, the reaction mixture was filtered through a pad of silica using 5% MeOH in CH_2Cl_2 as eluent. The filtrate was collected and concentrated *in vacuo*. The crude product was purified by flash chromatography using EtOAc:Cyclohexane = (1:1) as eluent to obtain the product as orange solid (45.3 mg, 0.20 mmol, 39%). $\mathbf{R_f}$ = 0.32 (EtOAc:Cyclohexane = (1:1)); ^{1}H NMR (500 MHz, $CDCl_3$): δ 7.93 (d, *J* = 7.5 Hz, 1H), 7.36 (t, *J* = 7.3, 2H), 7.11 (t, *J* = 7.3 Hz, 1H), 4.17 (s, 1H), 3.42 (s, 2H), 1.44 (s, 9H). ppm; **^{13}C NMR** (150.9 MHz,DMSO-*d*6): δ 166.92 (C_q), 153.06 (C_q), 138.88 (C_q), 128.69 (CH), 124.07 (CH), 118.56 (CH), 52.23 (C_q), 40.08 (CH_2), 28.71 (CH_3). ppm; **IR (Neat):** *v*max (cm^{-1}) = 3425 (w), 3310 (w), 2970 (w), 1690 (m), 1612 (s), 1342 (s), 1026 (m), 748 (m), 697 (m), 571 (m); **HRMS (ESI):** m/z calculated for $C_{13}H_{18}N_3O$ [$M+H^+$] = 232.1444, found = 232.1437.

ethyl 2-(1-(*tert*-butyl)-1*H*-tetrazol-5-yl)acetate (10a)

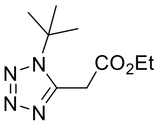

To a flame dried Schlenk flask under N_2 atmosphere, charged with 4Å MS and a stirring bar, was added TBA[Fe] (10.3 mg, 0.025 mmol, 0.05 equiv). Subsequently, 1,2-DCE (1.0 mL) was added and the mixture was stirred until the TBA[Fe] was dissolved. This was followed by the addition of ethyl diazoacetate (57.8 µL, 0.5 mmol, 1.0 equiv) and *t*-butyl isocyanide (56.6 µL, 0.5 mmol, 1.0 equiv.). The solution was placed in a pre-heated oil bath and stirred for 30 minutes at 80 °C. After that TMS-N_3 (83.8 µL, 0.6 mmol, 1.2 equiv.) was added at room temperature as a solution in 1,2-DCE (1.0 mL). The reaction was allowed to stir overnight at 80 °C. Subsequently, the reaction mixture was filtered through a pad of silica using 5% MeOH in CH_2Cl_2 as eluent. The filtrate was collected and concentrated *in vacuo*. The crude product was purified by flash chromatography using EtOAc:Cyclohexane = (30:70) as eluent to obtain the product as red oil (84.5 mg, 0.40 mmol, 80%). $\mathbf{R_f}$ = 0.38 (EtOAc:Cyclohexane = (30:70)); **^{1}H NMR** (500 MHz, $CDCl_3$): δ 4.17 (q, *J* = 7.1 Hz, 2H), 4.11 (s, 2H), 1.68 (s, 9H), 1.21 (t, *J* = 7.1 Hz, 3H). ppm; **^{13}C NMR** (150.9 MHz, $CDCl_3$): δ 167.22 (C_q), 147.90 (C_q), 62.11 (CH_2) , 61.58 (C_q), 32.14 (CH_2), 29.72 (CH_3), 14.00 (CH_3) ppm; **IR (Neat):** *v*max (cm^{-1}) = 2924 (w), 1736 (s), 1628 (s), 1512 (w), 1427 (m), 1281 (m), 1026 (m), 879 (w), 733 (w), 501 (m); **HRMS (ESI):** m/z calculated for $C_9H_{17}N_4O_2$ [$M+Na^+$] = 234.1165 , found = 234.1143.

diethyl 2-(1-(*tert*-butyl)-1*H*-tetrazol-5-yl)pentanedioate (10b)

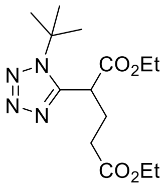

To a flame dried Schlenk flask under N_2 atmosphere, charged with 4Å MS and a stirring bar, was added TBA[Fe] (10.3 mg, 0.025 mmol, 0.05 equiv). Subsequently, 1,2-DCE (1.0 mL) was added and the mixture was stirred until the TBA[Fe] was dissolved. This was followed by the addition of ethyl diazoacetate (57.8 µL, 0.5 mmol, 1.0 equiv) and *t*-butyl isocyanide (56.6 µL, 0.5 mmol, 1.0 equiv.). The solution was placed in a pre-heated oil bath and stirred for 2 hours at 80 °C. After that TMS-N_3 (83.8 µL, 0.6 mmol, 1.2 equiv. was added at room temperature as a solution in 1,2-DCE (1.0 mL). The reaction was allowed to stir overnight at 80 °C. Subsequently, the reaction mixture was filtered through a pad of silica using 5% MeOH in CH_2Cl_2 as eluent. The filtrate was collected and concentrated *in vacuo*. The crude product was purified by flash chromatography using MeOH:CH_2Cl_2 = (2: 98) as eluent to obtain the product as a yellow transparent oil (138 mg, 0.44 mmol, 88%). **R_f** = 0.74 (MeOH:CH_2Cl_2 = (2: 98)); **^{1}H NMR** (500 MHz, $CDCl_3$): δ 4.47 (t, *J* = 7.2 Hz, 1H), 4.15 (qd, *J* = 7.1, 1.8 Hz, 2H), 4.10 (qd, *J* = 7.2, 2.0 Hz, 2H), 2.65 – 2.22 (m, 4H), 1.73 (s, 10H), 1.22 (t, *J* = 7.2 Hz, 3H), 1.19 (t, *J* = 7.1 Hz, 3H) ppm; **^{13}C NMR** (150.9 MHz, $CDCl_3$): δ 172.88 (C_q), 169.28 (C_q), 151.91 (C_q), 62.21 (CH_2), 61.63 (C_q), 60.78 (CH_2), 41.41 (CH), 30.97 (CH_2), 30.10 (CH_3), 26.48 (CH_2), 14.28 (CH_3), 14.07 (CH_3) ppm; **IR (Neat):** *v*max (cm^{-1}) = 2986 (w), 1728 (s), 1651 (w), 1489 (w), 1373 (w), 1273 (w), 1180 (m), 1026 (w), 741 (w), 501 (s); **HRMS (ESI):** m/z calculated for $C_{14}H_{17}N_3NaO$ [M+Na$^+$] = 335.1701 , found = 335.1690.

6. Attempted bis-nucleophiles

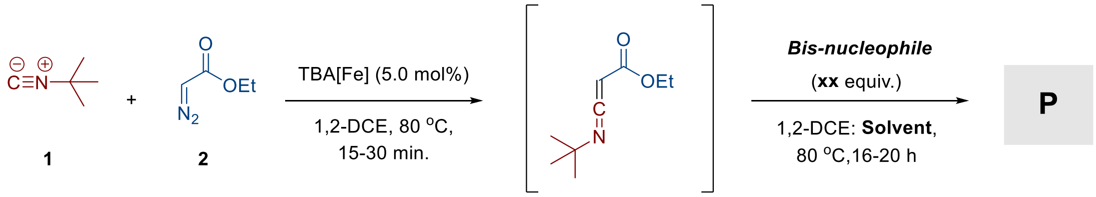

To a flame dried Schlenk flask under N_2 atmosphere, charged with a stirring bar, was added TBA[Fe] (10.3 mg, 0.025 mmol, 0.05 equiv). Subsequently, 1,2-DCE (1.0 mL) was added and the mixture was stirred until the catalyst was dissolved. This was followed by the addition of α-diazo compound **2** (0.5 mmol, 1.0 equiv) and *tert* -butyl isocyanide (**1**) (56.6 μL, 0.5 mmol, 1.0 equiv.). The solution was placed in a pre-heated oil bath and stirred for 15-30 minutes at 80 °C. Subsequently, the bis-nucleophile and additive were added to a separate Schlenk tube and stirred as a solution in the indicated solvent (1.0 mL). *(Deprotonations were performed in a salt-ice bath at -10 °C for 30 minutes).* After stirring the solution for a given time, the mixture was added to the Schlenck containing the ketenimine. The reaction was allowed to stir overnight at 80 °C. Subsequently, the reaction mixture was filtered through a pad of silica using 5%-10% MeOH in CH_2Cl_2 as eluent. The filtrate was collected and concentrated *in vacuo*.

➢ 1,4-bisnucleophiles

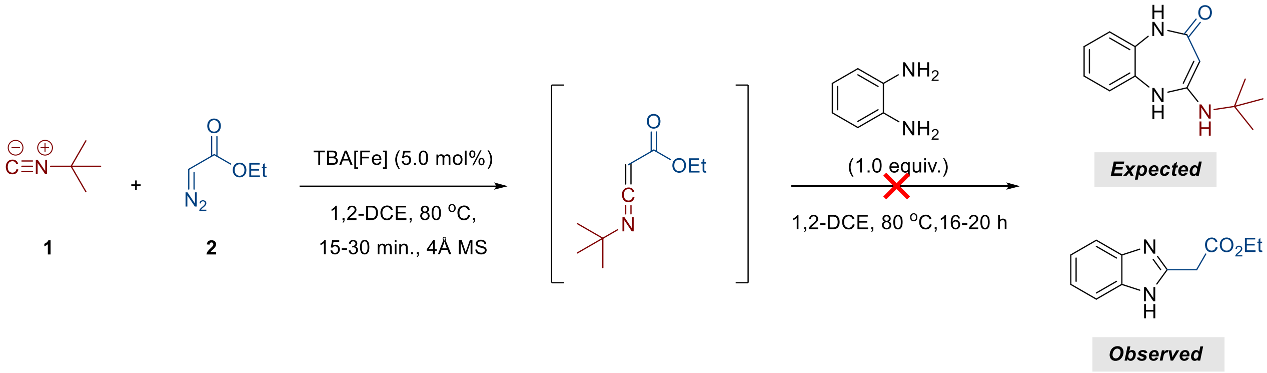

- ***Expected*** product not formed. ***Observed*** product is main product in ^{1}H-NMR of crude reaction mixture.

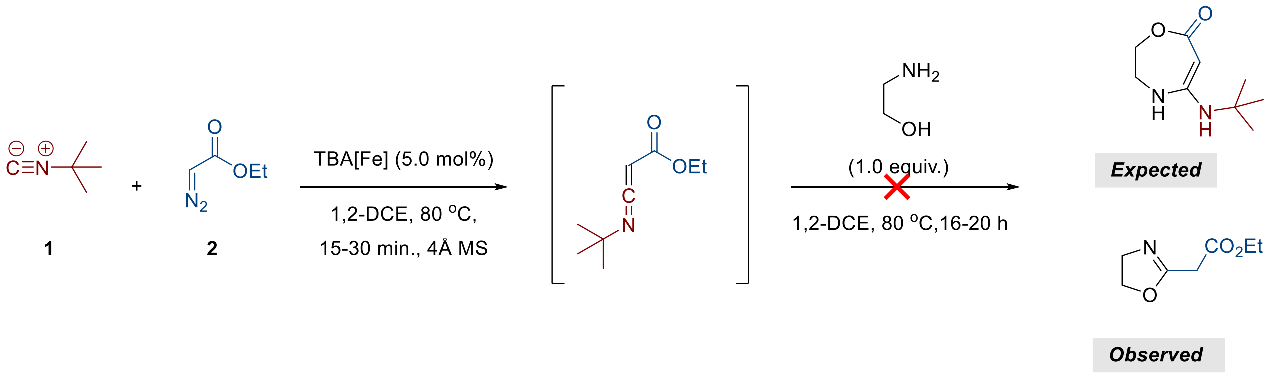

- ***Expected*** product not formed. ***Observed*** product is main product in ^{1}H-NMR of crude reaction mixture.

➢ 1,3-bisnucleophiles

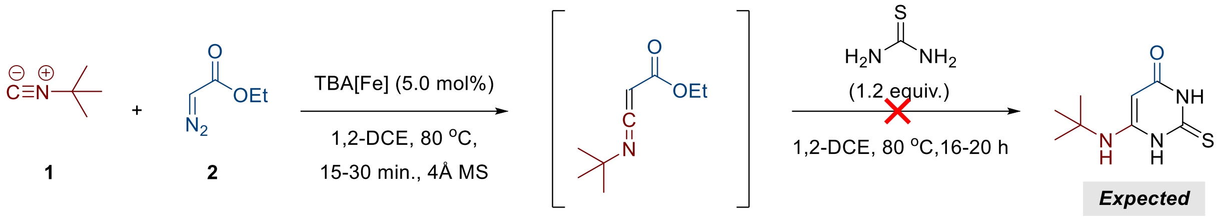

- ***Expected*** product could not be isolated. Mass of expected product not observed by SFC-MS analysis. An unknown product was isolated that still contained the characteristic signals of the ester and *t*-Bu moieties, indicating that at least no cyclisation towards the desired heterocycle occurred.

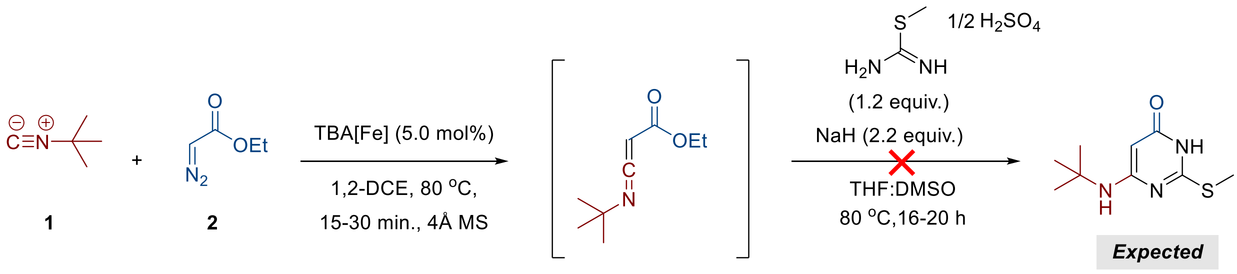

- Deprotonation of iso-thiourea at room temperature. No product could be isolated. ^{1}H-NMR of crude reaction mixture could indicate product formation, however, only in trace amounts.

➢ 1,2-bisnucleophiles

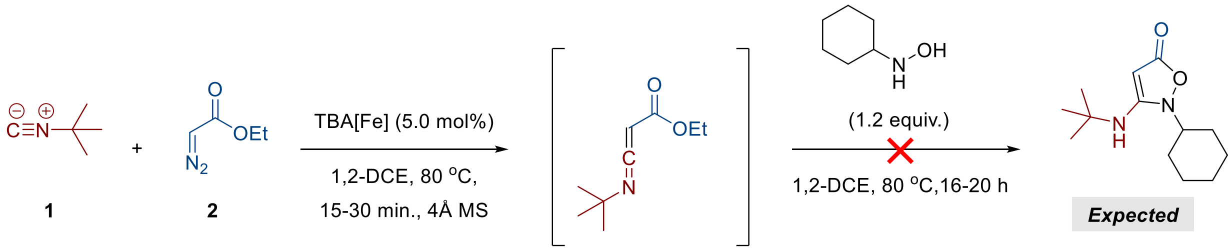

- ^{1}H-NMR of crude reaction mixture did not indicate formation of the expected product. Presumably, degradation occurred.

7. Computational studies

7.1 Computational methods

All calculations in this work were performed utilizing the Amsterdam Density Functional (ADF) software package (v2019.303).[12] Equilibrium structures and transition state geometries were optimized using the restricted BP86-functional[13] and the TZ2P basis set. Solvent effects of 1,2-dichloroethane (1,2-DCE) were implemented using the conductor-like screening model (COSMO) of solvation.[14] In addition, dispersion interactions were included using Grimme's DFT-D3 correction with Becke-Johnson (BJ) damping.[15] The zeroth-order regular approximation (ZORA) was employed to implement scalar relativistic effects.[16] Throughout this work this level is referred to as COSMO(1,2-DCE)-ZORA-BP86-D3(BJ)/TZ2P. All optimized (transition state) structures were illustrated using CYLview.[17]

All stationary points were verified to be minima (zero imaginary frequencies) or transition structures (one imaginary frequency) via vibrational analysis. For transition state structures, the character of the normal mode associated with the imaginary frequency has been visually analyzed to ensure it resembles the reaction under consideration. Intrinsic reaction coordinate (IRC) calculations were performed to unambiguously connect reactant and product complexes with transition state structures for all transition states provided.[18]

For the thermochemistry calculations of we used a standard approach. Geometries were optimized and the vibrational frequencies were obtained through numerical differentiation of the analytical gradient. Enthalpies at 298.15 K and 1 atm (ΔH°) were calculated from the electronic bond energies and vibrational frequencies by using a standard thermochemistry relation for an ideal gas [Eq. 1].

[Eq. 1] $\Delta H° = \Delta E_{trans,298} + \Delta E_{rot,298} + \Delta E_{vib,0} + \Delta(\Delta E_{vib,298}) + \Delta(pV)$

[Eq. 2] $\Delta G° = \Delta H – T\Delta S$

$\Delta E_{trans,298}$, $\Delta E_{rot,298}$, and $\Delta E_{vib,0}$ are the differences between the reactants in the translational, rotational, and zero-point vibrational energy, respectively, whereas $\Delta E_{vib,298}$ takes the vibrational energy change upon going from 0 to 298.15 K into account. The vibrational energy corrections and the entropic term TΔS° are based on frequency calculations. Thermal corrections for the electronic term are neglected and $\Delta(pV) \approx \Delta(nRT)$ was used. The change of the Gibbs free energy (ΔG) in all calculations were then calculated for 298.15 K and 1 atm (ΔG°) [Eq. 2].

7.2 Computational results

Calculations in this work were performed using the following level of theory; COSMO(1,2-DCE)-ZORA-BP86-D3(BJ)/TZ2P, unless stated otherwise. Chosen functional (BP86) was based on previous DFT calculations[19a-c] involving species; $[Fe(CO)_3NO]^-$.

7.2.1 Benchmark studies

Table S2a. Calculated and experimental values of bond lengths of $[Fe(CO)_3NO]^-$

Bond length	Calculated (Å)	Calculated [19a] (Å)	Crystal structure[20] (Å)
Fe-C	1.764	1.776	1.800(8)
Fe-N	1.648	1.661	1.659(11)
C-O	1.172	1.175	1.150(9)
N-O	1.2	1.2	1.212(14)

Table S2b. Calculated and experimental values of bond angles of $[Fe(CO)_3NO]^-$

Bond angle	Calculated	Calculated [19a]	Crystal structure[20]
C-Fe-N	116.8 °	116.1°	116.7(3)°
C-Fe-C	101.3 °	102.2°	101.4(3)°
Fe-N-O	179.8 °	180.0°	180.0°
Fe-C-O	178.7 °	178.4 °	-

Table S2c. Calculated and experimental values of vibrational frequencies of $[Fe(CO)_3NO]^-$

Method	v CO (cm^{-1})	v CO (cm^{-1})	v NO (cm^{-1})
Experimental[21],*	1979	1875	1647
BP86/def-TZVP[19a]	1953	1875	1681
COSMO(H_2O)-BP86/def-TZVP[19a]	1949	1828	1613
ZORA-OPBE-D3(BJ)/TZ2P	1995	1914	1767
ZORA-BP86-D3(BJ)/TZ2P	1950	1871	1675
COSMO(H_2O)-ZORA-BP86-D3(BJ)/TZ2P	1941	1811	1587
COSMO(1,2-DCE)-ZORA-BP86-D3(BJ)/TZ2P	1944	1819	1603

* IR spectrum recorded using the non-coordinating cationic PPN^+ and crown-ether complexed cations (Na^+ & K^+) in THF solution.

7.2.2 Isocyanide complex & Iron-carbene formation

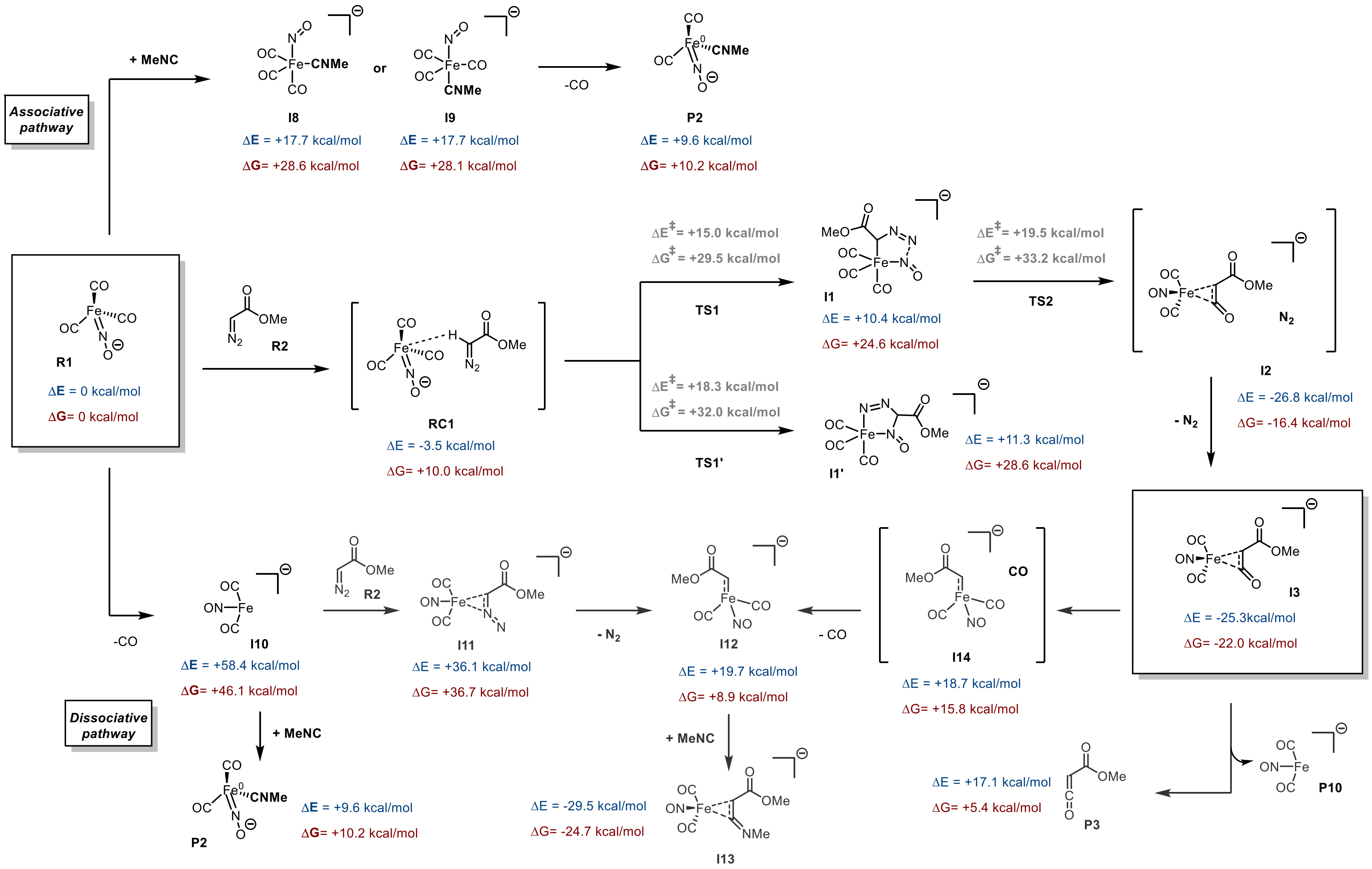

Scheme S5. Roadmap of PES for the formation of isocyanide iron complex **P2** and [Fe]-carbene **I3** formation. Relative energies in kcal mol^{-1}. Computed at COSMO(1,2-DCE)-ZORA-BP86-D3(BJ)/TZ2P.

7.2.3 Isomerization and isocyanide approach

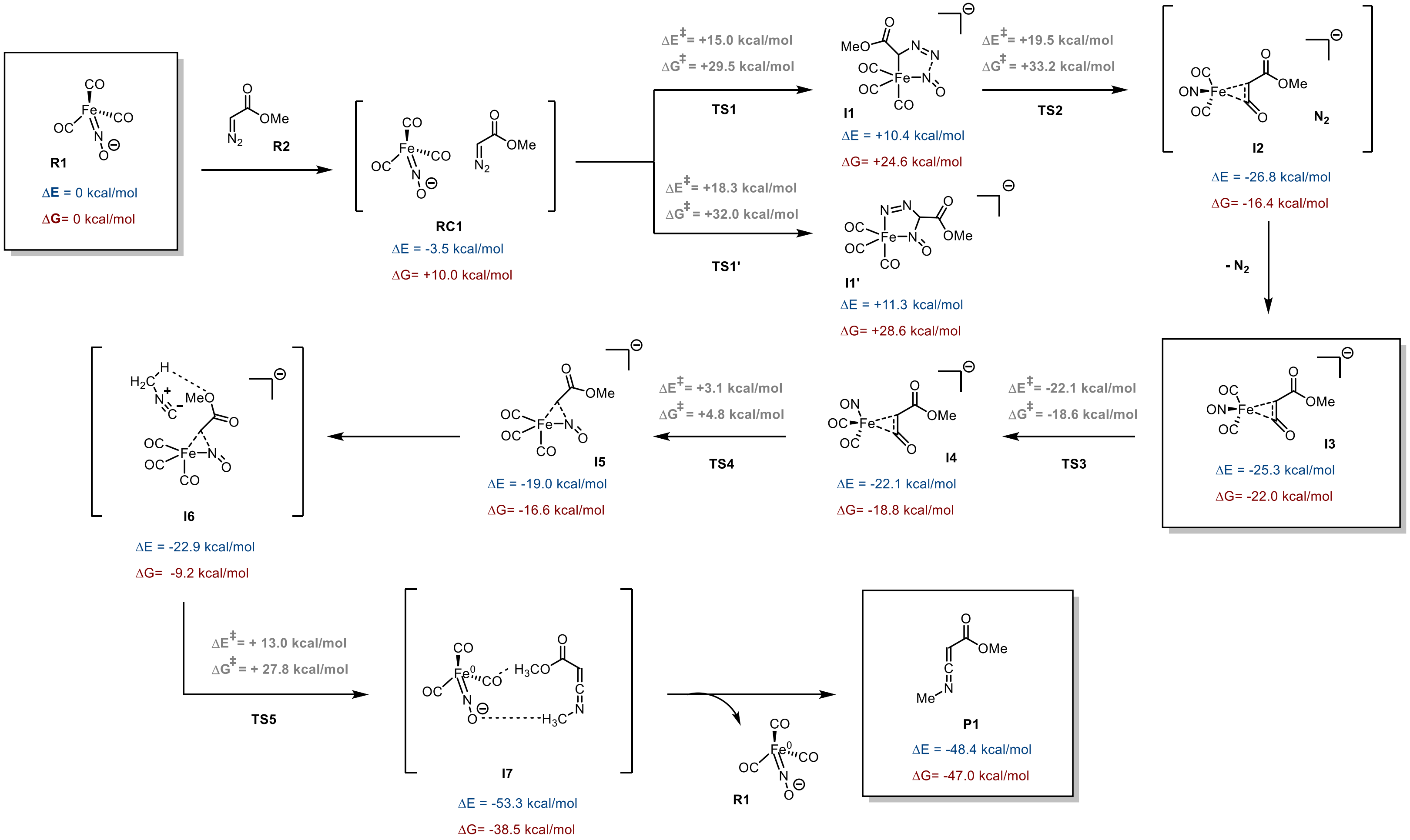

Scheme S6. Roadmap of PES for carbene formation followed by isomerization and carbene transfer to the isocyanide. Relative energies in kcal mol^{-1}. Computed at COSMO(1,2-DCE)-ZORA-BP86-D3(BJ)/TZ2P.

7.2.4 Entire PES of proposed pathway

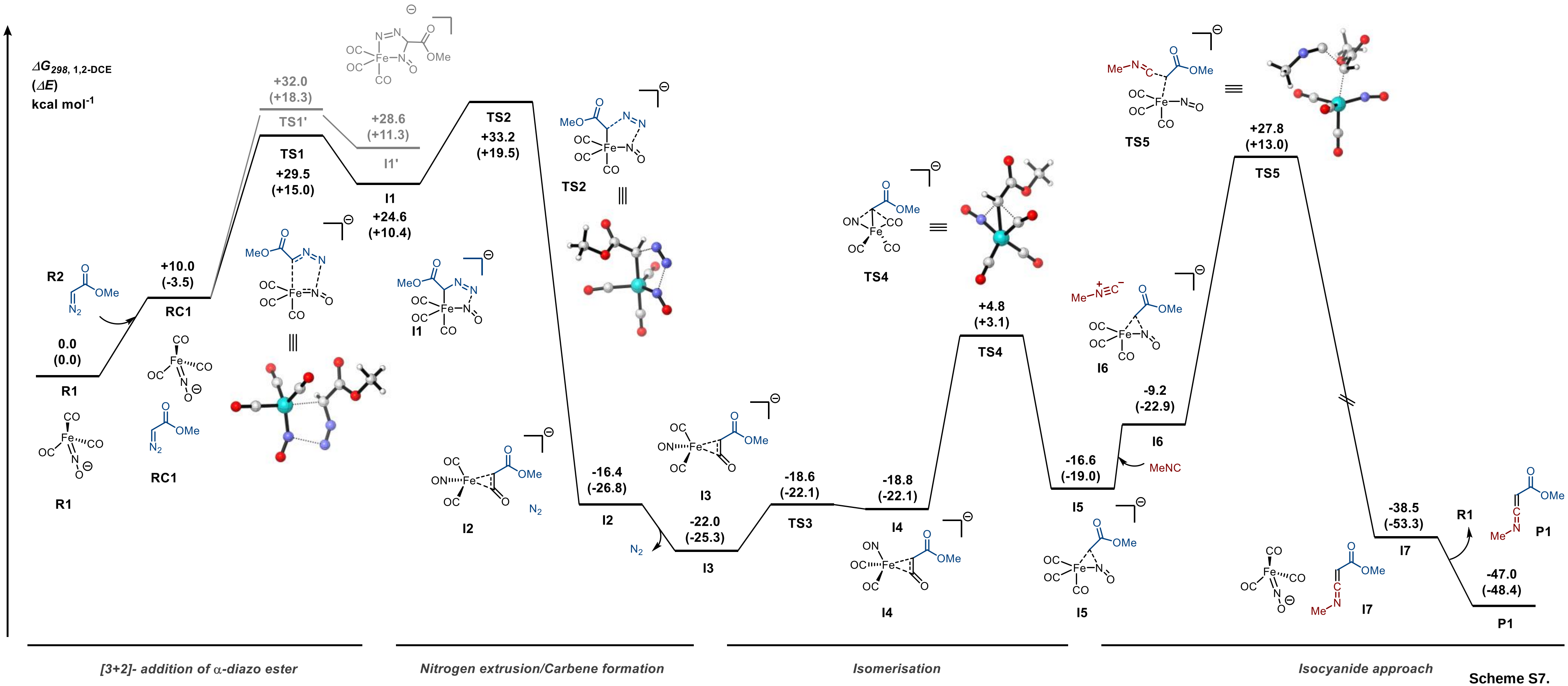

Scheme S7. PES for $[Fe(CO)_3NO]^-$ catalyzed carbene transfer to isocyanides. Computed at COSMO(1,2-DCE)-ZORA-BP86-D3(BJ)/TZ2P. Relative energies in kcal mol^{-1}.

7.3 Computational details

Table S3: Cartesian coordinates (in Å), energies (in kcal mol^{-1}), and number of imaginary frequencies of all stationary points, computed at COSMO(1,2-DCE)-ZORA-BP86-D3(BJ)/TZ2P.

R1, $[Fe(CO)_3NO]^-$
COSMO(1,2-DCE)-ZORA-BP86-D3(BJ)/TZ2P
E = -1722.29
G = -1725.59
N_{imag} = 0

Fe	-0.67526399	-5.09783660	-0.72332704
C	-2.00168159	-5.61672660	-1.76455305
C	0.01312889	-3.77494331	-1.66609910
C	-1.54469632	-4.21180768	0.53026626
N	0.36630652	-6.26738967	-0.20846301
O	-2.11050318	-3.63647298	1.38136260
O	0.49221101	-2.90443939	-2.28853745
O	-2.87641417	-5.98490006	-2.45390321
O	1.12305374	-7.11827276	0.17087026

R1 unrestricted, $[Fe(CO)_3NO]^-$
COSMO(1,2-DCE)-ZORA-(U)BP86-D3(BJ)/TZ2P
E = -1722.29
G = -1725.59
N_{imag} = 0

Fe	-0.67526452	-5.09783660	-0.72332704
C	-2.00168159	-5.61672660	-1.76455358
C	0.01312889	-3.77494331	-1.66609910
C	-1.54469580	-4.21180768	0.53026626
N	0.36630599	-6.26738967	-0.20846301
O	-2.11050265	-3.63647298	1.38136260
O	0.49221048	-2.90443886	-2.28853745
O	-2.87641417	-5.98490006	-2.45390321
O	1.12305374	-7.11827276	0.17087026

MeNC
COSMO(1,2-DCE)-ZORA-BP86-D3(BJ)/TZ2P
E = -821.06
G = -808.69
N_{imag} = 0

C	-0.00435407	-1.55922118	1.13822737
N	-0.65410272	-1.51636259	0.16460533
C	-1.44235557	-1.46517475	-1.01795703
H	-1.61967227	-2.48343818	-1.38089587
H	-0.91147758	-0.88982471	-1.78411515
H	-2.40036741	-0.98282178	-0.79566874

CO
COSMO(1,2-DCE)-ZORA-BP86-D3(BJ)/TZ2P
E = -342.58
G = -351.56
N_{imag} = 0

C	-3.83455089	-0.44381352	-1.26596758
O	-2.90980049	0.18537131	-1.06902210

N_2
COSMO(1,2-DCE)-ZORA-BP86-D3(BJ)/TZ2P
E = -384.07
G = -392.28
N_{imag} = 0

N	-0.95421923	-2.17381183	-1.05547093
N	-0.57207443	-1.23618813	-0.62181021

R2
COSMO(1,2-DCE)-ZORA-BP86-D3(BJ)/TZ2P
E = -1611.64
G = -1585.5
N_{imag} = 0

C	-4.44187011	0.17293935	-0.27217330
C	-3.28483579	1.04363218	-0.24934671
O	-2.13386477	0.37436218	0.02457764
O	-3.34487147	2.25324027	-0.45533424
C	-0.93632404	1.19076461	0.06283133
N	-4.32046945	-1.10845269	-0.04474193
N	-4.20458388	-2.22065089	0.15415303
H	-5.44042804	0.54561028	-0.47418512
H	-1.02504219	1.95960989	0.83819501
H	-0.76745936	1.66325848	-0.91107647
H	-0.12599286	0.49790972	0.29949366

RC1
COSMO(1,2-DCE)-ZORA-BP86-D3(BJ)/TZ2P
E = -3337.42
G = -3301.12
N_{imag} = 0

Fe	-0.36826553	3.39543012	0.21482213
O	0.87301645	1.94456062	2.32710494
N	0.34871667	2.57550866	1.45223160
O	-3.01096700	2.43476181	-0.63109674
C	-1.95857588	2.83174844	-0.30293543
O	1.11484938	3.46396598	-2.31855661
C	0.51193238	3.44218345	-1.31534669
O	-0.87402401	6.25694530	0.65156108
C	-0.68129026	5.11702933	0.46279510
C	0.09470208	-0.27916955	-0.39347977
C	0.38162514	-0.93423009	-1.65087838
O	0.47677438	-2.28936614	-1.52651909
C	0.76031547	-3.00032366	-2.75615071
O	0.52436011	-0.33544279	-2.71260101
N	-0.05975734	-0.97037448	0.70137783
N	-0.19868435	-1.56658786	1.66117193
H	-0.01377078	0.80883784	-0.31560023

H	-0.03341119	-2.82417591	-3.49083601
H	0.79588041	-4.05533527	-2.47544813
H	1.72330369	-2.67956871	-3.16921427

I1
COSMO(1,2-DCE)-ZORA-BP86-D3(BJ)/TZ2P
E = -3323.74
G = -3286.54
N_{imag} = 0

O	1.75563344	7.81807591	10.17192192
N	1.18217680	7.31921897	9.21335814
O	-3.03561217	6.82641952	7.88248169
C	-1.91680719	7.14385970	7.97994238
O	0.27821036	9.28946426	5.75083100
C	0.07589480	8.61219469	6.67934545
O	-1.00643452	9.96468962	9.78405725
C	-0.68935736	9.05141336	9.13620517
C	0.26532013	5.82952003	7.25919614
C	0.15767861	5.84903503	5.77575684
O	1.34445217	6.12786961	5.17718597
C	1.29440417	6.27340922	3.73710692
O	-0.88482484	5.69687012	5.14973808
N	1.57684934	5.37643110	7.72372112
N	2.15200464	5.88324263	8.63922627
H	-0.44143360	5.08257105	7.64573628
H	0.94845881	5.34427670	3.27051338
Fe	-0.23174608	7.67185367	8.14363012
H	2.31983584	6.49720461	3.43456044
H	0.62346621	7.09541035	3.46345669

TS1
COSMO(1,2-DCE)-ZORA-BP86-D3(BJ)/TZ2P
E = -3318.92
G = -3281.59
N_{imag} = 1, ν = -237*i* cm^{-1}

O	-2.53038942	2.97296520	4.57059300
N	-3.73707539	3.07950021	4.52249026
O	-7.30738217	1.79780708	2.46705114
C	-6.52393267	2.03202038	3.30447091
O	-6.65679107	1.87567815	7.10700129
C	-6.14463008	2.08748715	6.07680703
O	-4.21212995	-0.41715304	4.41041318
C	-4.63103192	0.67383944	4.45650557
C	-6.33540112	4.61084539	4.31275458
C	-7.35665863	4.71195528	5.36171981
O	-6.86817461	5.23773782	6.51631252
C	-7.79795273	5.25929174	7.62617176
O	-8.50838848	4.31017643	5.23463896
N	-5.23077226	5.41235307	4.44049902
N	-4.07513761	5.37834920	4.53148998
H	-6.72807124	4.63272423	3.29624590
H	-8.67452367	5.86873190	7.37915342
Fe	-5.27329641	2.28486443	4.54676733
H	-7.24339460	5.70279781	8.45613541
H	-8.11302326	4.23980168	7.87400449

I1’
COSMO(1,2-DCE)-ZORA-BP86-D3(BJ)/TZ2P
E = -3322.63
G = -3282.45
N_{imag} = 0

O	-7.58930610	4.17851026	8.86730716
N	-7.30744673	3.36323656	7.92685977
O	-10.01225244	0.78705267	6.11741979
C	-9.22774563	1.64782661	6.17286698
O	-7.56575295	3.71263005	3.50552067
C	-7.75244032	3.40483889	4.61449460
O	-10.08769882	5.09038472	6.63148000
C	-9.29329799	4.25275382	6.49697956
C	-6.13755393	2.49690150	8.30729321
C	-6.40945259	1.67590581	9.56554802
O	-7.61880614	1.10268360	9.51826128
C	-7.96034716	0.25848402	10.65041740
O	-5.59711953	1.52463363	10.46278968
N	-5.81167708	1.59283081	7.19083142
N	-6.55925895	1.68045250	6.20720635
H	-5.27172899	3.14216591	8.50504144
H	-7.24605741	-0.56784525	10.73010990
Fe	-8.06031139	2.97080562	6.31583532
H	-8.96389576	-0.11353497	10.43621334
H	-7.95242644	0.84958925	11.57242349

TS1’
COSMO(1,2-DCE)-ZORA-BP86-D3(BJ)/TZ2P
E = -3315.65
G = -3279.06
N_{imag} = 1, ν = -180*i* cm^{-1}

O	-7.07024199	4.31583333	9.23024388
N	-7.20311151	3.69171009	8.20406343
O	-9.99028788	0.97712307	6.96485053
C	-9.28879893	1.90705201	6.89381378
O	-8.43658919	4.41625741	4.10120329
C	-8.34770012	3.96667215	5.17427572
O	-10.34129694	5.20841347	7.78035442
C	-9.50729778	4.48033812	7.41382564
C	-5.60180592	2.15474134	8.08284640
C	-6.18630067	1.37806900	9.15685176
O	-7.08706401	0.45413036	8.72399856
C	-7.84231525	-0.20707339	9.76302849
O	-5.94269024	1.58292885	10.34868320
N	-5.82122026	1.81569909	6.77821449
N	-6.73077481	2.03655437	6.01772069
H	-4.64853075	2.63776160	8.29193013
H	-7.17835078	-0.78877885	10.41303274
Fe	-8.27188543	3.35941220	6.85561406
H	-8.53798007	-0.86580059	9.23828526
H	-8.39034704	0.52926558	10.36207614

I2
COSMO(1,2-DCE)-ZORA-BP86-D3(BJ)/TZ2P
E = -3361.09
G = -3327.43
$\boldsymbol{N}_{\mathbf{imag}}$ = 0

O	-4.20401184	4.84448295	7.16932831
N	-5.33294897	4.71163566	6.81151138
O	-8.51196731	2.56084992	7.76499716
C	-7.87158774	3.39070880	7.25643088
O	-6.52349715	5.87457825	3.74578512
C	-6.90740040	5.03075439	4.52731742
O	-8.55748396	6.96308784	6.98180113
C	-7.93526575	6.00526756	6.71845768
C	-7.50220406	3.71734290	4.55809860
C	-6.78161075	2.52467008	4.13562521
O	-5.43661585	2.72309142	4.01190887
C	-4.67791702	1.57635594	3.57507149
O	-7.31038102	1.44019864	3.88294203
N	-5.14061793	1.42925737	7.00297669
N	-4.10058204	1.60249465	7.32399622
H	-8.58411586	3.63643647	4.46855123
H	-4.84186829	0.72454362	4.24477700
Fe	-6.91594926	4.62109556	6.37537569
H	-3.63180299	1.89108038	3.60738782
H	-4.95943771	1.29303340	2.55359067

I3
COSMO(1,2-DCE)-ZORA-BP86-D3(BJ)/TZ2P
E = -2974.86
G = -2941.85
$\boldsymbol{N}_{\mathbf{imag}}$ = 0

O	-4.19728335	4.81648525	7.13398403
N	-5.32920663	4.68433647	6.78353431
O	-8.50317503	2.52209510	7.72406054
C	-7.86744587	3.36204115	7.22528827
O	-6.52891328	5.91061575	3.75261997
C	-6.91187830	5.05226862	4.51883047
O	-8.54100697	6.94575358	7.02582868
C	-7.92399269	5.99102264	6.73987084
C	-7.51102227	3.74086377	4.52708564
C	-6.79073218	2.54435970	4.11554240
O	-5.44712637	2.74416590	3.98332907
C	-4.67859172	1.57997181	3.61153022
O	-7.31787893	1.45310422	3.88741464
H	-3.64220239	1.92266909	3.55820397
H	-5.00422039	1.19403228	2.63834422
H	-8.59314256	3.66435480	4.43544537
H	-4.78166644	0.79347212	4.36872830
Fe	-6.91658004	4.60760101	6.35931675

TS2
COSMO(1,2-DCE)-ZORA-BP86-D3(BJ)/TZ2P
E = -3314.78
G = -3277.86
$\boldsymbol{N}_{\mathbf{imag}}$ = 1, ν = -360*i* cm^{-1}

O	-4.72478889	4.94103451	8.09198793
N	-5.44456568	4.25222782	7.41390290

O	-9.60783616	3.76271879	7.35313007
C	-8.53448539	4.00278585	6.97104137
O	-6.91438290	5.16566230	3.55467489
C	-6.88126011	4.80895717	4.66269576
O	-7.22579839	7.20722904	7.10164072
C	-7.08445728	6.08934324	6.82245952
C	-6.84183958	2.44064414	5.88957778
C	-6.49556030	1.97410617	4.52504778
O	-5.33202291	2.51161792	4.06553675
C	-4.93052082	2.09699170	2.74202538
O	-7.12764766	1.14342019	3.86667036
N	-5.47930140	1.58963987	6.88350064
N	-4.82150714	2.24261598	7.56734683
H	-7.67365959	1.81045335	6.22771302
H	-4.80889155	1.00839110	2.69880325
Fe	-6.90251557	4.37804023	6.38653392
H	-3.97694560	2.59756794	2.55665725
H	-5.67685119	2.40902951	2.00137520

I4
COSMO(1,2-DCE)-ZORA-BP86-D3(BJ)/TZ2P
E = -2971.98
G = -2937.63
N_{imag} = 0

O	-5.93748896	1.60414727	7.32789520
N	-6.29347546	2.71559192	7.09468151
O	-8.78590701	5.49529419	8.35195418
C	-8.01932079	4.99496825	7.62218917
O	-7.61432559	6.18210853	4.51732973
C	-7.46143307	5.09635331	5.03651713
O	-4.56468897	6.10101054	6.52316324
C	-5.46336152	5.35545171	6.55843555
C	-7.72907873	3.69745537	4.82008894
C	-6.91707323	2.87782495	3.93088125
O	-5.75327549	3.48277981	3.55363452
C	-4.89148236	2.69760096	2.70241647
O	-7.23466793	1.75598408	3.52996072
H	-4.01346095	3.32344985	2.52406523
H	-5.39127277	2.46126353	1.75554063
H	-8.74265735	3.32551523	4.97180617
H	-4.60099106	1.76664701	3.20367006
Fe	-6.80700067	4.21776568	6.60326057

TS3
COSMO(1,2-DCE)-ZORA-BP86-D3(BJ)/TZ2P
E = -2971.94
G = -2937.47
N_{imag} = 1, ν = -14*i* cm^{-1}

O	-6.87985250	1.47898998	8.14622225
N	-7.04962366	2.49565053	7.55046343
O	-9.61490015	5.36032702	7.61084361
C	-8.70407451	4.74988353	7.20699408
O	-8.50951404	5.07178626	4.15512909
C	-7.99639736	4.20931314	4.83786612
O	-5.16228350	5.78047890	6.44315112
C	-6.03635461	5.00423785	6.46242587
C	-7.48863860	2.86441455	4.75744600
C	-6.23853787	2.52361225	4.09009110
O	-5.48715016	3.61276849	3.75952044

C	-4.21193362	3.32685299	3.14698830
O	-5.88415530	1.37424147	3.81767755
H	-3.74786584	4.30147570	2.97674299
H	-4.34719132	2.79614910	2.19719299
H	-8.19615541	2.03750583	4.82055885
H	-3.59144318	2.72079215	3.81798130
Fe	-7.34000119	3.82073778	6.59166894

TS4
COSMO(1,2-DCE)-ZORA-BP86-D3(BJ)/TZ2P
E = -2946.78
G = -2913.9
N_{imag} = 1, ν = -461*i* cm^{-1}

O	-6.50587873	1.56012131	6.89211459
N	-6.67141436	2.74289165	6.65209727
O	-8.25658956	5.75382636	8.77474560
C	-7.69528071	5.22354106	7.89350309
O	-8.27008676	5.57362671	4.28632747
C	-7.70422275	4.98153826	5.15540473
O	-4.41219439	6.10074171	6.49922750
C	-5.37598696	5.43683122	6.51980932
C	-7.51616640	3.16784105	5.15635672
C	-6.77990574	2.69514716	3.98799376
O	-5.51334866	3.17017631	3.90831238
C	-4.75517583	2.72446464	2.75950252
O	-7.27016937	1.93944929	3.14521714
H	-3.78291378	3.21435891	2.84889325
H	-5.25471436	3.02532622	1.83157387
H	-8.51065918	2.73464548	5.25042379
H	-4.63972312	1.63454744	2.77729504
Fe	-6.83158307	4.45254785	6.57699115

I5
COSMO(1,2-DCE)-ZORA-BP86-D3(BJ)/TZ2P
E = -2968.88
G = -2935.4
N_{imag} = 0

Fe	0.41696201	1.95467901	-0.25624983
O	2.35057289	3.18589076	1.55355581
N	1.78220111	2.41639460	0.72584275
O	-1.20071685	-0.45447485	0.23752913
C	-0.58012798	0.50922405	0.02074533
O	0.39378246	2.14302430	-3.19101584
C	0.36490261	2.06528181	-2.02655292
O	-1.55516133	4.02560503	0.37015205
C	-0.78070255	3.19245044	0.11350110
C	2.32260640	1.28784482	0.03291959
C	2.54611551	0.01636957	0.71721927
H	3.04667694	1.49423610	-0.75787702
O	1.92601772	-0.07702227	1.92445717
C	2.07392539	-1.34489224	2.60177596
H	1.66496137	-2.15640666	1.98862097
H	1.50578751	-1.24419775	3.52974429
H	3.12942491	-1.54688130	2.81827770
O	3.22875253	-0.89944356	0.25227836

I6
COSMO(1,2-DCE)-ZORA-BP86-D3(BJ)/TZ2P
E = -3793.91
G = -3736.81
N_{imag} = 0

Fe	1.98725199	3.53528001	2.57567165
O	0.66431055	0.97885719	2.09461781
N	1.52337895	1.90218516	2.17672230
O	3.01129350	5.53007966	0.67034529
C	2.59514960	4.76790731	1.44998048
O	3.41862501	4.21215164	5.05466103
C	2.86252750	3.96016963	4.06076092
O	-0.58518109	4.75812388	3.24873055
C	0.44717961	4.28515217	2.98525057
C	2.92797137	1.84748465	1.91417526
C	3.42783693	1.79484157	0.54346923
H	3.54918838	1.31384647	2.63582269
O	2.49404236	2.09698429	-0.39793227
C	2.97350972	2.12049828	-1.76100333
H	3.76869056	2.86670641	-1.87394404
H	2.10605862	2.39194449	-2.36744200
H	3.35355897	1.13508247	-2.05443156
O	4.59315553	1.51394901	0.24941551
C	6.02279391	1.81969967	4.42776225
N	6.04826480	2.58638431	3.54316793
C	6.05860069	3.50132112	2.45622471
H	7.07215814	3.89343773	2.31949537
H	5.36826362	4.32401441	2.67183743
H	5.72957894	2.97443208	1.55185980

TS5
COSMO(1,2-DCE)-ZORA-BP86-D3(BJ)/TZ2P
E = -3757.48
G = -3699.71
N_{imag} = 1, ν = -534*i* cm^{-1}

Fe	1.87173207	3.37433976	2.10869657
O	0.31386347	0.97878363	1.71809870
N	1.08008561	1.89396704	1.81394751
O	2.84691677	5.20749958	0.02418234
C	2.46936418	4.48913940	0.85698185
O	3.50461578	3.81173753	4.51241737
C	2.86779387	3.66708165	3.54880578
O	-0.35959126	5.05768210	2.96064172
C	0.53276133	4.39246348	2.61950075
C	3.28953435	1.86034736	1.48539143
C	3.02702964	1.19475091	0.20484450
H	3.35316685	1.11622418	2.28285779
O	2.58142116	2.03852820	-0.77643632
C	2.36122787	1.42822177	-2.06283225
H	3.29524153	1.01871429	-2.46653573
H	1.98883370	2.23060842	-2.70571113
H	1.61765716	0.62465665	-1.98909935
O	3.29405194	0.01187156	-0.04201085
C	5.21451118	2.15858528	1.36788816
N	5.78903887	3.18948070	1.63773309
C	5.72291712	4.57278379	2.03145998
H	4.69492848	4.94956485	2.04185196
H	6.32402751	5.17191082	1.33676884
H	6.15731923	4.67973157	3.03292838

I7
COSMO(1,2-DCE)-ZORA-BP86-D3(BJ)/TZ2P
E = -3824.18
G = -3765.99
$\boldsymbol{N_{imag}}$ = 0

Fe	1.35057613	3.43699540	2.17217561
O	-0.24214726	1.31029781	1.14792031
N	0.43910702	2.20000980	1.57846894
O	2.81371884	5.01794354	0.17547728
C	2.25070657	4.38418821	0.98441567
O	3.37030531	2.71551625	4.17898864
C	2.57936054	3.01435479	3.36561892
O	-0.10400978	5.55544100	3.59010595
C	0.49078223	4.71566270	3.02509391
C	4.76307909	1.22638194	1.24578317
C	3.70530306	0.88836206	0.30728527
H	5.05672641	0.47072906	1.97147404
O	3.48223106	1.87394245	-0.60225764
C	2.36095852	1.65856838	-1.48838340
H	2.53379785	0.78084648	-2.12204295
H	2.29881089	2.56338574	-2.09741875
H	1.44260217	1.52247988	-0.90764899
O	3.08290758	-0.17226676	0.31635854
C	5.30059296	2.44246293	1.32258954
N	5.88004995	3.49913403	1.33055524
C	5.60336753	4.74267984	2.03821334
H	5.52822436	5.54711916	1.29898717
H	6.45569262	4.95955307	2.69194511
H	4.67952413	4.68525353	2.62415169

P1
COSMO(1,2-DCE)-ZORA-BP86-D3(BJ)/TZ2P
E = -2097.02
G = -2048.95
$\boldsymbol{N_{imag}}$ = 0

N	-3.54782945	-0.04844935	0.33972119
C	-2.35028766	0.07385568	0.36175401
C	-4.61866663	0.93642829	0.41490880
C	-1.01705902	0.08236273	0.36611284
C	-0.22672703	0.21254297	-0.84921089
O	-0.99825100	0.30823038	-1.96840270
O	1.00217221	0.23523779	-0.87873580
C	-0.27229237	0.43864769	-3.21396943
H	-5.23611113	0.70651402	1.29043990
H	-5.24559240	0.82902913	-0.47735965
H	-4.23182698	1.96006604	0.48401934
H	-0.48330972	0.00391538	1.31104977
H	0.36758501	-0.43588380	-3.37786620
H	-1.04003166	0.50178224	-3.98868857
H	0.34073668	1.34720369	-3.20635669

I8
COSMO(1,2-DCE)-ZORA-BP86-D3(BJ)/TZ2P
E = -2525.68
G = -2506.2
N_{imag} = 0

Fe	-0.07205277	-2.89033788	-1.63857712
C	-1.52378218	-2.87530131	-0.65234109
C	0.29574550	-1.29656143	-2.27373213
N	0.97507939	-2.42148052	-0.01647805
C	-0.88298139	-3.46703838	-3.12221910
O	0.72200780	-1.42154409	0.64364671
O	0.48581061	-0.20839422	-2.67745994
O	-2.50589381	-2.80476516	-0.00942465
O	-1.44711128	-3.79981305	-4.09325664
C	1.10504849	-4.36285126	-1.72027256
N	1.83544163	-5.28692855	-1.77684266
C	2.69078568	-6.41184500	-1.86178619
H	2.13732665	-7.32638810	-1.61227913
H	3.52520712	-6.29724373	-1.15930820
H	3.09181840	-6.50185751	-2.8794077

I9
COSMO(1,2-DCE)-ZORA-BP86-D3(BJ)/TZ2P
E = -2525.6
G = -2505.87
N_{imag} = 0

Fe	0.54525309	-2.88459895	-2.24115016
C	-1.16990127	-3.24761346	-2.04297647
C	0.87551100	-1.17388598	-2.51924495
N	0.66603143	-2.55709488	-0.30039168
C	0.51631397	-3.27437977	-4.06866524
O	-0.04575266	-1.72153571	0.24328922
O	1.07229930	-0.02900949	-2.69538317
O	-2.31696273	-3.46495077	-1.90905547
N	0.47244042	-3.49488950	-5.22578424
C	1.83569246	-4.12095377	-2.06444519
O	2.67194115	-4.94324065	-2.04000355
C	0.43071321	-3.79193996	-6.60966361
H	1.30733653	-3.36374669	-7.11204011
H	-0.47773378	-3.36922844	-7.05681465
H	0.42959770	-4.87899748	-6.75954776

P2
COSMO(1,2-DCE)-ZORA-BP86-D3(BJ)/TZ2P
E = -2191.17
G = -2172.48
N_{imag} = 0

Fe	-1.41285711	-5.72655892	-0.69067257
C	-2.89525552	-6.08163630	-1.55583921
C	-0.78350613	-4.39481356	-1.63937724
C	-2.06456266	-4.84323251	0.77478793
N	-0.39259551	-6.97502237	-0.36355215
N	-2.46404066	-4.28859870	1.74173440
O	-0.33262651	-3.52976493	-2.30380897
O	-3.86861826	-6.35333916	-2.16590486
O	0.36773689	-7.89849058	-0.16814341
C	-3.00485659	-3.64763016	2.88043115

H	-2.48197923	-3.98255911	3.78589940
H	-4.07241023	-3.88576996	2.98082348
H	-2.89167299	-2.55891578	2.79225435

I10
COSMO(1,2-DCE)-ZORA-BP86-D3(BJ)/TZ2P
E = -1321.29
G = -1327.96
$\boldsymbol{N}_{\mathbf{imag}}$ = 0

Fe	-0.72105369	-5.03199954	-0.56485593
C	-2.08431103	-5.51305285	-1.53452607
C	-0.05960546	-3.70754730	-1.48250107
O	-2.85848988	-5.93355400	-2.32789606
N	0.33481730	-6.22885816	-0.22444681
O	1.13926773	-7.13717603	-0.28561546
O	0.46753865	-2.97137079	-2.24816969

I11
COSMO(1,2-DCE)-ZORA-BP86-D3(BJ)/TZ2P
E = -2955.30
G = -2922.56
$\boldsymbol{N}_{\mathbf{imag}}$ = 0

Fe	-0.19447368	-2.81936834	-0.33726104
C	-1.90141468	-3.23076710	-0.47147097
C	-0.00011854	-2.45657820	-2.05804849
O	-3.01700438	-3.57175144	-0.59048239
N	0.88453876	-3.95991245	0.16854982
O	1.60996557	-4.88666473	0.39676437
O	0.11903418	-2.20044532	-3.19247531
C	0.13076868	-0.87096378	0.30071606
C	-0.63467927	0.17551221	-0.35827626
O	-1.97114754	0.10670647	-0.11484363
C	-2.78045846	1.06835587	-0.82861531
O	-0.12641197	1.04182769	-1.07209610
N	-0.49286295	-1.66939482	1.28588422
N	-0.86757228	-1.72007888	2.40147603
H	1.18873998	-0.66759092	0.45965285
H	-2.50392739	2.09114323	-0.54816779
H	-3.80984678	0.85480482	-0.53134631
H	-2.65938960	0.93969172	-1.91065200

I12
COSMO(1,2-DCE)-ZORA-BP86-D3(BJ)/TZ2P
E = -2587.56
G = -2558.31
$\boldsymbol{N}_{\mathbf{imag}}$ = 0

Fe	0.75469137	2.49457894	0.30808644
O	2.07906529	4.71676021	1.49886534
N	1.61985060	3.71907860	1.00762802
O	-1.52869877	1.38058418	1.76787417
C	-0.61534472	1.84526316	1.19749839
O	-0.96157479	3.16115437	-1.95288775
C	-0.27088105	2.88771051	-1.04482759
H	-0.74839998	-1.64543632	-1.93743313
O	1.48714195	-1.24435545	-0.51781631
C	1.72929768	1.11075037	-0.35371845
C	1.24662191	-0.11930089	-0.97386334
H	2.81477243	1.06619101	-0.17655310

O	0.45796583	0.06534121	-2.06477063
C	-0.08572830	-1.14110980	-2.65064389
H	0.71904070	-1.82459350	-2.94638780
H	-0.64855483	-0.80897807	-3.52633798

I13
COSMO(1,2-DCE)-ZORA-BP86-D3(BJ)/TZ2P
E = -3457.79
G = -3400.61
N_{imag} = 0

Fe	0.14183325	1.89216149	-0.21695472
O	-0.48293665	0.79800664	2.32279850
N	-0.20948750	1.18356198	1.22489284
N	-2.19108840	0.85345118	-1.79969994
C	-0.97349345	1.03082927	-1.53237443
O	2.92064703	2.82034202	-0.52931638
C	1.81865666	2.46240973	-0.38181088
O	-1.16910592	4.43831087	-0.78233823
C	-0.62441376	3.41906529	-0.57776997
C	0.35414021	0.66796505	-1.92021847
C	0.95638303	-0.62609972	-1.62017605
H	0.83240104	1.19120330	-2.74732615
O	0.18080504	-1.40925024	-0.81652520
C	0.76208927	-2.67171414	-0.43299396
H	0.96289244	-3.29231941	-1.31448678
H	0.01784491	-3.15073434	0.20832384
H	1.69588278	-2.51343723	0.12030897
O	2.04634574	-1.00976590	-2.05380026
C	-2.52937182	-0.05962980	-2.89815171
H	-3.14124408	-0.88358042	-2.50437666
H	-1.63658001	-0.47729562	-3.39214234
H	-3.13965231	0.47526411	-3.63942950

P3
COSMO(1,2-DCE)-ZORA-BP86-D3(BJ)/TZ2P
E = -1611.52
G = -1585.59
N_{imag} = 0

O	-3.49572137	0.09392737	0.32360245
C	-2.33417210	0.06178885	0.35390843
H	0.34580037	1.38557168	-3.15877519
C	-1.00857155	0.02495653	0.38346774
C	-0.21409346	0.17089725	-0.83100084
O	-0.99812770	0.34433984	-1.92891020
O	1.00972039	0.14154855	-0.86541271
C	-0.29097179	0.49442403	-3.18590240
H	0.31886525	-0.39308342	-3.38693365
H	-1.07206434	0.60340860	-3.94134256
H	-0.51488519	-0.11863624	1.34157059

I14
COSMO(1,2-DCE)-ZORA-BP86-D3(BJ)/TZ2P
E = -2931.67
G = -2903.00
N_{imag} = 0

Fe	-0.13144127	0.26568030	-0.71005157
O	-1.29911788	-0.92010105	1.60337467

N	-0.73974741	-0.37459661	0.68908345
O	3.13269998	-1.65217910	0.32672301
C	2.58737652	-1.03064353	1.10913321
O	-1.95130551	1.56639259	-2.58381992
C	-1.21753251	1.04373008	-1.83199140
O	0.80191938	-1.56873261	-2.79149181
C	0.40988585	-0.85036238	-1.95104039
C	1.16406445	1.52035153	-0.50203359
C	1.95282213	2.18427631	-1.53474568
H	1.56072299	1.73200230	0.50200978
O	1.21984026	2.85019979	-2.46716121
C	1.98158926	3.45826409	-3.53643468
H	2.72109484	4.16089077	-3.13440076
H	1.24737758	3.98388153	-4.15174818
H	2.49365341	2.68790855	-4.12511363
O	3.18546530	2.10732812	-1.59908886

8. References

[1] Dar'In, D.; Kantin, G.; Krasavin, M. A.; *Chem. Commun.*, **2019**, *55*, 5239–5242.

[2] Dar'In, D.; Kantin, G.; Krasavin, M.; *Synthesis,* **2019**, *51*, 4284–4290.

[3] Xiao, M.Y.; Zheng, M.M.; Peng, X.; Zhang, F.G.; Ma, J.A.; *Org. Lett.*, **2020**, *22*, 7762-7776.

[4] Matheis, C.; Krause, T.; Bragoni, V.; Goossen, L. J.; *Chem. A Eur. J.*, **2016**, *22*, 12270-12273.

[5] Takamura, N.; Mizoguchi, T.; *Tetrahedron*, **1975**, *31*, 227-230.

[6] Yu, Z.; Pan, Y.; Wang, Z.; Wang, J.; Lin, Q.; *Angew. Chem. Int. Ed.*, **2012**, *51*, 10600–10604.

[7] Chen, K.; Zhang, S. Q.; Brandenberg, O. F.; Hong, X.; Arnold, F. H.; *J. Am. Chem. Soc.*, **2018**, *140*, 16402–16407.

[8] Peng, C.; Cheng, J.; Wang, J.; *J. Am. Chem. Soc.*, **2007**, *129*, 8708–8709.

[9] Abid, I.; Gosselin, P.; Mathé-Allainmat, M.; Abid, S.; Dujardin, G.; Gaulon-Nourry, C.; *J. Org. Chem.* **2015**, *80*, 9980–9988.

[10] Klein. J.E.M.N.; Rommel, S.; Plietker, B.; *Organometallics*, **2014**, *33*, 5802-5810.

[11] Fuks, R.; Baudoux, D.; Piccinni-Leopardi, C.; Declercq, J.P.; van Meerssche, M., *J. Org. Chem.*, **1988**, *1*, 18-22.

[12] a) G. te Velde, F. M. Bickelhaupt, E. J. Baerends, C. Fonseca Guerra, S. J. A. van Gisbergen, J. GG. Snijders, T. Ziegler, *J. Comput. Chem.* **2001**, *22*, 931-967; b) C. Fonseca Guerra, J. G. Snijders, G. te Velde, E. J. Baerends, *Theor. Chem. Acc.* **1998**, *99*, 391-403; c) ADF2019.303, SCM Theoretical Chemistry, Vrije Universiteit: Amsterdam (The Netherlands), **2021**. http://www.scm.com.

[13] a) J. C. Slater, *Quantum Theory of Molecules and Solids*, McGraw-Hill, New York, **1974**; b) A. D. Becke, *J. Chem. Phys.* **1986**, *84*, 4524-4529; c) A. D. Becke, *Phys. Rev. A* **1988**, *38*, 3098-3100; d) C. Lee, W. Yang, R. G. Parr, *Phys. Rev. B* **1988**, *37*, 785-789.; e) J. P. Perdew, *Phys. Rev. B,* **1986**, *33*, 8822–8824; f) J. P. Perdew, *Phys. Rev. B*, **1986**, *34*, 7406– 7406.

[14] A. Klamt, G. Schüürmann, *J. Chem. Soc. Perkin Trans. 2,* **1993**, 799-805; b) A. Klamt, *J. Phys. Chem.* **1995**, *99*, 2224-2235; c) A. Klamt, V. Jonas, *J. Chem. Phys.* **1996**, *105*, 9972-9981; d) C. C. Pye, T. Ziegler, *Theor. Chem. Acc.* **1999**, *101*, 396-408.

[15] S. Grimme, J. Antony, S. Ehrlich, H. Krieg, *J. Chem. Phys.* **2010**, *132*, 154104; b) A. D. Becke, E. R. Johnson, *J. Chem. Phys.* **2005**, *123*, 154101.

[16] E. van Lenthe, E. J. Baerends, J. G. Snijders, *J. Chem. Phys.* **1993**, *99*, 4597-4610; b) E. van Lenthe, E. J. Baerends, J. G. Snijders, *J. Chem. Phys.* **1994**, *101*, 9783-9792.

[17] C. Y. Legault*, CYLView* (Unversité de Sherbrooke, Sherbrooke, QC, Canada), 1.0b, **2009**.

[18] L. Fan, T. Ziegler, *J. Chem. Phys.* 1990, **92**, 3645–3652.

[19] a) Klein, J. et al., *Angew. Chem. Int. Ed*., **2014**, *53*, 1790-1794.; b) Klein, J.E.M.N.; Knizia, G.; Miehlich, B.; Kastner, J.; Plietker, B., *Chem. Eur. J.*, **2014**, *20*, 7254-7257.; c) Klein, J.E.M.N.; Miehlich, B.; Kastner, J.; Plietker, B., *Dalton Trans.*, **2013**, *42*, 7519-7525.

[20] Clarkson, L.M.; *Acta Crystallogr. Sect.C,* **1992**, *48*, 236-239.

[21] Pannell, K.H. et al., *Inorg. Chem.*, **1983**, *22*, 418-427.

9. ^{1}H- & ^{13}C-NMR spectra

^{1}H-NMR 500 MHz, $CDCl_3$ **4aa**

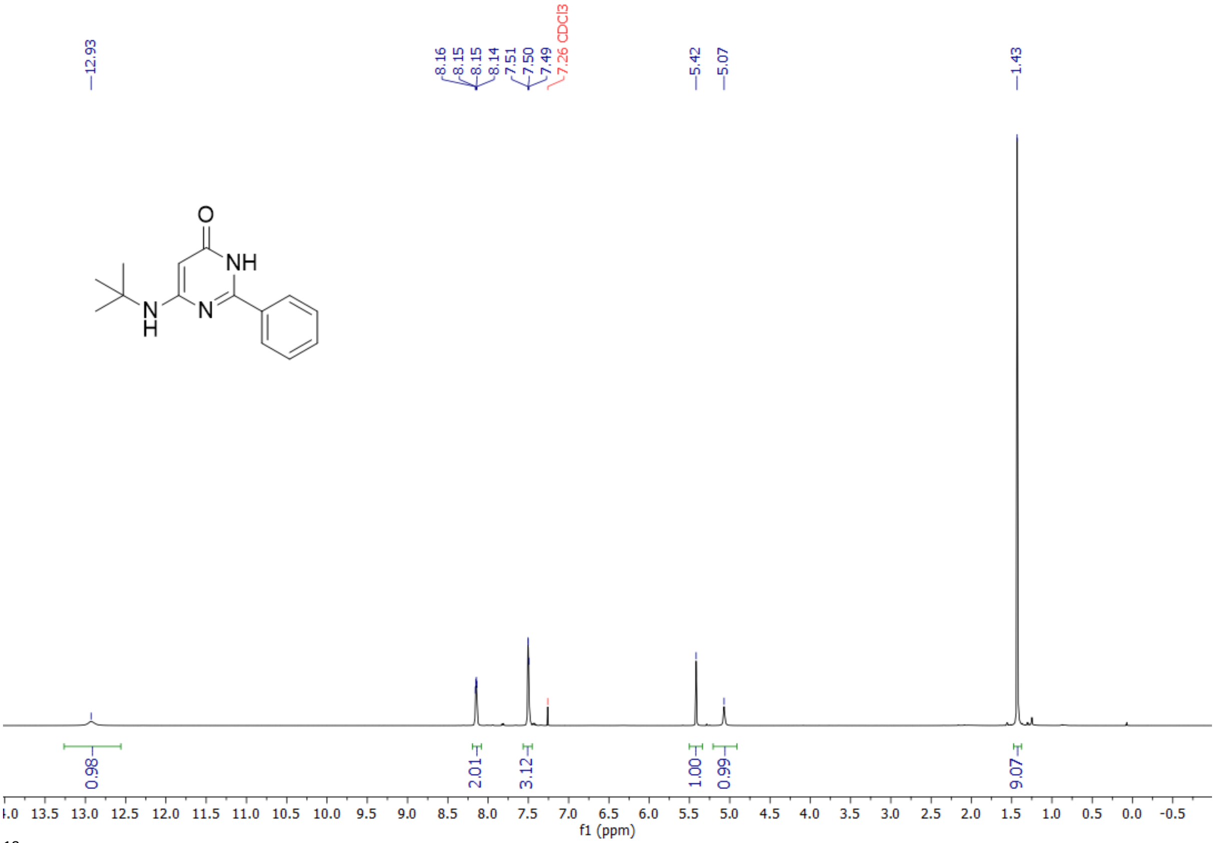

^{13}C-NMR 125.7 MHz, $CDCl_3$ **4aa**

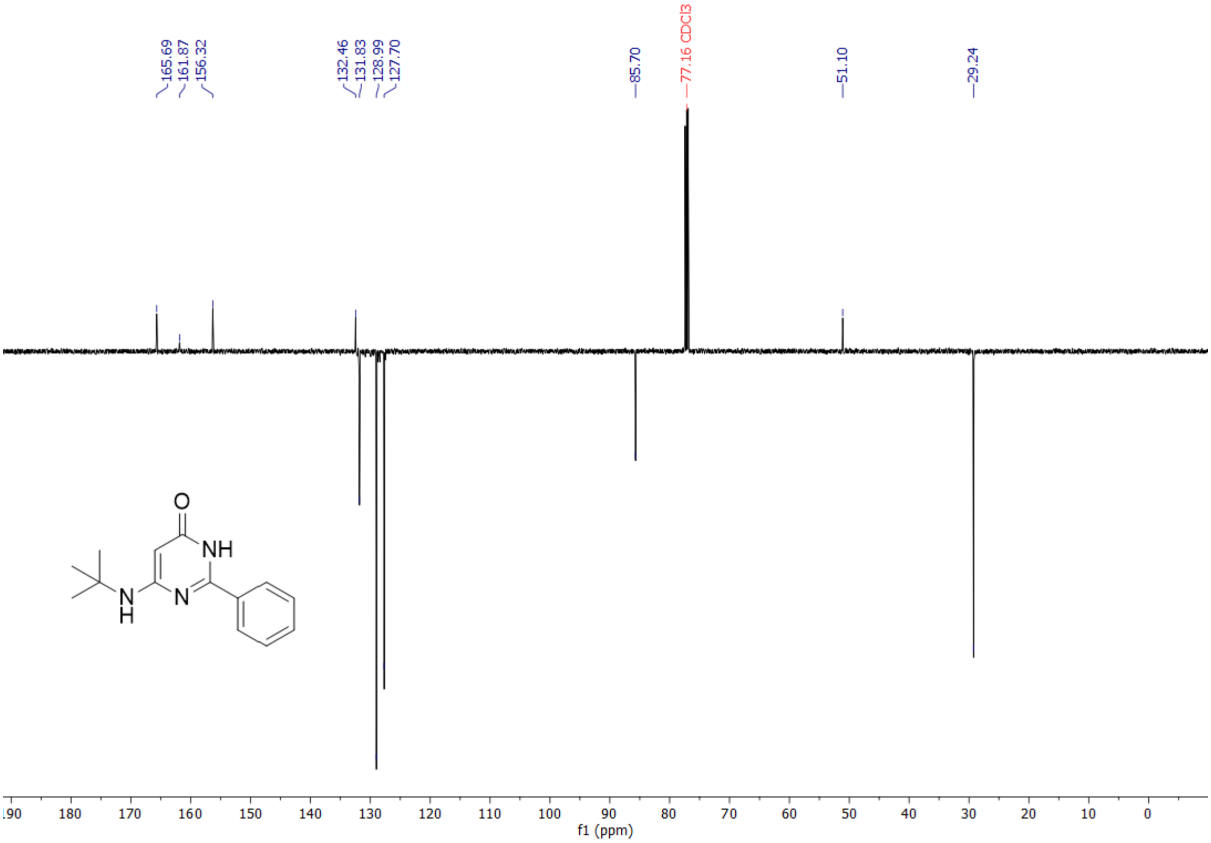

^{1}H-NMR 500 MHz, DMSO-*d*6 **4ba**

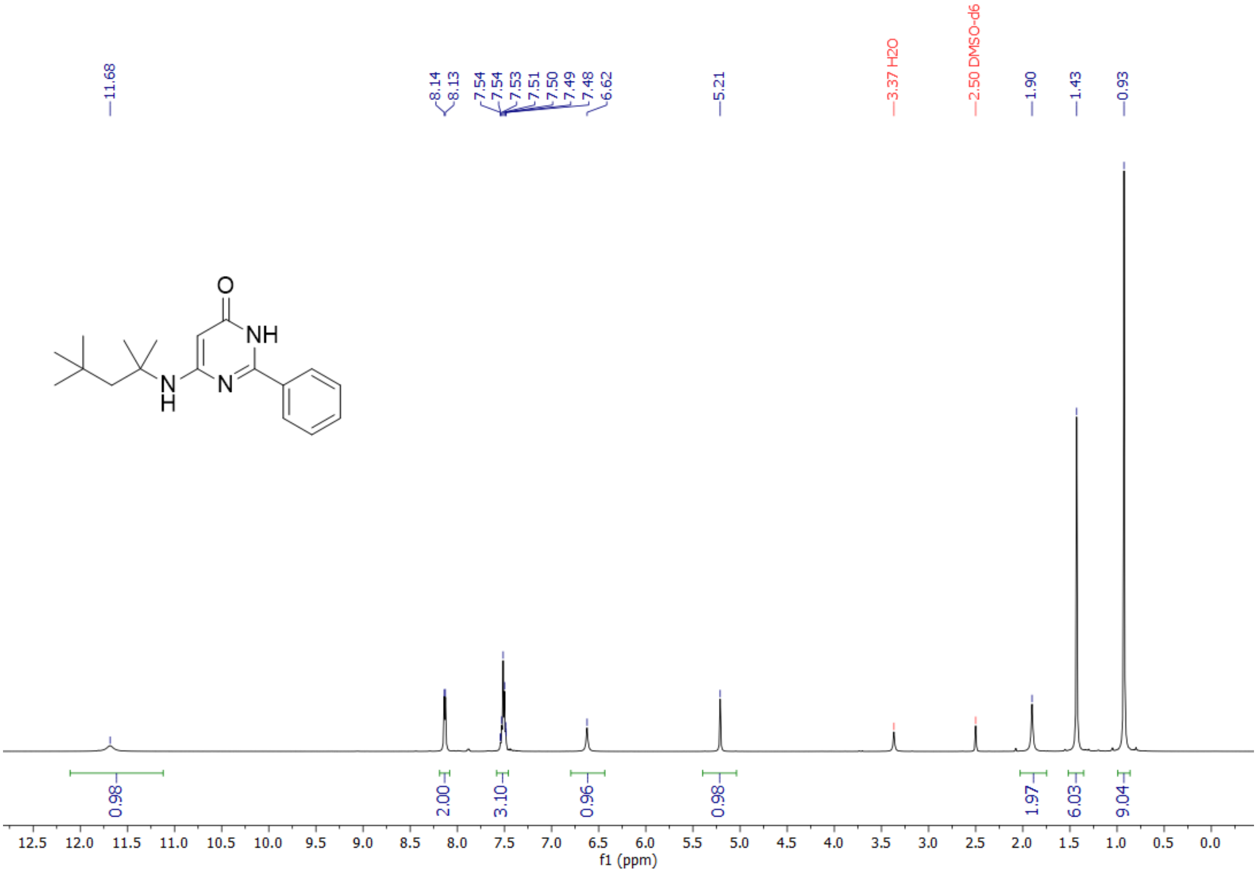

^{13}C-NMR 125.7 MHz, DMSO-*d*6 **4ba**

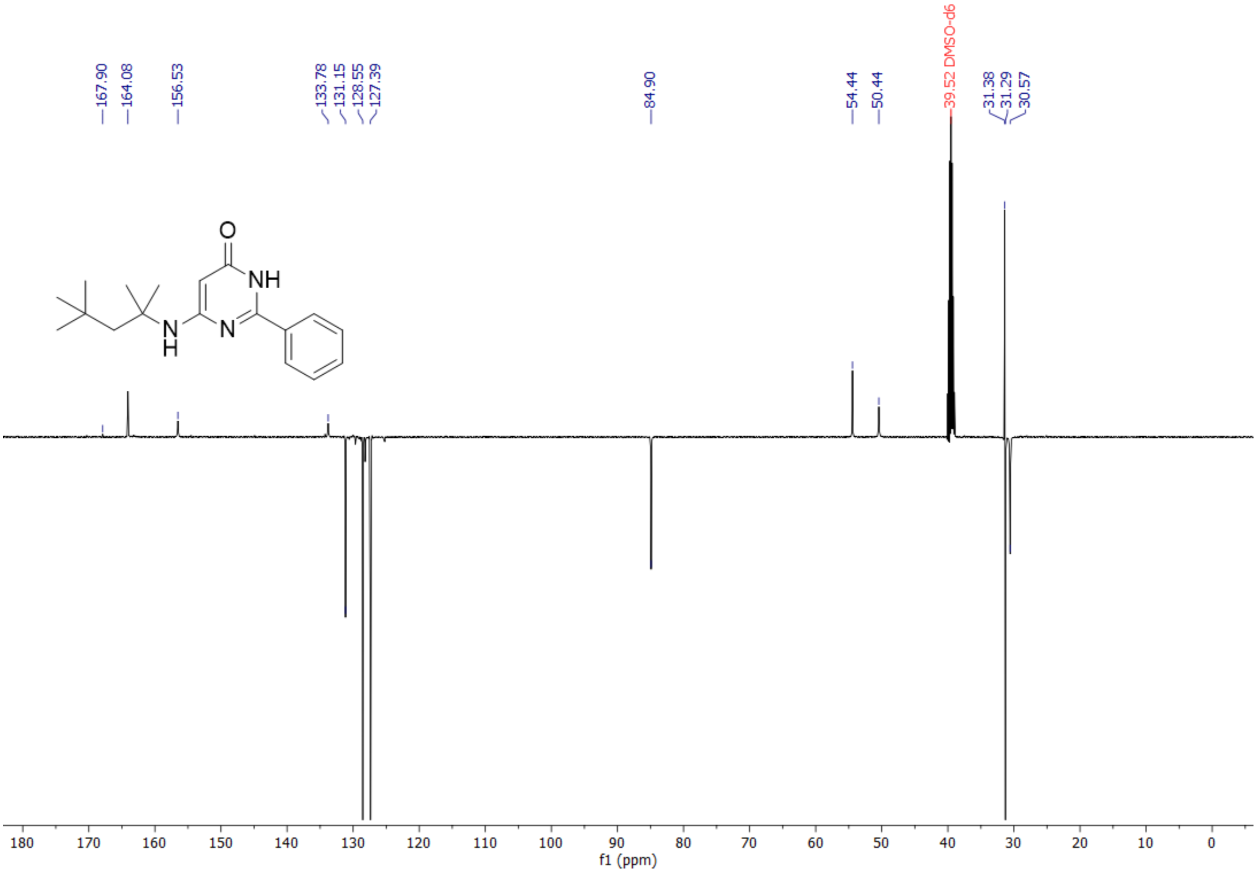

^{1}H-NMR 500 MHz, DMSO-*d*6 @80 °C, **4ca**

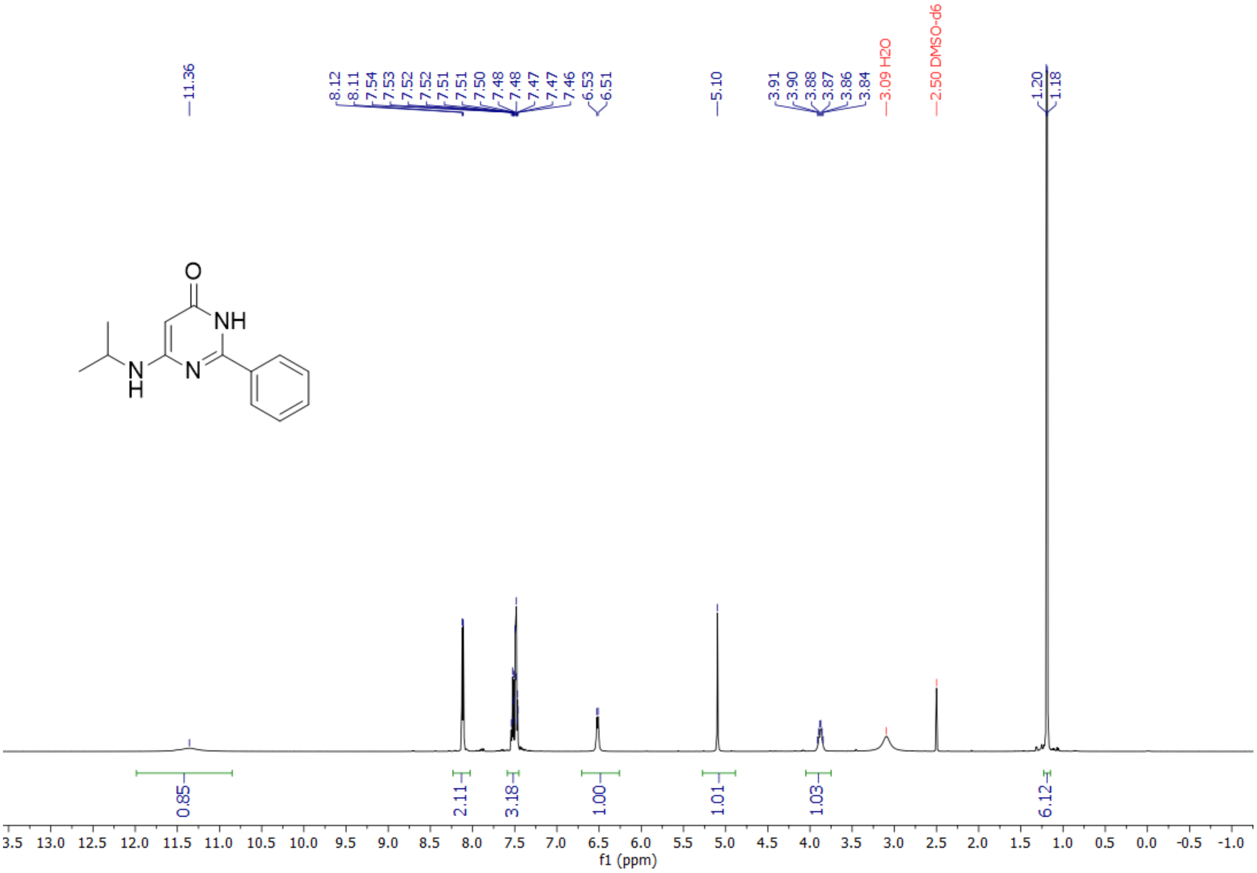

^{13}C-NMR 125.7 MHz, DMSO-*d*6 @80 °C, **4ca**

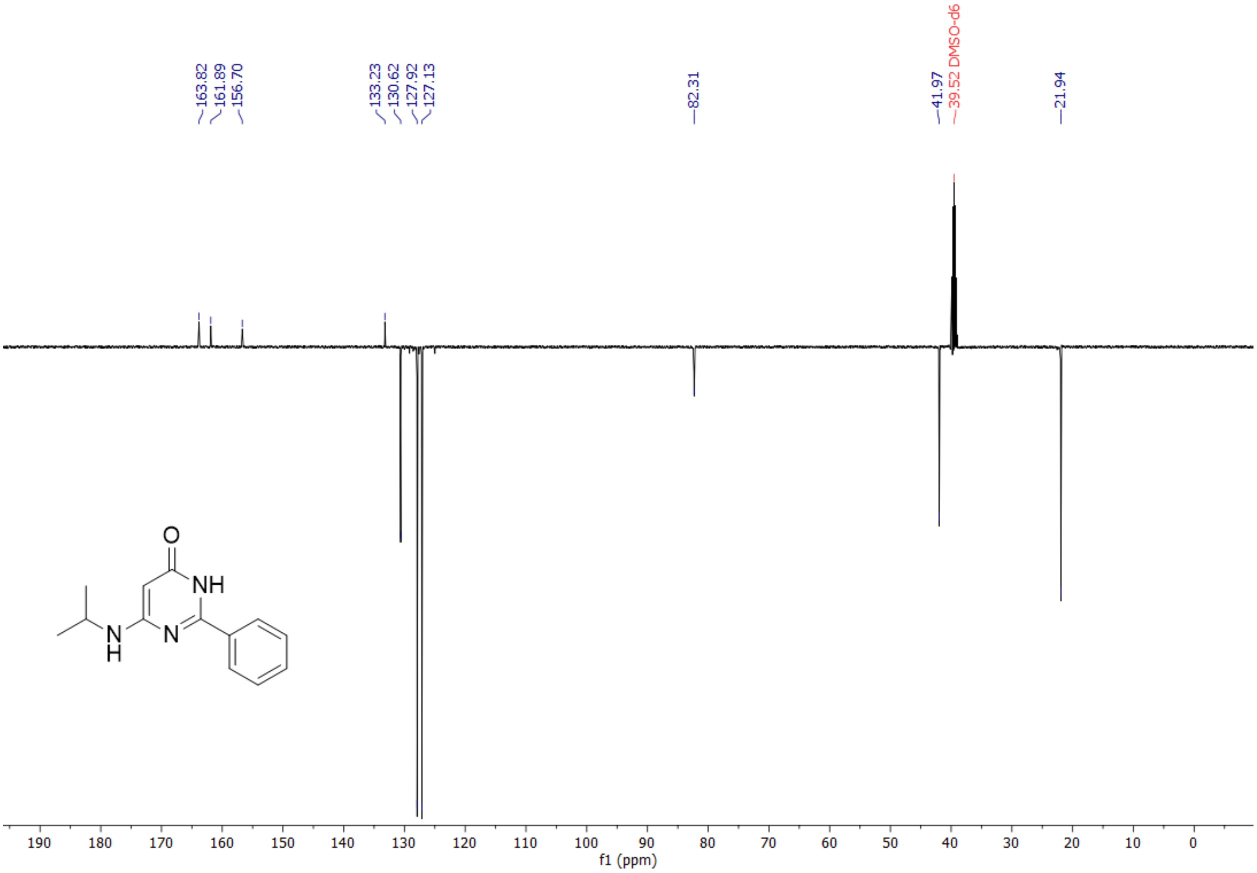

^{1}H-NMR 500 MHz, $CDCl_3$ **4da**

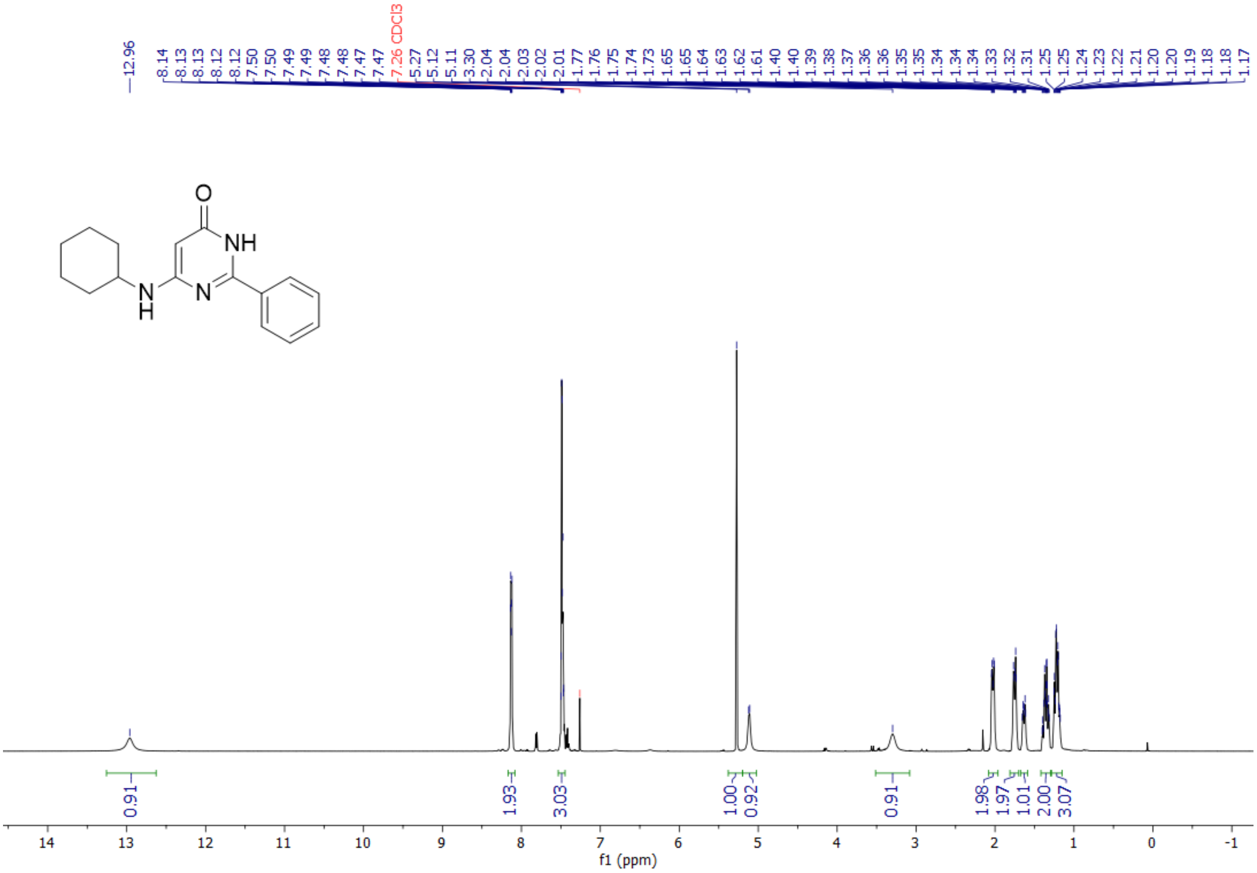

^{13}C-NMR 125.7 MHz, $CDCl_3$ **4da**

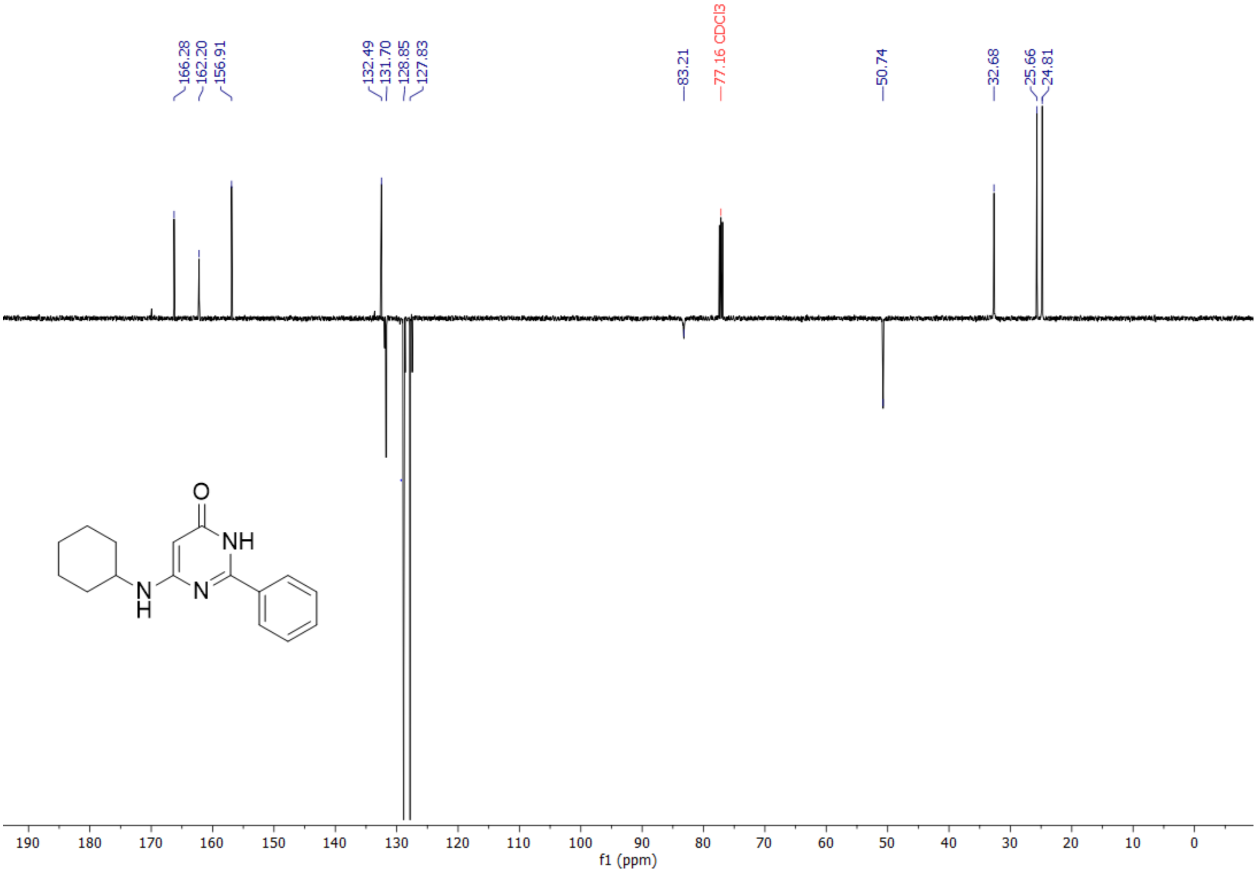

^{1}H-NMR 500 MHz, DMSO-*d*6 **4ea**

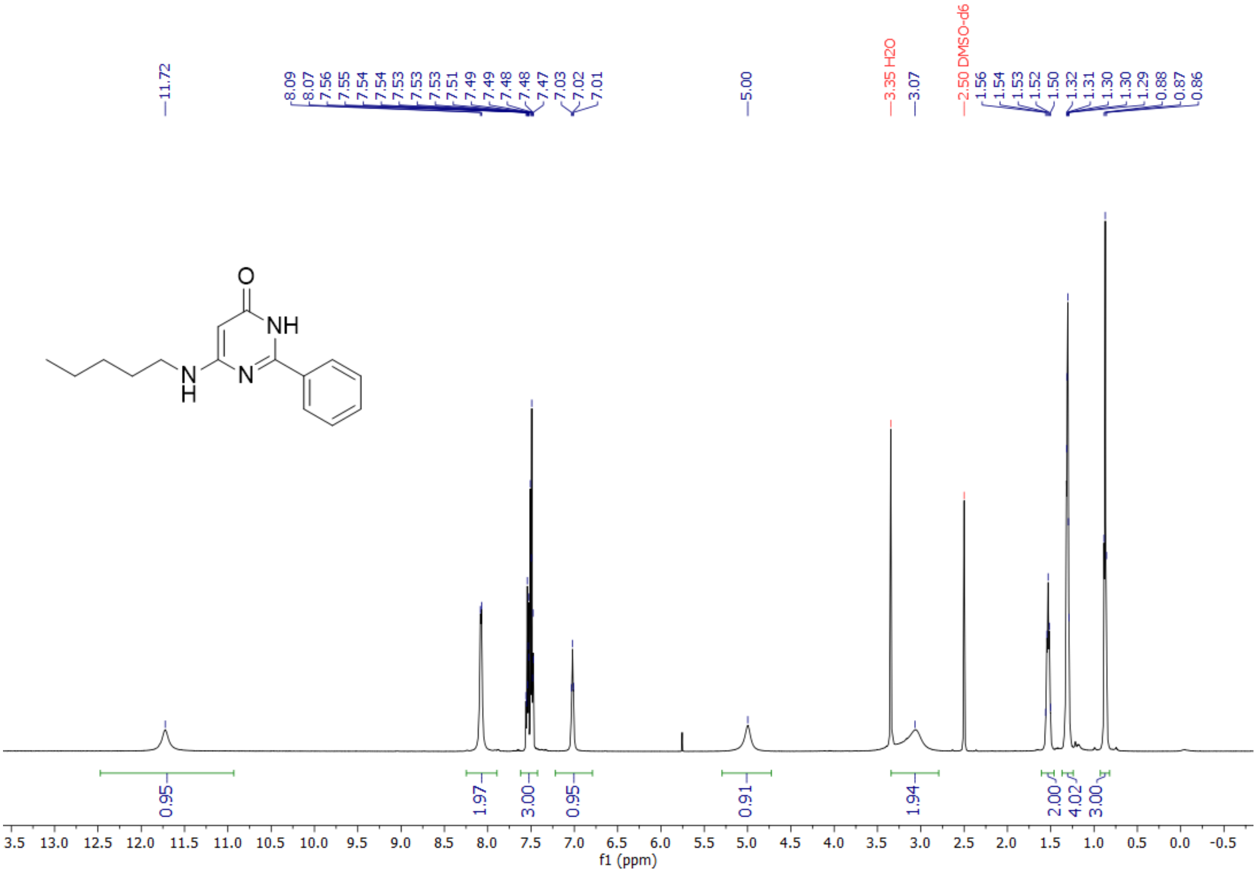

^{13}C-NMR 125.7 MHz, DMSO-*d*6 @80 °C, **4ea**

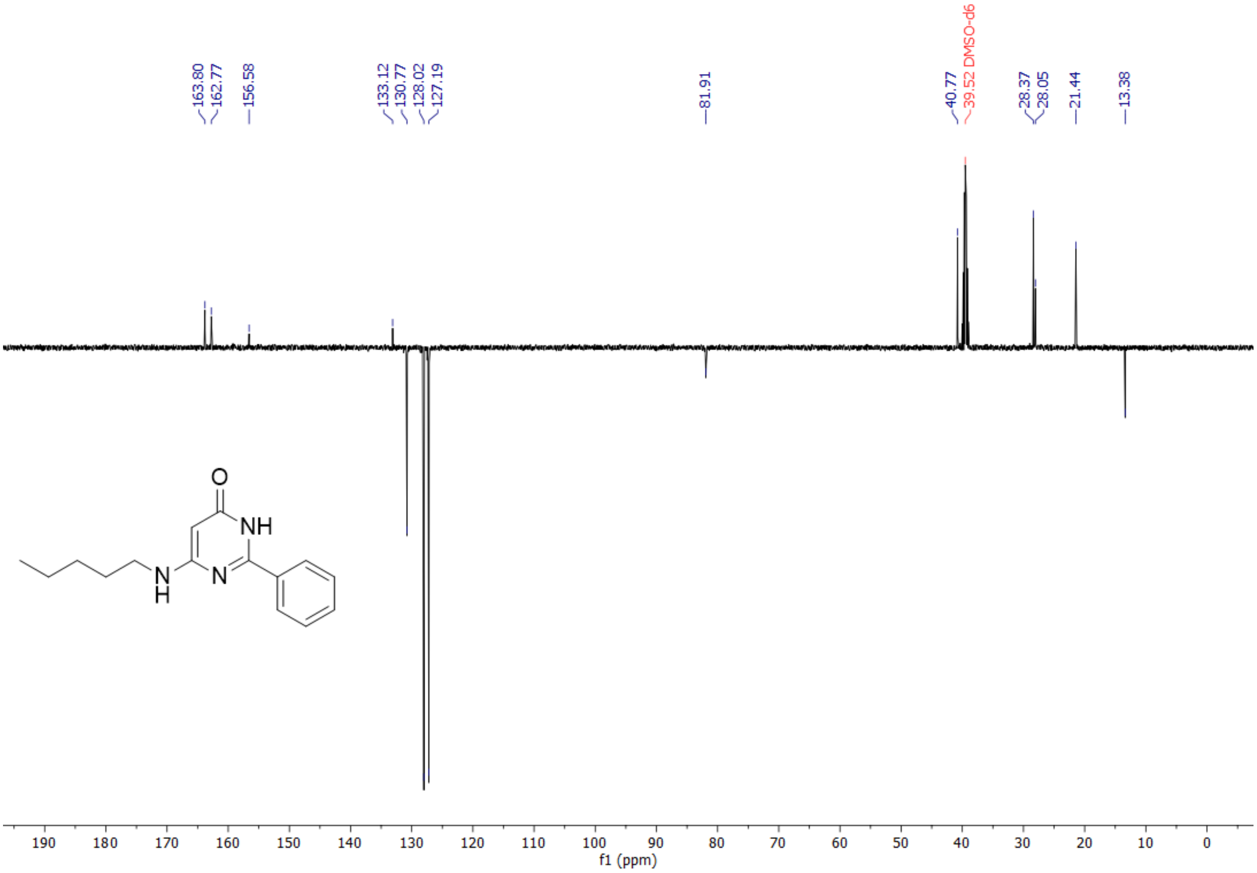

^{1}H-NMR 500 MHz, $CDCl_3$ **4fa**

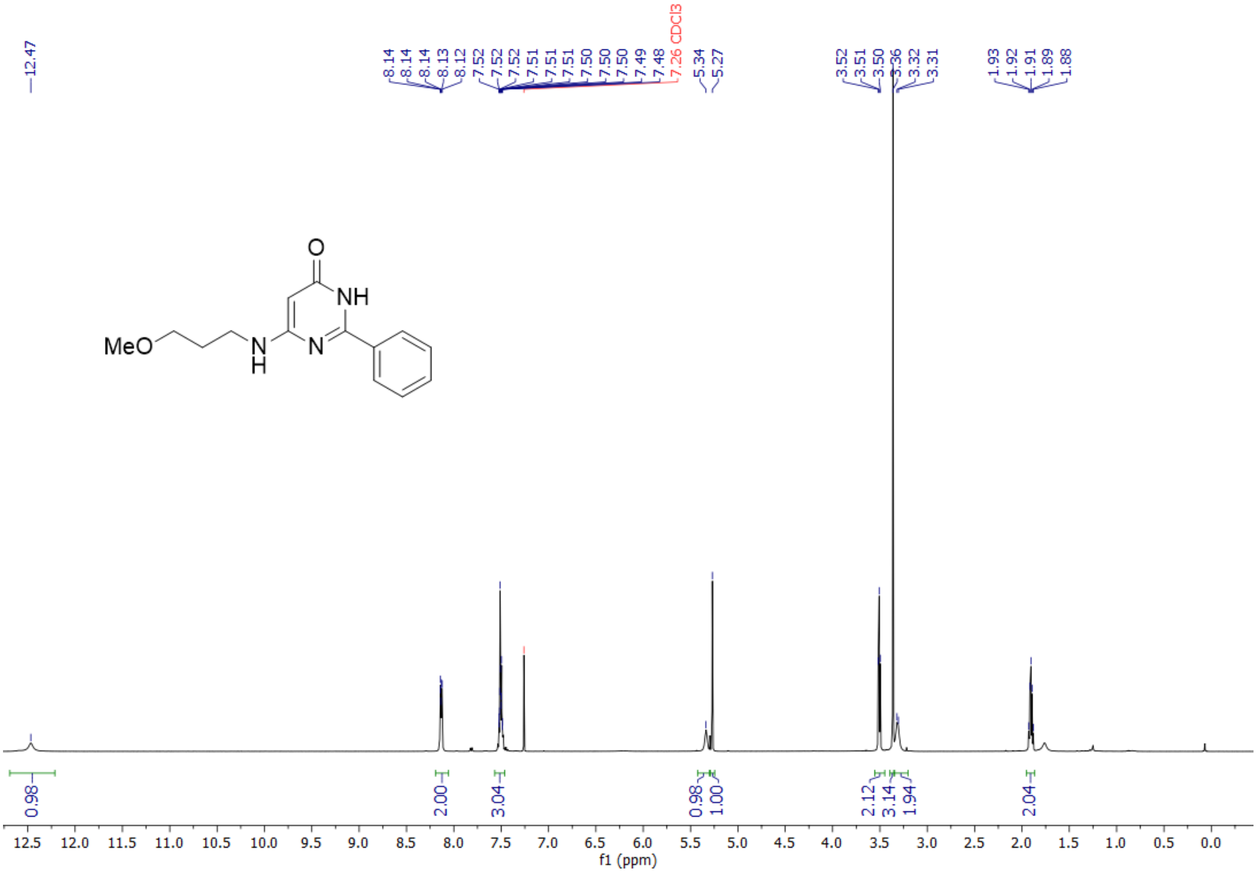

^{13}C-NMR 125.7 MHz, $CDCl_3$ **4fa**

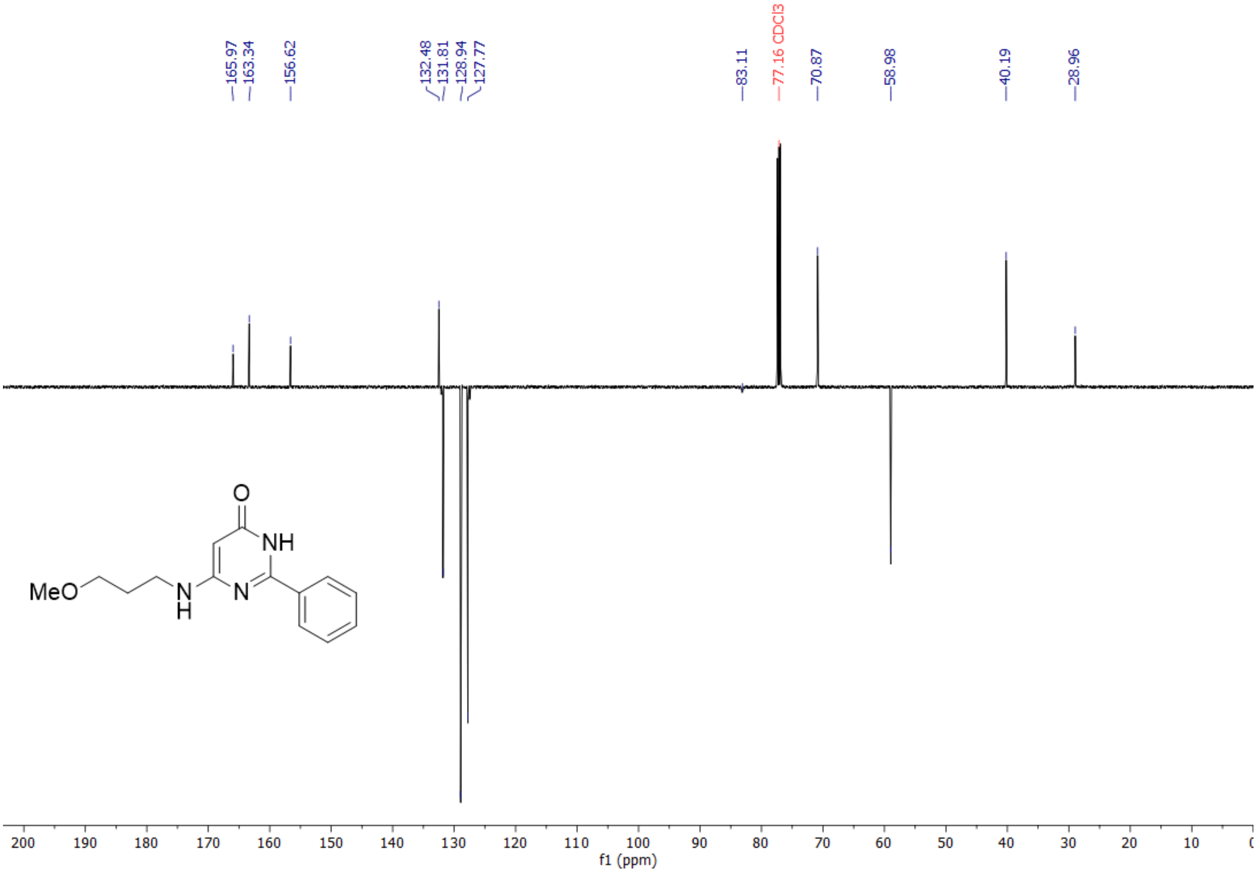

^{1}H-NMR 500 MHz, DMSO-*d*6 @80 °C, **4ga**

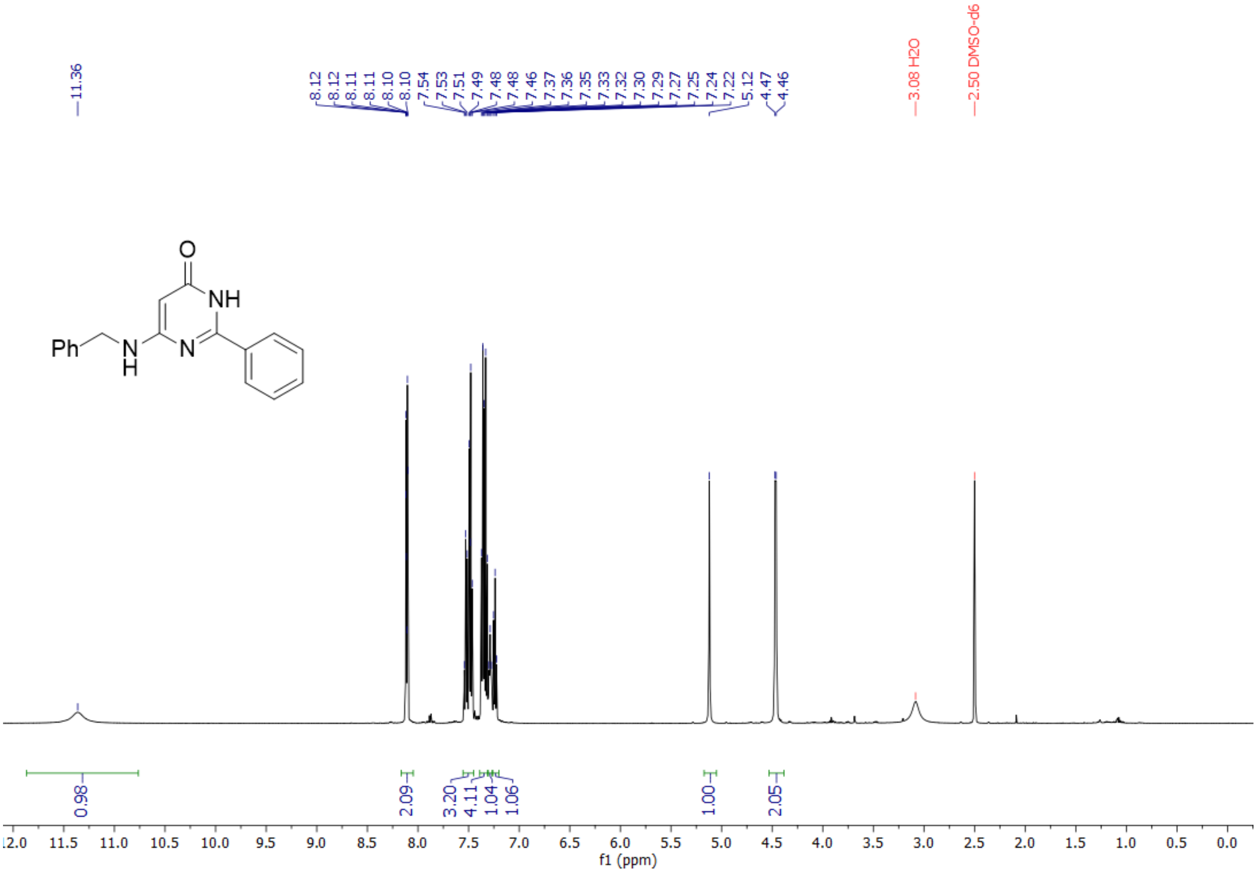

^{13}C-NMR 125.7 MHz, DMSO-*d*6 @80 °C, **4ga**

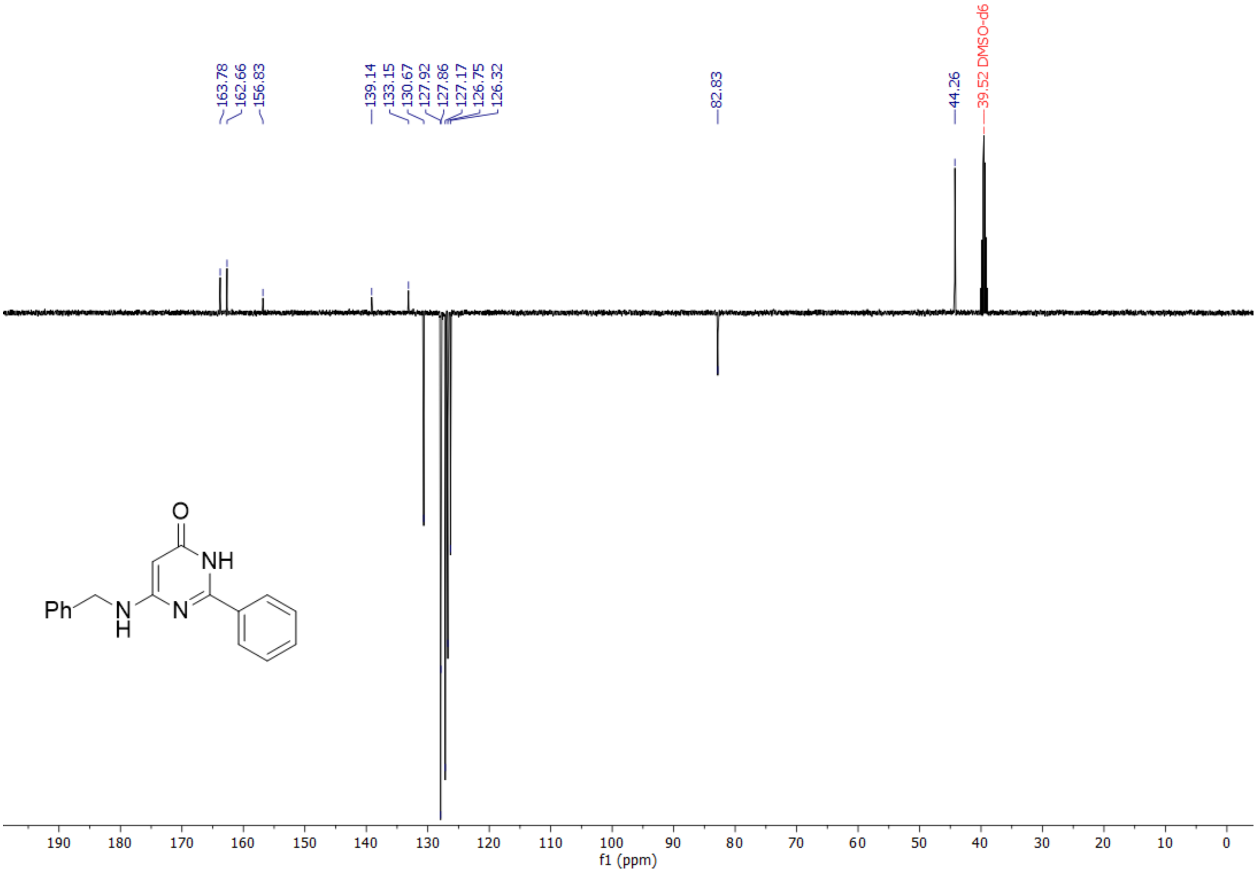

^{1}H-NMR 500 MHz, DMSO-*d*6 @80 °C, **4ha**

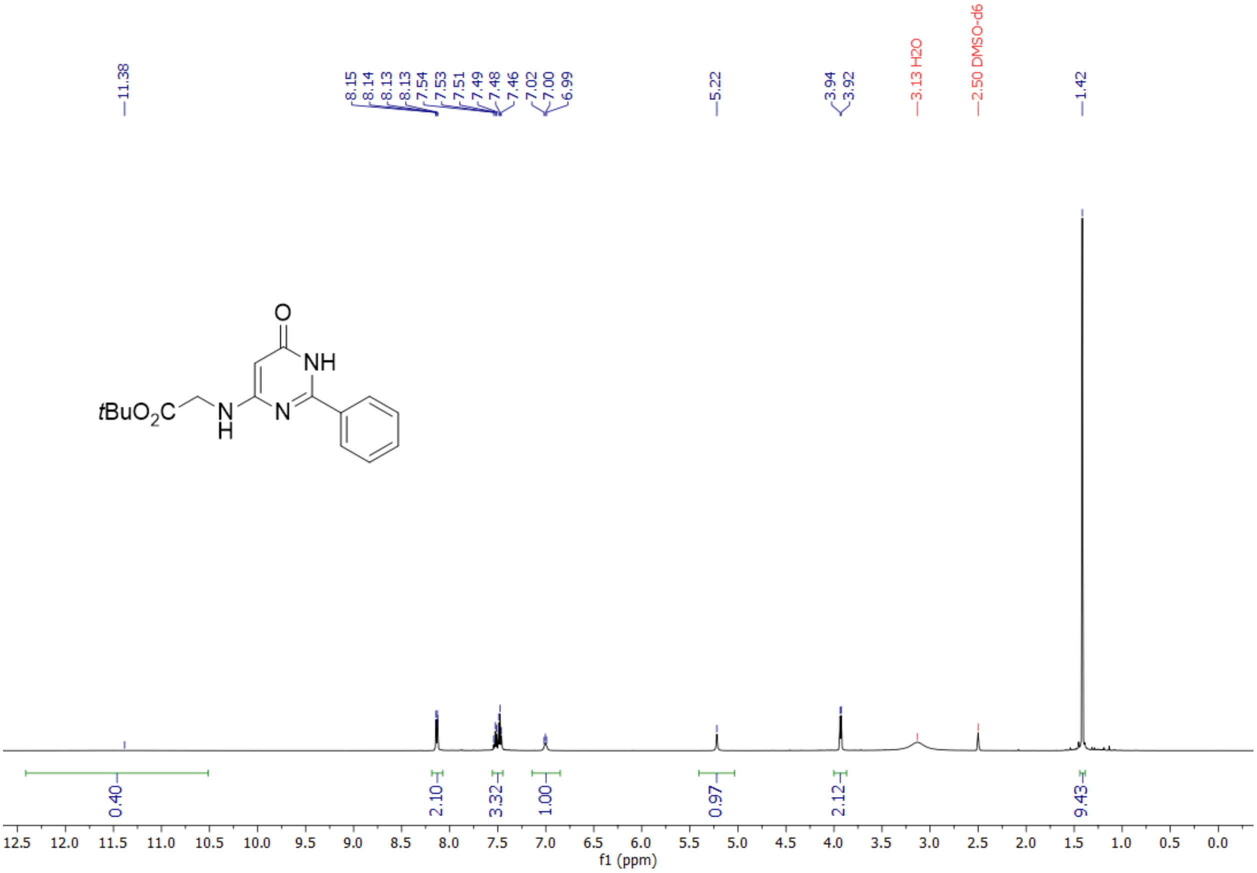

^{13}C-NMR 125.7 MHz, DMSO-*d*6 @80 °C, **4ha**

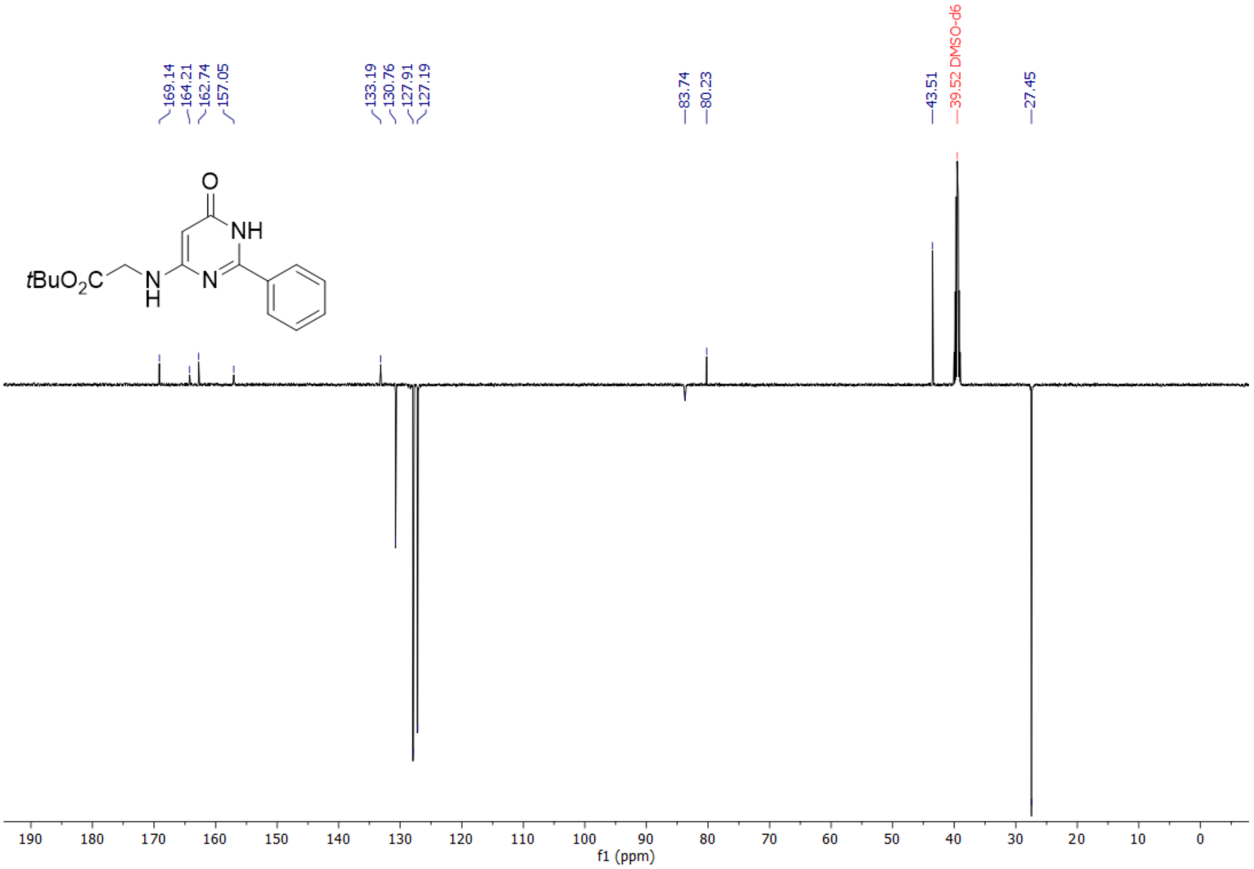

^{1}H-NMR 500 MHz, $CDCl_3$ **4ja**

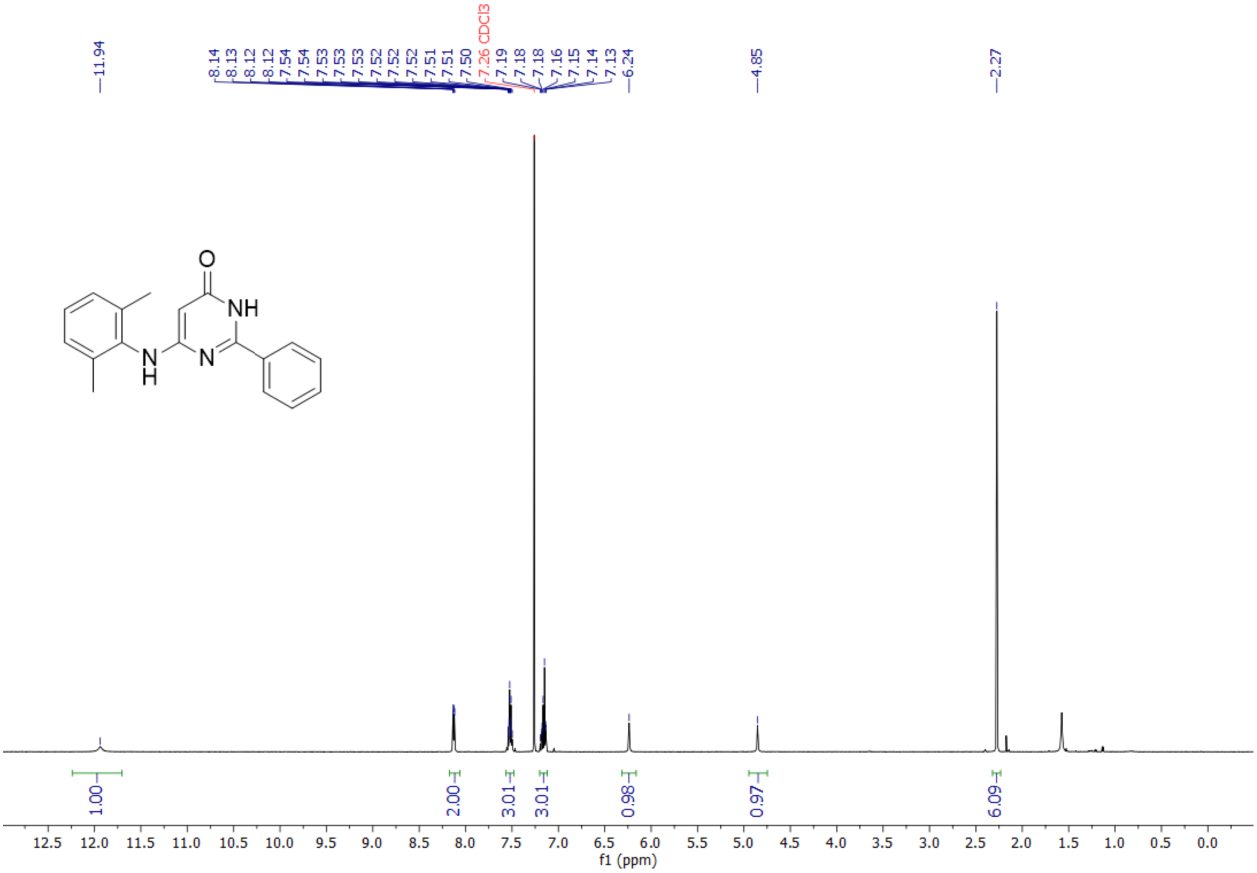

^{13}C-NMR 125.7 MHz, DMSO-*d*6 @80 °C, **4ja**

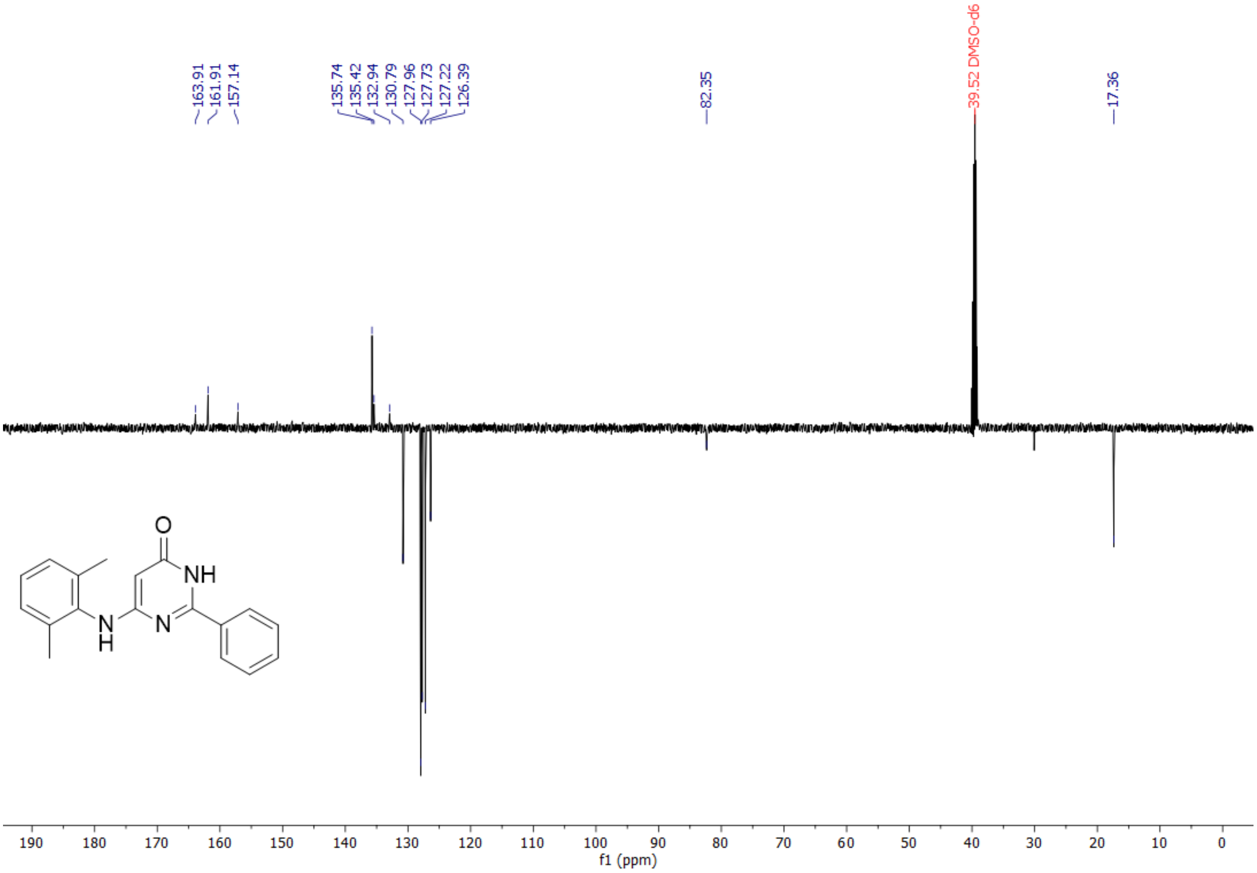

^{1}H-NMR 500 MHz, DMSO-*d*6 **4ka**

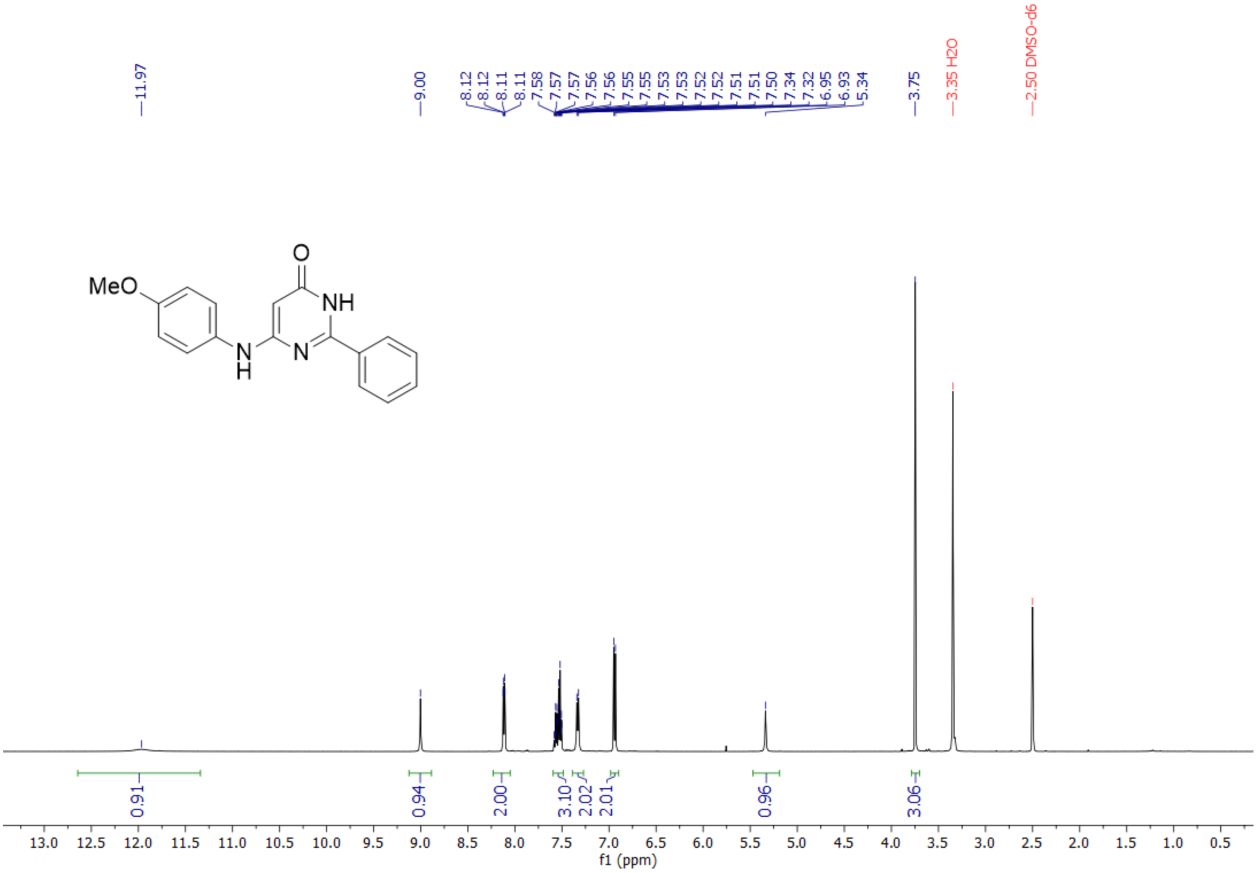

^{13}C-NMR 125.7 MHz, DMSO-*d*6 **4ka**

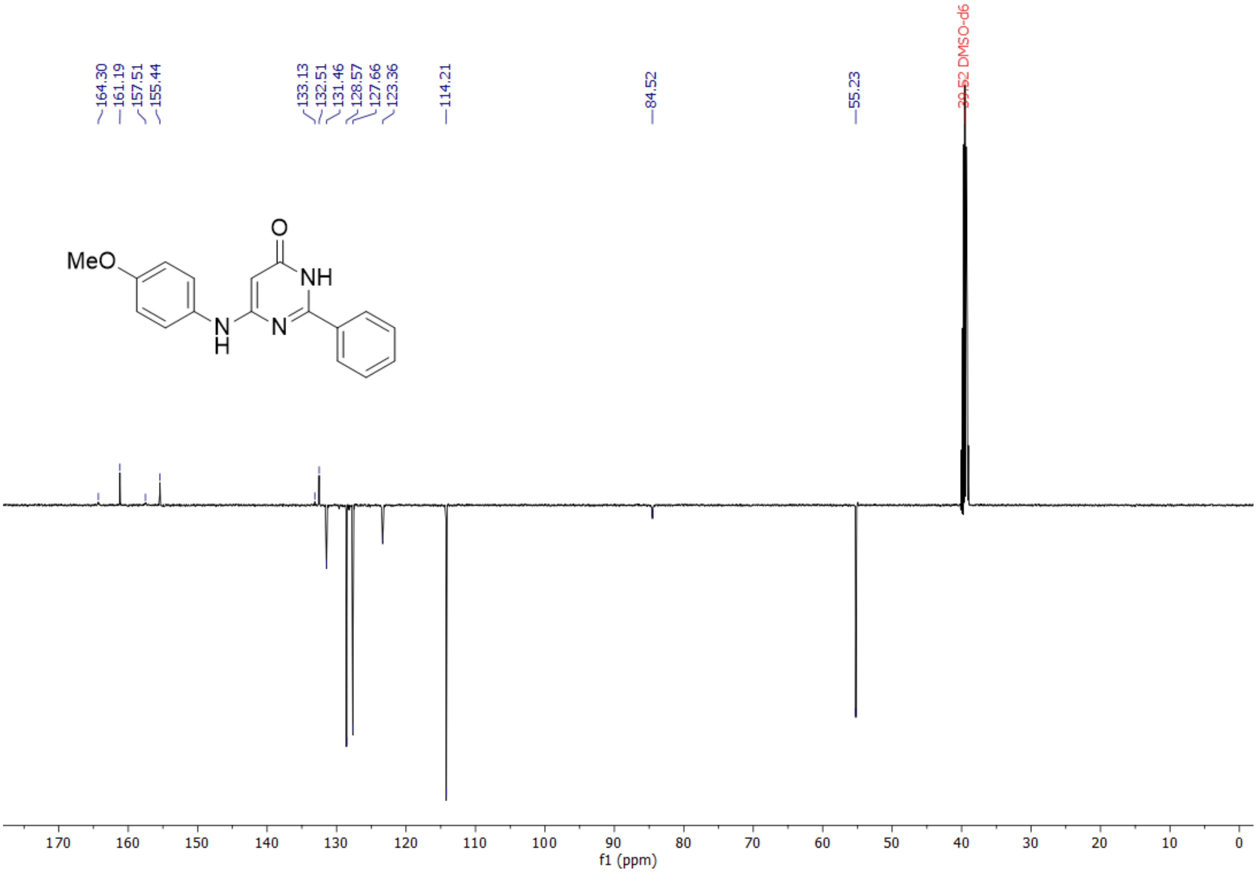

^{1}H-NMR 500 MHz, $CDCl_3$ **4ab**

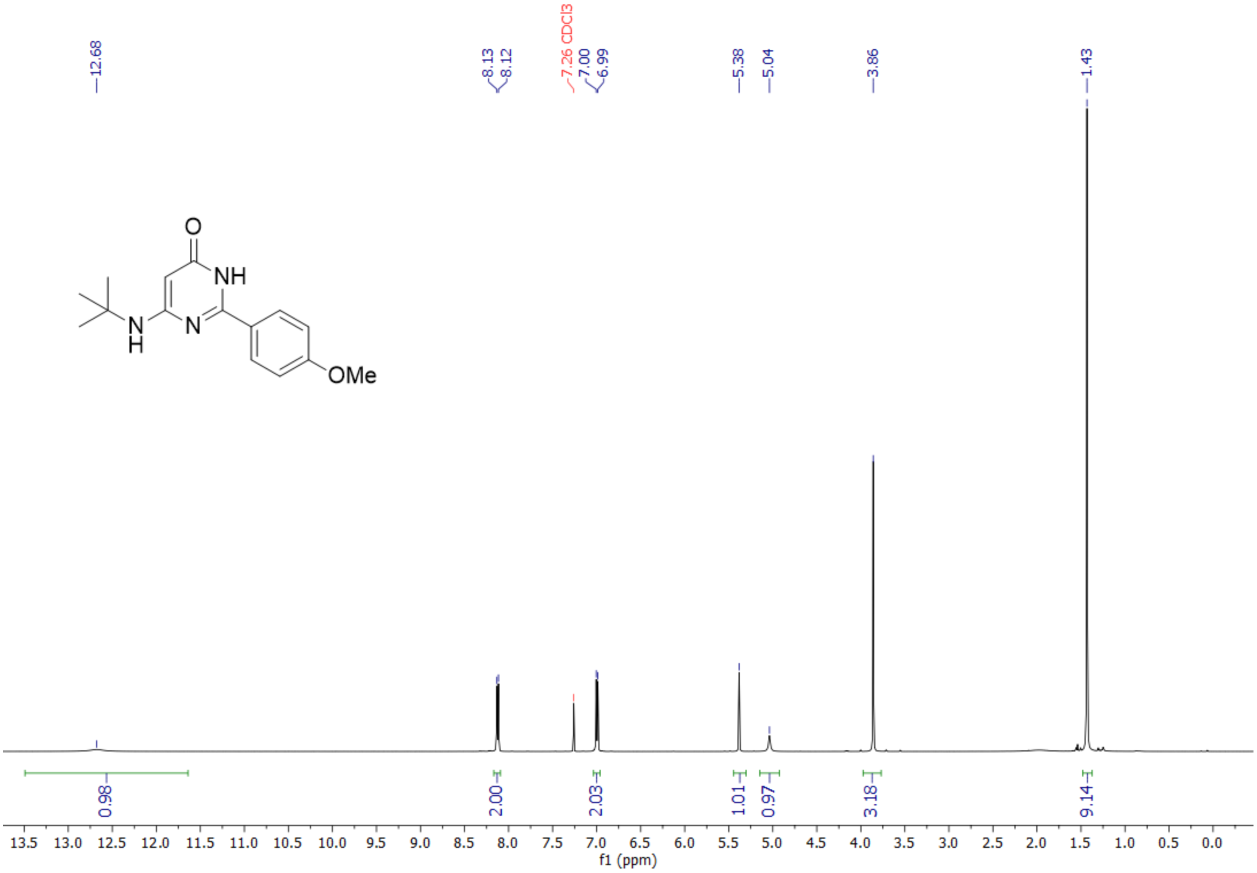

^{13}C-NMR 125.7 MHz, $CDCl_3$ **4ab**

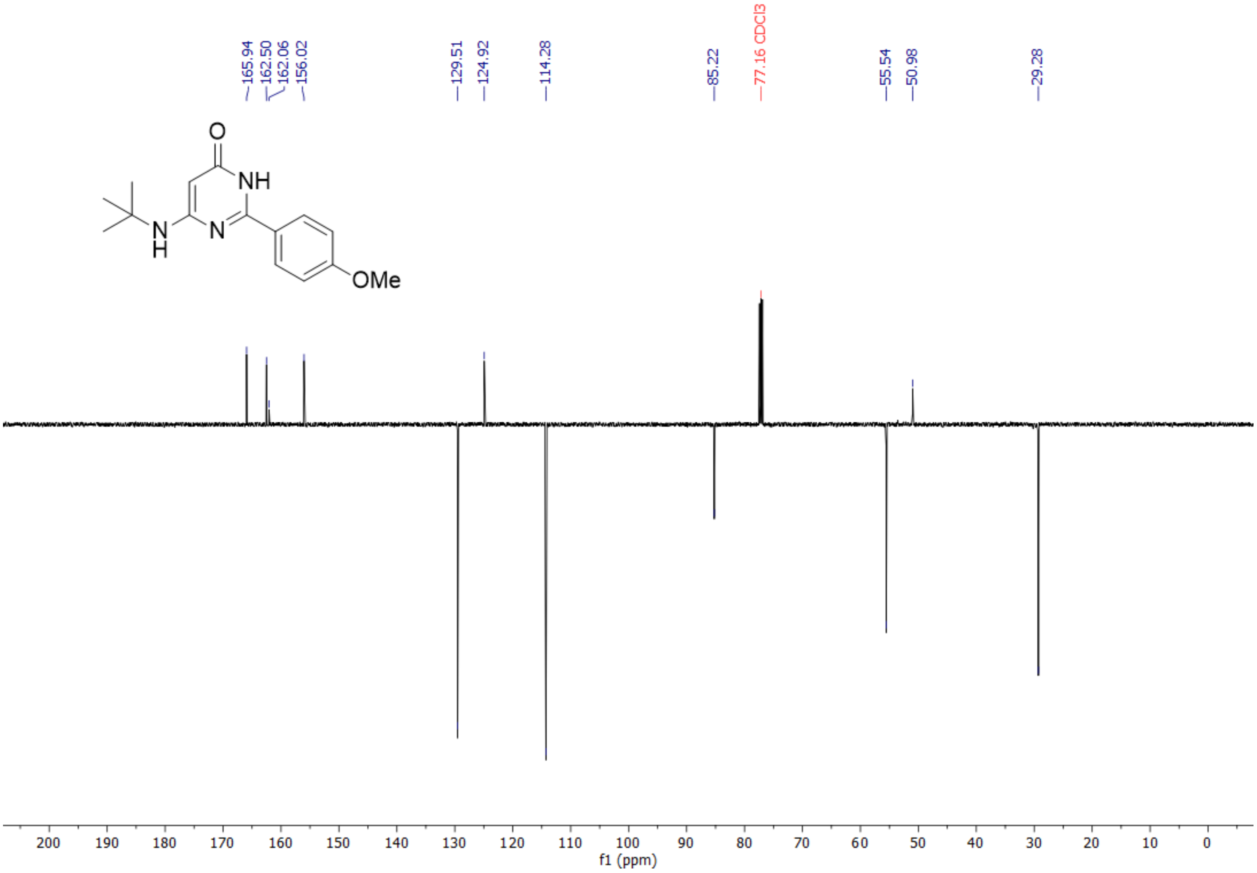

^{1}H-NMR 500 MHz, $CDCl_3$ **4ac**

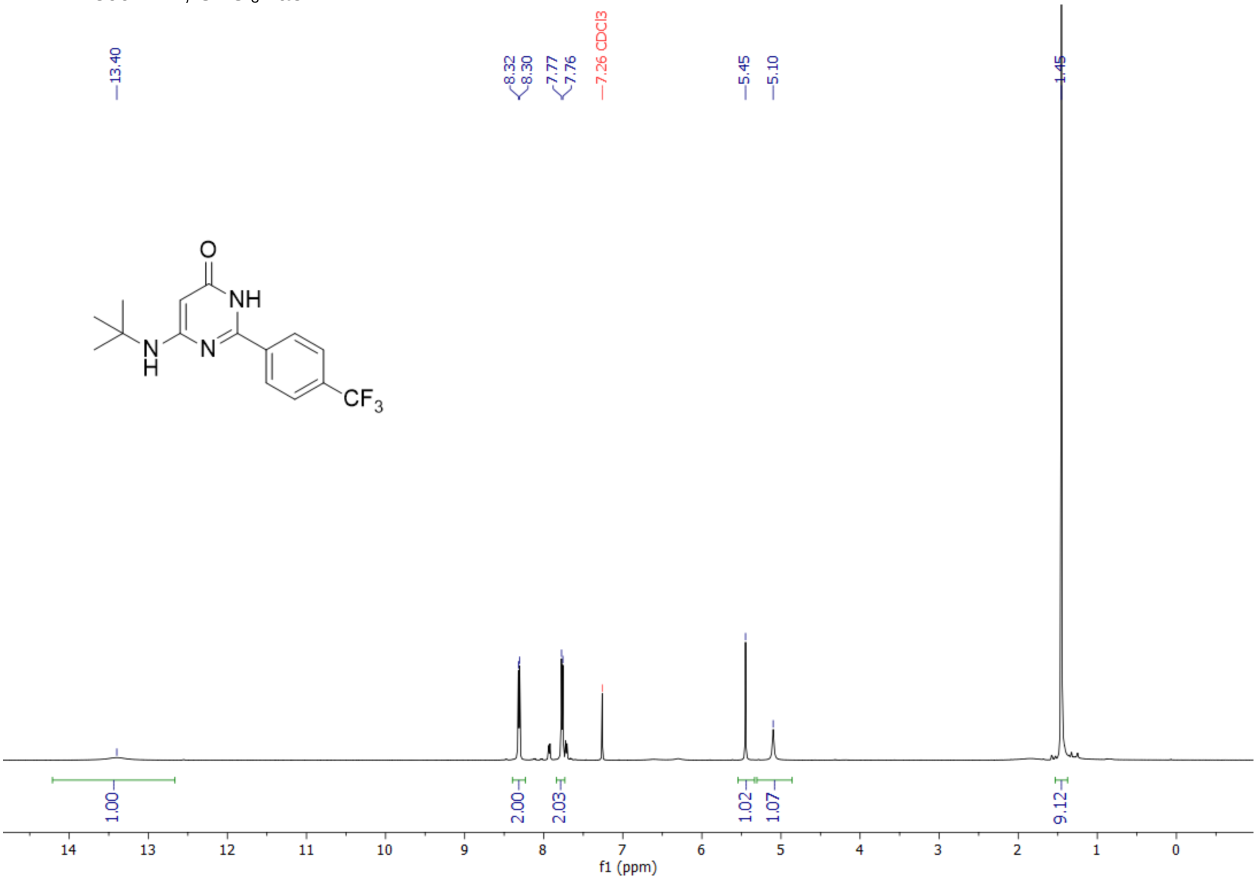

^{13}C-NMR 125.7 MHz, $CDCl_3$ **4ac**

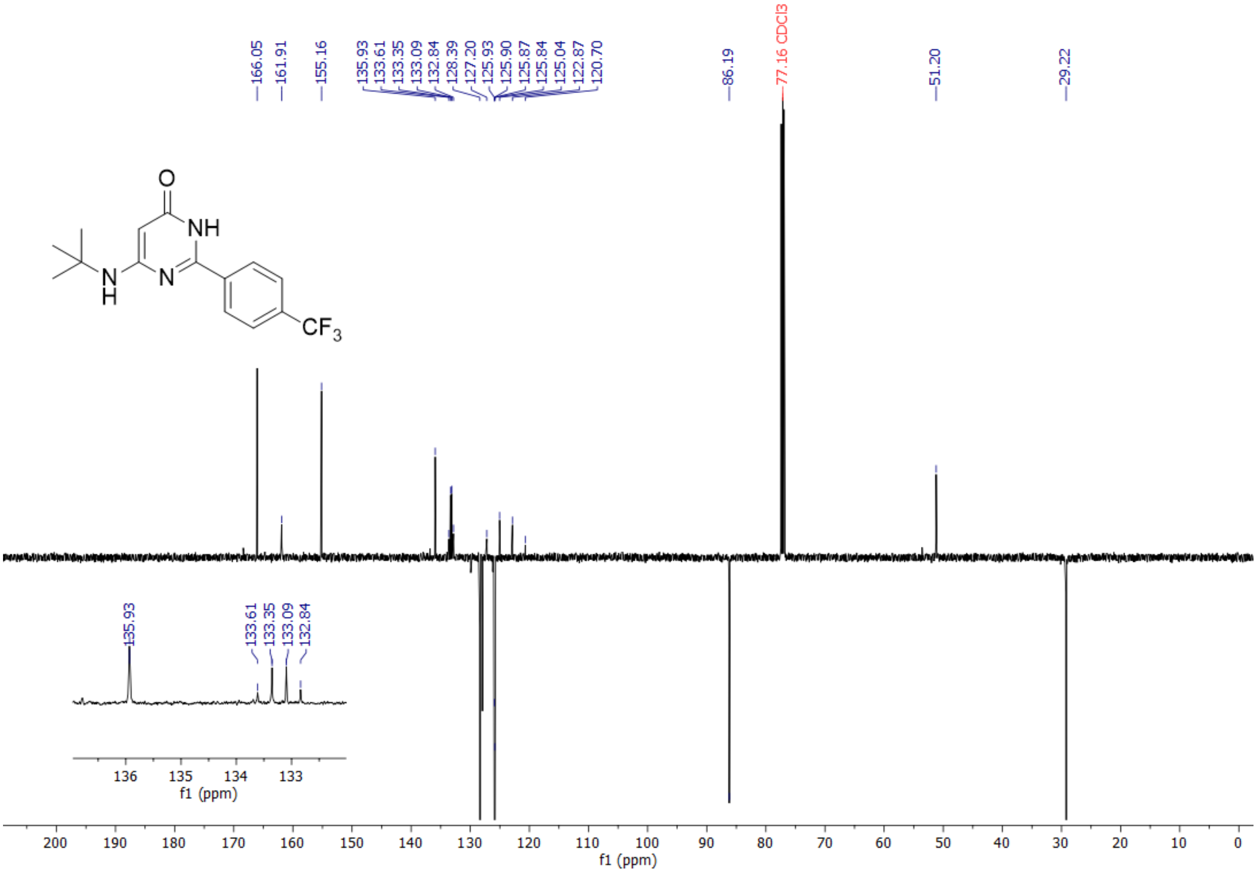

^{19}F-NMR 470.4 MHz, $CDCl_3$ **4ac**

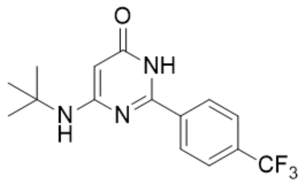

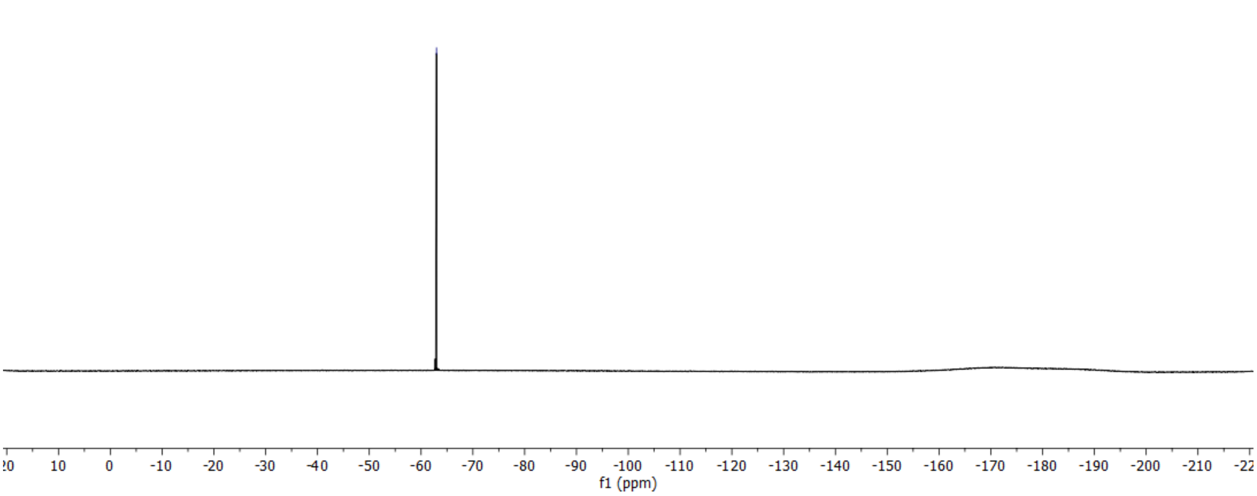

^{1}H-NMR 500 MHz, DMSO-*d*6 **4ad**

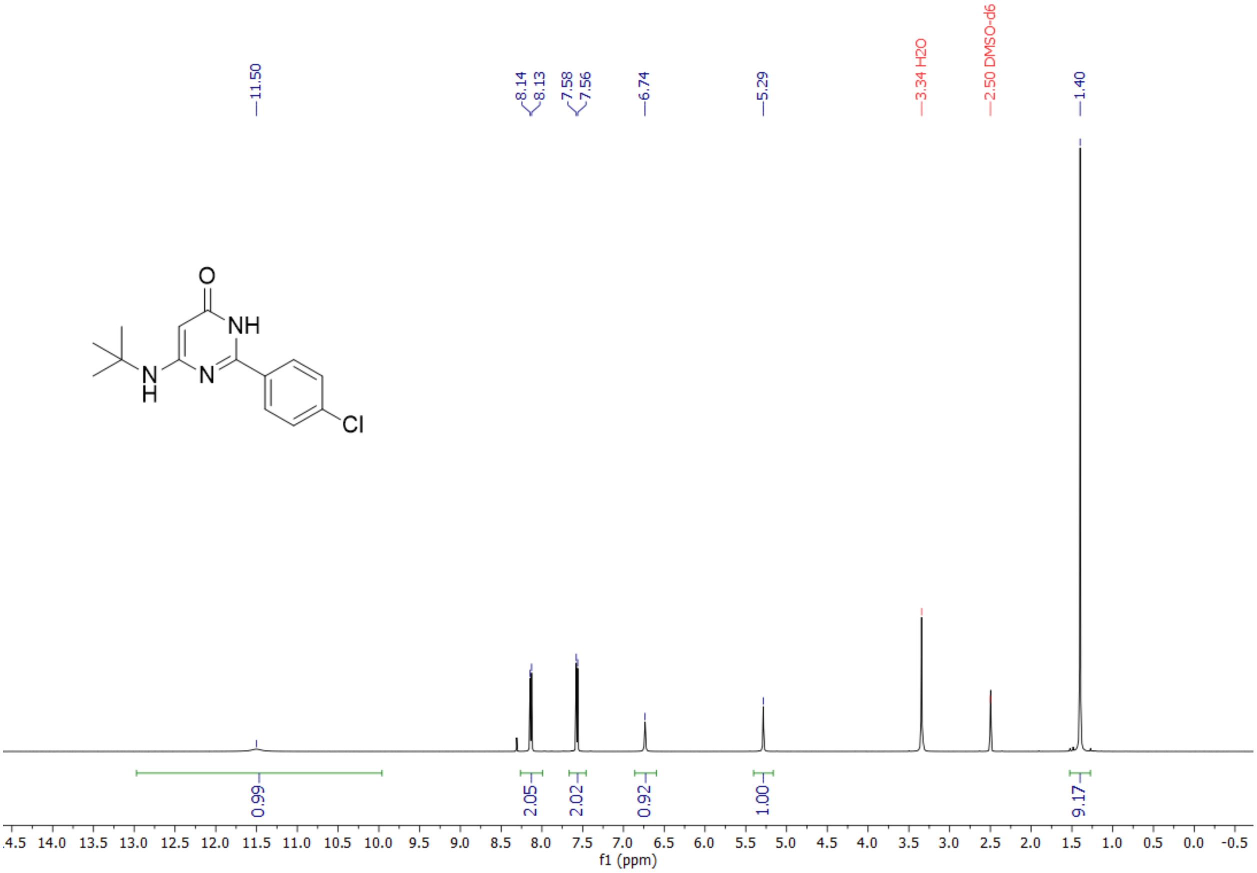

^{13}C-NMR 125.7 MHz, DMSO-*d*6 **4ad**

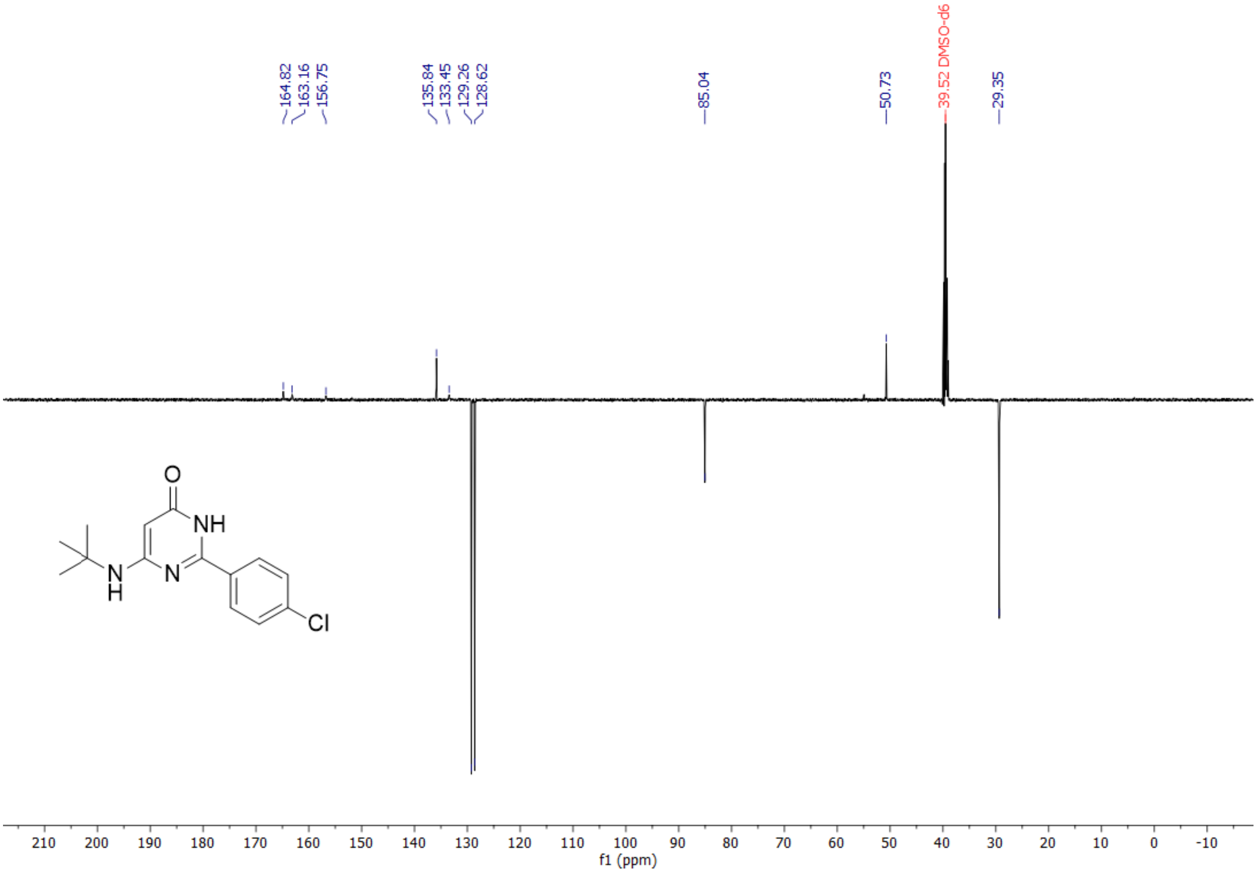

^{1}H-NMR 500 MHz, $CDCl_3$ **4ae**

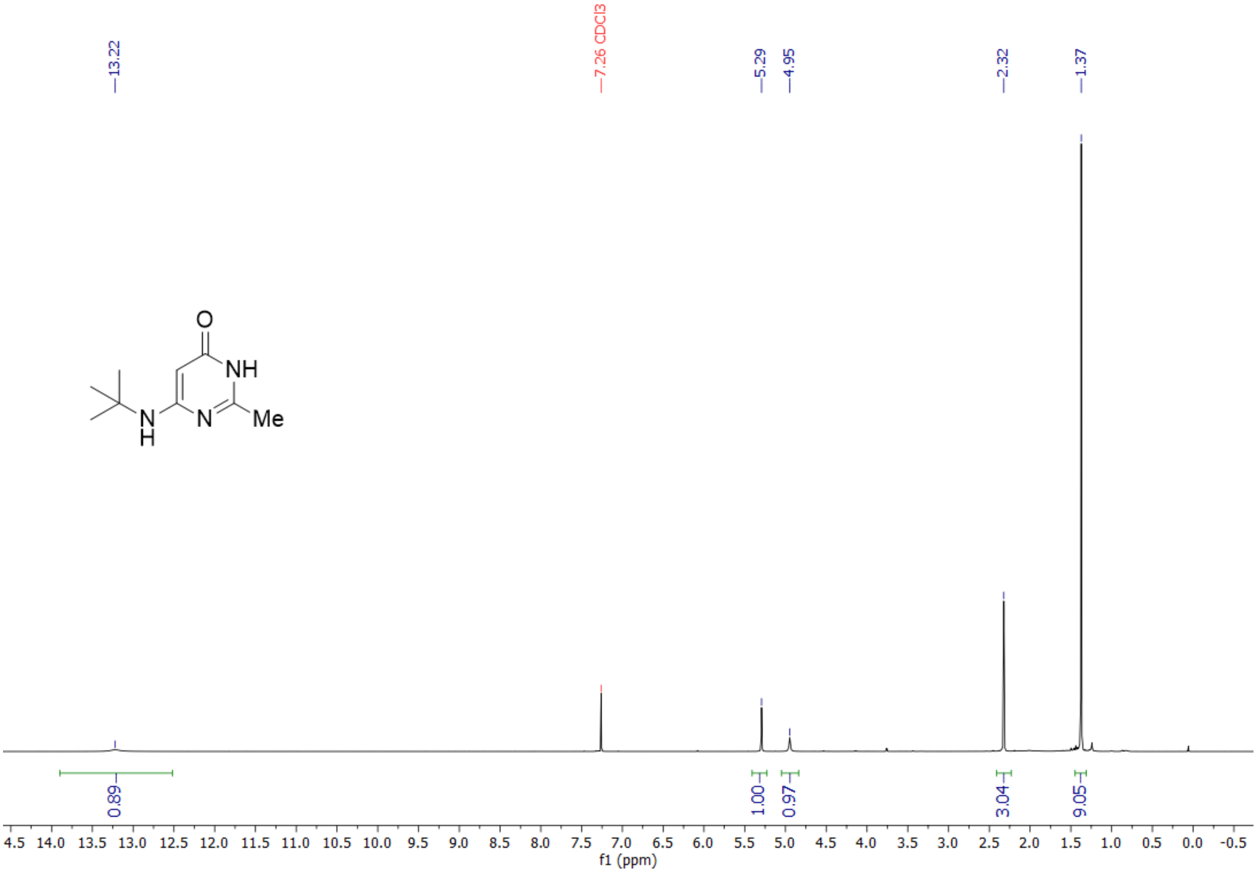

^{13}C-NMR 125.7 MHz, $CDCl_3$ **4ae**

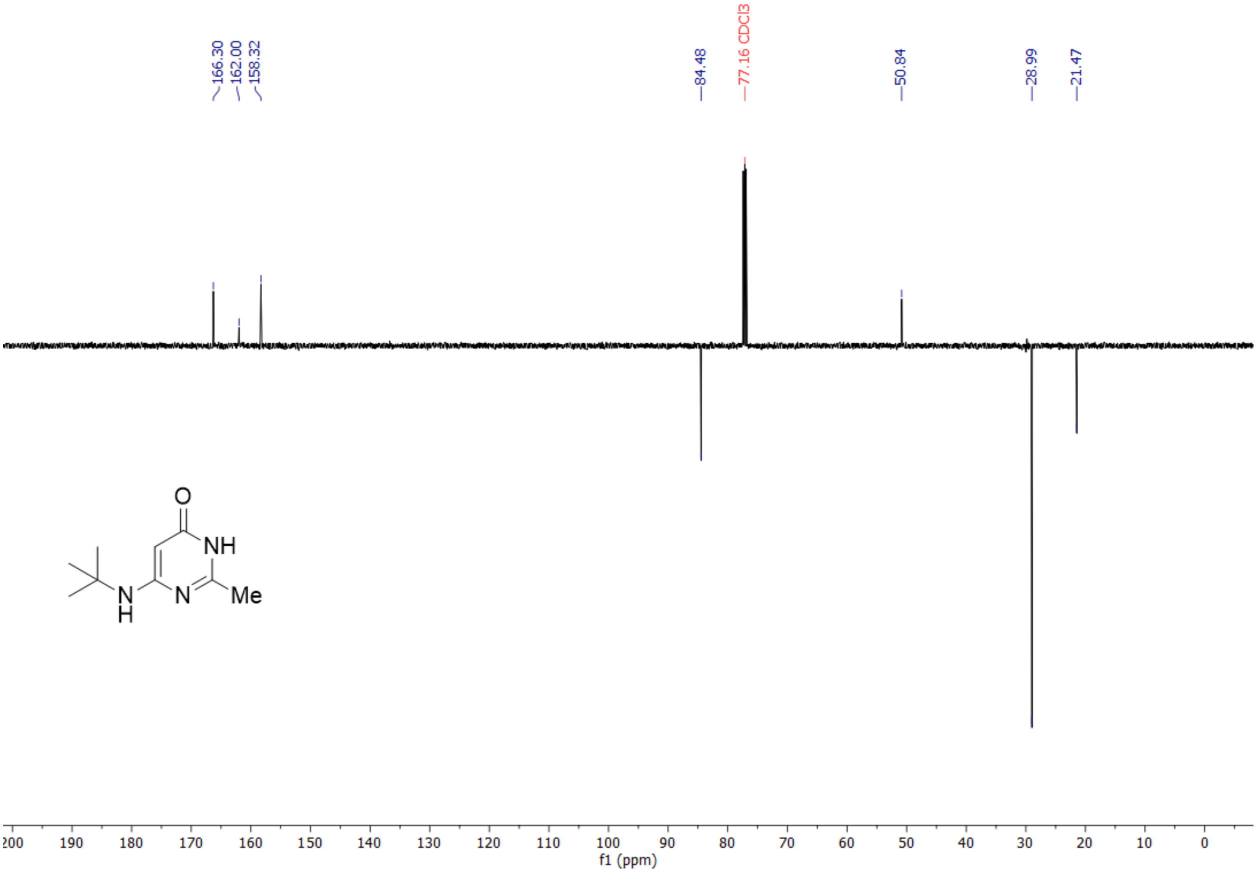

^{1}H-NMR 500 MHz, $CDCl_3$ **4af**

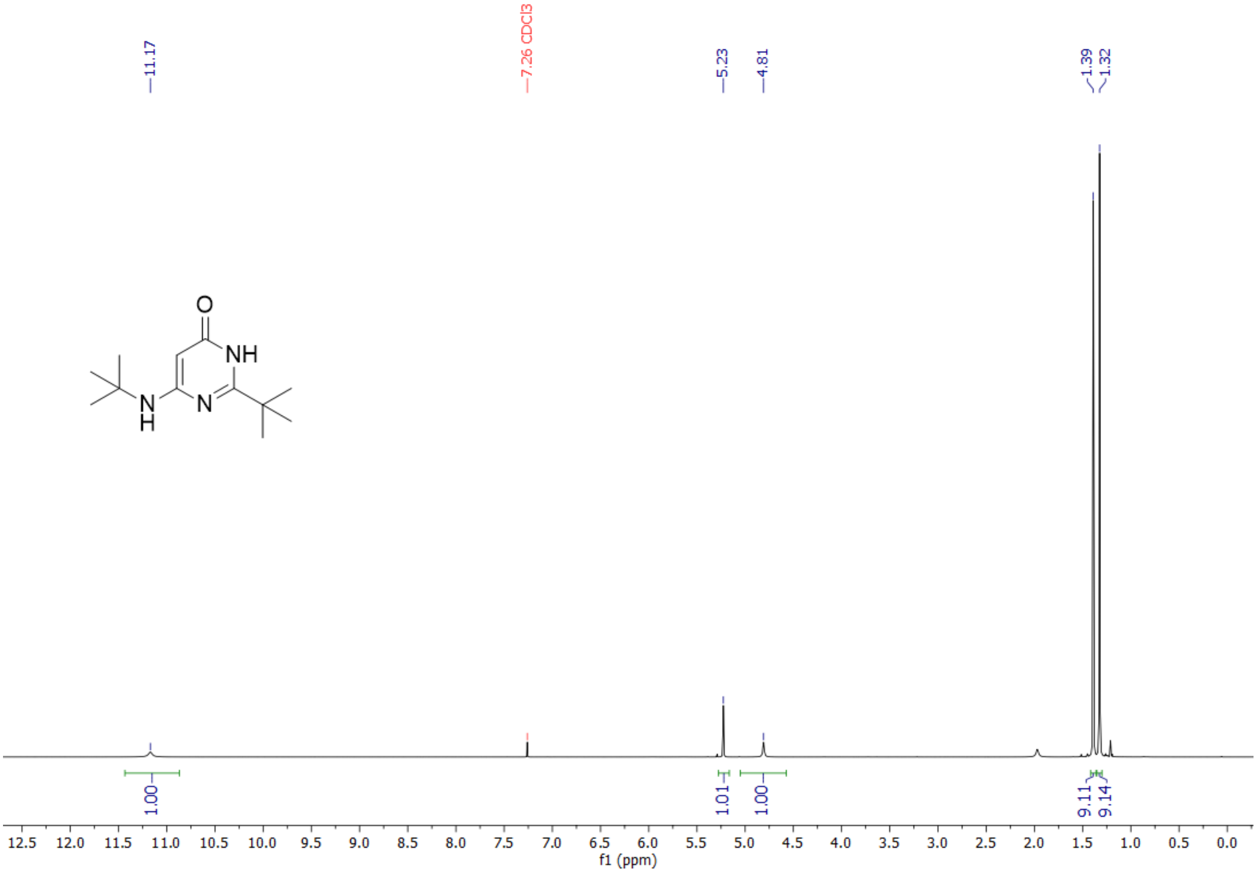

^{13}C-NMR 125.7 MHz, $CDCl_3$ **4af**

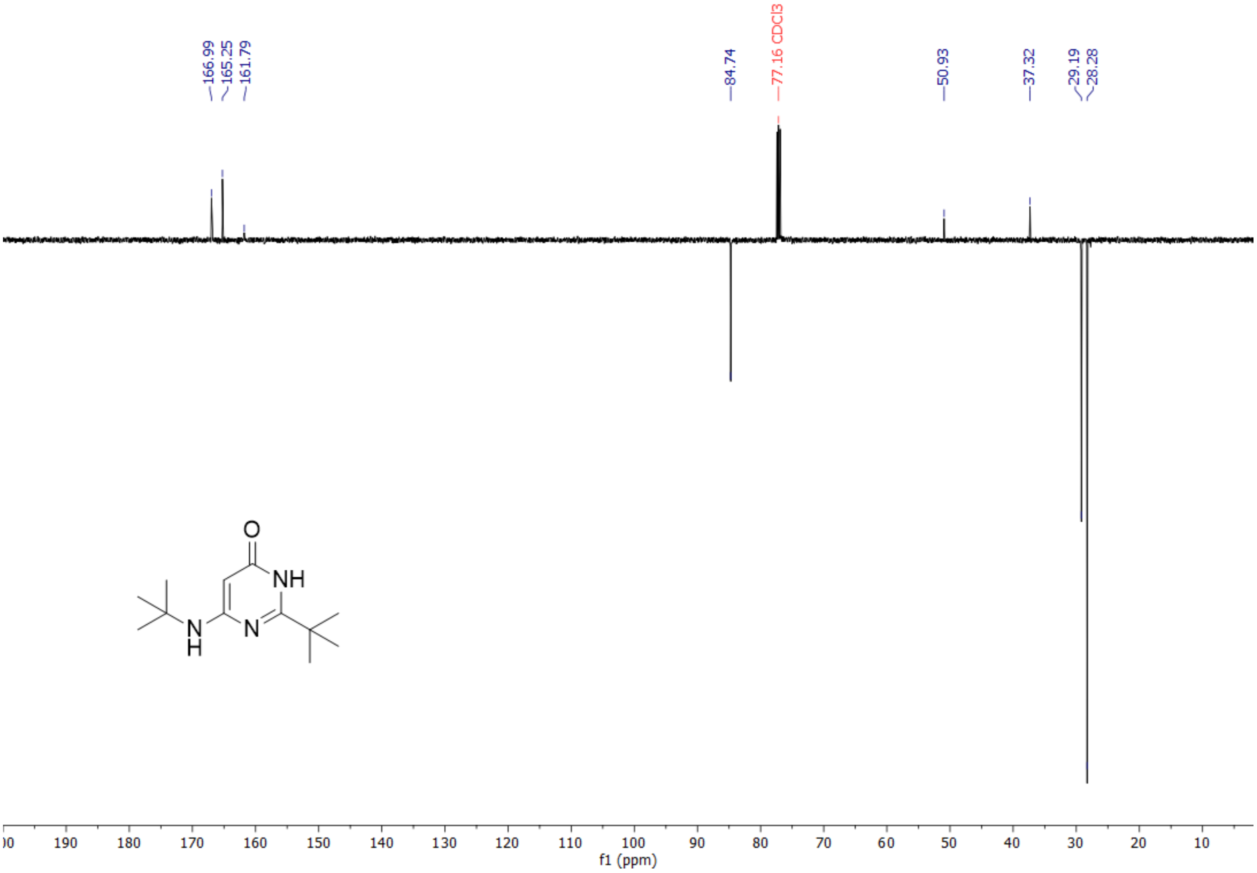

^{1}H-NMR 500 MHz, DMSO-*d*6 **4ag**

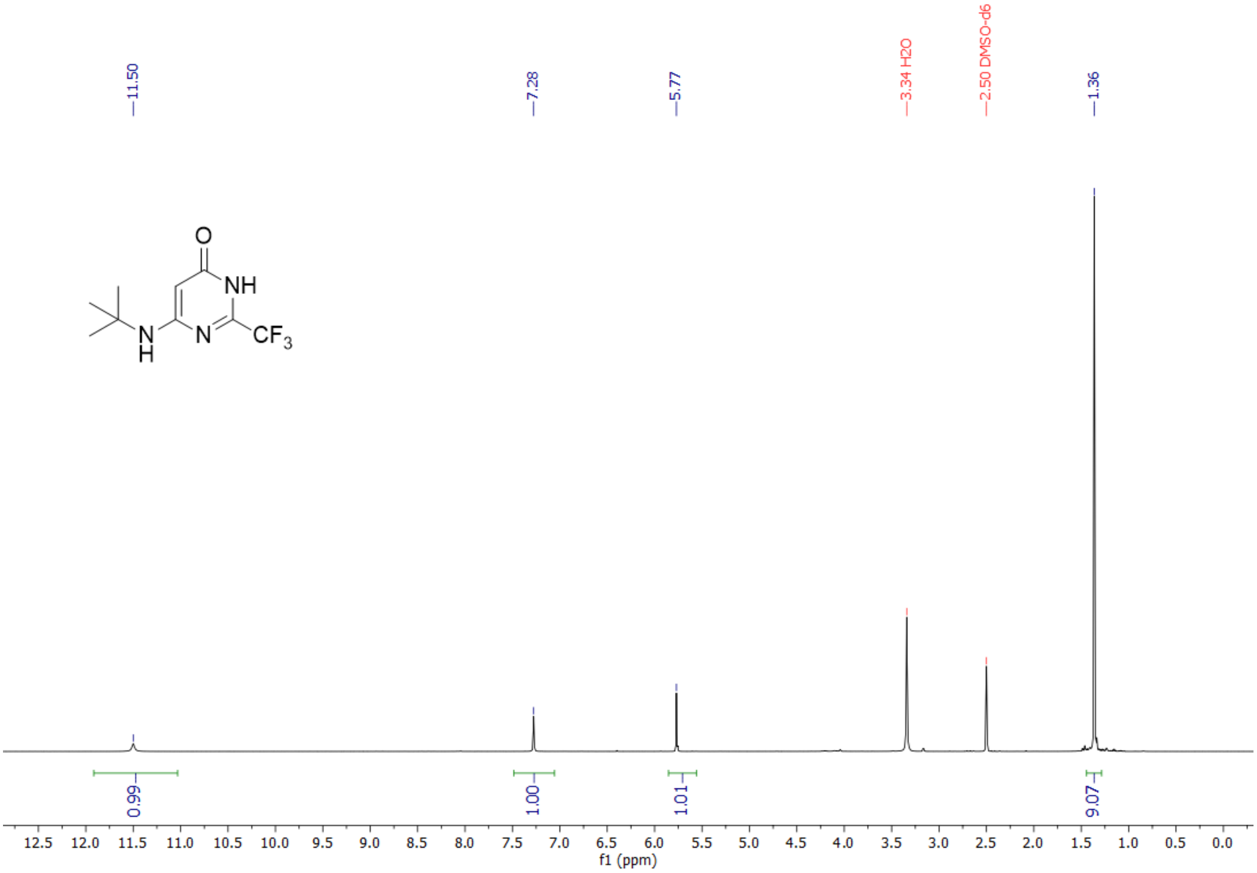

^{13}C-NMR 125.7 MHz, DMSO-*d*6 **4ag**

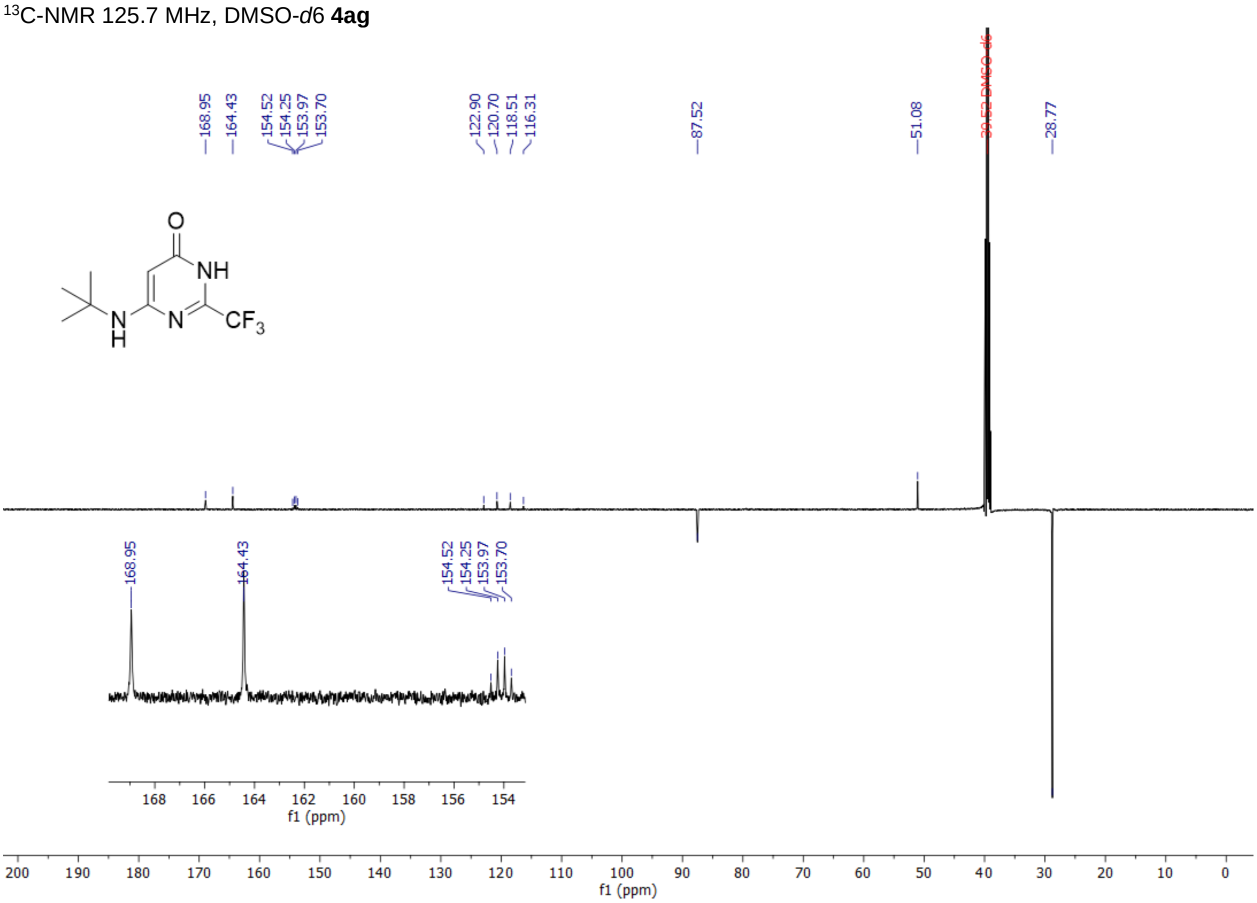

^{19}F-NMR 470.4 MHz, DMSO-*d*6 **4ag**

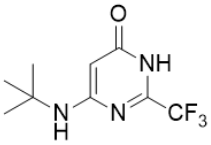

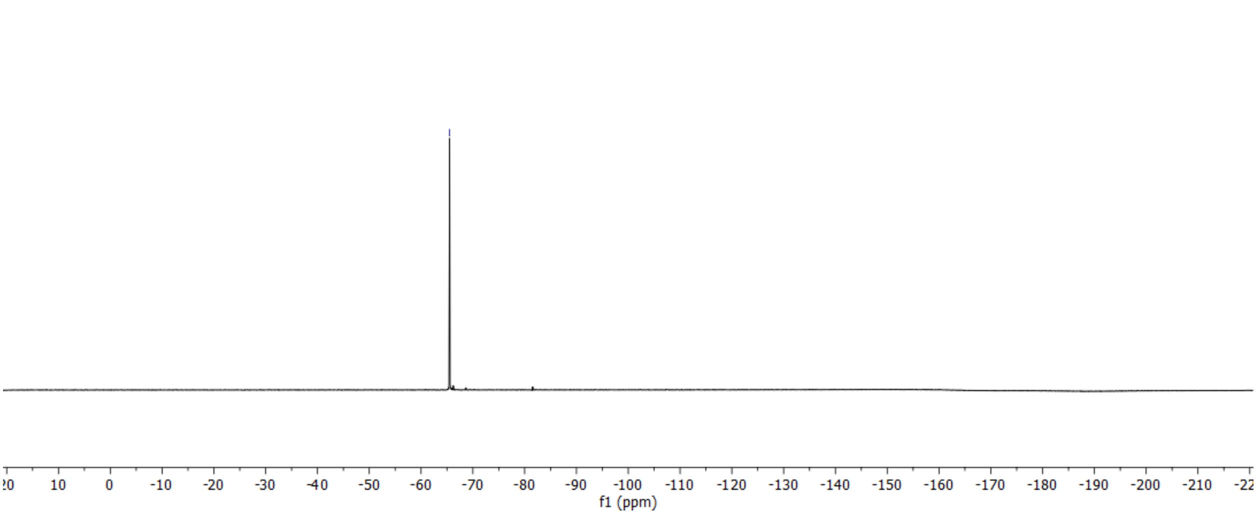

^{1}H-NMR 500 MHz, DMSO-*d*6 **4ah**

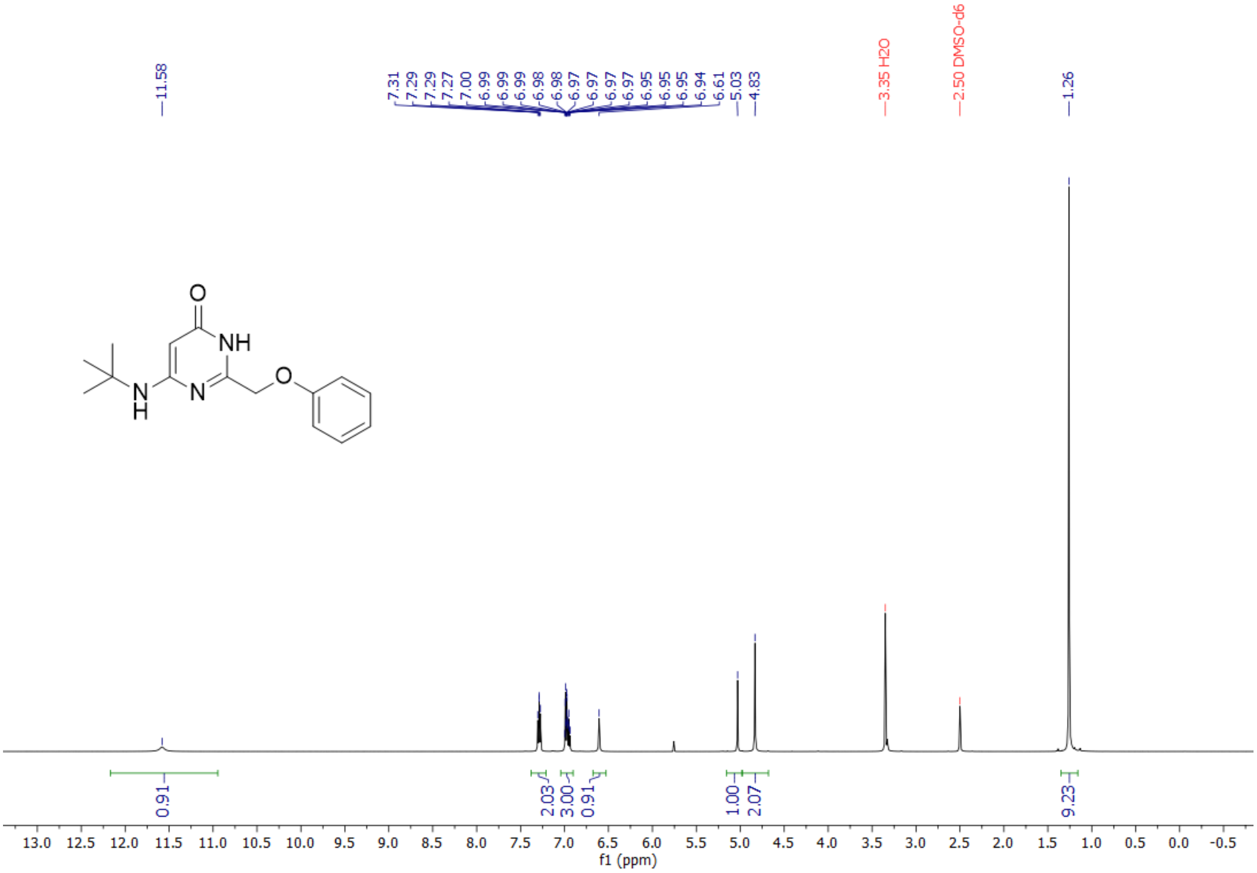

^{13}C-NMR 125.7 MHz, DMSO-*d*6 **4ah**

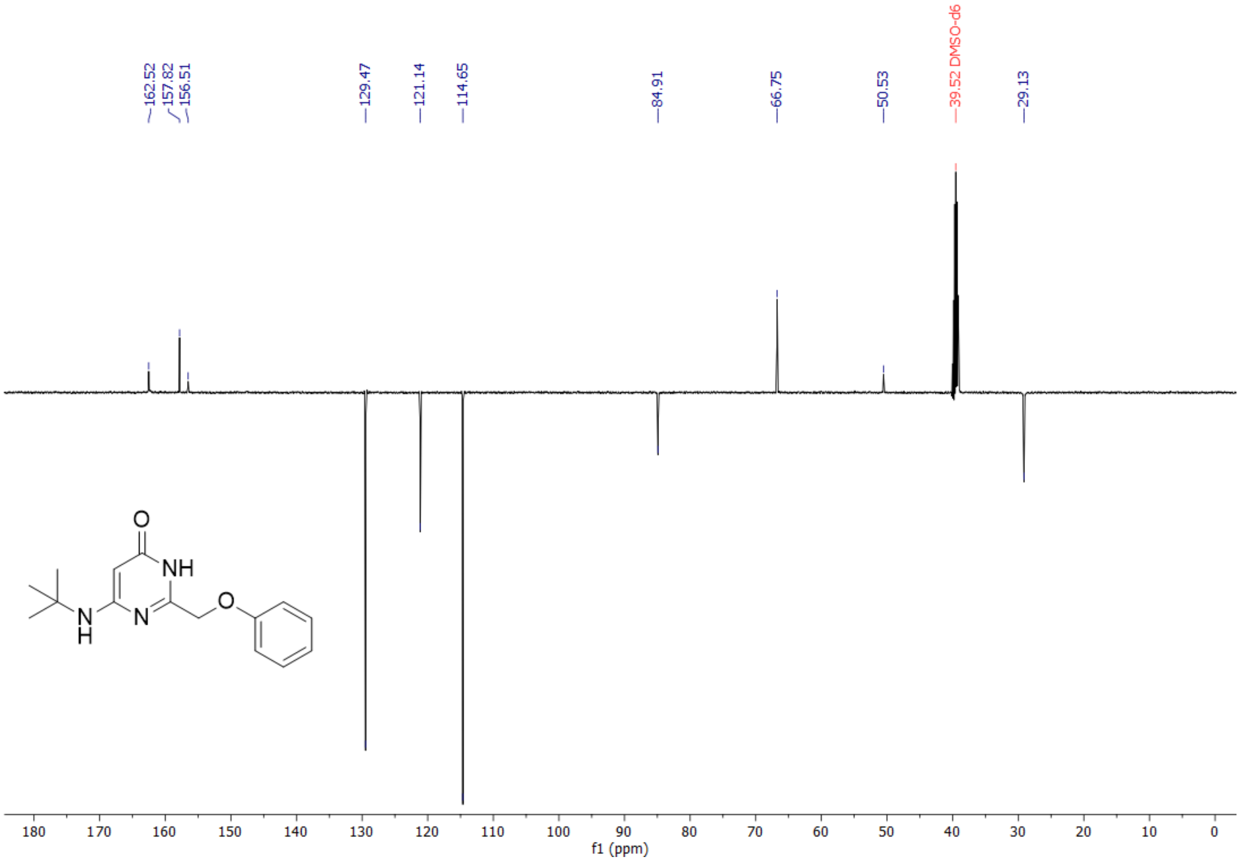

^{1}H-NMR 500 MHz, DMSO-*d6* **4ai**

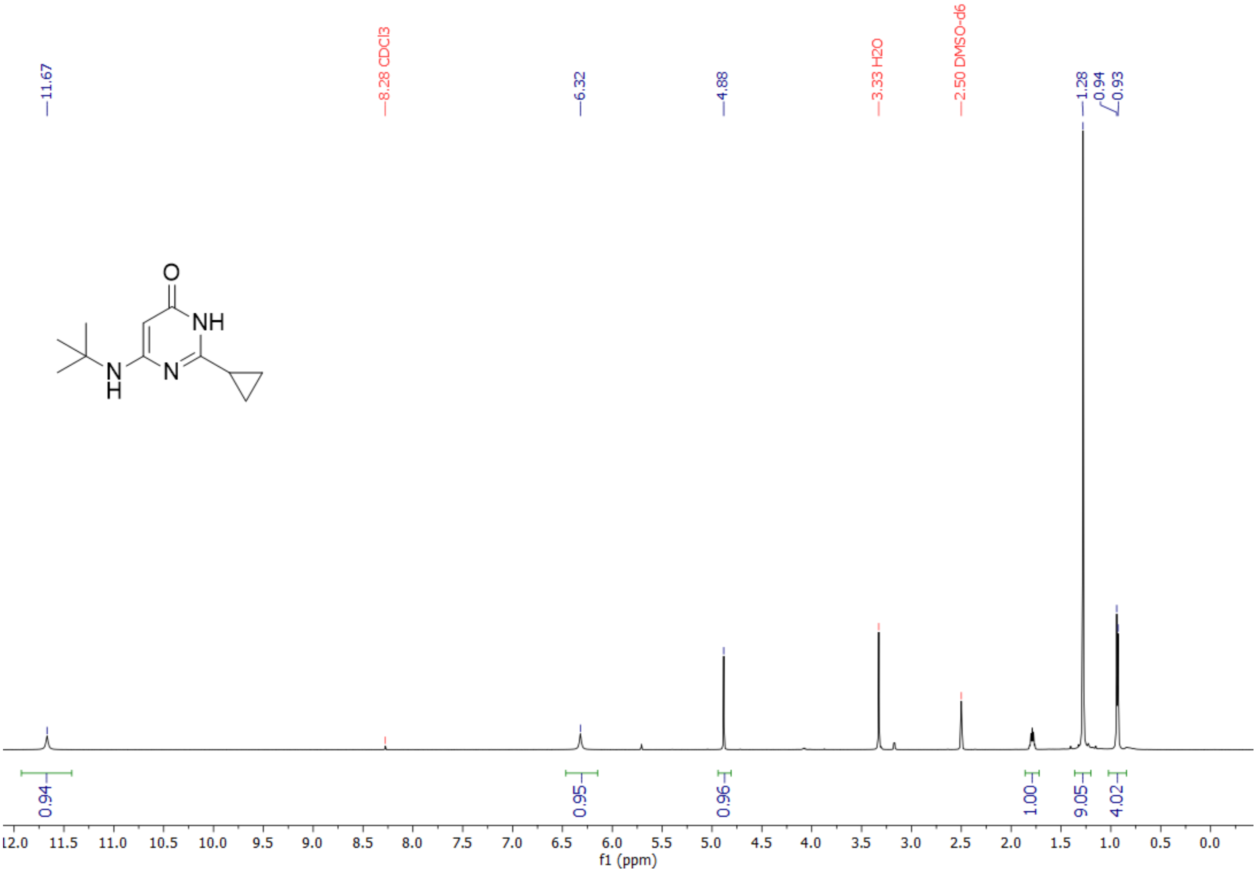

^{13}C-NMR 125.7 MHz, DMSO-*d6* **4ai**

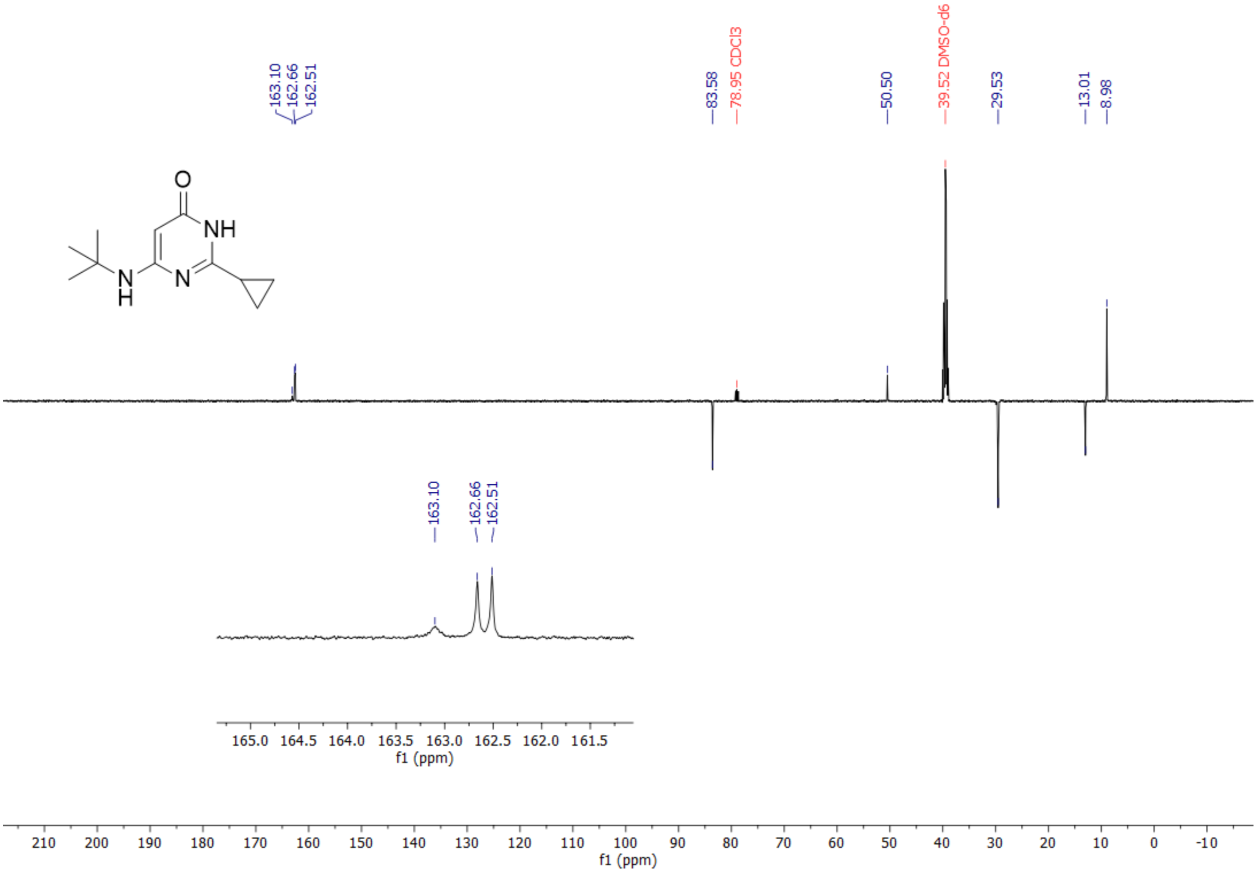

^{1}H-NMR 500 MHz, $CDCl_3$ **6ab**

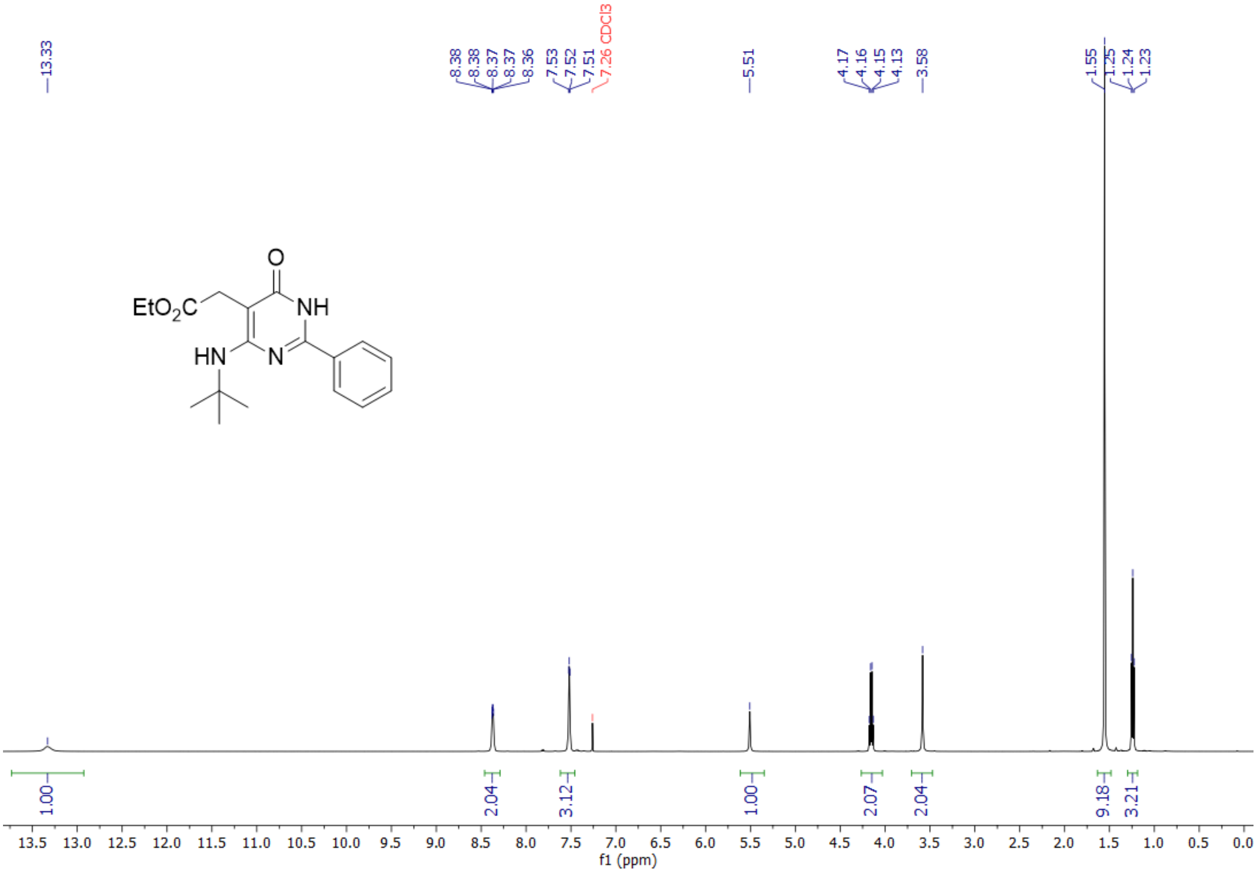

^{13}C-NMR 125.7 MHz, $CDCl_3$ **6ab**

TRR_318_B.4.fid

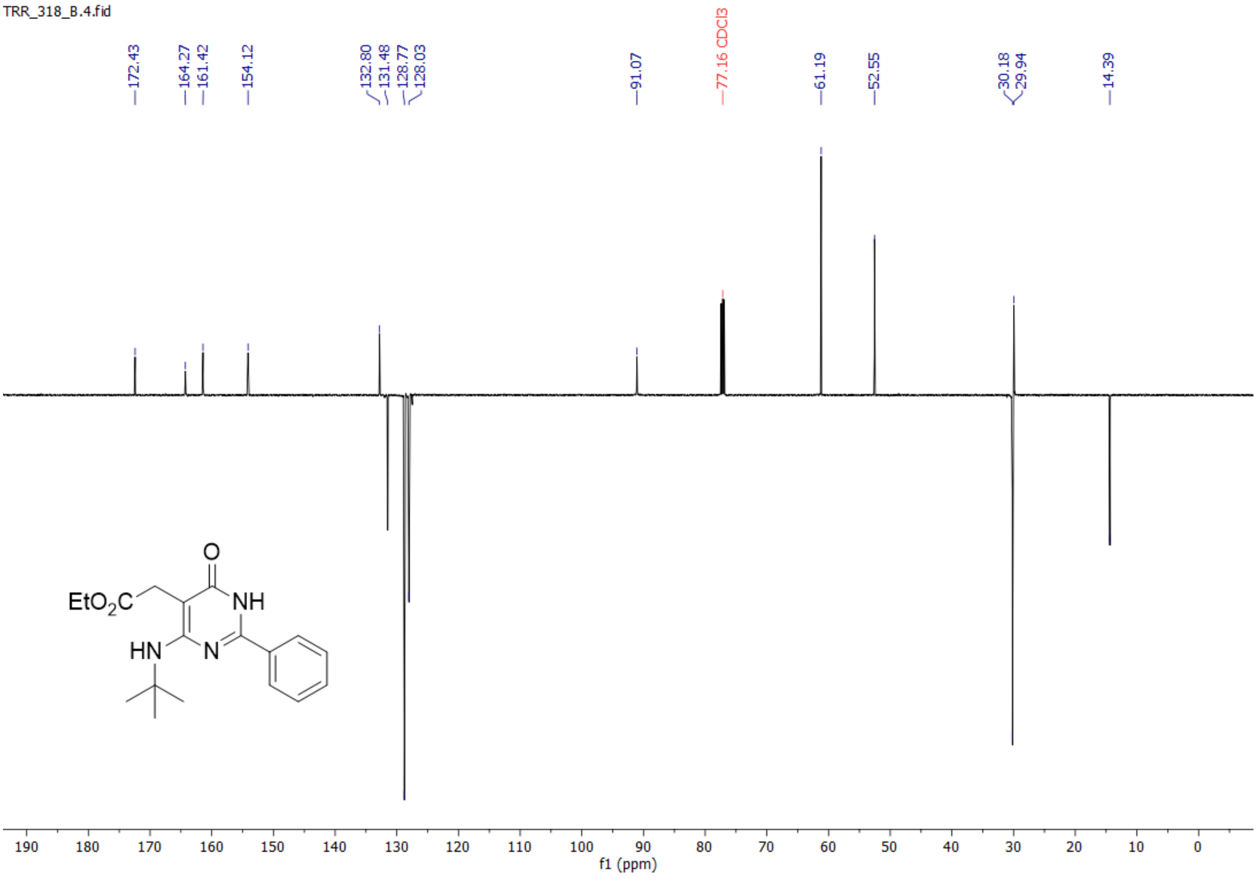

^{1}H-NMR 500 MHz, $CDCl_3$ **6ac**

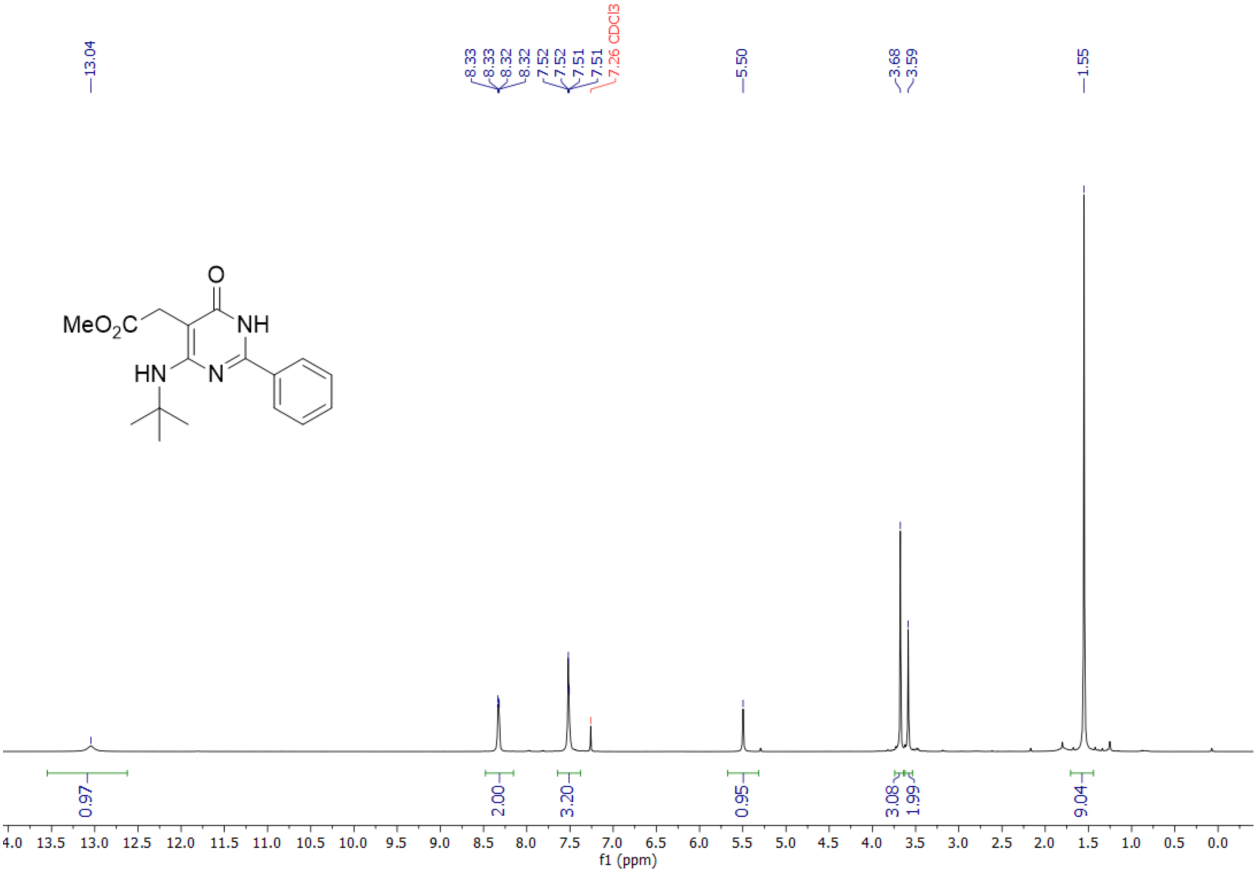

^{13}C-NMR 125.7 MHz, $CDCl_3$ **6ac**

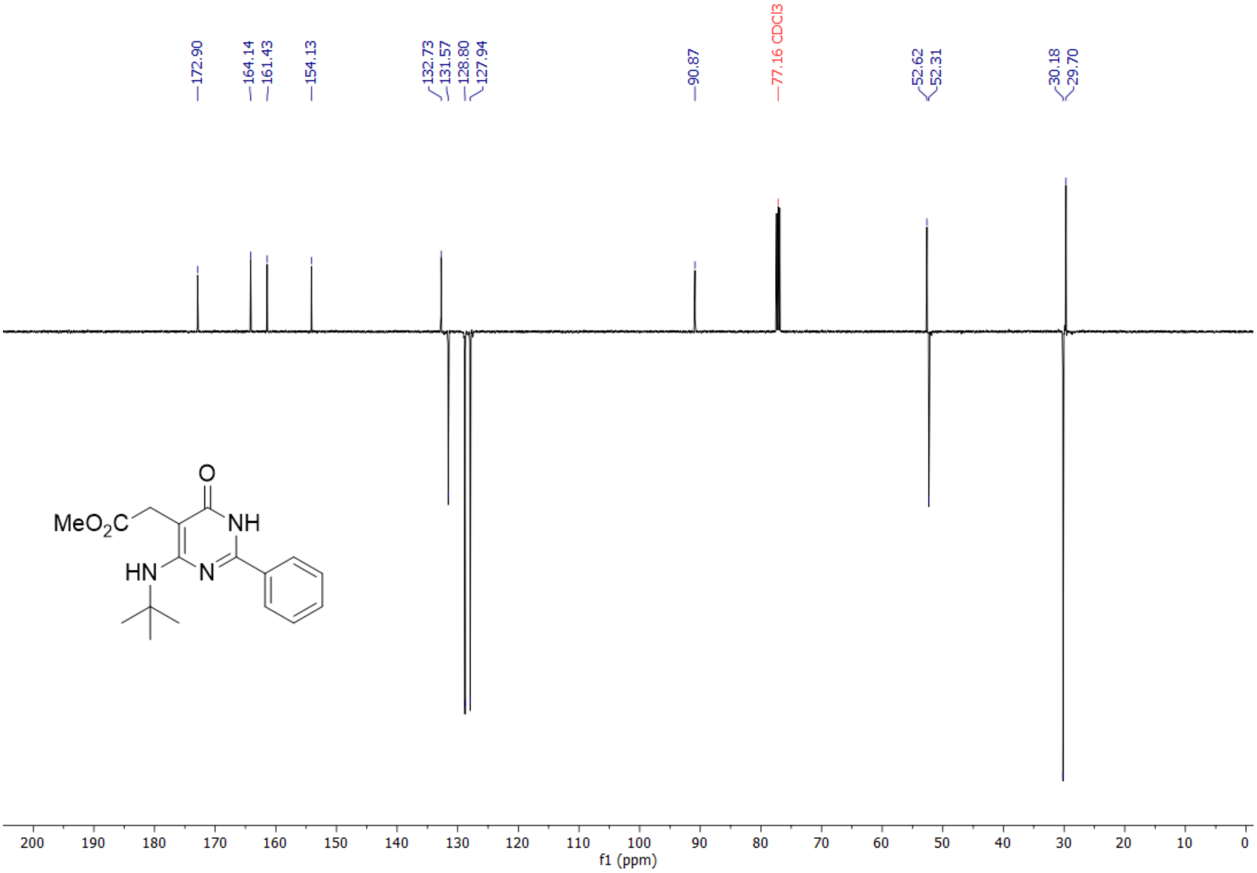

^{1}H-NMR 500 MHz, $CDCl_3$ **6ad**

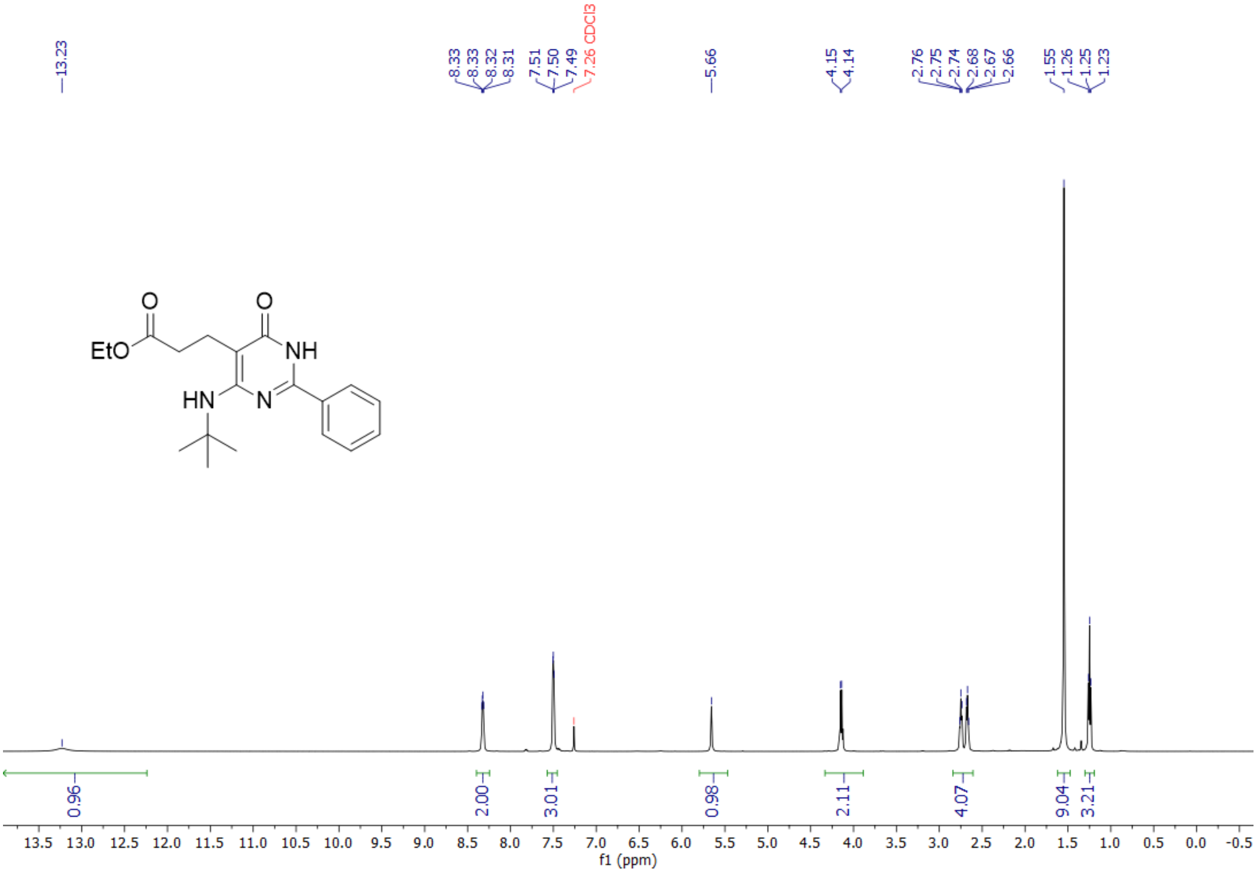

^{13}C-NMR 125.7 MHz, $CDCl_3$ **6ad**

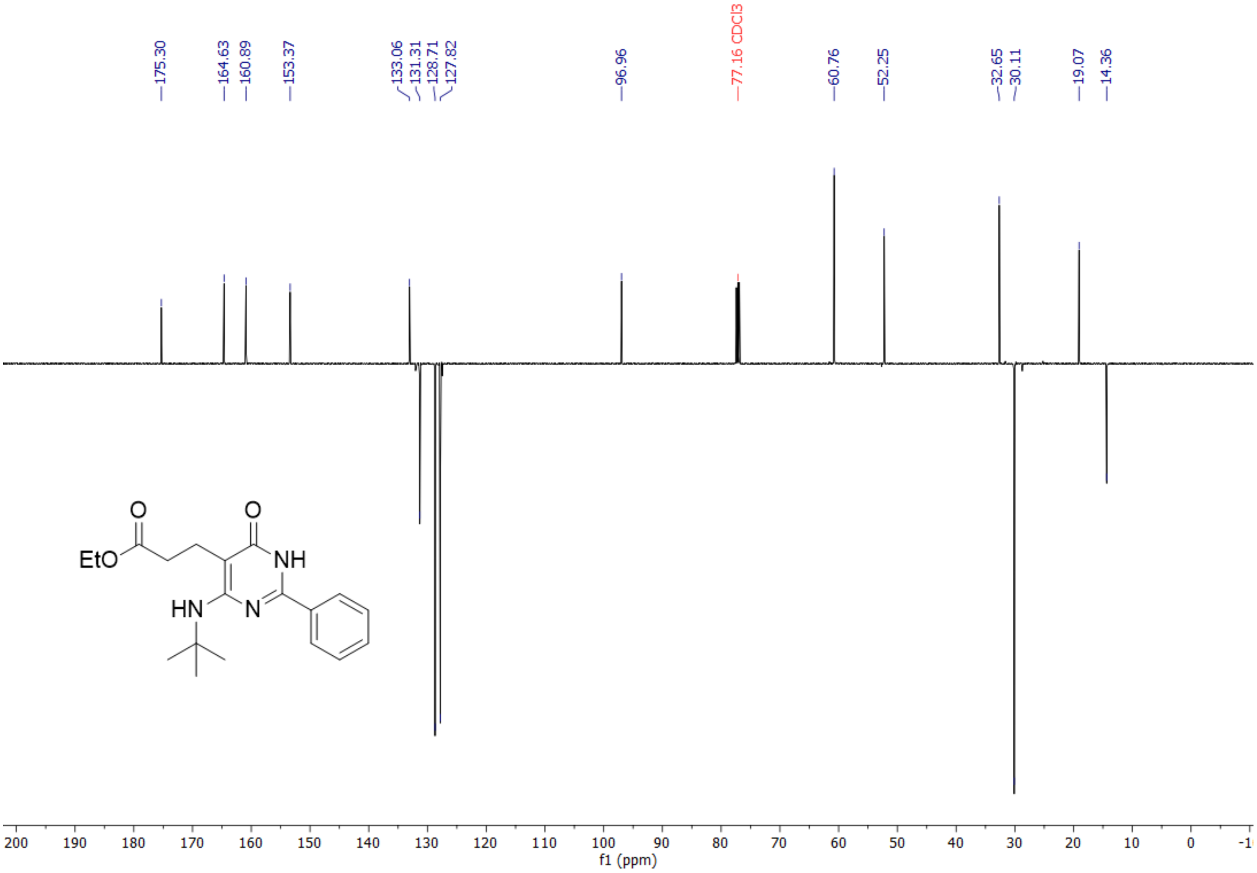

^{1}H-NMR 500 MHz, $CDCl_3$ **6ae**

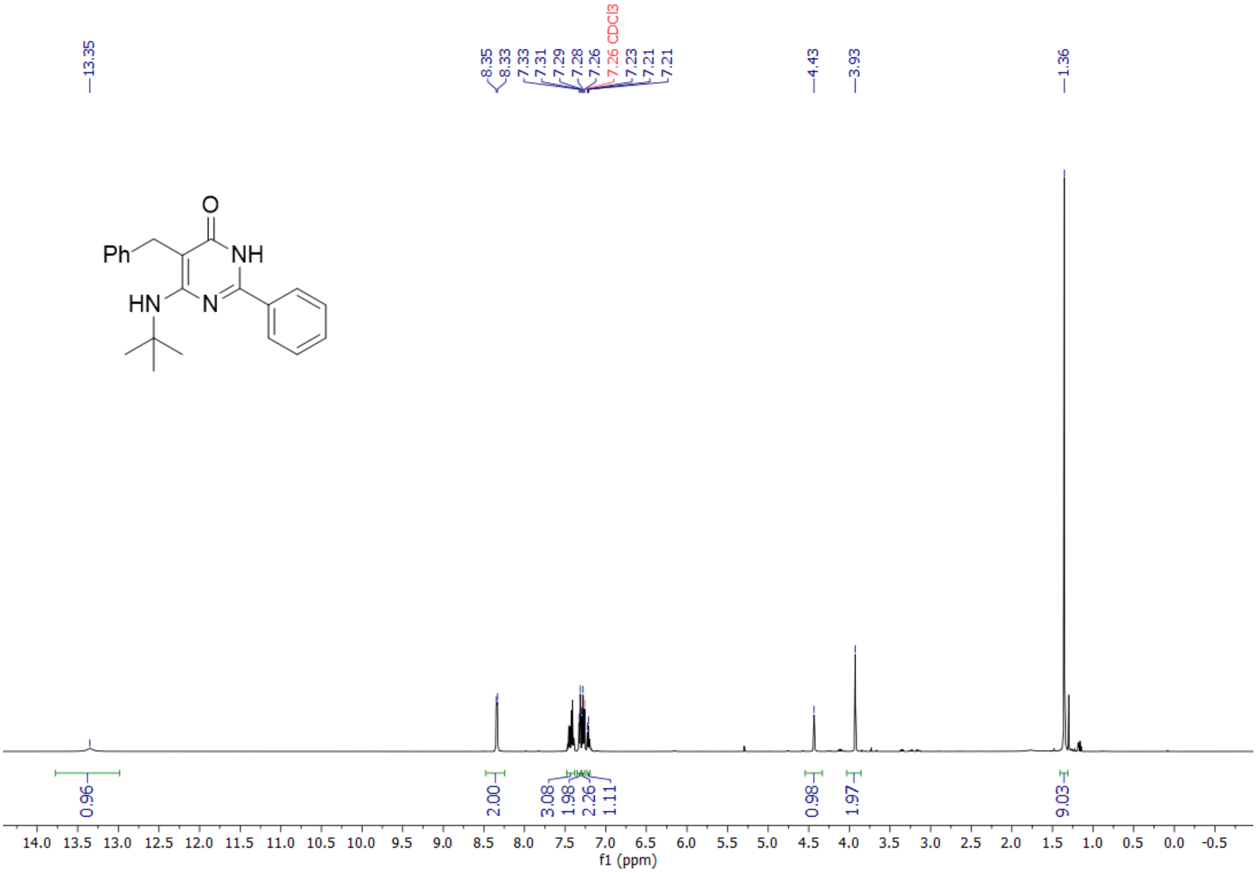

^{13}C-NMR 125.7 MHz, $CDCl_3$ **6ae**

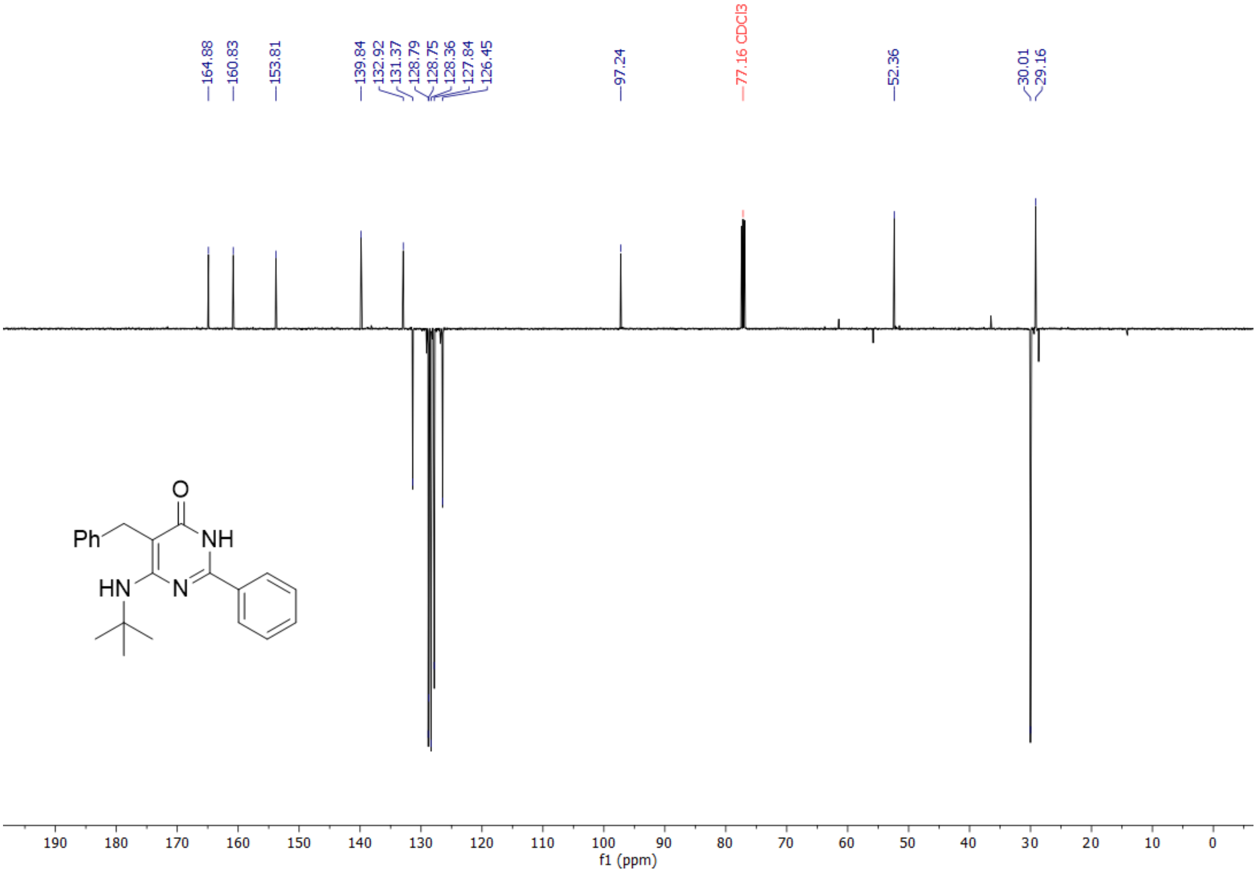

^{1}H-NMR 500 MHz, DMSO-*d*6 @80 °C, **6ag**

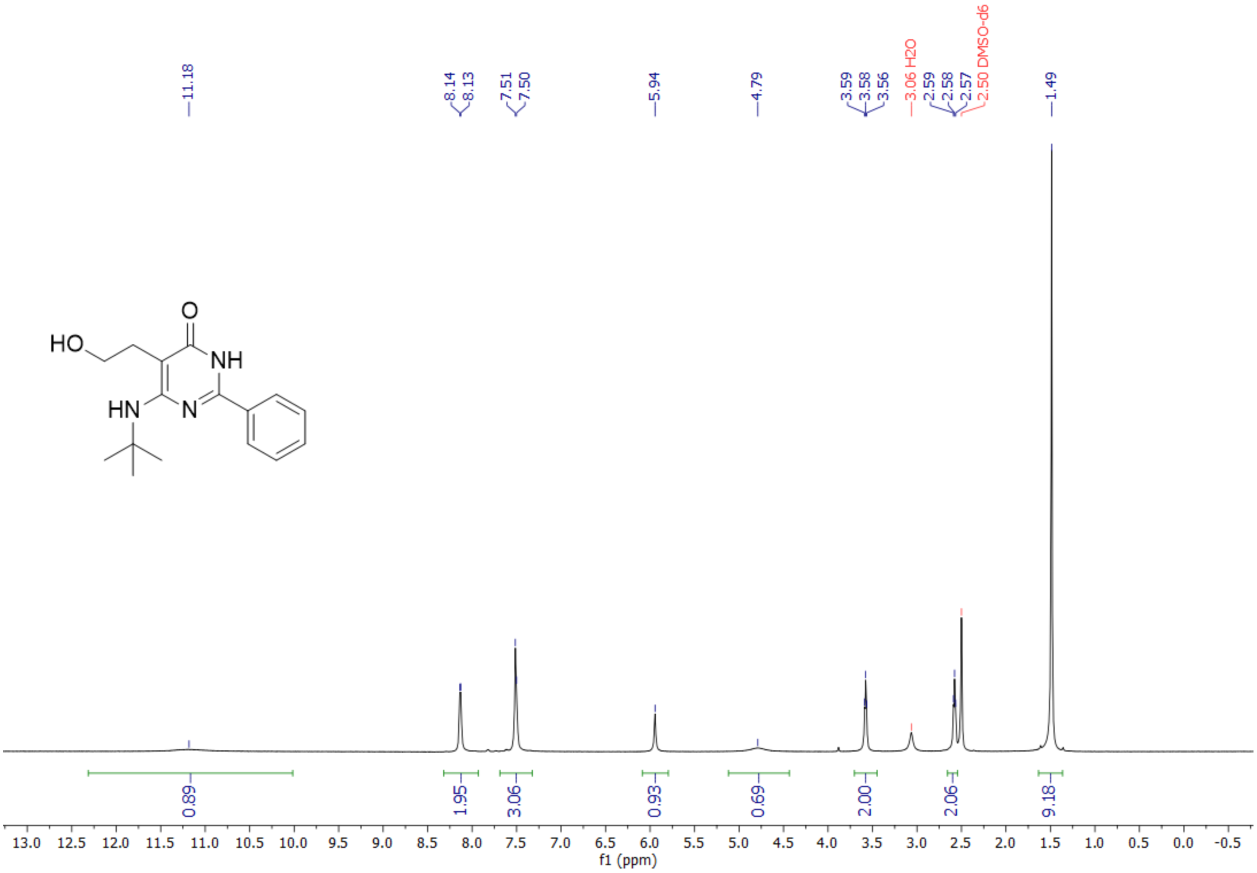

^{13}C-NMR 125.7 MHz, DMSO-*d*6 **6ag**

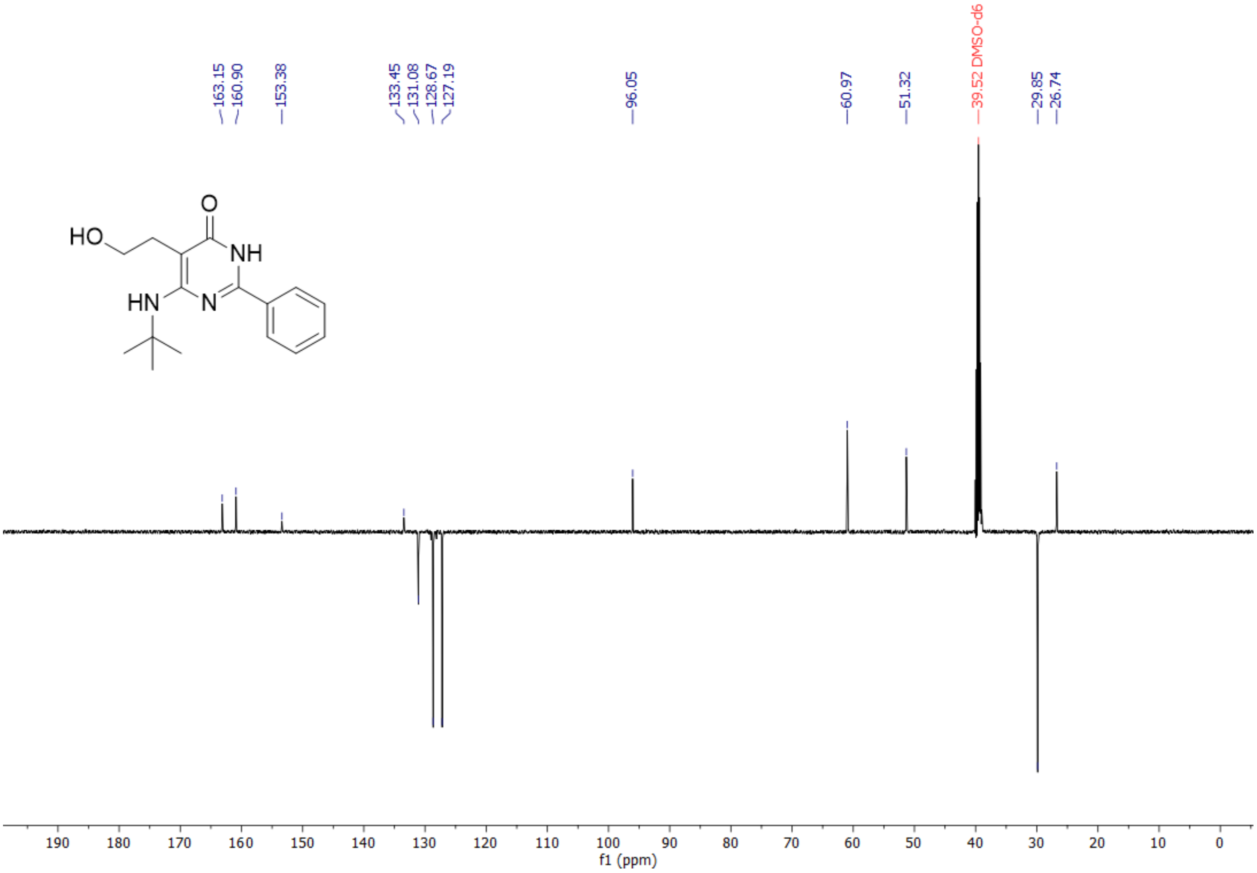

^{1}H-NMR 500 MHz, $CDCl_3$ **6af**

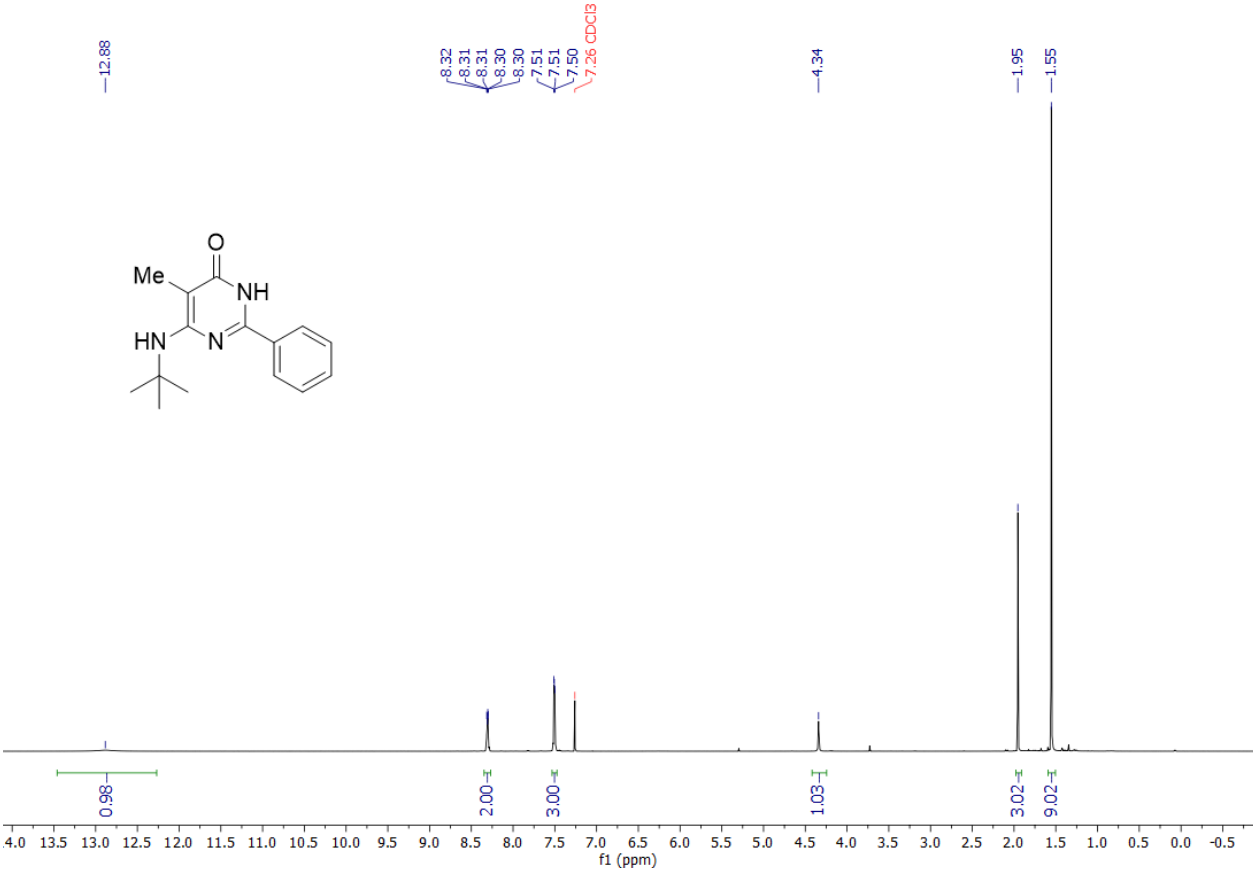

^{13}C-NMR 125.7 MHz, $CDCl_3$ **6af**

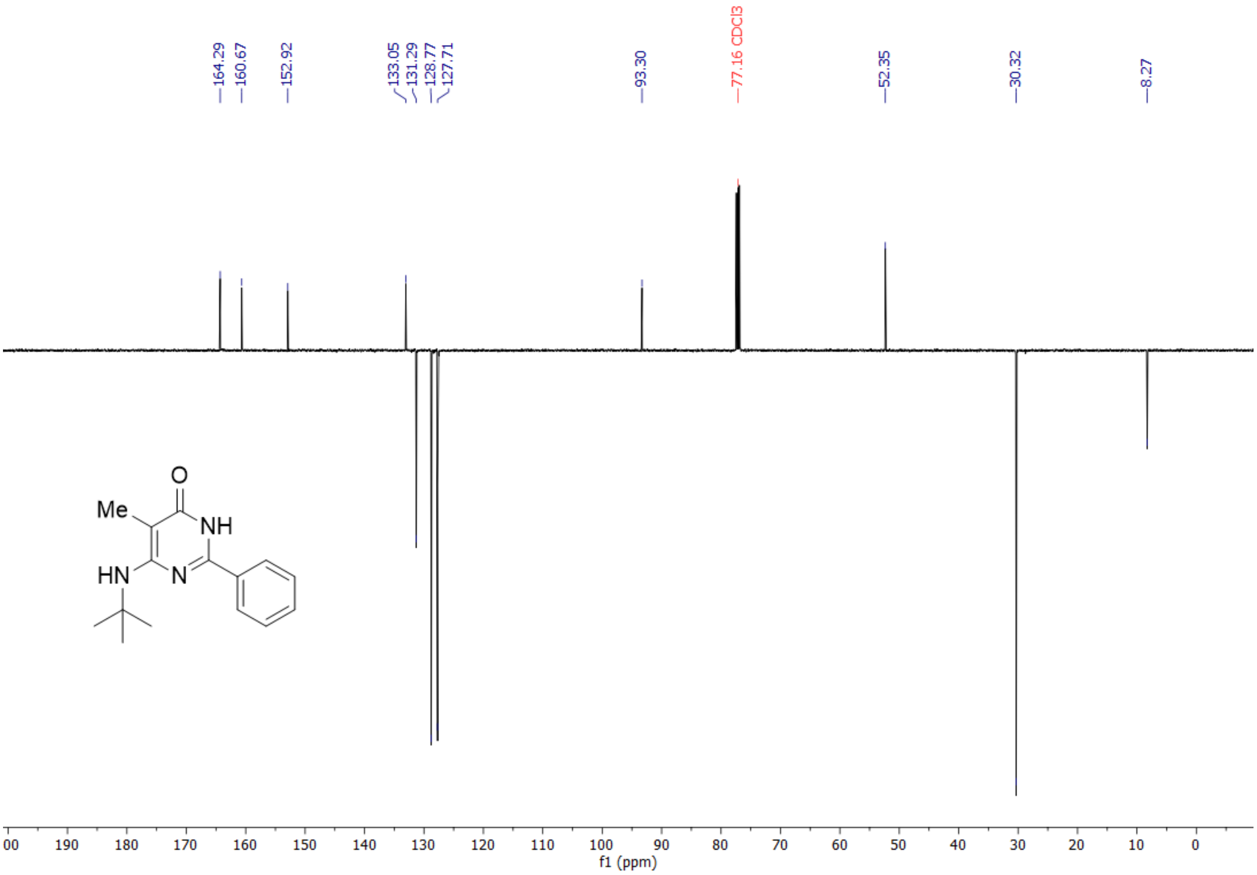

^{1}H-NMR 500 MHz, $CDCl_3$ **7aj**

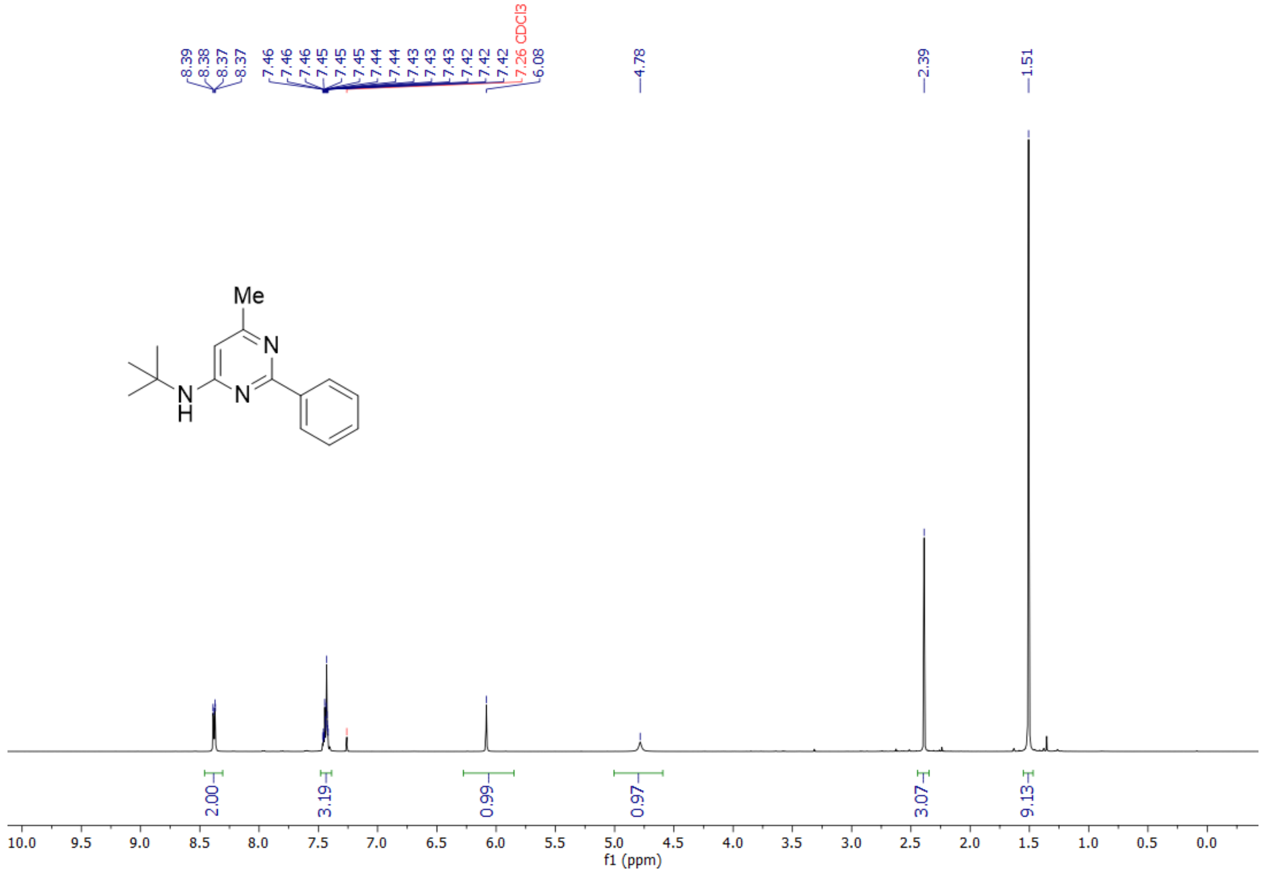

^{13}C-NMR 125.7 MHz, $CDCl_3$ **7aj**

TRR_313.4.fid

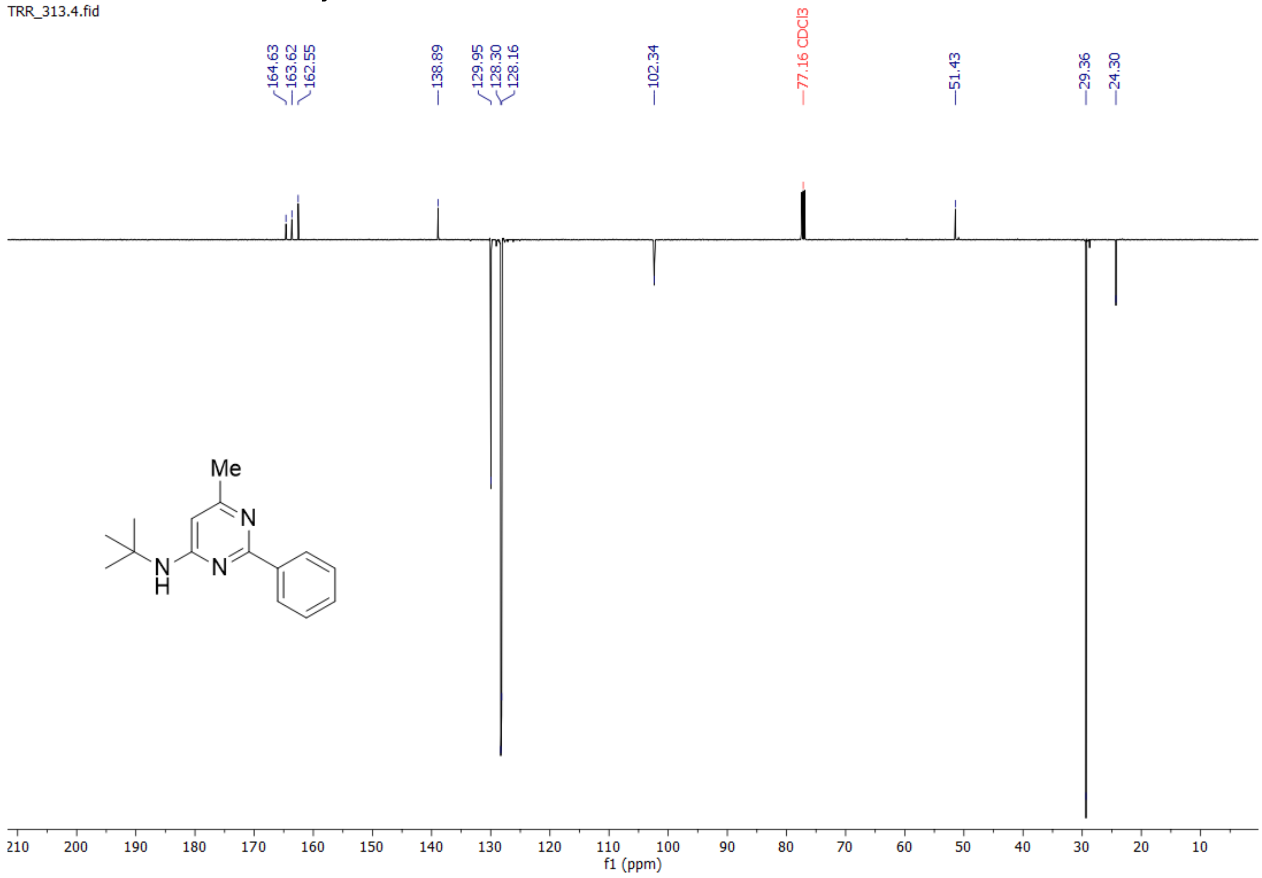

^{1}H-NMR 500 MHz, $CDCl_3$ **7al**

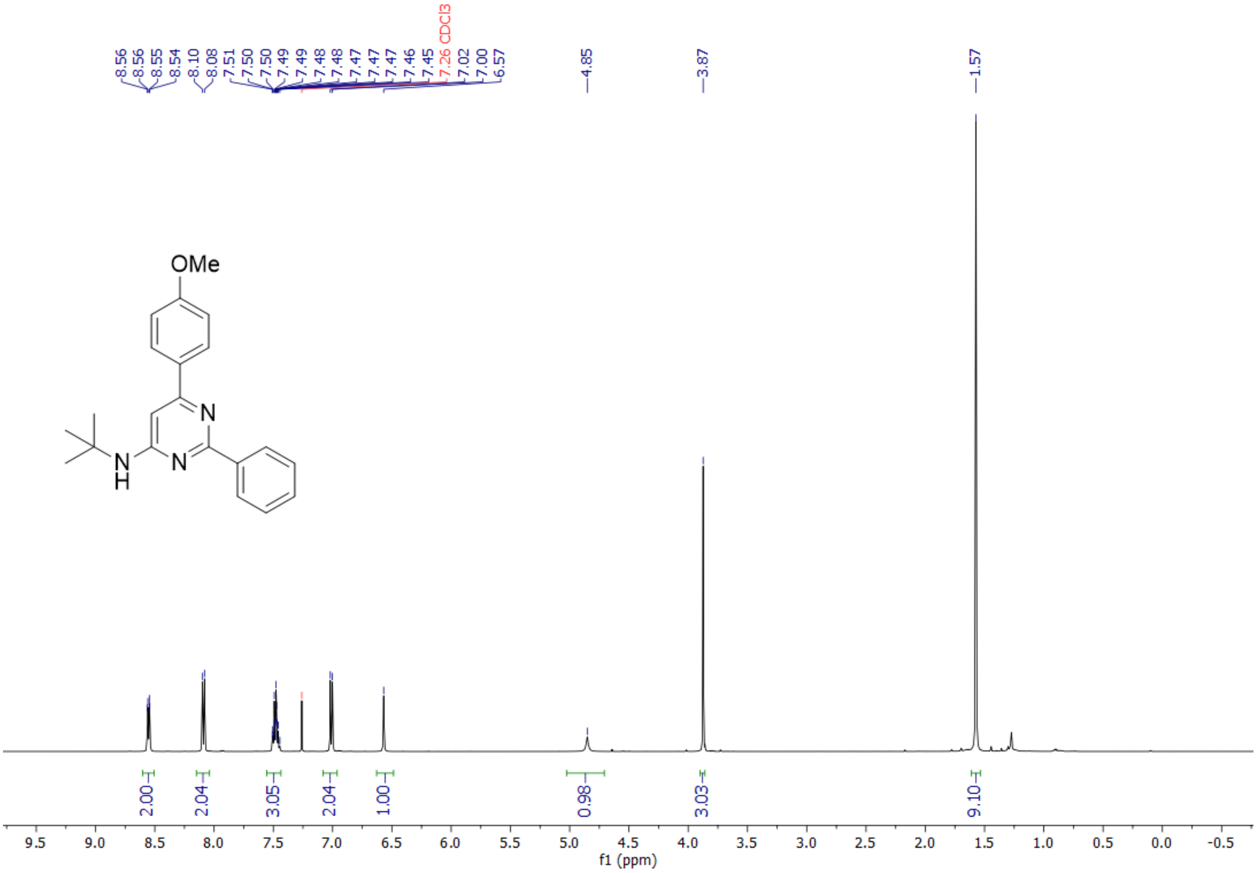

^{13}C-NMR 125.7 MHz, $CDCl_3$ **7al**

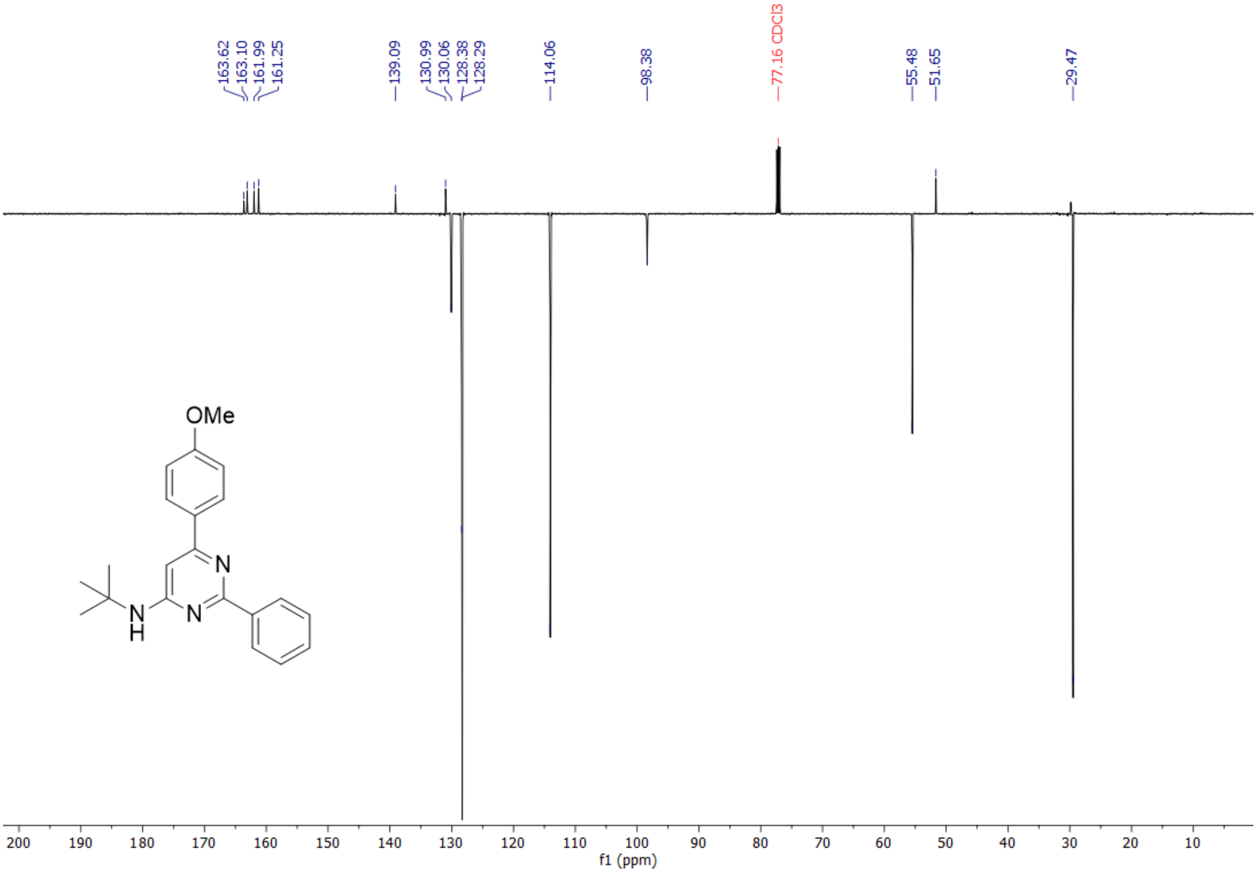

^{1}H-NMR 500 MHz, $CDCl_3$ **7ak**

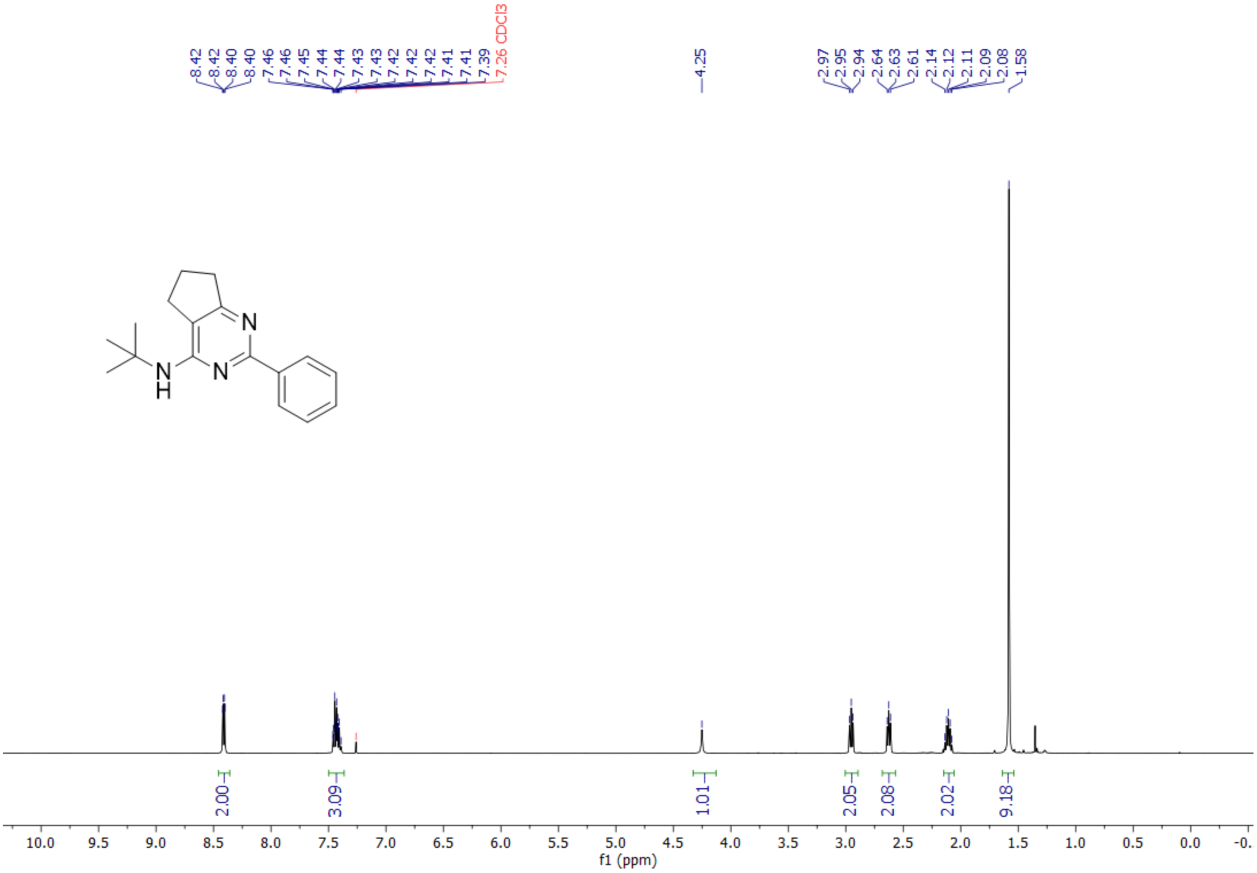

^{13}C-NMR 125.7 MHz, $CDCl_3$ **7ak**

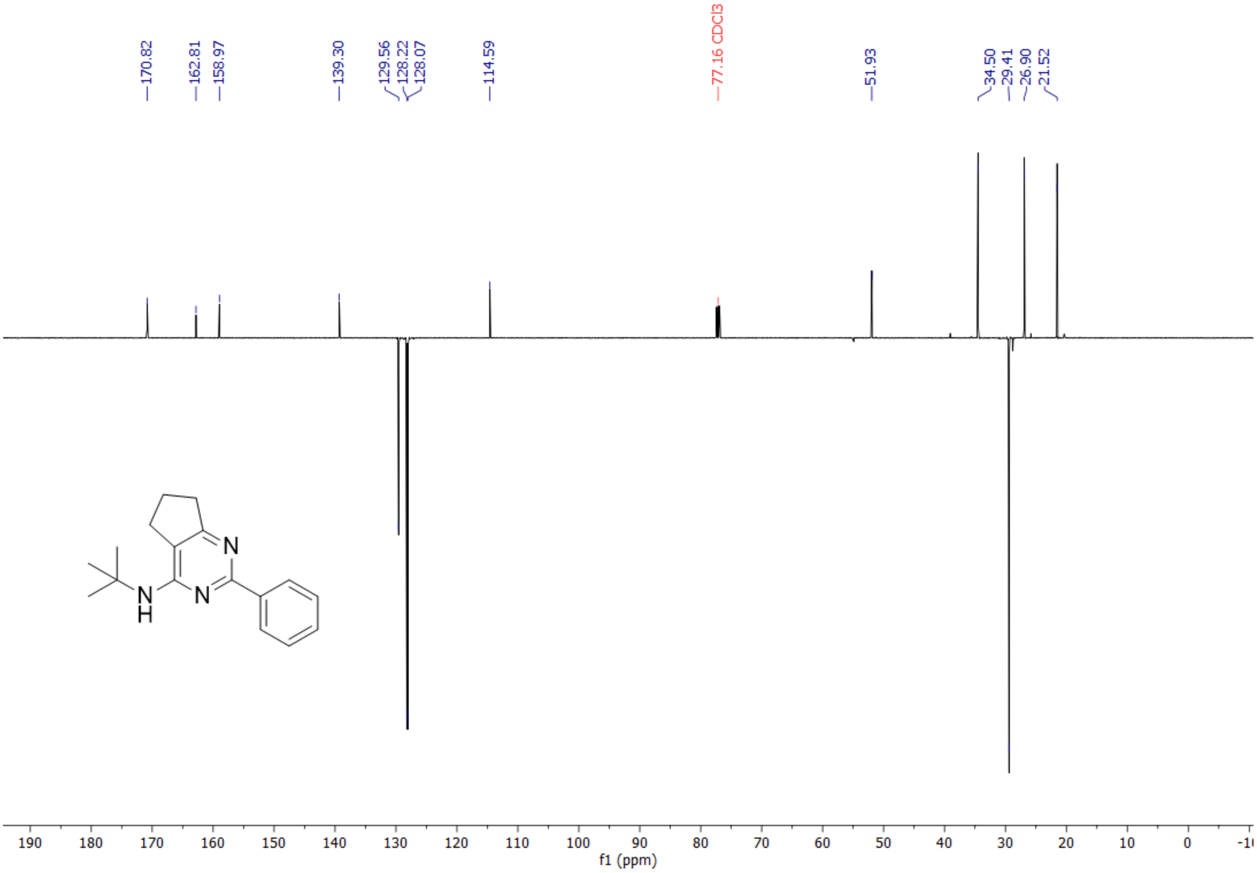

^{1}H-NMR 600 MHz, $CDCl_3$ **7am**

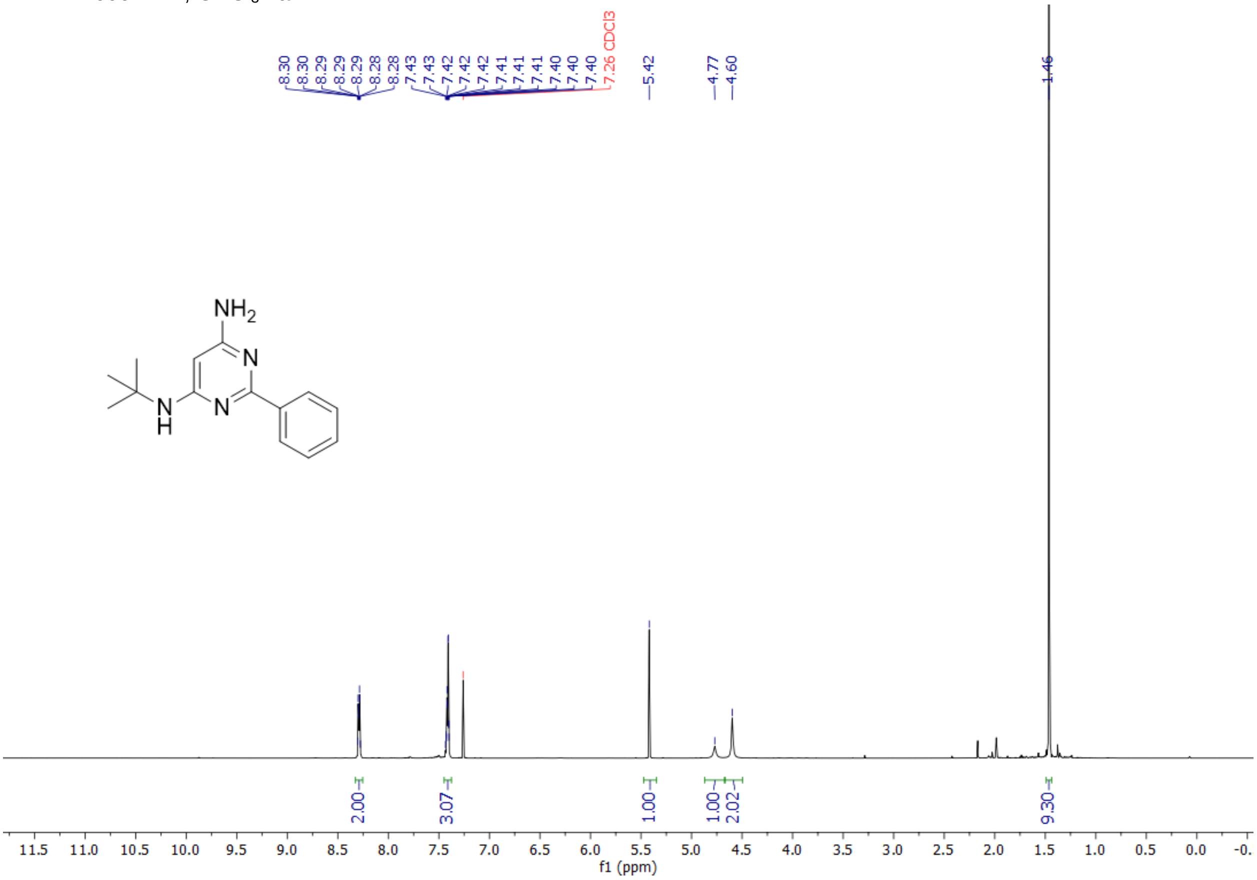

^{13}C-NMR 151 MHz, $CDCl_3$ **7am**

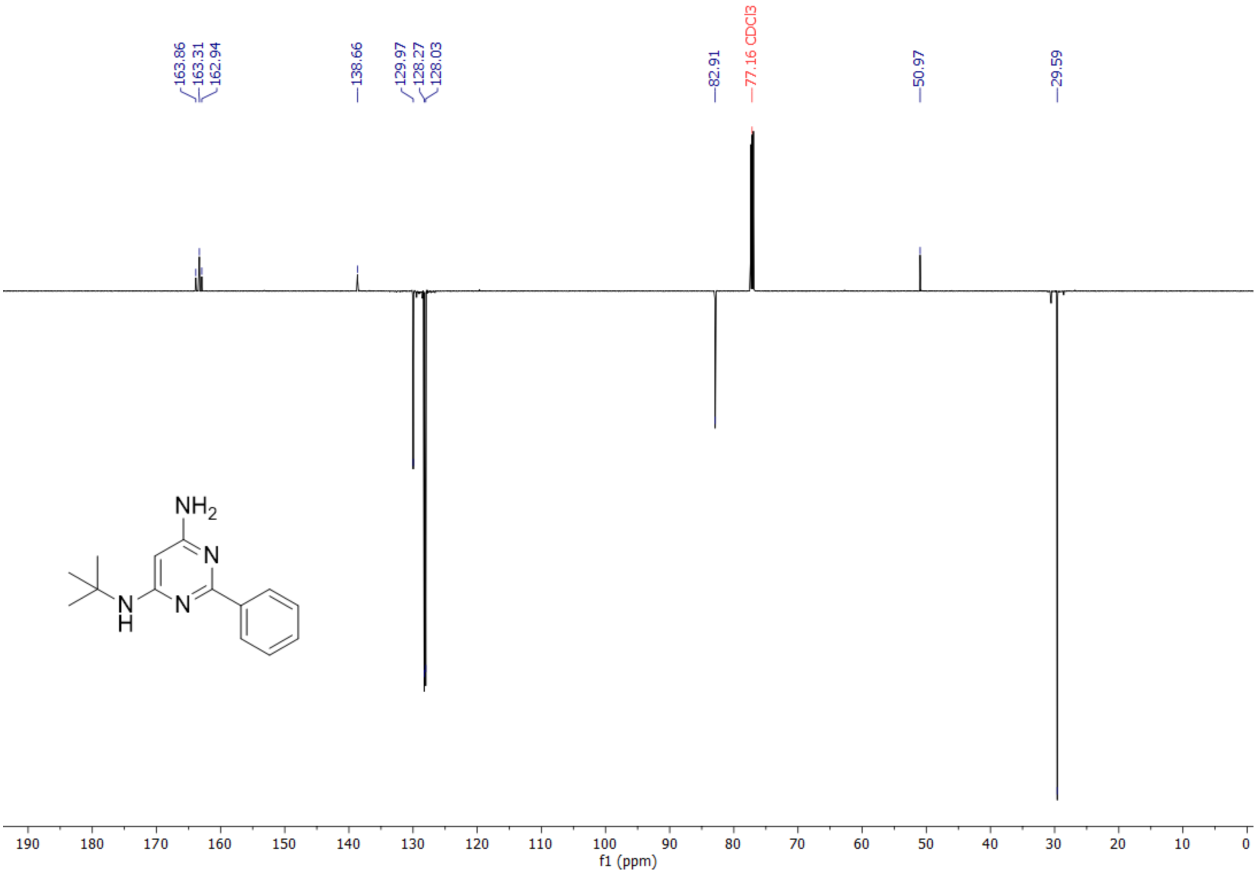

^{1}H-NMR 500 MHz, $CDCl_3$ **9a**

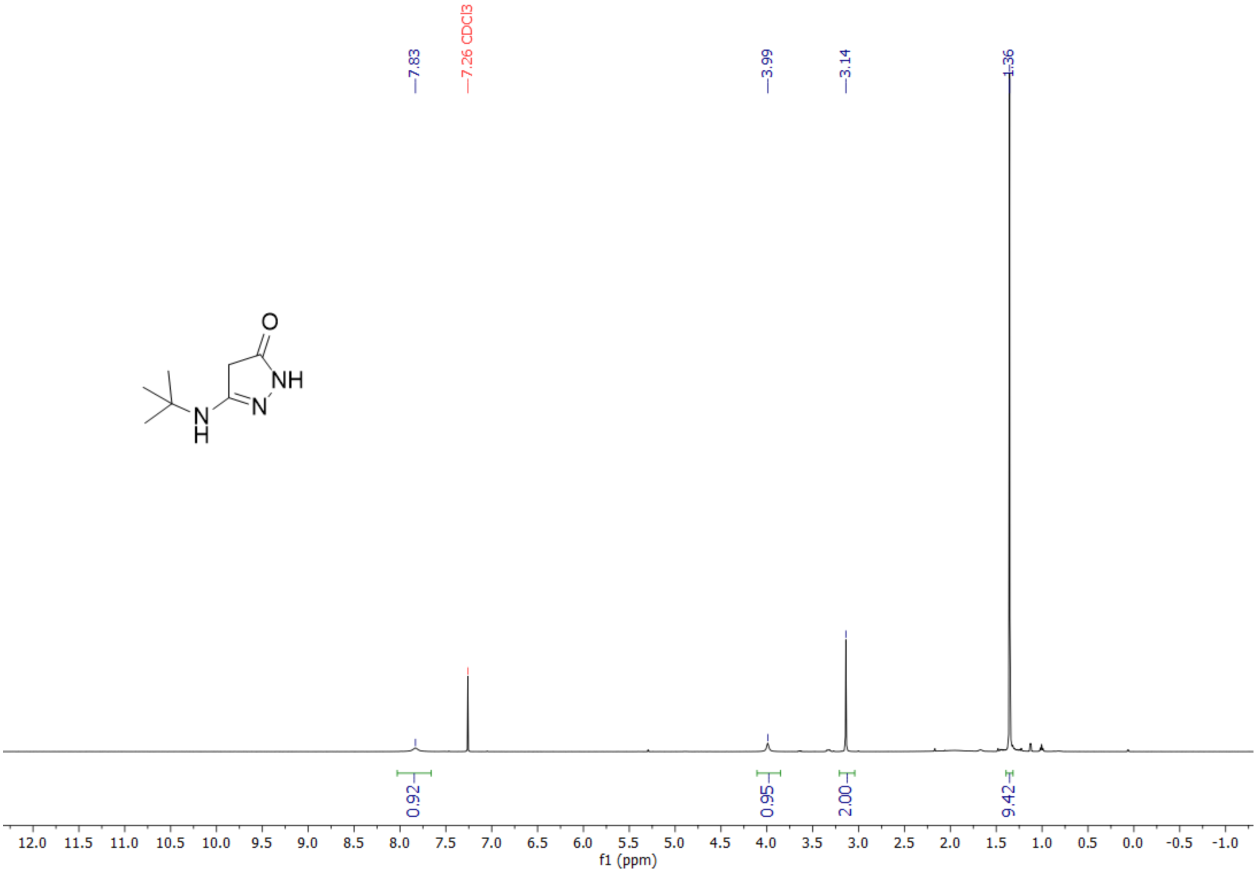

^{13}C-NMR 125.7 MHz, $CDCl_3$ **9a**

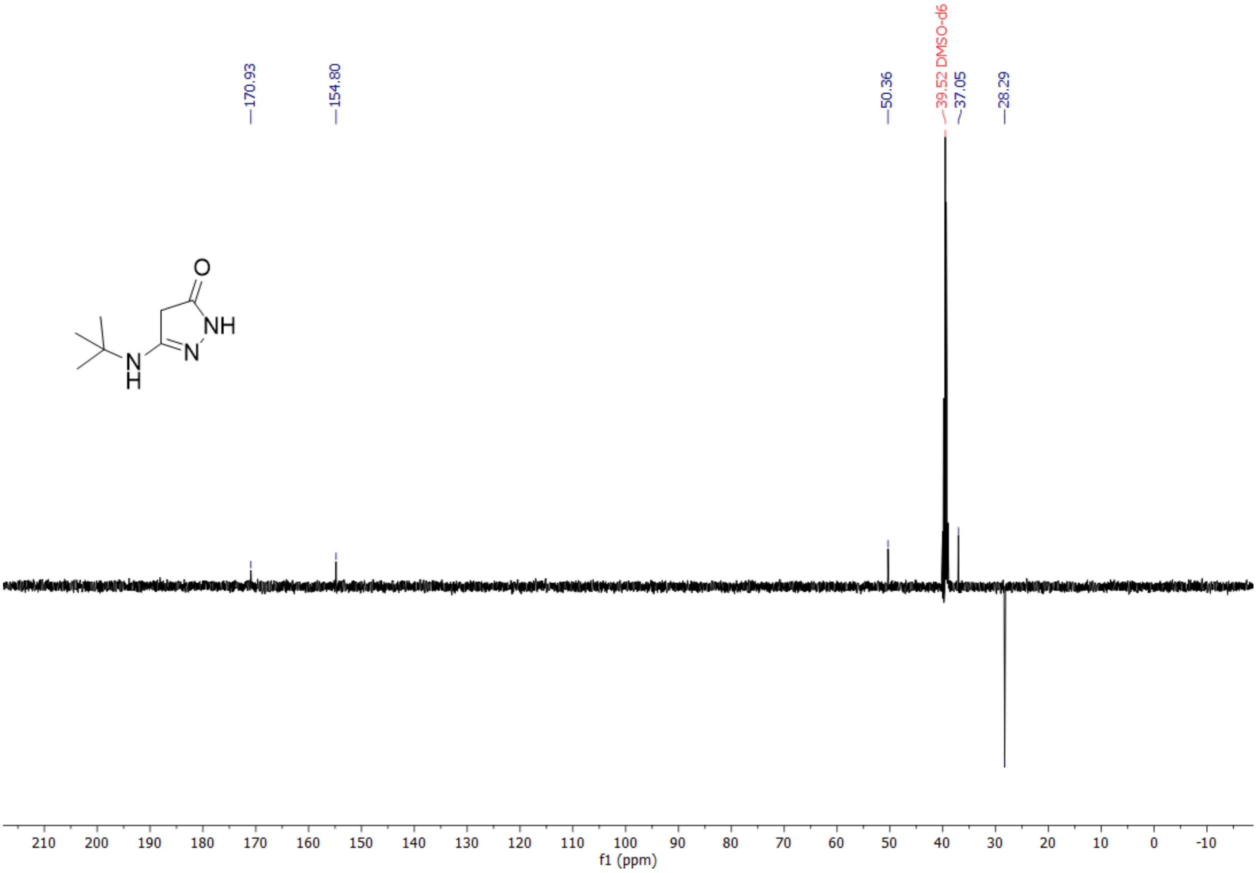

^{1}H-NMR 500 MHz, $CDCl_3$ **9b**

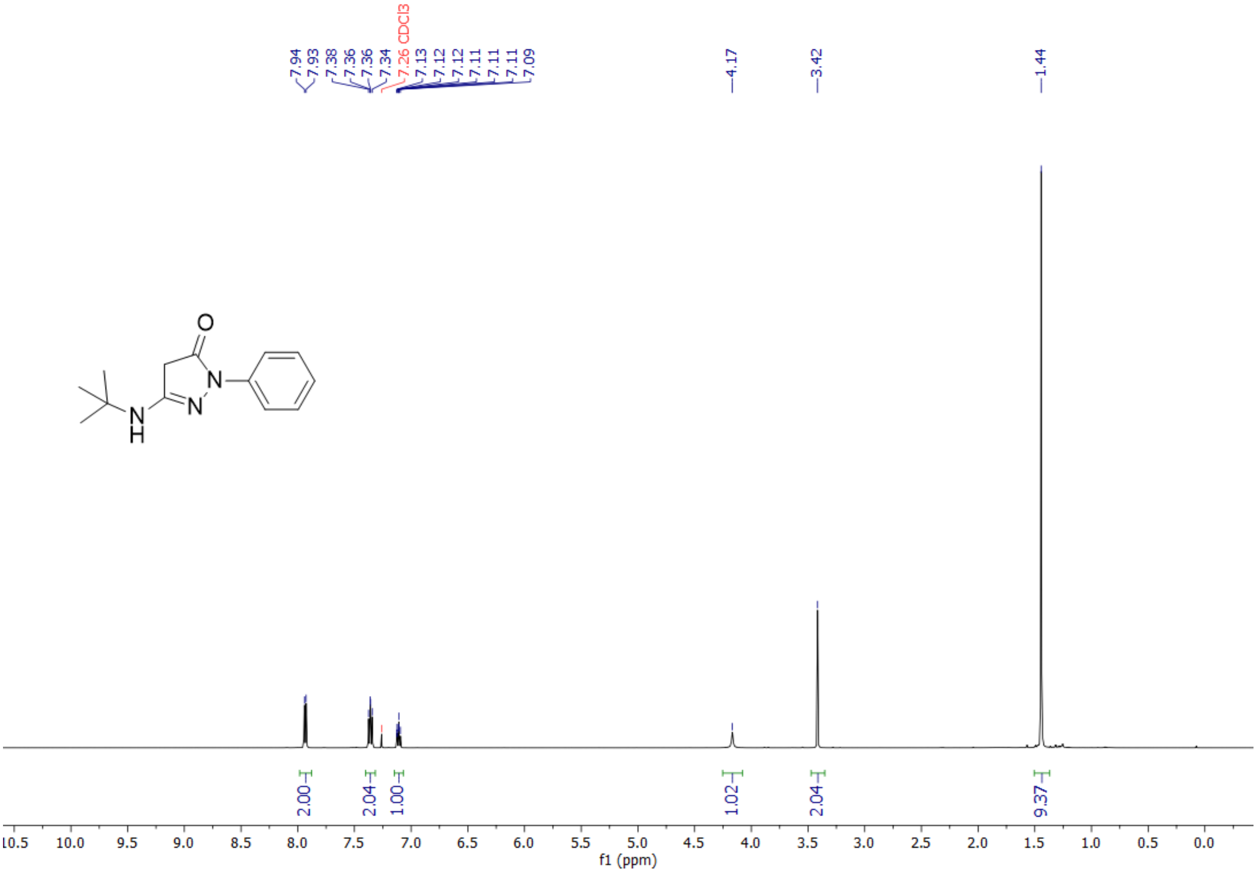

^{13}C-NMR 125.7 MHz, $CDCl_3$ **9b**

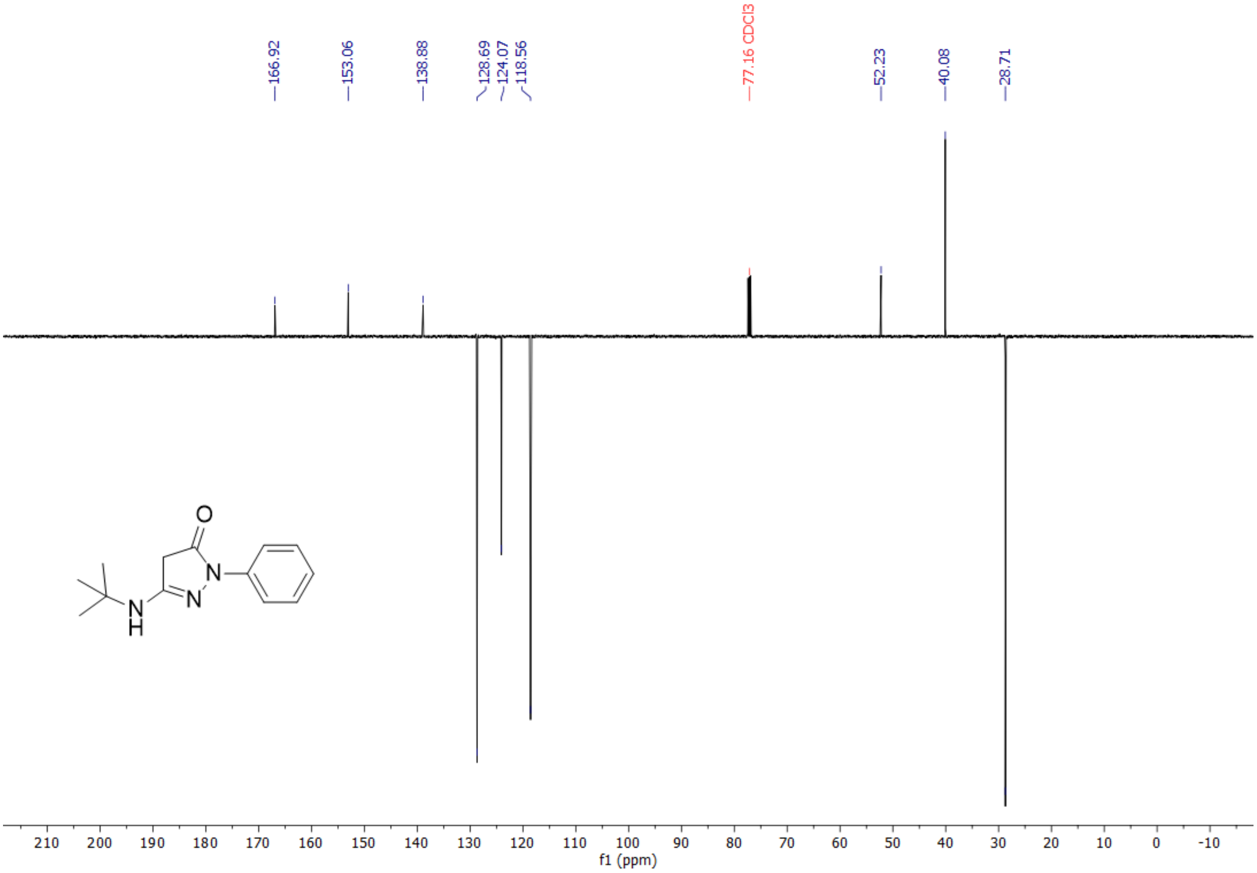

^{1}H-NMR 500 MHz, $CDCl_3$ **10a**

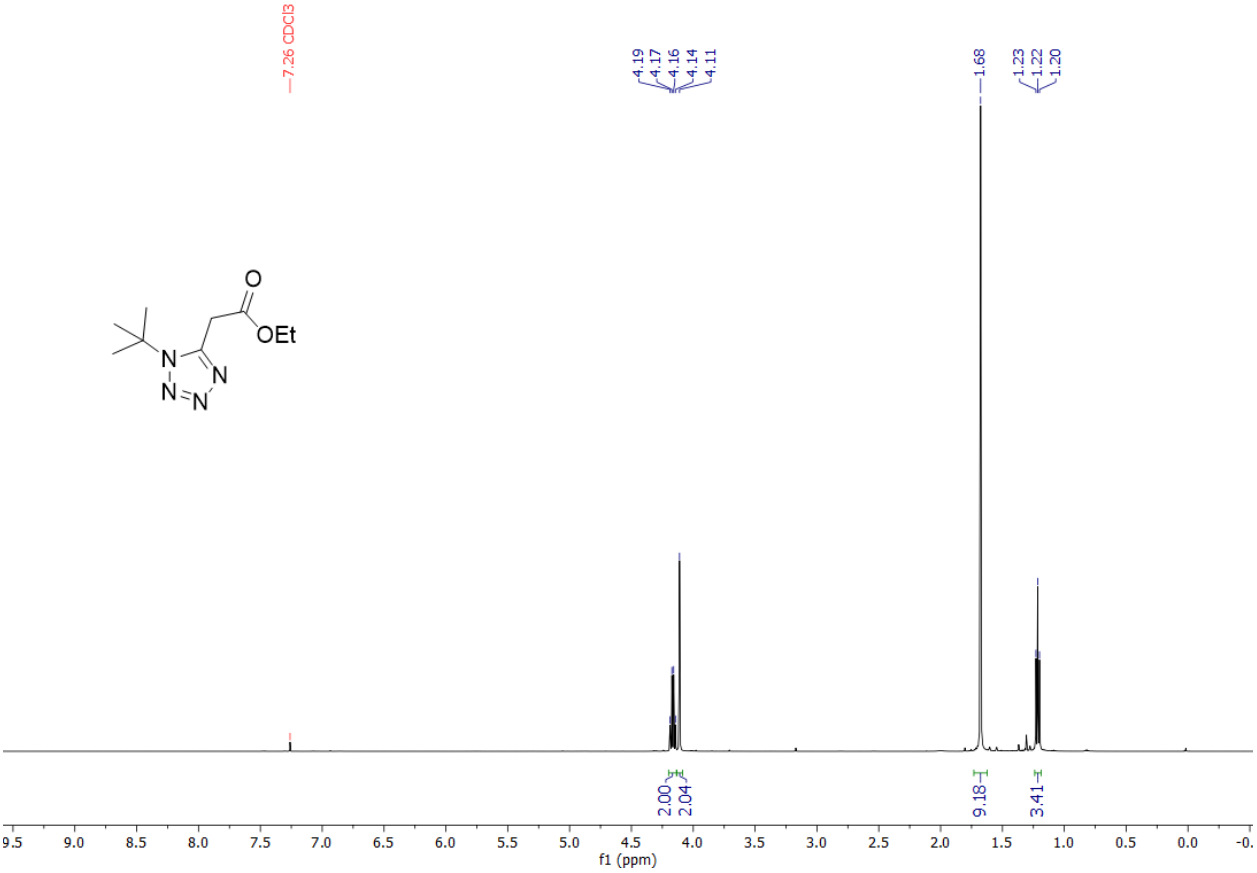

^{13}C-NMR 125.7 MHz, $CDCl_3$ **10a**

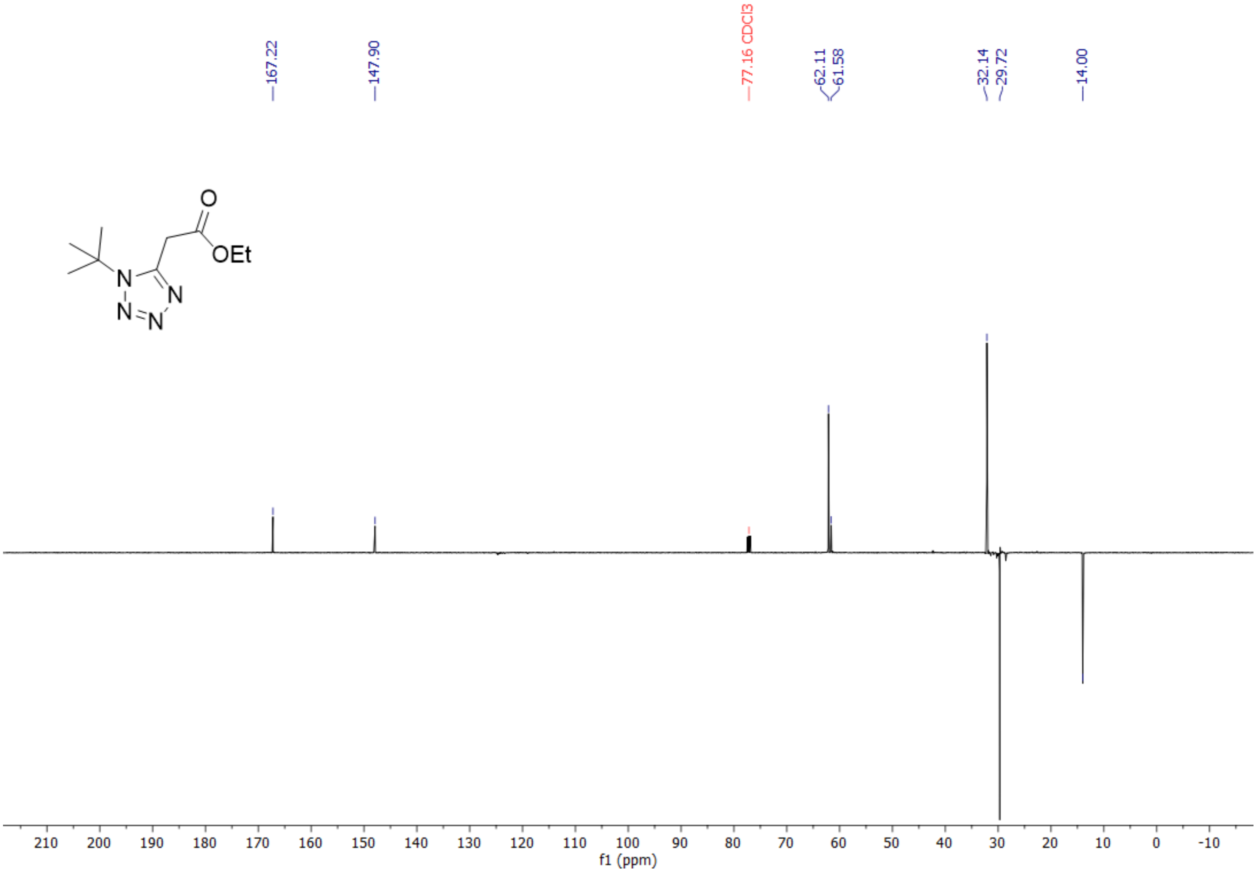

^{1}H-NMR 500 MHz, $CDCl_3$ **10b**

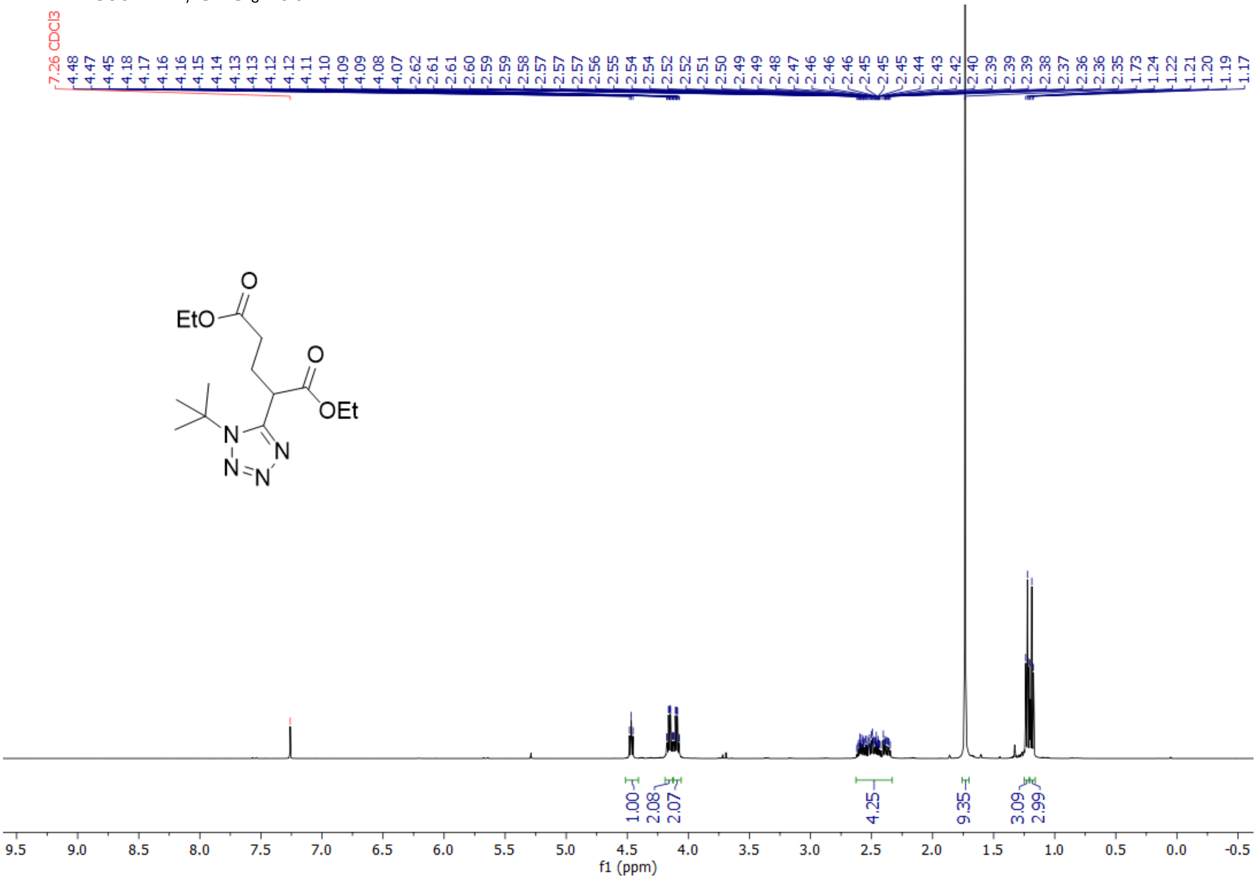

^{13}C-NMR 125.7 MHz, $CDCl_3$ **9b**

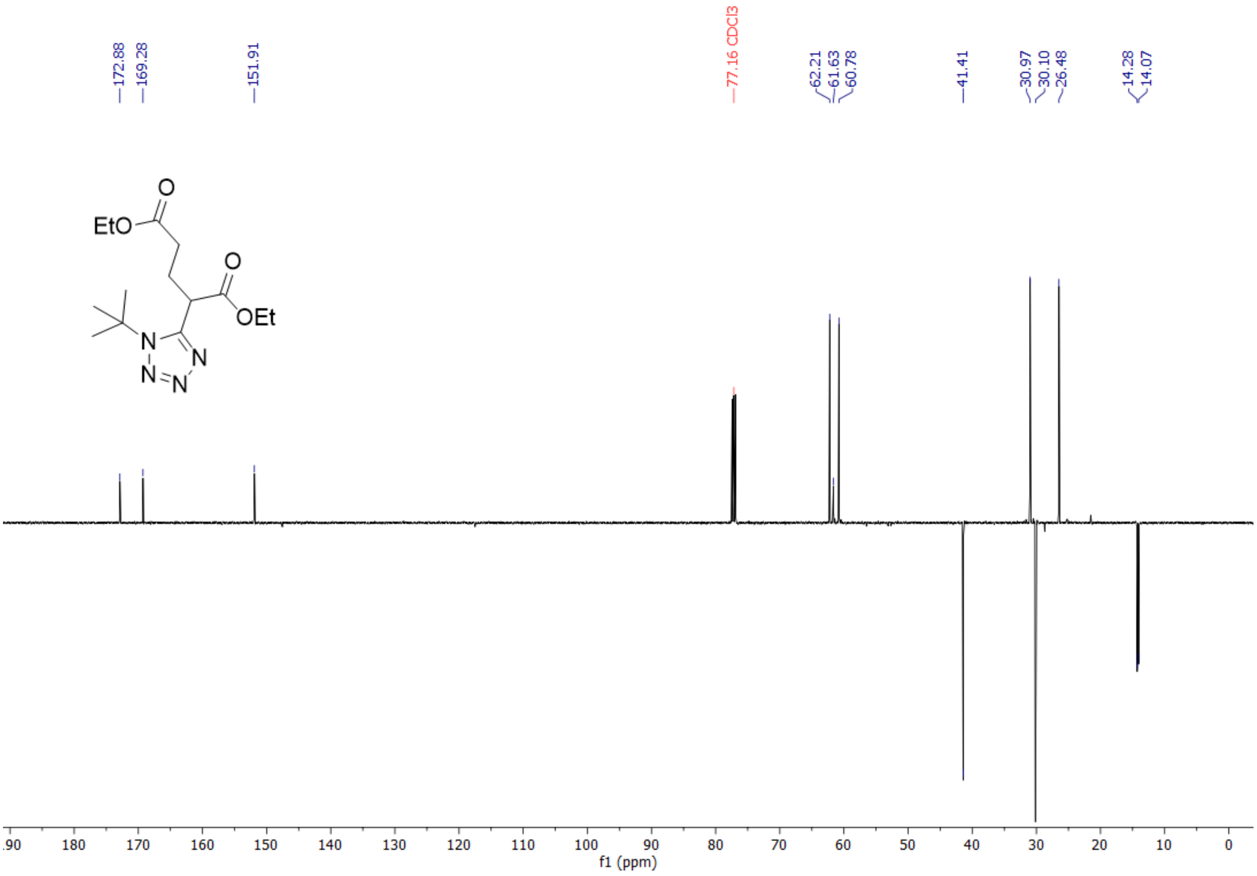

Supplement: Supplementary file 1 — Supporting Information [file CHEM-29-0-s001.pdf]
